# Extracellular cyclophilin A promotes B-cell acute lymphoblastic leukemia progression via MC2R

**DOI:** 10.1038/s44321-026-00466-w

**Published:** 2026-06-10

**Authors:** Han Zhong Pei, Heqiao Li, Hongman Xue, Wenqing Wang, He Zhang, Zeqiu Fan, Xiaoyuan Bai, Fei Han, Qingmei Li, Yuna Zhao, Lu Yu, Xiaoxiao Jia, Yao Guo, Wenjun Liu, Lei Sun

**Affiliations:** 1 Institute of Human Immunology, Shenzhen Medical Academy of Research and Translation, Shenzhen, 518107 Guangdong, China; 2 Institute of Infectious Diseases, Shenzhen Bay Laboratory, Shenzhen, 518107 Guangdong, China; 3 Key Laboratory of Pathogenic Microbiology and Immunology, Institute of Microbiology, Chinese Academy of Sciences, Beijing, 100101 China; 4 Pediatric Hematology Laboratory, Division of Hematology/Oncology, Department of Pediatrics, The Seventh Affiliated Hospital of Sun Yat-Sen University, Shenzhen, 518107 Guangdong, China; 5 Beijing Institute of Heart, Lung and Blood Vessel Diseases, Beijing Anzhen Hospital, Capital Medical University, Beijing, 100029 China; 6 University of Chinese Academy of Sciences, Beijing, 100049 China; 7 Shandong Public Health Clinical Center, Jinan, 250100 Shandong China

**Keywords:** Cancer, Haematology

## Abstract

B-cell acute lymphoblastic leukemia (B-ALL) is an aggressive hematologic malignancy characterized by rapid proliferation of immature lymphoid cells. Despite treatment advancements, relapse remains a significant challenge. Understanding the molecular mechanisms that drive B-ALL progression is essential for developing more effective treatments. Here, we show that extracellular cyclophilin A (eCypA) is elevated in patients with B-ALL via both autocrine and paracrine mechanisms. Moreover, eCypA administration exacerbated B-ALL progression in a mouse model. Mechanistically, eCypA binds to the melanocortin 2 receptor and activates the downstream cAMP-PKA-CREB signaling pathway. This activation upregulates CD44, FN1, and MMP9 to promote trans-endothelial migration of B-ALL cells and increases the expression of anti-apoptotic proteins, thereby inhibiting cell apoptosis. Antibodies against CypA modestly reduced leukemia burden and delayed disease progression in both NALM6 xenograft and patient-derived xenograft mouse models. Furthermore, single-cell transcriptomics suggests that anti-CypA treatment reduces the proportion of cells with multipotent progenitor features and is accompanied by decreased CREB pathway activation. Collectively, these findings indicate that eCypA contributes to the progression of B-ALL and may represent a potential therapeutic target.

## Synopsis

Extracellular cyclophilin A (eCypA) was identified as a secreted driver of B-Cell acute lymphoblastic leukemia (B-ALL) progression by engaging MC2R-cAMP/PKA/CREB signaling to enhance trans-endothelial migration and survival and was partially counteracted by anti-CypA antibodies in vivo.

- Samples from patient with B-ALL were found to contain elevated eCypA, generated via both autocrine and paracrine routes.
- B-ALL progression was exacerbated in mice by exogenous eCypA, indicating an extracellular, disease-driving function.
- MC2R was identified as the eCypA receptor, and cAMP-PKA-CREB signaling was activated downstream.
- Trans-endothelial migration was enhanced by CREB-linked induction of CD44, FN1, and MMP9, while apoptosis resistance was increased via upregulated anti-apoptotic proteins.
- B-ALL burden was modestly reduced and progression was delayed by anti-CypA antibodies in NALM6 and PDX models, with scRNA-seq-defined shifts in leukemic subsets.

## Abstract (web summary)

Extracellular cyclophilin A (eCypA) was identified as a secreted driver of B-Cell acute lymphoblastic leukemia (B-ALL) progression by engaging MC2R-cAMP/PKA/CREB signaling to enhance trans-endothelial migration and survival and was partially counteracted by anti-CypA antibodies in vivo.

## Graphical abstract

**Figure:**
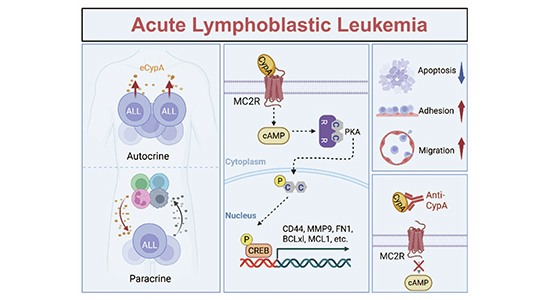

**Figure:**
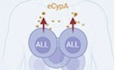

The paper explained **Problem**

B-cell acute lymphoblastic leukemia (B-ALL) is an aggressive hematologic malignancy in which relapse and treatment resistance remain major clinical challenges. Increasing evidence suggests that the tumor microenvironment and extracellular signaling molecules contribute to leukemic progression and survival. Cyclophilin A (CypA) is a secreted protein implicated in several malignancies. However, the role of extracellular CypA (eCypA) in B-ALL progression and the underlying signaling mechanisms remain poorly understood.

**Results**

We found that eCypA levels were elevated in patients with B-ALL and that exogenous eCypA accelerated leukemia progression in mouse models. Mechanistically, eCypA bound to the melanocortin 2 receptor (MC2R) and activated the cAMP/PKA/CREB signaling pathway, leading to increased leukemic cell migration and survival. Anti-CypA monoclonal antibody treatment reduced leukemia burden and delayed disease progression in both xenograft and patient-derived xenograft models. Single-cell transcriptomic analysis further indicated that anti-CypA treatment reduced leukemic cell populations with multipotent progenitor features and was associated with decreased CREB pathway activity.

**Impact**

This study identifies the eCypA/MC2R/CREB signaling axis as a regulator of B-ALL progression and supports eCypA as a potential therapeutic target. These findings provide mechanistic insight into how extracellular signaling contributes to leukemic cell-state regulation and may support the future development of antibody-based therapeutic strategies for B-ALL.

## Introduction

B-cell acute lymphoblastic leukemia (B-ALL) is a highly aggressive hematological malignancy characterized by the rapid proliferation of immature lymphoid progenitors (Cunningham et al, 2023; Malard and Mohty, 2020). Despite significant advances in treatment protocols, including chemotherapy, targeted therapy, and stem cell transplantation, relapse and treatment resistance remain substantial challenges (Terwilliger and Abdul-Hay, 2017). The persistence of minimal residual disease (MRD) and the survival of leukemic cells within protective niches, such as the bone marrow (BM) microenvironment, are key contributors to disease recurrence and poor prognosis (Gokbuget and Hoelzer, 2002; Hunger and Mullighan, 2015). Therefore, understanding the molecular mechanisms that drive B-ALL progression is needed for developing more effective treatments.

Cyclophilin A (CypA; encoded by peptidyl-prolyl isomerase A [*PPIA*]) is a highly conserved protein traditionally recognized for its role in protein folding, immune regulation, and cell signaling, particularly as a target for the immunosuppressive drug cyclosporine A (Bedir et al, 2024; Gothel and Marahiel, 1999; Liu et al, 1991). Extracellular cyclophilin A (eCypA) has gained attention for its role in promoting inflammation, where it acts as a cytokine-like mediator that modulates immune responses and recruits inflammatory cells (Bai et al, 2024; Dawar et al, 2017). Elevated levels of eCypA are associated with various inflammatory conditions, including cardiovascular diseases, rheumatoid arthritis, sepsis, breast cancer, and multiple myeloma, highlighting its significance in inflammatory pathways (Kim et al, 2005; Sigle et al, 2024; Wang et al, 2010). eCypA exerts its biological functions largely through interactions with cell-surface receptors, and accumulating evidence suggests that distinct receptors may mediate context-dependent eCypA signaling across different cell types and pathological conditions (Bai et al, 2024; Ji et al, 2021; Seizer et al, 2024; Yang et al, 2024; Zhao et al, 2026). However, the receptor-mediated signaling mechanisms of eCypA in hematological malignancies remain poorly understood.

Through proteomic analysis in B-ALL cell lines, we identified melanocortin 2 receptor (MC2R) as a candidate receptor for eCypA.The MC2R is a G protein-coupled receptor that plays a key role in the adrenal response by mediating the action of adrenocorticotropic hormone (ACTH) to produce glucocorticoids (Dallman et al, 2004; Gantz and Fong, 2003; Roy et al, 2007). Upon activation, MC2R triggers a signaling cascade that increases cyclic AMP (cAMP) levels, which in turn activates protein kinase A (PKA), leading to the phosphorylation and activation of cAMP response element-binding protein (CREB) (Gantz and Fong, 2003; Kilianova et al, 2006). CREB is a transcription factor that regulates gene expression in cell cycle progression, survival, and apoptosis (Persengiev and Green, 2003). Aberrant activation of this pathway has been associated with various cancers, contributing to malignancy and apoptosis resistance (Sakamoto and Frank, 2009). In the context of B-ALL, by inducing anti-apoptotic genes such as BCL2 family proteins, CREB helps protect B-ALL cells from chemotherapy-induced cell death (Zheng et al, 2021). However, the role of MC2R in hematological malignancies, including B-ALL, has not been previously reported. Despite the recognized importance of CREB signaling in B-ALL, whether upstream extracellular signals such as eCypA contribute to CREB activation through MC2R remains unknown.

In this study, we investigated the effects of eCypA on B-ALL progression in both in vitro and in vivo models, including a patient-derived xenograft (PDX) model. We further evaluated the therapeutic potential of a monoclonal antibody (mAb) targeting CypA. By blocking eCypA, we aimed to disrupt B-ALL progression, with the goal of improving treatment responses and reducing the risk of relapse. Our results suggest that eCypA contributes to B-ALL progression and indicate that targeting the eCypA–MC2R interaction may have therapeutic relevance.

## Results

### eCypA is upregulated in patients with B-ALL through both autocrine and paracrine signaling

**Figure 1:**
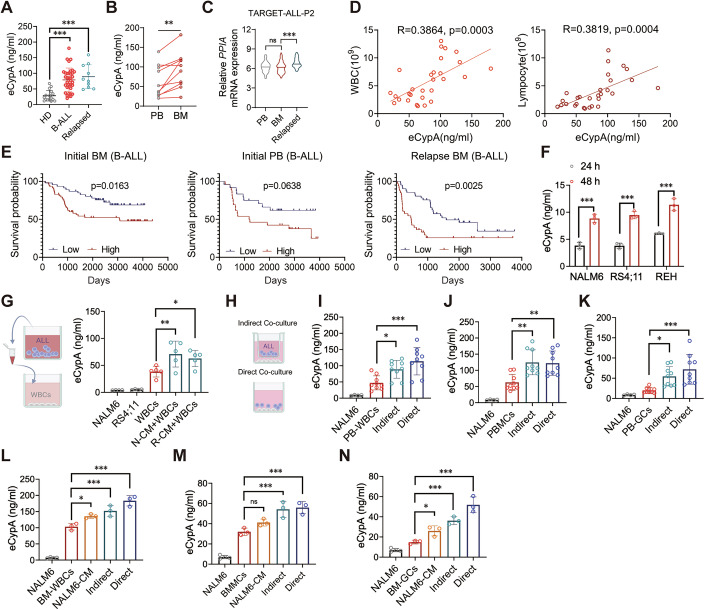
eCypA levels in patients with B-ALL. (**A**) eCypA levels were measured by enzyme-linked immunosorbent assay (ELISA) in peripheral blood (PB) plasma from healthy donors (HD, *n* = 17), patients with ALL (*n* = 26), and relapsed patients (*n* = 10). (**B**) Comparison of eCypA levels measured by ELISA in paired PB and bone marrow (BM) plasma samples from patients with ALL (*n* = 10). (**C**) *PPIA* mRNA levels in bone marrow (BM) and peripheral blood (PB) samples from the TARGET-ALL-P2 cohort obtained from the UCSC Xena platform. Only B-ALL samples were included. Samples were stratified into initial PB (*n* = 52), initial BM (*n* = 105), and relapse BM (*n* = 74). (**D**) Correlation analysis between eCypA levels and white blood cell (WBC, left) or lymphocyte counts (right) in patients with ALL (*n* = 29). (**E**) Kaplan–Meier survival curves of B-ALL patients stratified by *PPIA* mRNA levels in initial BM (*n* = 104), relapse BM (*n* = 74), and initial PB (*n* = 50). Patients were divided into high and low expression groups using the median expression level as the cutoff. Statistical significance was assessed using the log-rank test. For the relapse BM cohort, survival time was calculated from relapse to death or last follow-up. (**F**) ELISA analysis of eCypA levels in the culture supernatant of NALM6, RS4;11, and REH cells collected at the indicated time points (*n* = 3). (**G**) Schematic illustration of the conditioned medium (CM) assay. Supernatants from NALM6 (N-CM) or RS4;11 (R-CM) cells cultured for 24 h were used to stimulate WBCs from healthy donors (*n* = 5), and eCypA levels in the supernatant were measured by ELISA after 48 h. (**H**) Schematic illustration of indirect and direct co-culture systems involving NALM6 cells and PB- or BM-derived hematopoietic cells from healthy donors. (**I**–**K**) ELISA analysis of eCypA levels in the supernatant from indirect and direct co-cultures of NALM6 cells with peripheral blood WBCs (PB-WBCs) (**I**), peripheral blood mononuclear cells (PBMCs) (**J**), and peripheral blood granulocytes (PB-GCs) (**K**) from healthy donors (*n* = 9). (**L**–**N**) ELISA analysis of eCypA levels in the supernatant from NALM6-conditioned medium (NALM6-CM)-stimulated bone marrow WBCs (BM-WBCs, **L**), bone marrow mononuclear cells (BMMCs, **M**), and bone marrow granulocytes (BM-GCs, **N**) from healthy donors (*n* = 3), as well as from indirect and direct co-cultures of NALM6 cells with these BM-derived cells. Data are presented as mean ± SD; *n* represents individual patients or healthy donors (**A**–**E**). In (**F**), *n* represents independent wells. In (**G**, and **I**–**N**), n represents independent healthy donor samples. Statistical significance was determined using one-way ANOVA for (**A**), paired two-tailed Student’s *t*-test for (**B**), unpaired two-tailed Student’s *t*-test for (**C**), Spearman’s rank correlation analysis for (**D**), log-rank (Mantel–Cox) test for (**E**), two-way ANOVA for (**F**), and one-way ANOVA for (**G**) and (**I**–**N**). *p < 0.05, **p < 0.01, ***p < 0.001; ns not significant. Source data are available online for this figure.

To investigate the relationship between eCypA and B-ALL, we measured its expression in the peripheral blood (PB) of patients with B-ALL. The enzyme-linked immunosorbent assay (ELISA) analysis revealed that eCypA levels in PB were significantly higher in patients with B-ALL (*n* = 36) than in healthy donors (*n* = 17), and all relapsed patient samples (*n* = 10) also exhibited consistently higher levels than those in healthy controls (Fig. 1A and Table EV1). BM samples exhibited higher levels of eCypA expression than those in PB samples (Fig. 1B). These findings, based on protein-level measurements prompted us to further examine *PPIA* mRNA levels using data from the TARGET-ALL-P2 cohort (Data ref: Genomic Data Commons, TARGET-ALL-P2). Analysis of processed mRNA data showed that *PPIA* mRNA level was not significantly different between initial peripheral blood (PB) and bone marrow (BM) samples, but was elevated in relapse BM (Fig. 1C). In addition, circulating eCypA levels were positively correlated with white blood cell (WBC) and lymphocyte counts in PB (Fig. 1D). To further assess the clinical relevance of *PPIA* mRNA levels, we performed survival analyses after stratifying samples into initial PB, initial BM, and relapse BM groups. Patients were divided into high and low expression groups using the median expression value as the cutoff. Kaplan–Meier analysis showed that higher *PPIA* mRNA level was associated with poorer overall survival in the initial BM (*p* = 0.0163) and relapse BM (*p* = 0.0025) groups, whereas the association in the initial PB group did not reach statistical significance (*p* = 0.0638) (Fig. 1E). Multivariate Cox proportional hazards analysis, including *PPIA* mRNA level, age, sex, and fusion status as covariates, identified *PPIA* mRNA level as an independent risk factor across all three groups (Table EV2; Dataset EV1) (Data ref: Genomic Data Commons, TARGET-ALL-P2). Together, these results link *PPIA* mRNA expression and circulating eCypA protein to disease burden and clinical outcome in B-ALL.

**Figure EV1:**
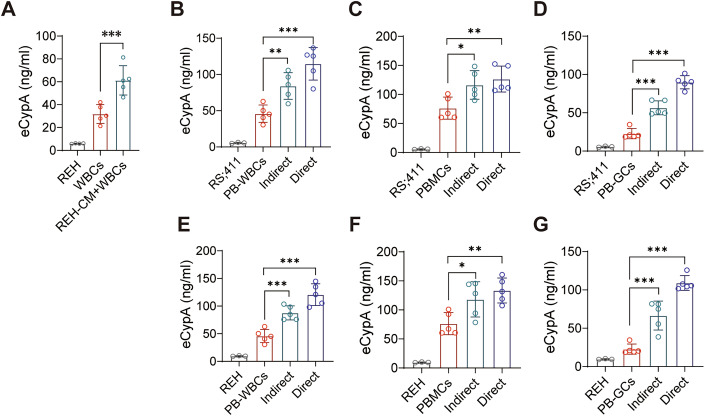
ALL cells stimulate eCypA secretion from peripheral blood-derived cells. (**A**) ELISA analysis of eCypA levels in the supernatant of WBCs cultured alone, REH cells (*n* = 4), or WBCs treated with REH-conditioned medium (REH-CM) (*n* = 5). (**B**–**D**) ELISA analysis of eCypA levels in the supernatant from indirect and direct co-cultures of RS4;11 cells with peripheral blood WBCs (PB-WBCs) (**B**), peripheral blood mononuclear cells (PBMCs) (**C**), and peripheral blood granulocytes (PB-GCs) (**D**) (*n* = 5). RS4;11-only control groups were analyzed using matched independent wells (*n* = 3). (**E**–**G**) ELISA analysis of eCypA levels in the supernatant from indirect and direct co-cultures of REH cells with PB-WBCs (**E**), PBMCs (**F**), and PB-GCs (**G**) (*n* = 5). REH-only control groups were analyzed using matched independent wells (*n* = 3). Data were presented as mean ± SD; in co-culture and conditioned medium groups, *n* represents independent donor samples. In cell line-only control groups, *n* represents independent wells. Statistical significance was determined using one-way ANOVA. *p < 0.05, **p < 0.01, ***p < 0.001; ns not significant. Source data are available online for this figure.

We further investigated the sources of the elevated eCypA levels. In vitro studies showed that human B-ALL cell lines (RS4;11, NALM6, and REH) secreted eCypA into culture supernatants (Fig. 1F). Moreover, a conditioned medium assay suggested that the supernatants from B-ALL cells significantly enhanced the secretion of eCypA by WBCs from healthy donors (*n* = 5) (Figs. 1G and EV1A). Subsequently, peripheral blood white blood cells (PB-WBCs), peripheral blood mononuclear cells (PBMCs), and peripheral blood granulocytes (PB-GCs) from healthy donors were co-cultured with B-ALL cells (NALM6, RS4;11, and REH) under direct or indirect conditions (Fig. 1H). The results showed that both direct and indirect co-culture with B-ALL cells significantly increased eCypA levels in the supernatants of PB-WBCs, PBMCs, and PB-GCs (Figs. 1I–K and EV1B–G). Similarly, eCypA levels were significantly elevated in the supernatant of NALM6 cell-conditioned medium (NALM6-CM)-stimulated bone marrow WBCs (BM-WBCs), bone marrow mononuclear cells (BMMCs), and bone marrow granulocyte cells (BM-GCs) from healthy donors, as well as from indirect and direct co-cultures of NALM6 cells with BM-WBCs, BMMCs, and BM-GCs (Fig. 1L–N). These findings suggest that leukemic cells may contribute to elevated extracellular CypA levels through both direct secretion and indirect modulation of surrounding hematopoietic cells, although the underlying mechanism remains unclear.

### eCypA accelerates B-ALL progression in NALM6-Luc mouse model

**Figure 2:**
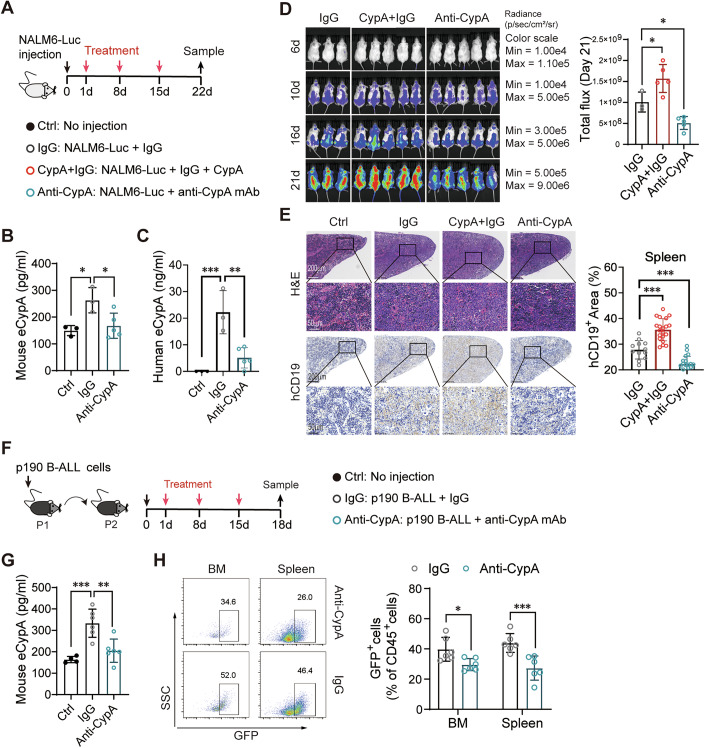
eCypA exacerbates ALL severity in a mouse model. (**A**) NALM6-Luc cells were injected via the tail vein into NCG mice, followed by weekly intraperitoneal administration of recombinant IgG (*n* = 3), CypA+IgG (*n* = 5), or anti-CypA monoclonal antibody (mAb) (*n* = 5) starting the next day. (**B**,** C**) ELISA analysis of mouse (**B**) and human (**C**) eCypA levels in peripheral blood (PB) plasma on day 18 after NALM6-Luc cell injection; samples from Ctrl mice (*n* = 3, no leukemia cell injection) were included as baseline controls. (**D**) Bioluminescence imaging of mice on day 21 following intraperitoneal injection of luciferin substrate (left), with quantitative analysis using an in vivo imaging system (right). Each data point represents the bioluminescence intensity from an individual mouse (IgG, *n* = 3; CypA + IgG, *n* = 5; Anti-CypA, *n* = 5). (**E**) Hematoxylin and eosin (H&E) staining and hCD19 immunohistochemistry (IHC) of spleen tissue sections collected on day 22 after leukemia cell injection (left), with quantification of hCD19⁺ areas using ImageJ (right). Scale bars, 200 and 50 μm as indicated. Each data point (*n*) represents one microscopic field, with four randomly selected fields analyzed per mouse (IgG, *n* = 12; CypA + IgG, *n* = 20; Anti-CypA, *n* = 20). (**F**) Schematic of the p190-BCR-ABL-driven B-ALL model in C57BL/6 mice. Primary leukemic spleen cells were harvested after 2 weeks of disease development and transplanted into secondary recipient mice via tail vein injection. Anti-CypA mAb treatment was initiated the next day and administered weekly for 3 weeks; mice were sacrificed on day 18 post-transplantation. (**G**) ELISA analysis of mouse eCypA levels in PB plasma from Ctrl (*n *= 4), IgG (*n* = 6), and Anti-CypA (*n* = 6) groups. (**H**) Flow cytometric analysis of GFP⁺ leukemic cell infiltration in bone marrow (BM) and spleen from IgG and Anti-CypA groups, including representative flow cytometry plots and quantification (*n* = 6). Data were presented as mean ± SD; *n* represents individual mice (**B**–**D**,** G**, **H**). In (**E**), *n* represents independently analyzed microscopic fields. Statistical significance was determined using one-way ANOVA for (**B**,** C**, **G**), unpaired two-tailed Student’s *t*-test for (**D**), and two-way ANOVA for (**E**, **H**). *p < 0.05, **p < 0.01, ***p < 0.001; ns not significant. Source data are available online for this figure.

**Figure EV2:**
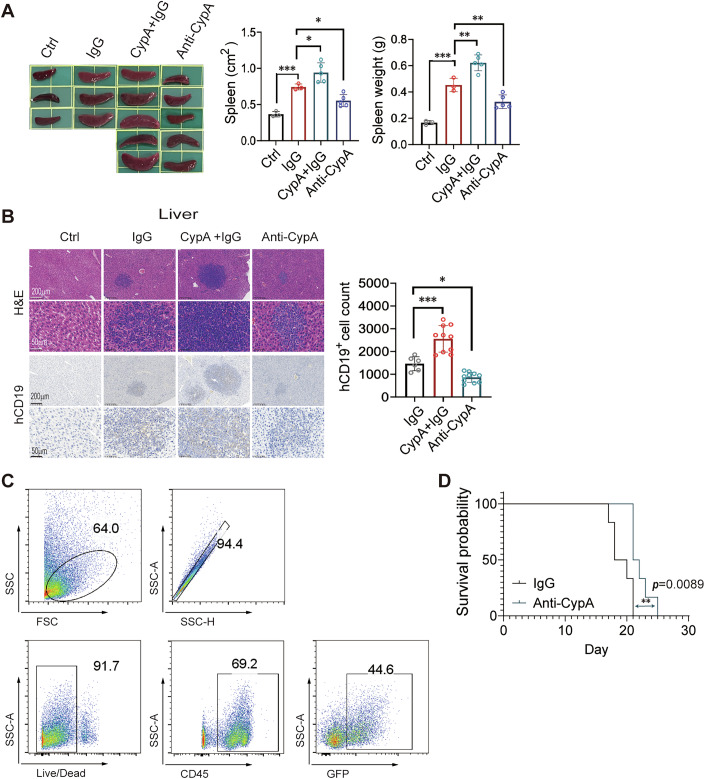
eCypA exacerbates splenomegaly and promotes leukemic infiltration in vivo. (**A**) Representative images of spleens from mice on day 22 post-ALL injection (left), with quantification of spleen area using ImageJ (middle) and spleen weight (right). Groups included Ctrl (*n* = 3), IgG (*n* = 3), CypA (*n* = 5), and Anti-CypA (*n* = 5). (**B**) H&E staining and hCD19 immunohistochemistry of mouse liver sections (left), with quantitative analysis of hCD19⁺ areas using ImageJ (right). Scale bars, 200 and 50 μm as indicated. Each data point represents one microscopic field, with two randomly selected fields analyzed per mouse (IgG, *n* = 6; CypA+IgG, *n* = 10; Anti-CypA, *n* = 10). (**C**) Flow cytometry gating strategy for the p190-BCR-ABL mouse model. Cells were sequentially gated on FSC/SSC to exclude debris, followed by singlet discrimination (SSC-H vs SSC-A), live cells, mCD45⁺ leukocytes, and finally GFP⁺ leukemic cells. (**D**) Kaplan–Meier survival analysis of p190-BCR-ABL mice treated with IgG (*n* = 6) or Anti-CypA mAb (*n* = 6). Data were presented as mean ± SD; in (**A**, **D**, *n* represents individual mice. In (**B**), *n* represents independently analyzed microscopic fields. Statistical significance was determined using unpaired two-tailed Student’s *t*-test for (**A**), one-way ANOVA for (**B**), and log-rank (Mantel–Cox) test for (**D**). *p < 0.05, **p < 0.01, ***p < 0.001; ns not significant. Source data are available online for this figure.

To investigate the role of eCypA in B-ALL progression, the NALM6-Luc mouse model was constructed (Fig. 2A). Eighteen days after injection with NALM6-Luc cells, the levels of both mouse (Fig. 2B) and human (Fig. 2C) eCypA were elevated in the PB plasma of mice. Notably, no cross-reactivity was observed between the mouse and human ELISA kits (Appendix Fig. S1). Treatment with anti-CypA mAb effectively reduced this elevation. These findings suggest that eCypA is secreted by human B-ALL cells and adjacent mouse cells, consistent with the results discussed in Fig. 1G–K. Bioluminescence imaging showed that eCypA enhanced leukemia burden in mice, while anti-CypA mAb reduced bioluminescence signals (Fig. 2D). Additionally, eCypA increased spleen size in ALL mice, whereas anti-CypA mAb treatment partially reduced splenomegaly (Fig. EV2A). Moreover, mice treated with eCypA exhibited increased infiltration of hCD19-positive cells in the spleen, which was attenuated by anti-CypA mAb treatment (Fig. 2E). Similar results were observed in the mouse liver samples (Fig. EV2B).

To further validate these findings in an immunocompetent model, we established a P190-BCR-ABL1-driven B-ALL mouse model. Bone marrow cells were transduced with P190-BCR-ABL1 and transplanted into primary recipient mice, followed by secondary transplantation two weeks later. Secondary recipient mice were then treated with either IgG or anti-CypA mAb (Fig. 2F). ELISA analysis of peripheral blood plasma showed that mouse CypA levels were significantly elevated in the leukemia-bearing IgG-treated group compared with control mice without leukemia, whereas anti-CypA mAb treatment effectively neutralized this increase (Fig. 2G). Mice received antibody treatment once weekly, starting 1 day after leukemia cell transplantation. On day 18, mice were sacrificed and flow cytometry analysis was performed, with the gating strategy for CD45⁺GFP⁺ leukemic cells in spleen and bone marrow shown in Fig. EV2C. The results demonstrated that anti-CypA mAb treatment reduced leukemic infiltration in the spleen and partially decreased leukemic cell engraftment in the bone marrow (Fig. 2H). Survival analysis further showed that anti-CypA mAb treatment modestly prolonged the survival of leukemia-bearing mice (Fig. EV2D). These results suggest that elevated eCypA levels are associated with B-ALL progression and that blocking eCypA may attenuate disease progression.

### eCypA binding to MC2R triggers cAMP–PKA–CREB signaling

**Figure 3:**
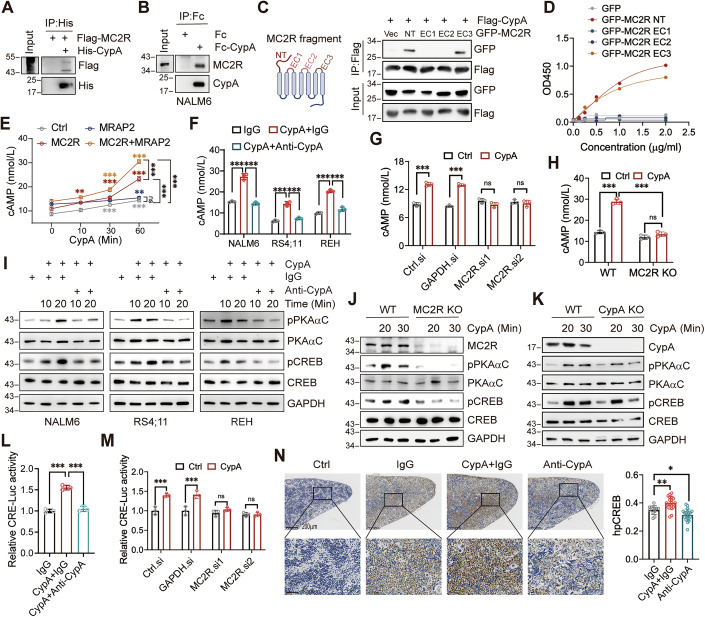
eCypA binding to MC2R activates cAMP-PKA-CREB signaling. (**A**) Flag-MC2R was transfected into 293T cells for 36 h, followed by incubation of cell lysates with or without His-tagged recombinant CypA protein. His pull-down assays were analyzed by western blotting. (**B**) NALM6 cell lysates were incubated with Fc-CypA, followed by Protein A pull-down and detection of MC2R by western blotting. (**C**) Schematic representation of MC2R. NT, N-terminal; EC1–EC3, extracellular loops. GFP-tagged MC2R fragments or an empty vector were co-transfected with Flag-CypA into 293 T cells, followed by IP and western blotting. (**D**) ELISA-based binding assay using coated recombinant CypA and GFP-tagged MC2R fragments. (**E**) 293T cells were transfected with Flag-Vec, Flag-MC2R, Flag-MRAP2, or co-transfected with Flag-MC2R and Flag-MRAP2 for 36 h, followed by treatment with CypA for the indicated time points. Intracellular cAMP levels were then measured. (**F**) NALM6, RS4;11, and REH cells were treated with IgG, IgG + CypA, or Anti-CypA + CypA for 10 min, followed by cAMP measurement. (**G**) 293T cells transfected with Ctrl.si, GAPDH-siRNA, or MC2R siRNAs were treated with CypA for 1 h, followed by cAMP measurement. (**H**) NALM6 WT and MC2R KO cells were treated with or without CypA for 10 min, followed by cAMP measurement. (**I**) NALM6, RS4;11, and REH cells were treated as indicated, and phosphorylation of PKAαC and CREB was analyzed by western blotting. (**J**) NALM6 WT and MC2R KO cells were treated with CypA for the indicated times, followed by western blotting. (**K**) Western blot analysis of WT and CypA KO cells. (**L**) CRE-luciferase activity in 293T cells transfected with Flag-Vec or Flag-MC2R and treated as indicated. (**M**) CRE-luciferase activity in 293T cells transfected with siRNAs and treated with CypA. (**N**) pCREB immunohistochemistry in spleen sections with quantification, each data point represents one microscopic field, with four randomly selected fields analyzed per mouse (IgG, *n* = 12; CypA + IgG, *n* = 20; Anti-CypA, *n* = 20). Data were presented as mean ± SD; in (**E**–**H**,** L**, **M**), *n* = 3 represents three independent wells. Western blot and immunoprecipitation results in (**A**–**C**, **I**–**K**) are representative of at least three independent experiments with similar results. Statistical significance was determined by one-way ANOVA for (**L**, **N**) and two-way ANOVA for (**F**–**H**, **M**). *p < 0.05, **p < 0.01, ***p < 0.001; ns not significant. Source data are available online for this figure.

**Figure EV3:**
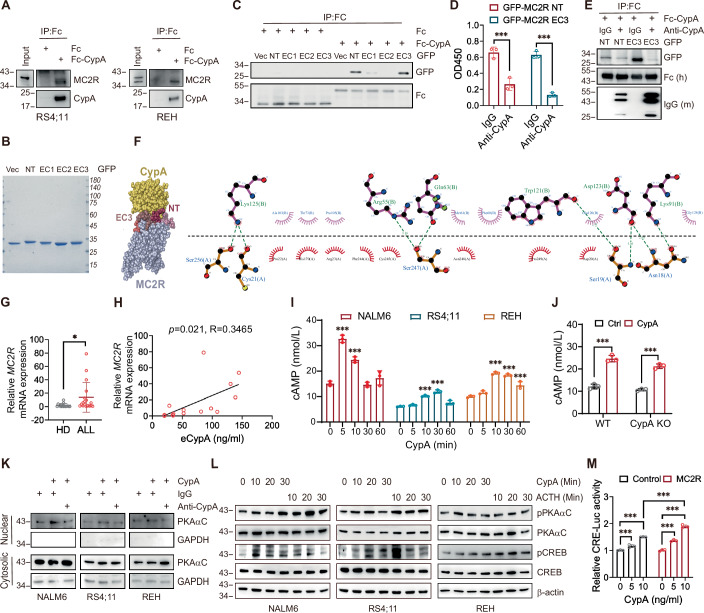
eCypA activates the cAMP–PKA–CREB signaling pathway. (**A**) RS4;11 and REH cell lysates were incubated with Fc-CypA, followed by Protein A pull-down and detection of MC2R by western blotting. (**B**) Coomassie brilliant blue staining of eukaryotically purified GFP-tagged proteins. (**C**) Fc or Fc-CypA recombinant protein was incubated with GFP-tagged fragments, followed by Protein A pull-down and western blot detection of GFP and Fc. (**D**) An ELISA-based binding assay in which recombinant CypA was coated and increasing concentrations of GFP-tagged NT and EC3 fragments, with or without Anti-CypA mAb, were added, followed by OD measurement. (**E**) Fc or Fc-CypA recombinant protein was incubated with GFP-tagged fragments in the presence of Anti-CypA mAb or IgG, followed by Protein A pull-down and western blot analysis. (**F**) Molecular docking simulation showing the predicted binding model between CypA and MC2R NT and EC3 domains. (**G**) qPCR analysis of *MC2R* mRNA levels in PBMCs from healthy donors (HD, *n* = 15) and patients with ALL (*n* = 18). (**H**) Correlation analysis between *MC2R* mRNA levels and eCypA levels in patients with ALL (*n* = 15). (**I**) cAMP levels in NALM6, RS4;11, and REH cells treated with CypA for the indicated time points. (**J**) cAMP levels in NALM6 WT and CypA KO cells treated with or without CypA for 10 min. (**K**) NALM6, RS4;11, and REH cells were treated with IgG, CypA + IgG, or CypA+Anti-CypA for 10 min. Nuclear and cytoplasmic fractions were isolated, and PKAα-C levels were detected by western blotting. (**L**) NALM6, RS4;11, and REH cells were treated with 5 ng/mL CypA or 0.8 ng/mL ACTH for the indicated time points, followed by western blotting to assess activation of the PKA–CREB pathway. (**M**) Luciferase reporter assay of CRE activity in 293 T cells transfected with Flag-Vec or Flag-MC2R and treated with increasing concentrations of CypA. Data are presented as mean ± SD; in (**D**,** I**,** J**, **M**), *n* = 3 represents independent wells. In (**G**, **H**), *n* represents individual donors or patient samples. Western blot and pull-down results in (**A**–**C**,** E**,** K**,** L**) are representative of at least three independent experiments with similar results. Statistical significance was determined using an unpaired two-tailed Student’s *t*-test for (**G**), Pearson correlation analysis for (**H**), and two-way ANOVA for (**D**, **I**, **J**, **M**). *p < 0.05, **p < 0.01, ***p < 0.001; ns not significant. Source data are available online for this figure.

To identify proteins interacting with eCypA in human B-ALL cell lines, we incubated Fc-CypA with NALM6 and RS4;11 cell lines and performed immunoprecipitation (IP) followed by mass spectrometry. Among the candidates identified, MC2R was detected in both cell lines and exhibited higher peptide counts, PSMs, and protein scores in the Fc-CypA group compared with the Fc control in RS4;11 cells, supporting its enrichment in the Fc-CypA pull-down (Dataset EV2). Consistently, *MC2R* transcripts were detected in NALM6 cells by qPCR analysis (Appendix Fig. S2). The interaction between CypA and MC2R was supported by pull-down assays. In an overexpression system, Flag-MC2R was detected in His-CypA pull-down samples (Fig. 3A). In addition, Fc-CypA pull-down assays detected MC2R in three B-ALL cell lines (NALM6, RS4;11, and REH) (Figs. 3B and EV3A). Furthermore, IP results showed that CypA interacted with the N-terminus (NT) and extracellular loop 3 (EC3) of MC2R (Fig. 3C). We then individually purified the four extracellular segments of MC2R (Fig. EV3B). The binding of CypA to the NT and EC3 of MC2R was further validated using IP assays (Fig. EV3C) and ELISA (Fig. 3D). Anti-CypA mAb effectively blocked the binding of CypA to the two regions of MC2R (Fig. EV3D,E). Molecular docking simulations were performed to visualize the binding model based on the experimentally identified interaction regions (Fig. EV3F). These results suggest that eCypA interacts with extracellular domains of MC2R (NT and EC3), potentially enabling receptor engagement and downstream signaling activation. Additionally, we observed that MC2R expression was significantly higher in the PBMCs of patients with ALL than in healthy individuals (Fig. EV3G), and showed a moderate positive correlation with eCypA levels in the PB of patients with ALL (Fig. EV3H), likely reflecting a bulk-level association given the heterogeneous nature of PBMCs.

To determine whether eCypA regulates MC2R signaling, we investigated the effects of eCypA on cAMP production and PKA and CREB activation. eCypA significantly increased cAMP production in NALM6, RS4;11 and REH cells (Fig. EV3I). Overexpression of MC2R with eCypA administration further increased cAMP levels, while co-expression of MRAP2 further enhanced cAMP production (Fig. 3E). These findings are consistent with a ligand-dependent mode of MC2R activation distinct from the classical ACTH–MC2R paradigm(Chan et al, 2009). Moreover, eCypA-induced cAMP production was effectively blocked by anti-CypA mAb in NALM6, RS4;11 and REH cells (Fig. 3F), by knockdown of MC2R in 293T cells (Fig. 3G), and by MC2R knockout in NALM6 cells (Fig. 3H). In addition, exogenous eCypA stimulation still increased cAMP production in CypA knockout (KO) cells (Fig. EV3J). Additionally, eCypA promoted the nuclear translocation of the protein kinase A catalytic subunit (PKAαC) (Fig. EV3K) and increased the phosphorylation of PKAαC and cAMP response element-binding protein (CREB), which was abolished by anti-CypA mAb (Fig. 3I). Accordingly, MC2R knockout suppressed the eCypA-triggered phosphorylation of PKAαC and activation of CREB (Fig. 3J). Notably, eCypA still activated this signaling pathway in CypA KO cells (Fig. 3K). In addition, both eCypA and ACTH, known ligands of MC2R (Metz et al, 2005), effectively activated the PKA-CREB pathway (Fig. EV3L). eCypA enhanced the CRE reporter gene activity, and this effect was further increased by MC2R overexpression (Fig. EV3M), but was suppressed by the anti-CypA mAb (Fig. 3L) and MC2R knockdown (Fig. 3M). Furthermore, eCypA increased pCREB expression in the spleens from NALM6-Luc-injected mice, which was inhibited by anti-CypA mAb (Fig. 3N). These findings support that the binding of eCypA to MC2R activates the cAMP–PKA–CREB signaling pathway.

### eCypA activates the MC2R-cAMP-PKA-CREB signaling to promote transendothelial migration

**Figure EV4:**
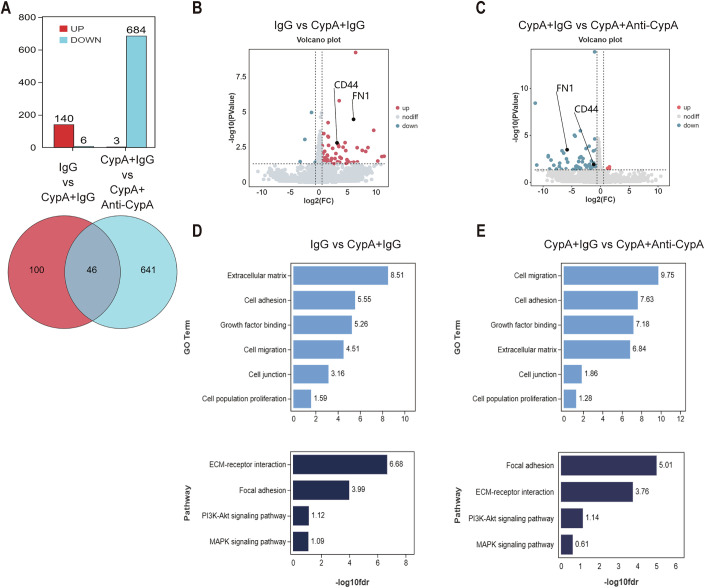
Transcriptomic analysis of eCypA-regulated genes in NALM6 cells. NALM6 cells were serum-starved for 6 h and then treated with IgG, CypA + IgG, or CypA + Anti-CypA with three biologically independent samples analyzed per group (*n* = 3 per group). Raw transcriptome reads were deposited in the China National Center for Bioinformation (BioProject accession: PRJCA035020; raw data accession: HRA010096). (**A**) Bar plot showing the numbers of upregulated and downregulated genes; 46 genes were upregulated by CypA and downregulated by Anti-CypA. (**B**, **C**) Volcano plots of differentially expressed genes generated from RNA-seq analysis. Differentially expressed genes were identified using a threshold of *P* < 0.05. (**D**) Gene Ontology (GO) enrichment analysis of differentially expressed genes between IgG and CypA + IgG-treated NALM6 cells. (**E**) KEGG pathway enrichment analysis of differentially expressed genes between CypA + IgG and CypA + anti-CypA-treated NALM6 cells. Source data are available online for this figure.

**Figure 4:**
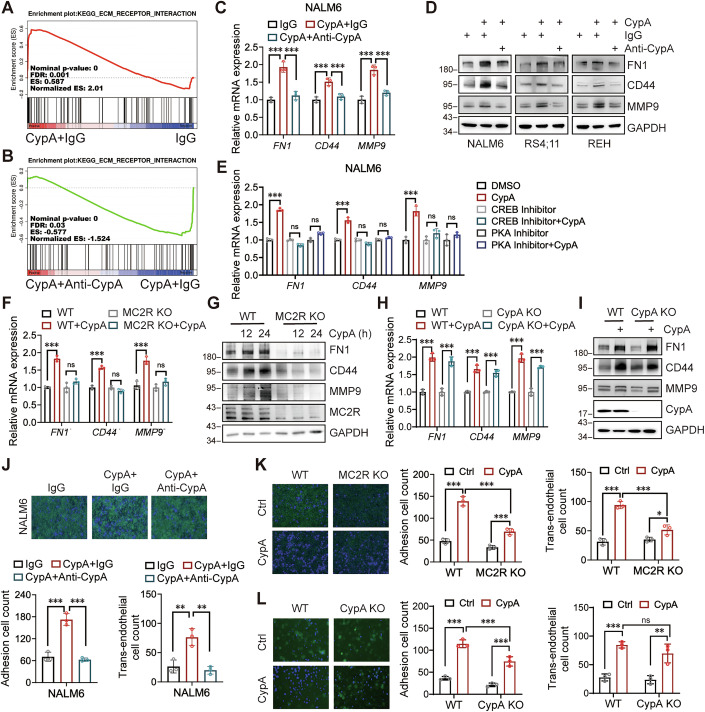
eCypA promotes transendothelial migration of ALL cells by activating the MC2R-cAMP-PKA-CREB signaling pathway. (**A**,** B**) Gene set enrichment analysis (GSEA) showing significant enrichment of the ECM–receptor interaction gene set in recombinant CypA plus IgG-treated versus IgG-treated NALM6 cells (**A**), and in recombinant CypA plus IgG-treated versus recombinant CypA plus anti-CypA monoclonal antibody-treated NALM6 cells (**B**). (**C**) qPCR analysis of *FN1*, *CD44*, and *MMP9* mRNA levels in NALM6 cells treated with IgG, CypA + IgG, or CypA + anti-CypA for 30 min. (**D**) Western blot analysis of FN1, CD44, and MMP9 expression in NALM6, RS4;11, and REH cells treated with IgG, CypA + IgG, or CypA + anti-CypA for 24 h. (**E**) qPCR analysis of *FN1*, *CD44*, and *MMP9* mRNA levels in NALM6 cells pretreated with a CREB inhibitor or a PKA inhibitor for 4 h, followed by CypA treatment for 30 min. (**F**) qPCR analysis of *FN1*, *CD44*, and *MMP9* mRNA levels in NALM6 wild-type (WT) and MC2R knockout (KO) cells treated with CypA for 30 min. (**G**) Western blot analysis of FN1, CD44, and MMP9 expression in NALM6 WT and MC2R KO cells treated with CypA for 12 or 24 h. (**H**) qPCR analysis of *FN1*, *CD44*, and *MMP9* mRNA levels in NALM6 WT and CypA KO cells treated with CypA for 30 min. (**I**) Western blot analysis of FN1, CD44, and MMP9 expression in NALM6 WT and CypA KO cells treated with CypA for 24 h. (**J**) In the transendothelial migration assay, HUVECs were seeded in the upper chamber for 48 h to form an endothelial layer, and Hoechst-stained NALM6 cells were then added and treated with IgG, CypA + IgG, or CypA + anti-CypA. The number of adherent ALL cells was quantified after 4 h, and the number of cells migrated across the endothelial layer was measured after 24 h. (**K**) Transendothelial migration assay showing the number of adherent and migrated NALM6 WT and MC2R KO cells after CypA treatment (4 h for adhesion and 24 h for migration). (**L**) Transendothelial migration assay showing the number of adherent and migrated NALM6 WT and CypA KO cells after CypA treatment (4 h for adhesion and 24 h for migration). Data were presented as mean ± SD; in (**C**,** E**,** F**,** H**, **J**–**L**), *n* = 3 represents three independent wells. Western blot results in (**D**,** G**, **I**) are representative of at least three independent experiments with similar results. Statistical significance was determined using one-way ANOVA for (**J**) and two-way ANOVA for (**C**,** E**,** F**,** H**,** K**, **L**). *p < 0.05, **p < 0.01, ***p < 0.001; ns not significant. Source data are available online for this figure.

To explore how eCypA influences ALL progression, we conducted transcriptomic analysis of NALM6 cells treated with IgG control, recombinant CypA plus IgG, or recombinant CypA plus anti-CypA monoclonal antibody. Differential expression analysis identified forty-six genes that were upregulated in recombinant CypA plus IgG-treated cells compared with IgG-treated control cells and downregulated in recombinant CypA plus anti-CypA monoclonal antibody-treated cells compared with recombinant CypA plus IgG-treated cells, including FN1 and CD44 (Fig. EV4A–C). Gene Ontology enrichment analysis showed that these genes were associated with cell adhesion- and migration-related biological processes. Kyoto Encyclopedia of Genes and Genomes pathway analysis highlighted significant enrichment of extracellular matrix (ECM) and focal adhesion pathways (Fig. EV4D,E). Furthermore, gene set enrichment analysis demonstrated enrichment of gene sets related to ECM–receptor interaction in recombinant CypA plus IgG-treated cells compared with IgG-treated control cells, whereas this enrichment was attenuated following anti-CypA monoclonal antibody treatment (Fig. 4A,B).

**Figure EV5:**
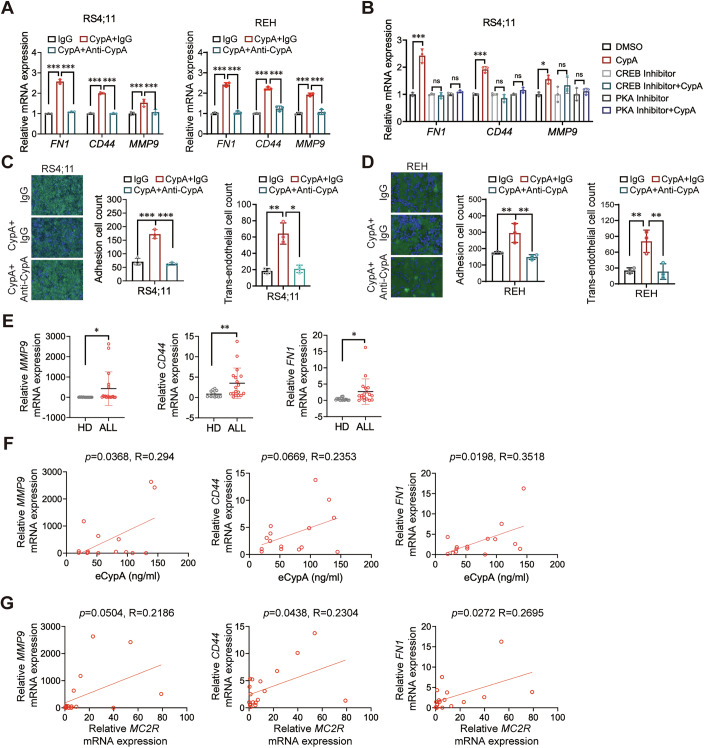
Effects of eCypA on transendothelial migration of ALL cells. (**A**) qPCR analysis of *FN1*, *CD44*, and *MMP9* mRNA levels in RS4;11 and REH cells treated with IgG, CypA + IgG, or CypA + anti-CypA for 30 min. (**B**) qPCR analysis of *FN1*, *CD44*, and *MMP9* mRNA levels in RS4;11 cells pretreated with a CREB inhibitor or a PKA inhibitor for 4 h, followed by CypA treatment for 30 min. (**C**, **D**) Transendothelial migration assay: HUVECs were seeded in the upper chamber for 48 h to form an endothelial layer. Hoechst-stained RS4;11 (**C**) and REH (**D**) cells were added and treated with IgG, CypA + IgG, or CypA + anti-CypA. The number of adherent cells was quantified after 4 h, and migrated cells were quantified after 24 h. (**E**) qPCR analysis of *FN1*, *CD44*, and *MMP9* mRNA levels in PBMCs from healthy donors (HD, *n* = 15) and patients with ALL (*n* = 18). (**F**) Pearson correlation analysis between *FN1*, *CD44*, and *MMP9* mRNA levels in PBMCs and eCypA levels in PB plasma (*n* = 15). (**G**) Pearson correlation analysis between *FN1*, *CD44*, and *MMP9* mRNA levels and *MC2R* mRNA levels in PBMCs (*n* = 18). Data were presented as mean ± SD; in (**A**–**D**), *n* = 3 represents independent wells. In (**E**–**G**), *n* represents individual donors or patient samples. Statistical significance was determined using two-way ANOVA for (**A**, **B**), one-way ANOVA for (**C**,** D**), unpaired two-tailed Student’s *t*-test for (**E**), and Pearson correlation analysis for (**F**, **G**). *p < 0.05, **p < 0.01, ***p < 0.001; ns not significant. Source data are available online for this figure.

We further examined the expression levels of genes associated with cell adhesion and migration. The quantitative PCR (qPCR) and western blotting results showed that CD44, FN1, and MMP9 were upregulated by CypA treatment, and their expression was reversed by anti-CypA mAb in NALM6, RS4;11 and REH cells (Figs. 4C,D and EV5A). CREB is a transcription factor that regulates diverse cellular responses, including proliferation, survival, and differentiation (Shaywitz and Greenberg, 1999). eCypA no longer enhanced *CD44, FN1*, or *MMP9* mRNA levels following treatment with PKA and CREB inhibitors in ALL cell lines (Figs. 4E and EV5B), suggesting that eCypA-induced upregulation of these genes depends on both PKA and CREB activity. Similar results were observed in MC2R knockout cells (Fig. 4F,G). Notably, CypA KO cells exhibited changes comparable to those observed in wild-type cells upon eCypA stimulation (Fig. 4H,I). These results indicated that eCypA enhanced gene expression in cell adhesion and migration through the MC2R-mediated cAMP-PKA-CREB signaling pathway.

The effect of eCypA on transendothelial migration was also investigated. eCypA treatment significantly enhanced the adhesion of ALL cells to human umbilical vein endothelial cells (HUVECs) in multiple B-ALL cell lines and promoted transendothelial migration, both of which were effectively blocked by anti-CypA mAb (Figs. 4J and EV5C,D). MC2R knockout similarly suppressed these effects (Fig. 4K). In contrast, CypA KO cells did not prevent eCypA-induced transendothelial migration, although a modest reduction in cell adhesion was observed (Fig. 4L). In addition, qPCR analysis of PBMCs from patients with ALL and healthy donors revealed that the mRNA levels of *FN1*, *CD44*, and *MMP9* were significantly upregulated in patients, and their levels showed a moderate positive correlation with eCypA protein levels and *MC2R* mRNA levels (Fig. EV5E–G). Given the heterogeneous nature of PBMCs, this likely reflects a bulk-level association between eCypA/MC2R expression and adhesion- and migration-related gene expression in patients with ALL. Together, these observations suggest that eCypA/MC2R signaling may function as an upstream regulator of these established pathways.

### eCypA activates the MC2R–cAMP–PKA–CREB signaling pathway to modestly promote cell survival and attenuate vincristine-induced apoptosis

**Figure 5:**
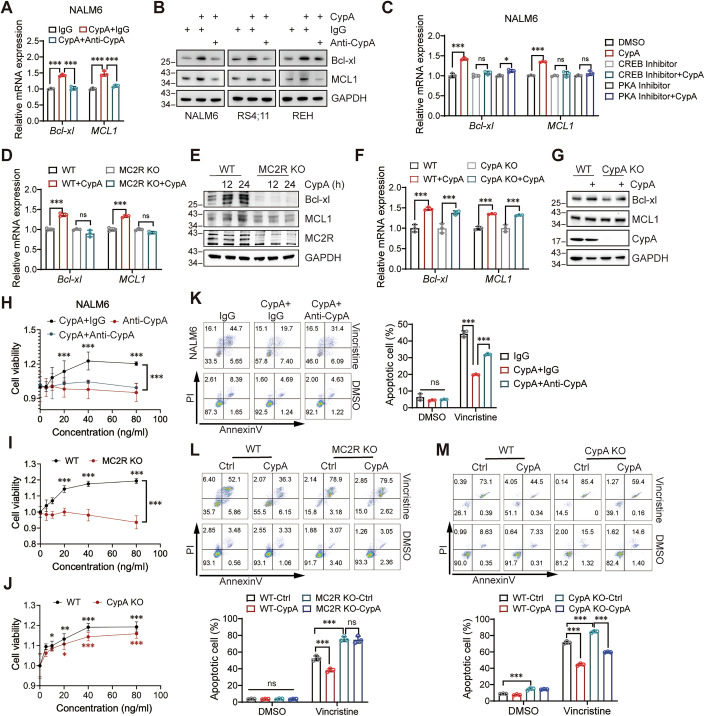
eCypA prevents apoptosis of ALL cells by activating the MC2R–cAMP–PKA–CREB signaling pathway. (**A**) qPCR analysis of Bcl-xL and MCL1 expression in NALM6 cells treated with IgG, CypA + IgG, or CypA + anti-CypA for 30 min. (**B**) Western blot analysis of Bcl-xL and MCL1 expression in NALM6, RS4;11, and REH cells treated with IgG, CypA + IgG, or CypA + anti-CypA for 24 h. (**C**) qPCR analysis of Bcl-xL and MCL1 expression in NALM6 cells pretreated with a CREB inhibitor or a PKA inhibitor for 4 h, followed by treatment with or without CypA for 30 min. (**D**) qPCR analysis of Bcl-xL and MCL1 expression in NALM6 wild-type (WT) and MC2R knockout (KO) cells treated with or without CypA for 30 min. (**E**) Western blot analysis of Bcl-xL and MCL1 expression in NALM6 WT and MC2R KO cells treated with CypA for 12 or 24 h. (**F**) qPCR analysis of Bcl-xL and MCL1 expression in NALM6 WT and CypA KO cells treated with or without CypA for 30 min. (**G**) Western blot analysis of Bcl-xL and MCL1 expression in NALM6 WT and CypA KO cells treated with CypA for 24 h. (**H**) NALM6 cells were treated with indicated concentrations of CypA + IgG, anti-CypA, or CypA + anti-CypA for 48 h, and cell viability was measured using the CCK-8 assay. Data were normalized to the untreated control (0 ng/mL). (**I**) NALM6 WT and MC2R KO cells were treated with indicated concentrations of CypA for 48 h, and cell viability was measured using the CCK-8 assay. Data were normalized to the untreated control (0 ng/mL). (**J**) NALM6 WT and CypA KO cells were treated with indicated concentrations of CypA for 48 h, and cell viability was measured using the CCK-8 assay. Data were normalized to the untreated control (0 ng/mL). (**K**) NALM6 cells were treated with IgG, CypA + IgG or CypA + anti-CypA, with or without vincristine, for 36 h, and apoptosis was assessed by Annexin V/PI staining. (**L**) NALM6 WT and MC2R KO cells were treated with vincristine, with or without CypA, for 36 h, and apoptosis was assessed by Annexin V/PI staining. (**M**) NALM6 WT and CypA KO cells were treated with vincristine, with or without CypA, for 36 h, and apoptosis was assessed by Annexin V/PI staining. Data were presented as mean ± SD; in (**A**,** C**,** D**,** F**, **H**–**M**), *n* = 3 represents three independent wells. Western blot results in (**B**, **E**, **G**) are representative of at least three independent experiments with similar results. Statistical significance was determined using two-way ANOVA. *p < 0.05, **p < 0.01, ***p < 0.001; ns not significant. Source data are available online for this figure.

**Figure EV6:**
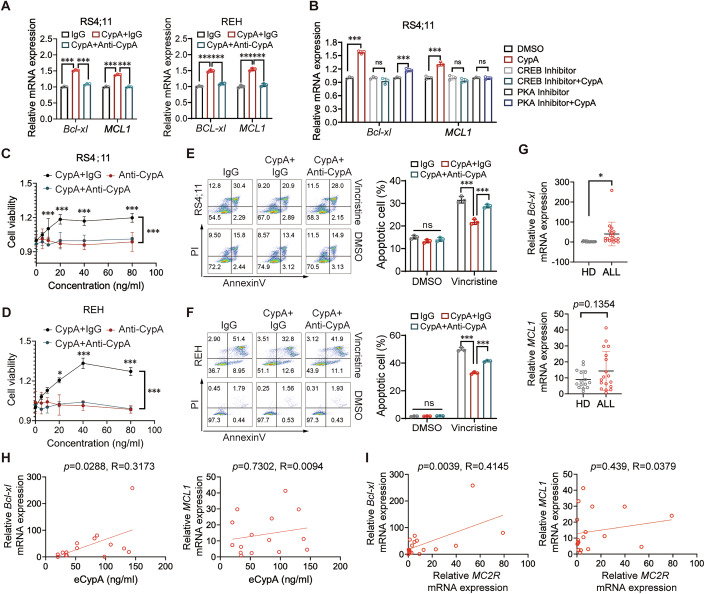
Anti-apoptotic effects of CypA on ALL cells. (**A**) qPCR analysis of Bcl-xL (BCL2L1) and MCL1 expression in RS4;11 and REH cells treated with IgG, CypA + IgG, or CypA + anti-CypA for 30 min. (**B**) qPCR analysis of Bcl-xL (BCL2L1) and MCL1 expression in RS4;11 cells pretreated with a CREB inhibitor or a PKA inhibitor for 4 h, followed by CypA treatment for 30 min. (**C**, **D**) Cell viability measured by CCK-8 assay in RS4;11 (**C**) and REH (**D**) cells treated with increasing concentrations of CypA + IgG, anti-CypA, or CypA + anti-CypA for 48 h (*n* = 3). (**E**, **F**) RS4;11 (**E**) and REH (**F**) cells were treated with IgG, CypA + IgG, or CypA + anti-CypA, with or without vincristine, for 36 h, and apoptosis was assessed by Annexin V/PI staining. (**G**) qPCR analysis of *Bcl-xL* and *MCL1* mRNA levels in PBMCs from healthy donors (HD, *n* = 15) and patients with ALL (*n* = 18). (**H**) Pearson correlation analysis between *Bcl-xL* and *MCL1* mRNA levels in PBMCs and eCypA protein levels in PB plasma (*n* = 15). (**I**) Pearson correlation analysis between *Bcl-xL* and *MCL1* mRNA levels and *MC2R* mRNA levels in PBMCs (*n* = 18). Data were presented as mean ± SD; in (**A**–**F**), *n* = 3 represents independent wells. In (**G**–**I**), *n* represents individual donors or patient samples. Statistical significance was determined using two-way ANOVA for (**A**–**F**), unpaired two-tailed Student’s *t*-test for (**G**), and Pearson correlation analysis for (**H**,** I**). *p < 0.05, **p < 0.01, ***p < 0.001; ns not significant. Source data are available online for this figure.

CREB induces survival signals that prevent apoptosis by upregulating anti-apoptotic proteins such as Bcl-xL and MCL-1 (Chiou et al, 2023; Wang et al, 2019), both of which play critical roles in ALL(Salomons et al, 1999). Therefore, we next examined whether eCypA regulates Bcl-xl and MCL1 expression through the MC2R-cAMP-PKA-CREB signaling. The results showed that eCypA upregulated *Bcl-xL* and *MCL1* expression in NALM6, RS4;11 and REH cells (Figs. 5A,B and EV6A), which was suppressed by anti-CypA mAb treatment. Moreover, CREB and PKA inhibitors suppressed the eCypA-induced upregulation of *Bcl-xL* and *MCL1* in all cell lines (Figs. 5C and EV6B). Furthermore, in MC2R knockout cells, eCypA failed to increase the mRNA (Fig. 5D) or protein (Fig. 5E) levels of Bcl-xL and MCL1. In contrast, eCypA stimulation still induced the expression of BCL-xL and MCL1 in CypA KO cells (Fig. 5F,G). CCK-8 assays demonstrated that eCypA enhanced the viability of ALL cells, which was inhibited by anti-CypA mAb in NALM6, RS4;11 and REH cells (Figs. 5H and EV6C,D). Similarly, eCypA did not increase the viability of MC2R knockout NALM6 cells (Fig. 5I) and eCypA still modestly increased cell viability in CypA knockout cells (Fig. 5J). Additionally, eCypA counteracted the pro-apoptotic effects of vincristine, whereas anti-CypA mAb reversed these effects in NALM6, RS4;11 and REH cells (Figs. 5K and EV6E,F). When eCypA was added to MC2R knockout cells, vincristine-mediated apoptosis was no longer attenuated (Fig. 5L). CypA KO cells exhibited higher basal apoptosis compared with wild-type cells, yet eCypA still partially attenuated vincristine-induced apoptosis (Fig. 5M). In contrast, under basal conditions, anti-CypA mAb alone did not significantly increase apoptosis (Appendix Fig. S3), and eCypA also had minimal effects on apoptosis in the absence of cytotoxic stress. qPCR analysis of PBMCs from patients with B-ALL and healthy donors revealed that the anti-apoptotic gene *Bcl-xL* was significantly upregulated in PBMCs from patients with B-ALL compared to those from healthy donors, and its expression showed a moderate positive correlation with eCypA protein levels and *MC2R* mRNA levels (Fig. EV6G–I). Given the heterogeneous nature of PBMCs, these results likely reflect a bulk-level association. In parallel, eCypA promotes leukemia cell survival capacity and resistance to cytotoxic stress through the MC2R–cAMP–PKA–CREB signaling pathway, accompanied by increased expression of genes such as BCL-xL and MCL1.

### Targeting eCypA modulates chemotherapy response and delays disease progression in B-ALL

**Figure 6:**
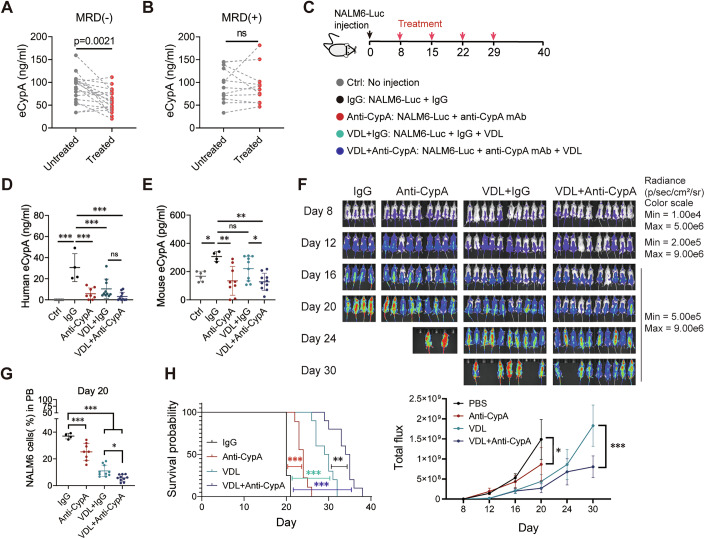
Anti-CypA mAb enhances chemotherapy efficacy and improves prognosis in ALL. (**A**, **B**) Peripheral blood (PB) samples were collected from patients classified as minimal residual disease (MRD)-negative (**A**, *n* = 18) or MRD-positive (**B**, *n* = 12). MRD status was determined by flow cytometry analysis of bone marrow samples at the end of induction therapy (day 28–30). Peripheral blood plasma samples collected at day 14 during induction therapy were used for eCypA measurement by ELISA. (**C**) NALM6-Luc cells were injected via the tail vein into NCG mice. Eight days later, mice were treated with IgG (*n* = 4), Anti-CypA (*n* = 9), VDL + IgG (*n* = 10), or VDL + anti-CypA (*n* = 10) by weekly intraperitoneal injection. (**D**, **E**) Human (**D**) and murine (**E**) eCypA levels in PB plasma were measured by ELISA on day 20. (**F**) Representative bioluminescence imaging of mice at the indicated time points following luciferin administration (top), with quantitative analysis using an in vivo imaging system (bottom). (**G**) Flow cytometric analysis of GFP⁺ leukemic cells in PB on day 20. (**H**) Kaplan–Meier survival analysis of mice in different treatment groups. Data were presented as mean ± SD; in (**A**, **B**), *n* represents individual patients. In (**D**–**H**), *n* represents individual mice. Statistical significance was determined using a paired two-tailed Student’s *t*-test for (**A**,** B**, **F**), one-way ANOVA for (**D**, **E**, **G**), and log-rank (Mantel–Cox) test for (**H**). *p < 0.05, **p < 0.01, ***p < 0.001; ns not significant. Source data are available online for this figure.

To investigate the relationship between eCypA levels and chemotherapeutic efficacy in B-ALL, we collected samples from patients with B-ALL and MRD-negative and -positive before and after chemotherapy. Two weeks after chemotherapy, eCypA levels significantly decreased in patients with MRD-negative (Fig. 6A) but remained unchanged in patients with MRD-positive (Fig. 6B), indicating a potential association between sustained eCypA levels and reduced chemotherapy response.

To investigate whether anti-CypA mAb therapy modulates chemotherapy efficacy, we established a NALM6-Luc mouse model to compare the effects of a low-dose, less toxic chemotherapy regimen (vincristine, dexamethasone, and L-asparaginase [VDL]) with or without anti-CypA mAb treatment (Fig. 6C). On day 20, eCypA levels in the PB were measured, revealing a significant reduction in human eCypA levels across all treatment groups compared with that in the control (Fig. 6D). Meanwhile, anti-CypA mAb significantly reduced the mouse eCypA levels, whereas VDL alone had little effect (Fig. 6E). Bioluminescence imaging demonstrated that anti-CypA mAb alone reduced B-ALL cell burden compared with the control group, and its combination with VDL resulted in a further reduction (Fig. 6F). Moreover, the combination of anti-CypA mAb and VDL further decreased the B-ALL cell count in PB (Fig. 6G). Survival analysis showed that anti-CypA mAb treatment modestly prolonged survival compared with IgG controls, and its combination with VDL resulted in a slight additional survival benefit compared with VDL alone (Fig. 6H). These findings suggest that targeting eCypA may contribute to therapeutic responses in B-ALL, although the magnitude of the effect is limited in this model.

### Anti-CypA mAb treatment reduces B-ALL cell burden and dissemination in patient-derived xenograft (PDX) models

**Figure EV7:**
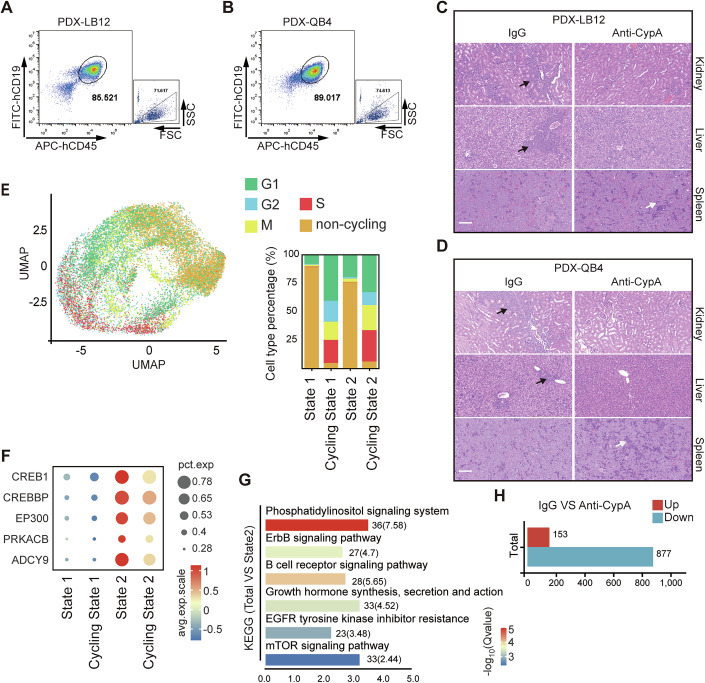
scRNA-seq analysis reveals CREB pathway activation and transcriptomic changes in a PDX model. (**A**, **B**) Representative flow cytometry plots showing the proportion of hCD19⁺hCD45⁺ leukemic cells in the spleen of first-generation PDX mice under the indicated conditions. (**C**, **D**) Representative hematoxylin and eosin (H&E) staining of kidney, liver, and spleen tissues from PDX-LB12 (**C**) and PDX-QB4 (**D**) mice on day 38. Black arrows indicate infiltrating B-ALL cells within the tissues, whereas white arrows indicate non–B-ALL cells. Scale bars, 100 μm. (**E**) UCell-based scoring of cell cycle gene signatures, classifying cells into non-cycling, G1, S, G2, and M phases. UMAP visualization of cell cycle states (right) and bar plot showing the distribution of cell cycle phases across different cell clusters (left). (**F**) Bubble plot showing elevated expression of CREB signaling-related genes in cells with multipotent progenitor features. (**G**) KEGG pathway enrichment analysis of genes upregulated in cells with multipotent progenitor features compared with total ALL cells. (**H**) Bar plot showing the number of upregulated and downregulated genes in Anti-CypA-treated versus IgG-treated ALL cells. Representative flow cytometry and histological images in (**A**–**D**) are shown from five independent mice per group with similar results. Source data are available online for this figure.

**Figure 7:**
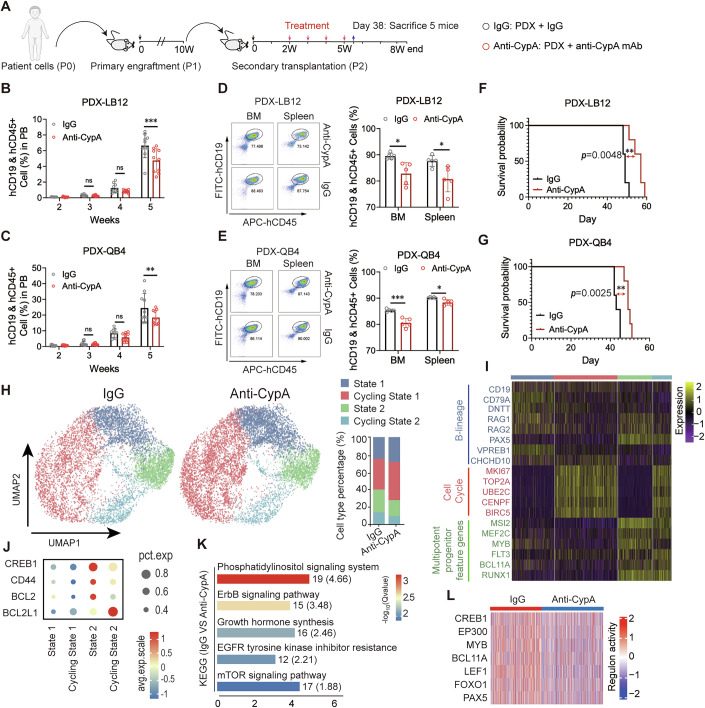
Anti-CypA mAb therapy inhibits the proliferation and dissemination of ALL cells and decreases the proportion of cells with multipotent progenitor features in a patient-derived xenograft (PDX) model. (**A**) Schematic diagram of PDX mouse model construction. (**B**–**C**) Flow cytometric analysis of human ALL cell proportions in peripheral blood (PB) of PDX-LB12 (**B**) and PDX-QB4 (**C**) mice during four weeks of Anti-CypA mAb treatment (*n* = 10). (**D**,** E**) Flow cytometric analysis of human ALL cell proportions in bone marrow (BM) and spleen of PDX-LB12 (**D**) and PDX-QB4 (**E**) mice on day 38 (*n* = 5). (**F**–**G**) Kaplan–Meier survival analysis of PDX-LB12 (**F**) and PDX-QB4 (**G**) mice treated with anti-CypA mAb or control IgG (*n* = 5). Survival differences were analyzed using the log-rank (Mantel–Cox) test. In (**F**), χ² = 7.962, df = 1, *p* = 0.0048; in (**G**), χ² = 9.151, df = 1, *p* = 0.0025. (**H**) UMAP plots (left) and corresponding bar plot (right) showing the distribution of cell states in IgG- and Anti-CypA–treated PDX-ALL samples. (**I**) Heatmap showing the expression of representative marker genes used for state annotation, including genes associated with B-lineage programs, cell cycle activity, and multipotent progenitor features. (**J**) Bubble plot showing the expression of representative genes across State 1, Cycling State 1, State 2, and Cycling State 2. (**K**) KEGG pathway enrichment analysis of downregulated genes following Anti-CypA mAb treatment. (**L**) Heatmap showing decreased transcription factor activity scores after Anti-CypA mAb treatment. Data were presented as mean ± SD; in (**B**–**G**), n represents individual mice per group. Statistical significance was determined using two-way ANOVA for (**B**–**E**), and log-rank (Mantel–Cox) test for (**F**,** G**). *p < 0.05, **p < 0.01, ***p < 0.001; ns not significant. Source data are available online for this figure.

To evaluate the potential therapeutic relevance of anti-CypA mAb in a clinically relevant setting, we established a PDX mouse model using the BM cells from patients with B-ALL. The clinical characteristics of these patients are summarized in Dataset EV3. By week 10, the first-generation xenograft mice exhibited ~57 and 10% human B-ALL cells in their PB, respectively (Appendix Fig. S4A,B). A substantial proportion of the leukocytes isolated from the spleens of first-generation xenograft mice were human B-ALL cells, exceeding 85% in both cases (Fig. EV7A,B). The second-generation xenograft mice were treated with an anti-CypA mAb starting at week 2, administered once weekly for four weeks (Fig. 7A). PB analysis revealed a significant reduction in B-ALL cell proportions following 4 weeks of anti-CypA mAb treatment compared to the controls (Fig. 7B,C). Similarly, antibody treatment significantly reduced the proportion of B-ALL cells in the BM and spleen (Fig. 7D,E). These findings suggest that the antibody reduces B-ALL cell burden in hematopoietic tissues and circulation. H&E staining revealed that antibody treatment reduced leukemic infiltration in the kidneys and livers and increased the proportion of immune cells in the spleen (Fig. EV7C,D). In addition, anti-CypA mAb treatment modestly prolonged survival in both PDX models (Fig. 7F,G). Overall, these results suggest that anti-CypA mAb may delay leukemia progression in PDX models.

### Single-cell transcriptomic analysis suggests that anti-CypA mAb reduces the proportion of cells with multipotent progenitor features and is associated with decreased CREB pathway activity

To further investigate the mechanism by which anti-CypA mAb affects B-ALL progression in second-generation xenograft mouse models, human CD45-positive cells were isolated from bone marrow cell suspensions for single-cell RNA sequencing (scRNA-seq). After stringent quality control and batch effect correction, 7370 cells in the IgG group and 8762 cells in the anti-CypA mAb group were analyzed and organized into transcriptionally defined subsets representing two major cellular states, termed State 1 and State 2 populations. All cells exhibited a B-lineage transcriptional identity but did not map to discrete stages of normal B-cell development; thus, a state-based classification was applied. State 2 was defined by upregulation of genes associated with multipotent progenitor features (*FLT3, MYB, MSI2, RUNX1, PBX3, MEF2C*, and *BCL11A*), whereas State 1 lacked this transcriptional program. Within each state, cells were further stratified into cycling and non-cycling fractions based on cell cycle activity, resulting in four functional subsets (Appendix Fig. S5). Following anti-CypA mAb treatment, the proportion of State 2 cells was reduced from 26.01 to 18.29%, and the proportion of Cycling State 2 cells decreased from 13.96 to 9.53% (Fig. 7H). To further characterize proliferative status, UCell-based scoring of cell cycle gene signatures was performed, confirming that cycling subsets exhibited elevated proliferative activity compared with their non-cycling counterparts (Fig. EV7E). The annotation of these subsets was supported by transcriptional features derived from B-lineage–associated genes, genes associated with multipotent progenitor features (e.g., *FLT3* and* MSI2*), and cell cycle–related genes (e.g., *MKI67* and* TOP2A*), as summarized in Appendix Fig. S5 and visualized in the heatmap (Fig. 7I). The State 2 cells exhibited high expression of CREB pathway–related genes, including key upstream factors (Fig. EV7F). Additionally, there was increased expression of CREB downstream targets, *Bcl-xl (BCL2L1)* and *CD44* (Fig. 7J). Together with the experimental data described above, these findings suggest that anti-CypA mAb treatment may reduce the abundance of State 2 cells in association with decreased CREB pathway activity. KEGG analysis revealed that State 2 cells were enriched in several pro-leukemic pathways associated with cell growth, hormone signaling, and therapy resistance (Fig. EV7G). Compared to the IgG group, anti-CypA mAb treatment resulted in the upregulation of 153 genes and downregulation of 877 genes across all B-ALL cells (Fig. EV7H). Notably, the downregulated genes were enriched in similar pro-leukemic pathways (Fig. 7K). Transcription factor activity analysis showed that anti-CypA mAb treatment significantly reduced the activity of several transcription factors, particularly CREB1 and its essential coactivator EP300 (Fig. 7L). These findings suggest that targeting CREB-related signaling is associated with changes in leukemic cell-state composition and decreased CREB pathway activity.

## Discussion

ALL is characterized by the uncontrolled proliferation of lymphoid precursor cells. In this study, we demonstrated that eCypA is significantly elevated in patients with B-ALL, contributing to disease progression by promoting adhesion and migration of B-ALL cells and reducing sensitivity to cell death. Mechanistically, eCypA binds to MC2R, activating the cAMP-PKA-CREB pathway and upregulating genes essential for adhesion and migration, including FN1, CD44, and MMP9, as well as cell survival-related genes, such as Bcl-xL and MCL1. Moreover, the anti-CypA mAb reduced leukemia burden and tissue infiltration in both NALM6 xenograft and patient-derived xenograft mouse models. These findings suggest that eCypA contributes to B-ALL progression and may have therapeutic relevance.

Consistent with the enhanced migratory phenotype, we observed that eCypA stimulation upregulated FN1, CD44, and MMP9. These molecules have been implicated in leukemic cell adhesion and extracellular matrix (ECM) remodeling. CD44 mediates leukemic cell interaction with the bone marrow microenvironment and cooperates with integrins such as VLA-4 to promote adhesion (Gutjahr et al, 2021; Wu et al, 2024), while FN1 provides structural support for cell motility (Longstreth and Wang, 2024), and MMP9 facilitates ECM degradation associated with leukemic cell invasion (Verma et al, 2020). Taken together, the coordinated upregulation of these genes suggests that eCypA may promote leukemic cell dissemination by enhancing cell–matrix interactions and ECM remodeling. However, we have not yet directly demonstrated that the pro-migratory effect of eCypA is mediated by the upregulated expression of FN1, CD44, and MMP9. Likewise, whether the anti-apoptotic effect of eCypA is attributable to increased BCL-XL and MCL1 expression remains to be determined in future studies.

It is widely accepted that CD147 is a receptor of eCypA (Zhu et al, 2015). Recent findings show that eCypA can bind multiple receptors (e.g., ACE2 and integrin β2) and activate various signaling pathways, including JAK-STAT3 and FAK-ERK-P65, thereby promoting inflammatory responses (Bai et al, 2024; Yang et al, 2024). In the present study, MC2R was identified to interact with eCypA and our data support that eCypA triggers the cAMP-PKA-CREB pathway in B-ALL. Notably, we identified that eCypA binds to the N-terminal (NT) region and the third extracellular loop (EC3) of MC2R. Peptide ligands typically engage GPCRs from the extracellular side and induce conformational changes required for receptor activation (Kim et al, 2025). Consistently, extracellular domains of GPCRs, including the N-terminus and extracellular loops, have been shown to play critical roles in ligand recognition and activation (Peeters et al, 2011). Therefore, the localization of eCypA binding to NT and EC3 is consistent with a model in which eCypA engages MC2R to initiate downstream signaling. This process is similar to the interaction of ACTH with MC2R, leading to the synthesis of corticosteroids (Metz et al, 2005), a pathway that can affect tumor progression in certain malignancies, such as small-cell lung cancer and prostate cancer (Clark et al, 2016; Hafiz et al, 2012). Additionally, MC2R is overexpressed in adrenal myelolipomas and congenital adrenal hyperplasia (Almeida et al, 2014), underscoring its potential oncogenic roles. A separate genetic study further indicated that the MC2R rs1893219 A > G variant might affect childhood B-ALL risk (de Carvalho et al, 2023). Therefore, our study suggests that eCypA binding to MC2R in B-ALL cell proliferation and migration and extends the relevance of MC2R signaling to B-ALL.

Single-cell RNA sequencing of B-ALL cells from a PDX mouse model identified transcriptionally distinct cell populations, including a subset characterized by elevated expression of genes associated with multipotent progenitor features (e.g., FLT3, MSI2, and MEF2C) (Dolence et al, 2014; Herglotz et al, 2016; Kharas et al, 2010) and higher CREB regulon activity. In the present study, treatment with anti-CypA mAb was associated with a reduction in the proportion of this cell population. These findings suggest that eCypA/MC2R signaling may contribute to the maintenance of specific leukemic cell states with multipotent progenitor features, thereby extending the biological relevance of CREB pathway activation in B-ALL.

CypA lacking a signal peptide is secreted via unconventional or vesicle-mediated pathways (e.g., in macrophages responding to LPS or oxidative stress) (Bai et al, 2024; Jin et al, 2000; Suzuki et al, 2006; Zhu et al, 2015). We demonstrated that B-ALL cells secrete eCypA into culture supernatants via autocrine signaling as a cytokine. Moreover, the results of the co-culture experiments revealed that B-ALL cells stimulated eCypA secretion from nearby cells. Therefore, B-ALL cells elevate eCypA levels via autocrine and paracrine mechanisms, thereby expanding our understanding of eCypA secretion in the tumor microenvironment. The higher eCypA levels detected in supernatants of cultured healthy donor cells may result from stress responses intrinsic to in vitro culture, where supraphysiological oxygen tension and altered nutrient conditions induce oxidative stress (Halliwell, 2003), subsequently triggering ER stress (Malhotra et al, 2008). These stress pathways are well known to enhance the secretion of cytokines and leaderless proteins such as CypA (Jin et al, 2000; Rubartelli et al, 1990), providing a plausible explanation for why cultured healthy donor cells release higher levels of eCypA than observed in the circulation of healthy individuals. We considered the possibility that soluble factors and cell adhesion events are involved in CypA secretion, which is interesting and worthy of further study.

In summary, our findings suggest that eCypA contributes to B-ALL progression and severity. Patients with B-ALL exhibit markedly increased eCypA levels, which interact with MC2R to enhance the transendothelial migration of leukemic cells and reduce sensitivity to apoptosis. Combining an anti-CypA mAb with standard chemotherapy (vincristine, dexamethasone, and L-asparaginase) further improved treatment responses in a preclinical model, supporting its potential therapeutic relevance. In addition, while our analysis of circulating eCypA levels in patients provides initial clinical insight, further validation in larger and well-annotated cohorts will be required. The regulatory mechanism of eCypA in other tumors also warrants further exploration.

## Methods

**Table:** Reagents and tools table

Reagent/resource	Reference or source	Identifier or catalog number
**Experimental models**		
NALM6, clone G5	ATCC	CRL-3273
RS4;11	ATCC	CRL-1873
REH	ATCC	CRL-8286
293T	ATCC	CRL-3216
HUVEC	ATCC	CRL-1730
C57BL/6JGpt	Gempharmatech	N000013
NCG mice	Gempharmatech	T001475
P190-BCR-ABL mouse model	This study	N/A
NALM6-luc mouse model	This study	N/A
B-ALL-PDX mouse model	This study	N/A
**Recombinant DNA**		
Flag-MC2R	YouBio	Cat# G124043
Flag-MRAP2	YouBio	Cat# F119180
p3xFLAG CMV10	Sigma	Cat# E7908
Flag-CypA	This study	N/A
pCMV-GFP	Addgene	Cat# 11153
GFP-MC2R fragment	This study	Appendix Table S1
pGL4.42[luc2P HRE Hygro]	Promega	Cat# E4001
**Antibodies**		
Anti-CypA monoclonal antibody	This study	N/A
DYKDDDDK Tag Antibody	Genscript	Cat# A01428-100
His Tag Antibody [HRP]	Genscript	Cat# A00612
MC2R Polyclonal Antibody	Signalway Antibody	Cat# 38534
GFP	Thermo Fisher Scientific	Cat# MA5-15256
Phospho-PKA C (Thr197) Antibody	Cell Signaling Technology	Cat# 4781
PKAalpha cat (C-20)	Santa Cruz Biotechnology	Cat# sc-903
PCREB	Proteintech	Cat# 81871-1-RR
Rabbit Anti-CREB-1, phospho (Ser133) Polyclonal antibody	Santa Cruz Biotechnology	Cat# sc-7978
CREB (48H2) Rabbit mAb	Cell Signaling Technology	Cat# 9197
Rabbit Anti-GAPDH Monoclonal Antibody	Cell Signaling Technology	Cat# 2118
GAPDH Monoclonal antibody	Proteintech	Cat# 60004
Fibronectin antibody	Proteintech	Cat# 15613-1-AP
CD44 Polyclonal antibody	Proteintech	Cat# 15675-1-AP
MMP9 (2C3)	Santa Cruz Biotechnology	Cat# sc-21733
Anti-MMP9 antibody	Abcam	Cat# ab283575
Bcl-xL (H-5)	Santa Cruz Biotechnology	Cat# sc-8392
Anti-Bcl-XL antibody [E18]	Abcam	Cat# ab32370
Mcl-1 Antibody (22)	Santa Cruz Biotechnology	Cat# sc-12756
Goat anti-human IgG Fc antibody	Biodragon	Cat# BF02020
Beta Actin Monoclonal antibody	Proteintech	Cat# 66009-1-Ig
APC-conjugated anti-human CD45	Thermo Fisher Scientific	Cat# 17-0459-42
FITC-conjugated anti-human CD19	BioLegend	Cat# 302206
CD45 Monoclonal Antibody (30-F11), APC, eBioscience	Thermo Fisher Scientific	Cat# 17-0451-82
Recombinant Alexa Fluor® 488 Anti-CD19 antibody	Abcam	Cat# ab196468
**Oligonucleotides and other sequence-based reagents**		
MC2R-siRNA1	This study	N/A
MC2R-siRNA2	This study	N/A
GAPDH-siRNA	This study	N/A
Ctrl-siRNA	This study	N/A
MC2R-sgRNA	This study	N/A
PPIA-sgRNA	This study	N/A
PCR primer	This study	Appendix Table S2
**Chemicals, enzymes and other reagents**		
Huam CypA ELISA kit	Cusabio	Cat# CSB-E09920h
Mouse CypA ELISA Kit	Cusabio	Cat# CSB-E09282m
Ficoll-Paque PLUS	cytiva	Cat# 17144002
ACK Lysis Buffer	Servicebio	Cat# G2015-500ML
Penicillin/streptomycin	Gibco	Cat# 15140122
PRMI1640	Gibco	Cat# 11875093
DMEM	Gibco	Cat# 11885084
D-Luciferin,potassium salt	YEASEN	Cat# 40902ES
Mouse IgG1 isotype control-InVivo	Selleckchem	Cat# A2106
Animal-Free IL-7 Protein, Mouse (His)	MedChemExpress	Cat# HY-P700217AF
IL-3 Protein, Mouse	MedChemExpress	Cat# HY-P7062
Lipofectamine 2000	Thermo Fisher Scientific	Cat# 11668019
RNAiMAX	Thermo Fisher Scientific	Cat# 13778075
LIVE/DEAD™ fixable violet dead cell stain kit	Thermo Fisher Scientific	Cat# L34964
OPTI-MEM	Gibco	Cat# HY-P7062
TRIzol Reagent	Sigma-Aldrich	Cat# T9424
Anti-His Affinity Resin	Genscript	Cat# L00439
Protein A-Agarose	Santa cruz biotechnology	Cat# sc-2001
Recombinant Human IgG1 Fc	BioLegend	Cat# 773006
Anti-DYKDDDDK IP Resin	Genscript	Cat# L00425
cAMP Elisa kit	Feiyubio	Cat# MM-0006H1
Firefly Glo Luciferase Reporter Gene Assay Kit	YEASEN	Cat# 11404ES
CREB inhibitor 666-15	MedChemExpress	Cat# HY-101120
H-8, Dihydrochloride	Sigma-Aldrich	Cat# 371958
CellTracker Green CMFDA	YEASEN	Cat# 40721ES
Hoechst 33342	Thermo Fisher Scientific	Cat# 62249
*Evo M-MLV* Plus cDNA Synthetic reagent kit	Accurate biology	Cat# AG11615
ChamQ Universal SYBR qPCR Master Mix	Vazyme	Cat# Q711
SteadyPure Universal RNA Extraction Kit	Accurate biology	Cat# AG21017
APC Annexin V Apoptosis Detection Kit with PI	BioLegend	Cat# 640932
Annexin V Binding Buffer	BioLegend	Cat# 422201
Cell Counting Kit (CCK-8)	YEASEN	Cat# 40203ES
vincristine	MedChemExpress	Cat# HY-N0488
dexamethasone	MedChemExpress	Cat# HY-14648
L-asparaginase	MedChemExpress	Cat# HY-P1923
OVA Conjugated Adrenocorticotropic Hormone (ACTH)	Uscnk	Cat# CPA836Hu21
**Software**		
iProx	www.iprox.cn	
R Studio	https://rstudio.com/products/rstudio/download	
FlowJo software	https://www.flowjo.com/	
ImageJ software	https://imagej.nih.gov/ij/	
SnapGene	https://www.snapgene.com	
Adobe Illustrator	https://www.adobe.com	
GraphPad Prism	https://www.graphpad.com	
Saiviewer	www.servicebio.cn	
IVIS system	https://www.perkinelmer.com/	
ZEN software	https://www.zeiss.com	
**Other**		
IVIS Spectrum imaging system	PerkinElmer	
CytoFLEX LX flow cytometer	Beckman Coulter	
Synergy Neo2 multimode microplate detector	BioTek Instruments	
CFX96 real-time PCR system	Bio-Rad	
Axio Vert A1 inverted fluorescence microscope	Carl Zeiss	
Q Exactive Hybrid Quadrupole-Orbitrap Mass Spectrometer	Thermo Fisher Scientific	
Amersham ImageQuant 800	Cytiva	

### Clinical samples and animal studies

Clinical samples and information were collected from the Department of Oncology of the Seventh Affiliated Hospital of Sun Yat-sen University under the approved IRB protocol KY-2024-415-01. Written informed consent was obtained from all participants. The experiments conformed to the principles set out in the WMA Declaration of Helsinki and the Department of Health and Human Services Belmont Report. All animal experiments were approved by the Ethics Committee of Shenzhen Bay Laboratory (AELWJ202301) and conducted in compliance with the relevant guidelines and regulations. Mice were randomly assigned to experimental groups using a random number generator, and investigators were blinded to group allocation during treatment and analysis. Only mice exhibiting engraftment of human CD19⁺ cells in peripheral blood were included in the study, while those that failed to engraft or developed unrelated health issues were excluded.

### Survival analysis

Gene expression data were obtained from the UCSC Xena platform (https://xenabrowser.net/), which provides processed and normalized transcriptomic data from the TARGET-ALL-P2 cohort (Data ref: Genomic Data Commons, TARGET-ALL-P2). Corresponding clinical information was retrieved from the Genomic Data Commons (GDC) Data Portal (https://portal.gdc.cancer.gov/). Expression and clinical data were matched at the individual patient level. Only patients diagnosed with B-cell acute lymphoblastic leukemia (B-ALL) were included in the analysis, and cases of other leukemia subtypes were excluded. Overall survival (OS) was used as the primary endpoint. Cox proportional hazards regression models were applied to evaluate the prognostic significance of *PPIA* mRNA level, which was treated as a continuous variable. Both univariate and multivariate Cox analyses were performed. In multivariate models, age and gender were included as covariates. Fusion status was additionally included in models where sufficient data were available. To account for disease stage and sample source, patients were stratified into three groups: initial peripheral blood (PB), initial bone marrow (BM), and relapse bone marrow (BM). Survival analyses were performed separately for each group. Kaplan–Meier survival curves were generated for each subgroup to visualize survival differences between patients with high and low *PPIA* mRNA levels. Patients were stratified using the median expression level as the cutoff, and statistical significance was evaluated using the log-rank test.

### Cell lines

NALM6, RS4;11, and REH cells were maintained in RPMI-1640 medium (Gibco, Carlsbad, CA, USA) supplemented with 10% fetal bovine serum (FBS) (Hyclone, Logan, UT, USA), 100 U/mL penicillin, and 100 µg/mL streptomycin at 37 °C in a humidified atmosphere containing 5% CO₂. The 293T and HUVEC cells were cultured in DMEM (Gibco) with 10% FBS, 100 U/mL penicillin, and 100 µg/mL streptomycin under the same conditions. Cell lines were authenticated using short tandem repeat (STR) profiling. All cell lines were tested for Mycoplasma contamination using PCR-based assays before experiments.

### Cell isolation

PB was collected from healthy donors into EDTA-coated tubes and processed within 2 h. For WBC isolation, blood was diluted 1:1 with PBS (pH 7.4), treated with ACK buffer for erythrocyte lysis, incubated for 5 min at room temperature, and centrifuged at 300×*g* for 10 min. The WBC pellet was washed twice with PBS. For PBMCs, diluted blood was layered over Ficoll-Paque™ PLUS, centrifuged at 400 × g for 30 min, and the PBMC layer was aspirated, washed, and resuspended. Granulocytes (GC) were obtained from the Ficoll pellet, treated with ACK buffer, washed, and resuspended. Cell count and viability were assessed using trypan blue exclusion.

### Anti-CypA monoclonal antibody and validation

The anti-CypA mouse monoclonal antibody was generated against recombinant human CypA and purified by Protein A chromatography. The antibody detects a predominant band at ~18 kDa corresponding to endogenous CypA and exhibits cross-reactivity with both human and mouse CypA, as validated by Western blot analysis in human leukemia cell lines and mouse-derived tissues (Appendix Fig. S6). Antibody specificity was further supported by reduced signal under CypA-depleted conditions. The antibody is not humanized.

### MC2R and CypA knockout cell line construction

MC2R and CypA (*PPIA*) knockout NALM6 cells were generated using the CRISPR/Cas9 ribonucleoprotein (RNP) system. Guide RNAs targeting MC2R (5′-GGGCAGCCTATATAAGATCT-3′) and *PPIA* (5′-TGGTGGCAAGTCCATCTATG-3′) were synthesized and complexed with recombinant Cas9 protein at a molar ratio of 1:1.2 in 30 μL Opti-MEM and incubated at room temperature for 15 min to allow RNP formation. A total of 2 × 10^5^ NALM6 cells were electroporated with the RNP complexes using a Nucleofector device (Lonza) under the following conditions: 540 V, 20 ms, 1 pulse. Cells were then transferred into pre-warmed complete RPMI-1640 medium for recovery. Single-cell clones were obtained by limiting dilution and expanded in 96-well plates. Genomic DNA from individual clones was subjected to PCR amplification followed by Sanger sequencing to confirm gene editing. Biallelic knockout clones were used for subsequent experiments. Loss of protein expression was further validated by immunoblotting.

### Conditioned medium assay and co-culture assay

#### Direct co-culture assay

ALL cells and peripheral blood-derived cells were mixed at a 1:1 ratio and co-seeded in 24-well plates at 1 × 10^6^ cells each per well in 1 mL of serum-free medium. After 24 h of co-culture, culture supernatants were collected and subjected to eCypA ELISA. Monocultured cells were processed in parallel under the same medium and incubation conditions.

#### Indirect co-culture assay

Peripheral blood-derived cells (1 × 10^6^ cells/well) were seeded in the lower chamber of a 24-well Transwell system, and ALL cells (1 × 10^6^ cells/insert) were seeded in the upper insert (0.4-μm pore size) in serum-free medium. After 24 h of co-culture, supernatants from the indicated chamber(s) were collected for eCypA ELISA. Cells cultured alone under the same conditions served as controls.

#### Conditioned medium assay

ALL cells were cultured in serum-free medium at 1 × 10^6 cells/mL for 24 h. The conditioned medium was harvested, centrifuged to remove cells, and filtered through a 0.22-μm filter. Peripheral blood-derived cells were then cultured with conditioned medium mixed 1:1 with fresh serum-free medium for the indicated time, and supernatants were collected for eCypA measurement. Fresh medium or mock-treated medium processed in parallel was used as a control.

### Transcriptomics

NALM6 cells were treated with 10 ng/mL recombinant CypA alone or in combination with 20 ng/mL anti-CypA mAb for 1 h. Subsequent experiments were conducted by Gene Denovo Biotechnology Co., Ltd. The raw sequence data reported in this study have been deposited at the China National Center for Bioinformation/Beijing Institute of Genomics, Chinese Academy of Sciences (GSA-Human: HRA010096) and are publicly accessible at https://ngdc.cncb.ac.cn/gsa-human.

#### NALM6-Luc mouse model

NCG mice were purchased from GemPharmatech (Nanjing, China). Animals were housed under specific pathogen-free (SPF) conditions. NALM6-Luc cells expressing luciferase were generated via lentiviral transfection and selected with puromycin. For the mouse model, 5 × 10⁵ cells were injected into the tail veins of mice, followed by treatment with recombinant CypA (1 mg/kg) or CypA mAb (5 mg/kg, weekly) via intraperitoneal injection. Bioluminescence imaging (IVIS, PerkinElmer) was performed weekly using D-luciferin (150 mg/kg).

Mice were divided into three groups: antibody-only, VDL chemotherapy (vincristine 0.2 mg/kg, dexamethasone 3.5 mg/kg, L-asparaginase 1.2 mg/kg), and VDL + anti-CypA mAb (5 mg/kg weekly). Treatment began on day 8 post-cell injection, after confirming disease using bioluminescence imaging. Bioluminescence signal intensity (photons/s) was quantified to monitor progression. On day 20, eCypA levels in PB plasma were measured via ELISA, and GFP-positive cells in PB were analyzed using flow cytometry. Survival was monitored, and survival curves were generated using the log-rank test.

### p190-BCR-ABL1-induced murine B-ALL model

Six- to eight-week-old C57BL/6 mice were maintained under specific pathogen-free conditions. All animal experiments were approved by the Institutional Animal Care and Use Committee. To establish the p190-BCR-ABL1-induced murine B-ALL model, bone marrow (BM) cells were harvested from the femurs and tibias of donor mice and transduced twice with MSCV-BCR-ABL1-IRES-GFP retroviral supernatant in the presence of IL-7 (10 ng/mL) and IL-3 (10 ng/mL). Transduced cells were washed, resuspended in sterile buffer, and 1 × 10^6^ cells were injected into primary recipient mice via the tail vein following a single dose of 700 cGy irradiation. Leukemia engraftment was monitored by flow cytometry, and successful model establishment was defined by the detection of mCD45⁺GFP⁺ leukemic cells in peripheral blood. For secondary transplantation, spleens from primary recipient mice were collected at week 2. Splenocytes were prepared by mechanical dissociation followed by red blood cell lysis, and 2 × 10^6^ cells were injected into secondary recipient mice via the tail vein. For therapeutic studies, an anti-CypA monoclonal antibody was administered at 5 mg/kg once weekly starting the day after transplantation and continued until the experimental endpoint.

### Patient samples and MRD assessment

Peripheral blood and bone marrow samples were collected from patients with B-cell acute lymphoblastic leukemia (B-ALL) and healthy donors. Clinical characteristics and sample information for all patients included in this study are summarized in Table EV1.

For cross-sectional analysis, peripheral blood plasma samples collected at diagnosis or relapse were used to measure eCypA levels, and patients were grouped as newly diagnosed B-ALL or relapsed B-ALL according to clinical records. Healthy donor samples were included as controls. In addition, paired peripheral blood plasma and bone marrow plasma samples from B-ALL patients were used to compare eCypA levels between the two compartments.

For MRD-related analysis, peripheral blood plasma samples collected on day 14 during induction therapy were used for eCypA measurement. Minimal residual disease (MRD) status was determined by routine flow cytometry analysis of bone marrow samples at the end of induction therapy (days 28–30). Patients were classified as MRD-positive or MRD-negative based on the presence or absence of detectable residual leukemic cells. Detailed MRD assessment results are provided in Table EV1. Plasma eCypA levels were measured by enzyme-linked immunosorbent assay (ELISA) according to the manufacturer’s instructions.

#### PDX mouse model

Primary engraftment was performed by injecting patient-derived bone marrow white blood cells (WBCs) into NCG mice to establish first-generation xenografts. Secondary transplantation was carried out by reinjecting ALL cells isolated from the spleens of first-generation xenografts into new recipient mice, thereby generating second-generation PDX models.

ALL cells were obtained from diagnostic bone marrow samples collected at initial presentation from two male pediatric B-ALL patients, designated as PDX-LB12 (12 years old) and PDX-QB4 (4 years old). These samples were cryopreserved and subsequently used for xenograft establishment.

Genetic features of the donor samples were determined by RNA-seq–based transcriptomic analysis. PDX-LB12 harbored a KMT2A::AFF1 fusion together with an FLT3-ITD mutation, whereas PDX-QB4 showed no recurrent fusion but carried an FLT3 Y572C mutation. Detailed genetic information is provided in Dataset EV3.

Immunophenotypic characterization was based on clinical diagnostic flow cytometry reports derived from the same bone marrow samples. Both samples showed high expression of B-lineage markers, including CD19 and CD22, together with expression of precursor-associated markers such as CD34 and HLA-DR. Expression levels of additional markers, including CD10, CD20, CD38, CD79a, and myeloid-associated markers (CD13 and CD33), are summarized in Dataset EV3; Appendix Fig. S7.

For both PDX-LB12 and PDX-QB4 models (*n* = 20 per model), mice were randomly assigned to control (*n* = 10) or anti-CypA mAb treatment groups (*n* = 10). Leukemia burden was monitored by flow cytometry using APC-conjugated anti-human CD45 and FITC-conjugated anti-human CD19 antibodies to identify human B-ALL cells.

First-generation PDX cells were harvested from spleens at week 10 and transplanted into secondary recipient mice (1 × 10⁶ cells per mouse). Anti-CypA mAb (5 mg/kg) was administered weekly starting from week 2 post-transplantation. Peripheral blood was collected weekly to monitor leukemia burden. On day 38, five mice per group were sacrificed for assessment of human ALL cell proportions in spleen and bone marrow, and the remaining mice were followed for survival analysis.

### Single-cell RNA sequencing and analysis

Bone marrow cells were isolated from IgG- and anti-CypA mAb-treated PDX-LB12 mice, and human CD45-positive cells were enriched by magnetic beads, each comprising pooled samples from four mice. Single-cell RNA sequencing was performed using the 10x Genomics platform by Gene Denovo (Guangzhou, China), including library preparation, sequencing, and initial data processing (quality control, alignment, and generation of gene expression matrices). Downstream analyses, including dimensionality reduction, cell clustering, marker-based annotation, differential gene expression, KEGG enrichment, and transcription factor activity inference, were conducted using Gene Denovo’s online single-cell analysis platform. Cell-state annotation of leukemic subclusters was performed based on established B-lineage developmental and proliferation-associated marker genes. All cells were first defined as B-lineage leukemic cells based on the expression of canonical B-cell markers (*CD19, CD79A, VPREB1, DNTT, RAG1*, and *RAG2*) (Kennedy et al, 2020; Morgan and Tergaonkar, 2022; Wentink et al, 2019), Cycling State 1 and Cycling State 2 cells were identified by enrichment of cell cycle–associated genes (*MKI67, TOP2A, UBE2C, CENPF*, and *BIRC5*), and State 2 cells were defined by the upregulation of genes associated with multipotent progenitor features (*FLT3, MYB, MSI2, RUNX1, PBX3, BCL11A*, and *MEF2C*) (Dolence et al, 2014; Guo et al, 2017; Herglotz et al, 2016; Kharas et al, 2010; Niebuhr et al, 2013; Sandberg et al, 2005; Yu et al, 2012). Cell cycle activity was further evaluated using UCell-based scoring of curated cell cycle gene signatures, and cells were assigned to non-cycling or proliferative states according to relative enrichment scores (Dataset EV4). The dataset has been deposited in GSA-Human (China National Center for Bioinformation) under accession number HRA012096 and is publicly available at https://ngdc.cncb.ac.cn/gsa-human.

### Histopathology and immunohistochemistry

Spleen and liver tissues were harvested from euthanized mice, fixed in 10% neutral-buffered formalin, embedded in paraffin, and sectioned at 4-μm thickness. For histopathological evaluation, tissue sections were stained with hematoxylin and eosin (H&E) according to standard procedures. For immunohistochemistry (IHC), sections were deparaffinized, rehydrated, and subjected to heat-induced antigen retrieval, followed by blocking of endogenous peroxidase activity and nonspecific binding. Sections were incubated with primary antibodies against human CD19 or phospho-CREB, followed by HRP-conjugated secondary antibodies and visualization using diaminobenzidine (DAB). Nuclei were counterstained with hematoxylin. Representative images were acquired using a bright-field microscope, and positive staining areas were quantified using ImageJ as previously described (Li et al, 2023).

### Mass spectrometry

Mass spectrometry was performed to identify CypA-interacting proteins. NALM6 and RS4;11 cells were treated with 10 µg/mL human IgA or CypA-Fc for 2 h at 37 °C, followed by cell lysis to obtain whole-cell protein extracts. Protein complexes were captured using Fc-mediated pull-down, and the eluted proteins were reduced, alkylated, and digested with trypsin. The resulting peptides were analyzed by liquid chromatography–tandem mass spectrometry (LC–MS/MS) on a high-resolution mass spectrometer (e.g., Q Exactive, Thermo Fisher Scientific). Raw data were processed using Proteome Discoverer with the Sequest HT algorithm against the UniProt human database, and protein identifications were filtered at a false discovery rate (FDR) <1%, with only proteins achieving a Sequest HT score >1 retained for downstream analysis. The mass spectrometry proteomics data have been deposited to the ProteomeXchange Consortium via the iProX partner repository with the dataset identifier PXD076421.

### Validation of CypA-MC2R interaction

293T cells were transfected with Flag-MC2R or co-transfected with Flag-CypA and GFP-tagged MC2R fragments. Protein interactions were assessed using His pull-down assays with His-CypA and Flag-MC2R lysates, followed by western blotting. Co-IP experiments were performed with anti-FLAG beads to detect FLAG and GFP-tagged proteins. GFP-tagged proteins were purified using GFP-Trap beads and confirmed by SDS-PAGE and Coomassie staining. ELISA binding assays were conducted to evaluate the interaction between His-CypA and GFP-tagged fragments, with or without anti-CypA mAb, by measuring OD at 450 nm. Fc-CypA binding assays used Protein A beads to detect interactions with GFP-tagged fragments, verifying the blocking effect of anti-CypA mAb as previously described (Bai et al, 2024). Sequences of MC2R extracellular segments are provided in Appendix Table S1.

### Quantitative reverse-transcription PCR

Total RNA was extracted using TRIzol reagent according to the manufacturer’s instructions and reverse-transcribed into cDNA using a commercial reverse-transcription kit. Quantitative PCR was performed using SYBR Green I Master Mix on a CFX96 real-time PCR detection system (Bio-Rad) under standard cycling conditions. Relative gene expression levels were calculated using the 2^−ΔΔCt^ method with GAPDH as the internal control. Primer sequences were based on OriGene reference sequences and synthesized commercially. All primer sequences used in this study are provided in Appendix Table S2. Experimental procedures were performed as previously described (Pei et al, 2023).

### eCypA detection using ELISA

eCypA levels in cell culture supernatants and mouse or human plasma samples were measured using a human Cyclophilin A ELISA kit (CUSABIO) according to the manufacturer’s instructions. Absorbance was measured using a Synergy Neo2 multimode microplate detector (BioTek Instruments), and protein concentrations were calculated based on standard curves. Assays were performed following standard procedures as previously described (Bai et al, 2024).

### cAMP detection using ELISA

Intracellular cAMP levels were measured using a cAMP ELISA kit (Camilo, Nanjing, China) according to the manufacturer’s instructions. After treatment, cell lysates were collected and processed for cAMP quantification. Absorbance at 450 nm was measured using a Synergy Neo2 multimode microplate detector (BioTek Instruments), and cAMP concentrations were calculated based on the standard curve provided in the kit.

### siRNA transfection

Cells were transfected with siRNAs targeting MC2R using Lipofectamine RNAiMAX transfection reagent (Thermo Fisher Scientific) according to the manufacturer’s instructions. The siRNA sequences used were as follows: MC2R si1, GCACAGGAGCAACGCGGAG; MC2R si2, GGGGAGCGGCGCCCAGCAG. A non-targeting control siRNA was used as a negative control where indicated. Cells were harvested and analyzed 48 h after transfection. The procedure was performed as previously described (Pei et al, 2025).

### Nuclear and cytoplasmic fraction

Cells were harvested, washed with cold PBS, and resuspended in hypotonic lysis buffer supplemented with protease and phosphatase inhibitors. After incubation on ice, samples were centrifuged to separate the cytoplasmic supernatant from the nuclear pellet. The nuclear pellet was then washed and lysed in nuclear extraction buffer to obtain nuclear proteins. Protein concentrations were determined, and equal amounts of cytoplasmic and nuclear extracts were subjected to immunoblotting analysis. The procedure was performed as previously described (Pei et al, 2025).

### Luciferase reporter assay

293T cells were co-transfected with CRE-luciferase reporter constructs together with a Renilla luciferase control plasmid using standard transfection procedures. After transfection, cells were treated with CypA in the presence or absence of anti-CypA mAb for the indicated times. Firefly luciferase activity was measured using the Firefly Glo Luciferase Reporter Gene Assay Kit (Yeasen), and luminescence signals were detected using a Synergy Neo2 multimode microplate detector (BioTek Instruments). Relative luciferase activity was normalized to Renilla luciferase activity. The assay was performed as previously described (Pei et al, 2023).

### Transendothelial migration assay

Transendothelial migration assays were performed using Transwell inserts with 3-μm pore-size polycarbonate membranes in 24-well companion plates. Inserts were coated with Matrigel, and HUVECs were seeded in the upper chamber and cultured until a confluent endothelial monolayer was formed. NALM6 cells were labeled with carboxyfluorescein diacetate succinimidyl ester (CFSE) and added to the upper chamber. After incubation for 24 h at 37 °C, cells that migrated into the lower chamber were collected and quantified. Representative fluorescence images were acquired using an Axio Vert A1 inverted fluorescence microscope (Carl Zeiss), and fluorescence intensity was measured using a microplate reader.

### Cell Counting Kit-8 (CCK-8) assay

Cell viability was assessed using a Cell Counting Kit-8 (CCK-8) assay after 48 h of treatment with CypA in the presence or absence of anti-CypA mAb. After incubation with CCK-8 reagent according to the manufacturer’s instructions, absorbance was measured using a Synergy Neo2 multimode microplate detector (BioTek Instruments). Cell viability was calculated relative to the untreated control group. The assay was performed as previously described (Pei et al, 2023).

### Flow cytometry

Flow cytometry was performed to assess apoptosis and cell-surface marker expression. For apoptosis analysis, cells were stained with Annexin V-FITC and propidium iodide (PI) according to the manufacturer’s instructions. For immunophenotypic analysis, cells were stained with the indicated fluorochrome-conjugated antibodies, including antibodies against CD19 and CD45, where applicable. After washing, samples were acquired on a CytoFLEX LX flow cytometer (Beckman Coulter), and data were analyzed using FlowJo software. The procedures were performed as previously described (Pei et al, 2025).

### Statistical analysis

All results are expressed as the mean ± standard deviation (SD). Mouse experiments were performed with random assignment to treatment groups, and investigators were blinded to group allocation during treatment and analysis where applicable. All in vitro experiments were independently repeated at least three times unless otherwise indicated. Statistical significance was determined using the Student’s *t*-test for comparisons between two groups, one-way ANOVA for comparisons among multiple groups with a single factor, and two-way ANOVA for comparisons involving two factors. Kaplan–Meier survival curves were analyzed using the log-rank test (Mantel–Cox). A *p* value <0.05 was considered significant (*p < 0.05, **p < 0.01, and ***p < 0.001). All *p* values were summarized in Appendix Table S3. Statistical analyses were performed using GraphPad Prism 10.

## Supplementary information

Appendix

Table EV2

Table EV1

Peer Review File

Dataset EV1

Dataset EV2

Dataset EV3

Dataset EV4

Source data Fig. 1

Source data Fig. 2

Source data Fig. 3

Source data Fig. 4

Source data Fig. 5

Source data Fig. 6

Source data Fig. 7

Figure EV1 Source Data

Figure EV2 Source Data

Figure EV3 Source Data

Figure EV4 Source Data

Figure EV5 Source Data

Figure EV6 Source Data

Figure EV7 Source Data

Expanded View Figures

## Data Availability

The datasets produced in this study are available in the following databases: Single-cell RNA-seq data: GSA-Human (CNCB-NGDC) HRA012096 and HRA010096 (https://ngdc.cncb.ac.cn/gsa-human). Mass spectrometry proteomics data: ProteomeXchange Consortium via the iProX repository PXD076421 (https://www.iprox.cn/). The source data of this paper are collected in the following database record: biostudies:S-SCDT-10_1038-S44321-026-00466-w.
