# Supplementary material for: Extracellular cyclophilin A promotes B-cell acute lymphoblastic leukemia progression via MC2R
Source: EMBO Mol Med. 2026 Jun 10;18(8):3028–56. doi: 10.1038/s44321-026-00466-w (PMC13469882; doi:10.1038/s44321-026-00466-w)
Supplement: Supplementary file 1 [file 44321_2026_466_MOESM1_ESM.pdf]

Appendix

Extracellular cyclophilin A promotes B-Cell acute lymphoblastic leukemia progression via MC2R

Han Zhong Pei,[1,2,8] Heqiao Li,[1,2,3,8] Hongman Xue,[4,8] Wenqing Wang,[4,8] He Zhang,[1,2] Zeqiu Fan,[3] Xiaoyuan Bai,[3,5] Fei Han,[2] Qingmei Li,[1] Yuna Zhao,[3] Lu Yu,[4] Xiaoxiao Jia,[3] Yao Guo,[4,*] Wenjun Liu,[1,2,3,*] Lei Sun[3,6,7,*]

Appendix Figure S1. Cross-reactivity validation of the eCypA ELISA kits between human and mouse samples……………………………………………………………. 2

Appendix Figure S2. Validation of *MC2R* mRNA levels in NALM6 cells by qPCR melting curve analysis…………………………………………………………... 3

Appendix Figure S3. Effects of anti-CypA monoclonal antibody alone on apoptosis in B-ALL cells..………………………………………………………………. 4

Appendix Figure S4. Flow cytometric analysis of hCD19$^+$hCD45$^+$ double-positive cells in peripheral blood and spleen in first-generation PDX mice.…………………... 5

Appendix Figure S5. Transcriptional features underlying cell state annotation in scRNA-seq analysis.……………………………………………………………… 6

Appendix Figure S6. Validation of CypA interaction in human leukemic cells and murine hematopoietic tissues……………………………………………………. 7

Appendix Figure S7. Representative clinical flow cytometry analysis of donor samples used to establish PDX-ALL models. …………………………………………………. 8

Appendix Table S1. Nucleotide sequences of MC2R extracellular fragments…...…. 9

Appendix Table S2. Primer sequences used for quantitative real-time PCR.…...…… 9

Appendix Table S3. All p values in this study……………………………………..10

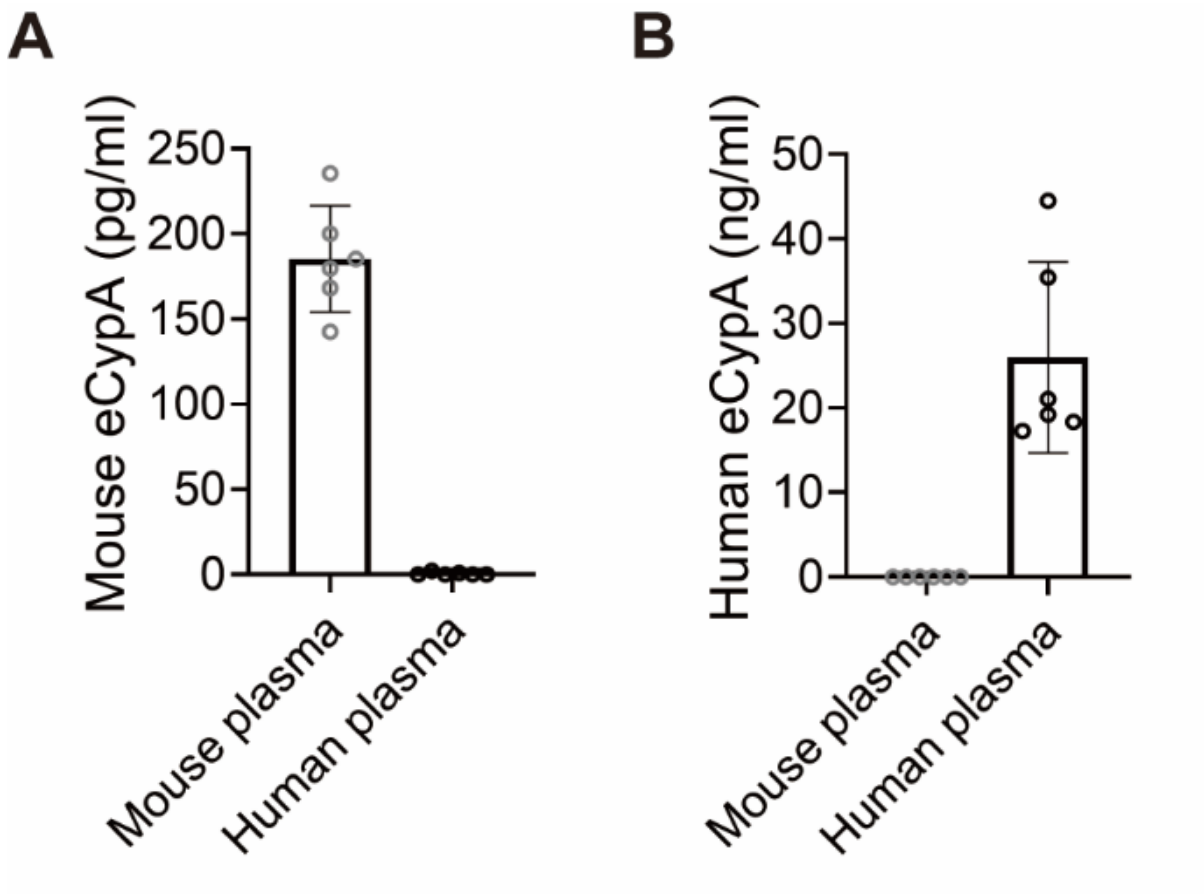

Appendix Figure S1. Cross-reactivity validation of the eCypA ELISA kits between human and mouse samples. (A–B) The same set of peripheral blood plasma samples from mice and humans were measured using the mouse eCypA ELISA kit (A) and the human eCypA ELISA kit (B), respectively (n = 6).

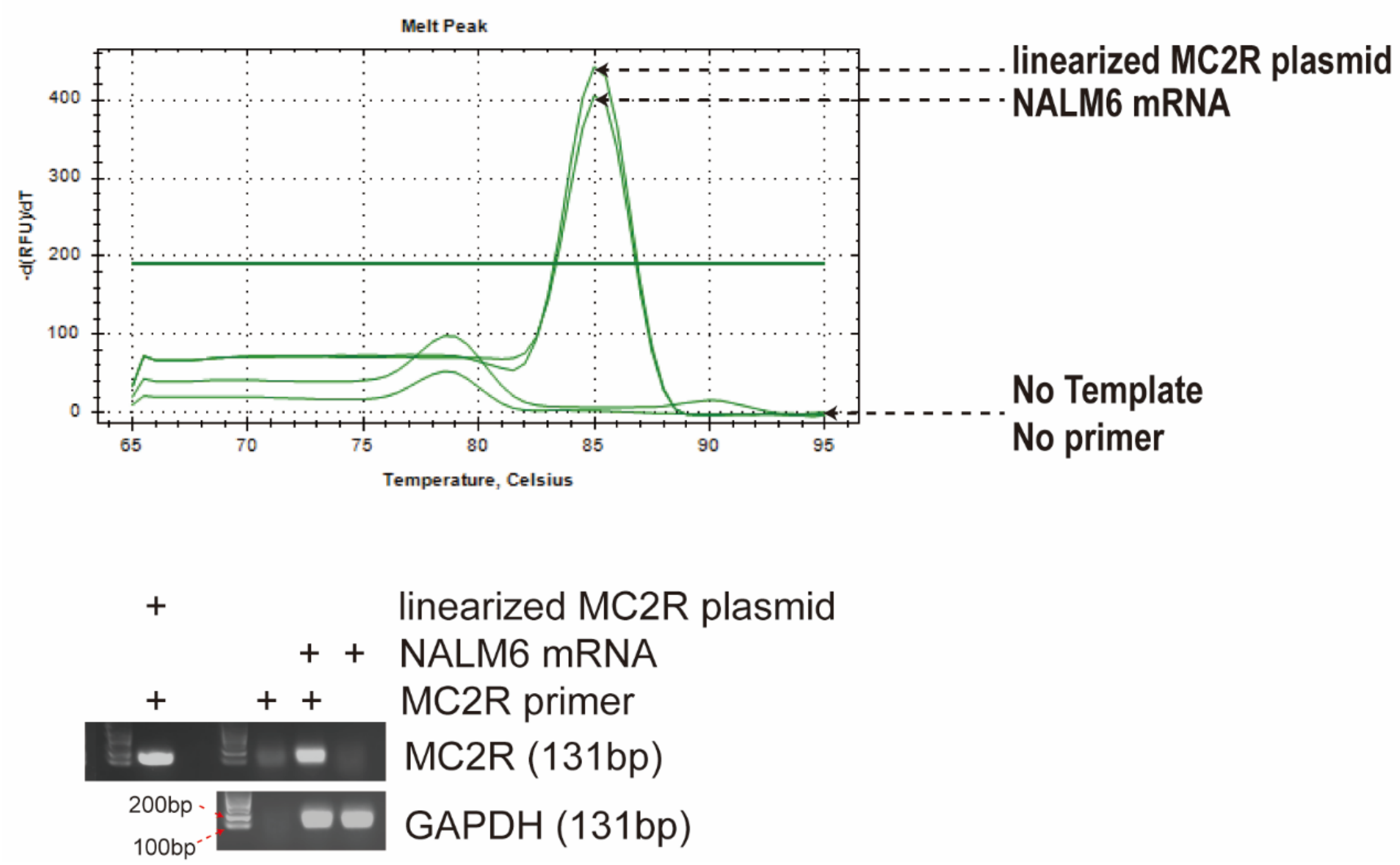

Appendix Figure S2. Validation of *MC2R* RNA levels in NALM6 cells by qPCR melting curve analysis. Melting curve analysis was performed following qPCR amplification using MC2R-specific primers. A linearized MC2R plasmid was used as a positive control. NALM6-derived mRNA was used as the experimental sample with MC2R primers. Negative controls included reactions containing mRNA only or primers only. GAPDH was used as an internal control for all samples except the linearized plasmid.

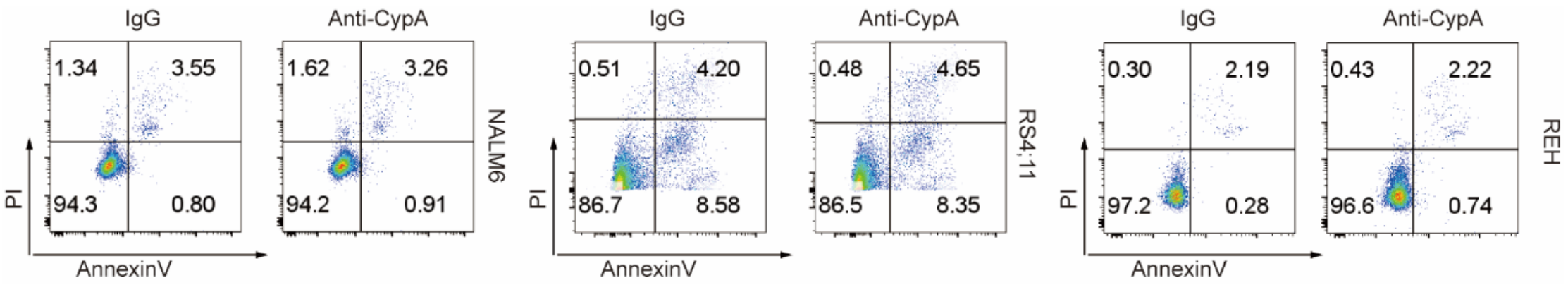

Appendix Figure S3. Effects of anti-CypA monoclonal antibody alone on apoptosis in B-ALL cells. NALM6, RS4;11, and REH cells were treated with IgG control or anti-CypA monoclonal antibody at 10 ng/mL for 36 h, followed by Annexin V/PI staining to assess apoptosis.

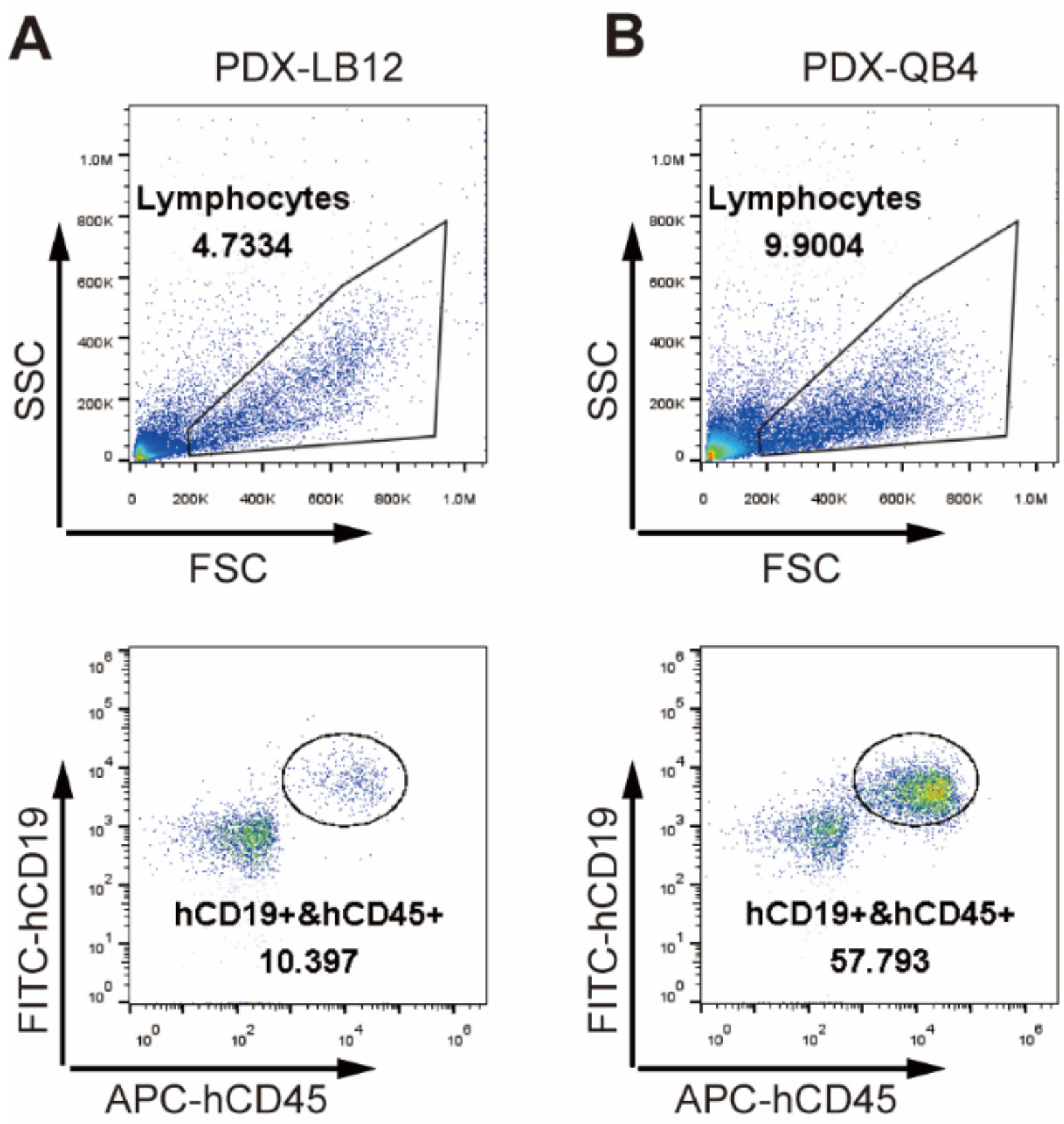

Appendix Figure S4. Flow cytometric analysis of hCD19^{+}hCD45^{+} double-positive cells in peripheral blood and spleen in first-generation PDX mice. (A–B) Proportions of hCD19^{+}hCD45^{+} double-positive cells were detected using flow cytometry in peripheral blood (PB) from the first-generation PDX mice injected with BM samples of two patients with ALL, PDX-LB12 (A) and PDX-QB4 (B) via the tail vein for 10 weeks. (C–D) Proportions of hCD19^{+}hCD45^{+} double-positive cells in the spleen were detected using flow cytometry under the same conditions as A and B.

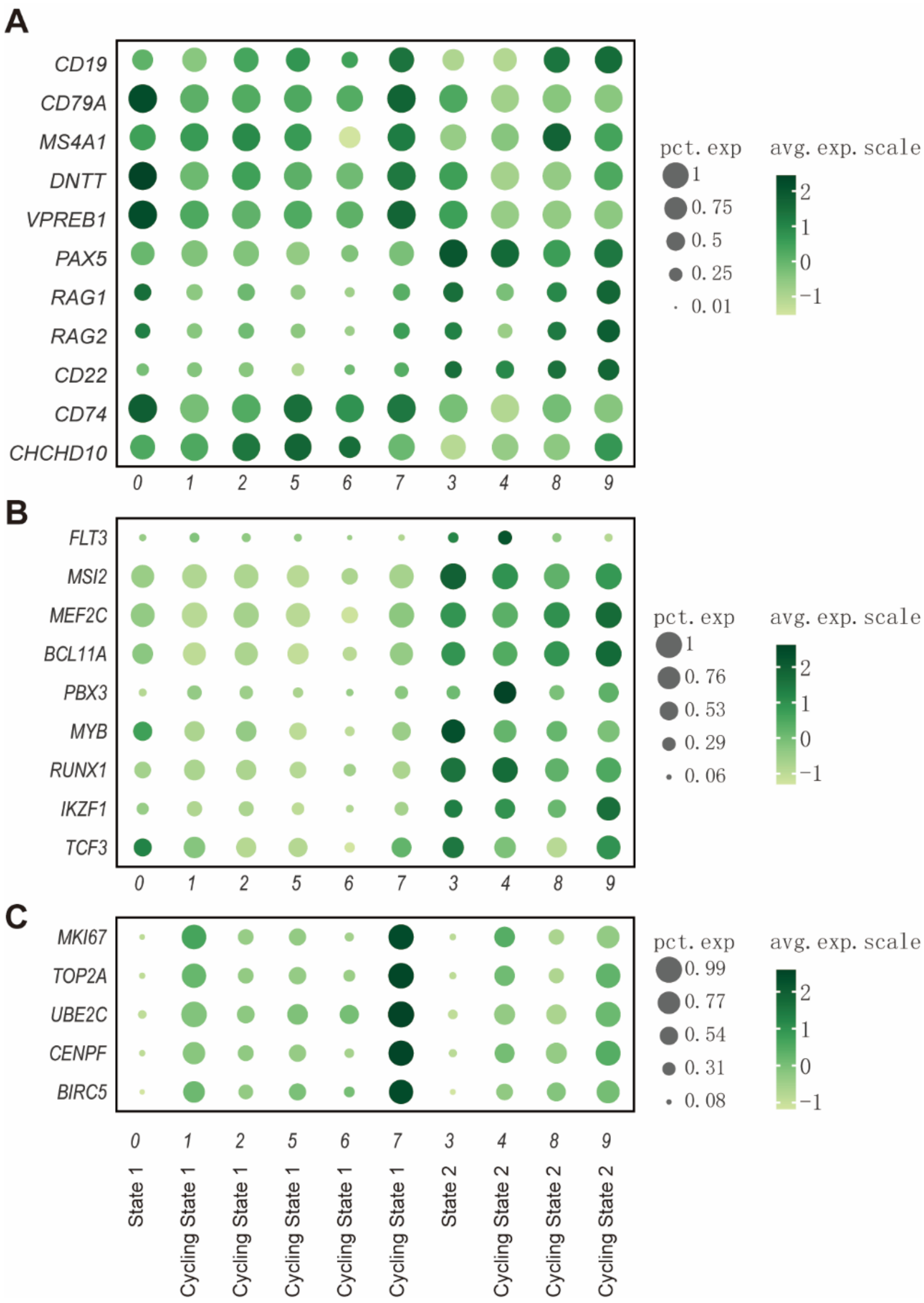

Appendix Figure S5. Transcriptional features underlying cell state annotation in scRNA-seq analysis. (A) Dot plot showing the expression of B-lineage–associated genes across the identified cell states. Canonical B-cell markers were broadly and consistently expressed across all states, supporting a homogeneous B-lineage identity. (B) Dot plot showing the expression of genes associated with progenitor-related transcriptional programs (*FLT3*, *MYB*, *MSI2*, *RUNX1*, *PBX3*, *BCL11A*, and *MEF2C*) across the defined states, highlighting their enrichment in State 2. (C) Dot plot showing the expression of cell cycle–related genes (*MKI67*, *TOP2A*, *UBE2C*, *CENPF*, and *BIRC5*), indicating proliferative activity and supporting the identification of Cycling State 1 and Cycling State 2 subpopulations.

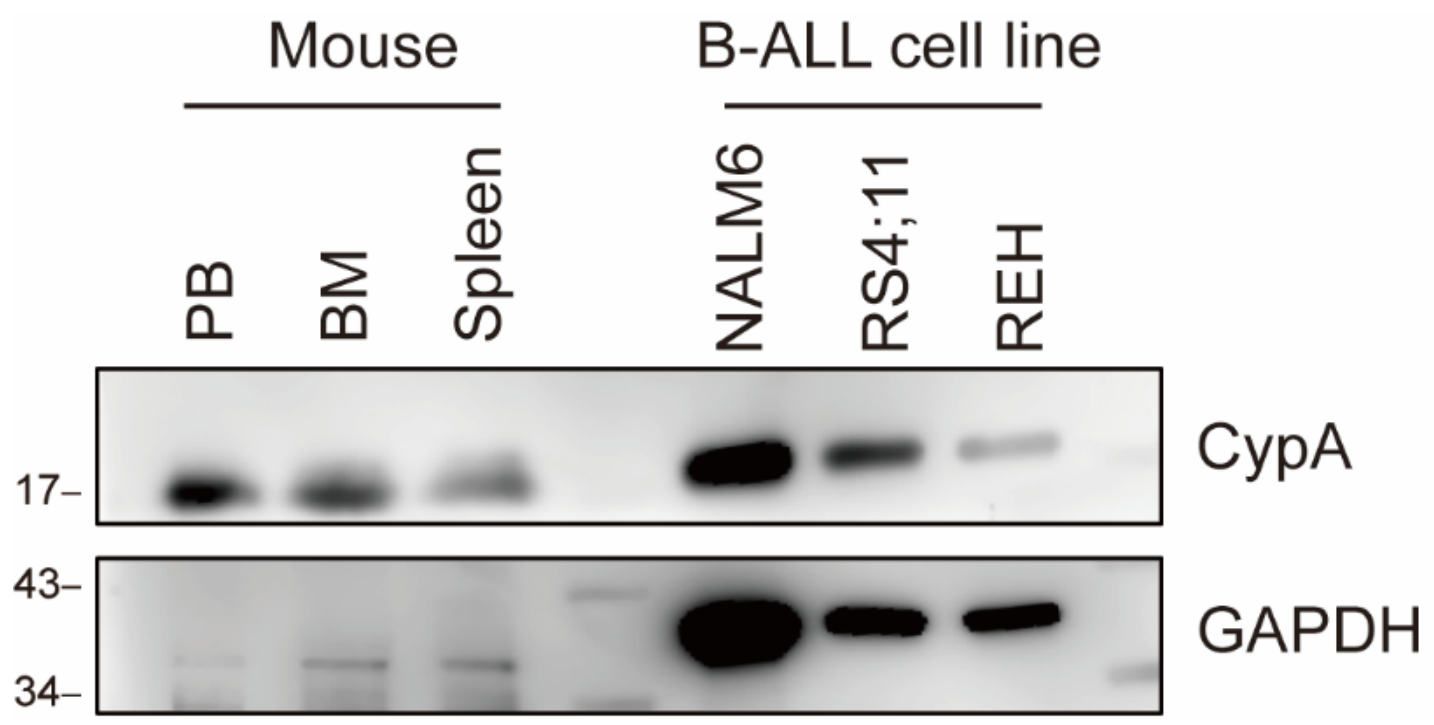

Appendix Figure S6. Validation of CypA interaction in human leukemic cells and murine hematopoietic tissues.

Red blood cells were lysed from mouse PB, bone marrow (BM), and spleen prior to protein extraction. Whole-cell lysates from these murine tissues, together with human B-ALL cell lines (NALM6, RS4;11, and REH), were subjected to western blot analysis to detect CypA. GAPDH was used as a loading control.

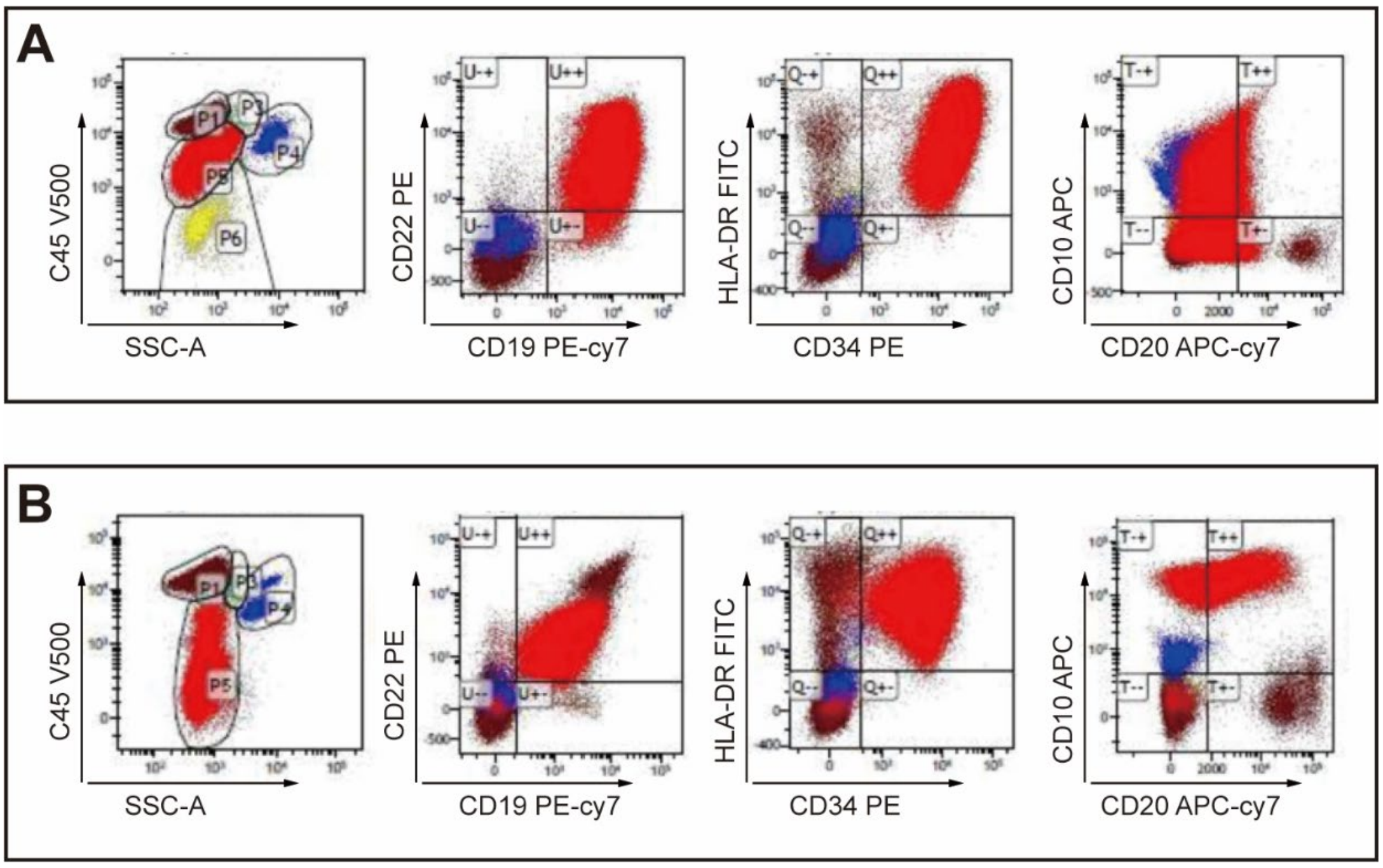

Appendix Figure S7. Representative clinical flow cytometry analysis of donor samples used to establish PDX-ALL models.

(A) PDX-LB12 and (B) PDX-QB4 donor samples. Flow cytometry data were obtained from diagnostic bone marrow samples at initial presentation, which were subsequently cryopreserved and used to establish the corresponding PDX models. Leukemic blast populations were identified in the CD45dim/SSC low region (P5 gate), and subsequent analyses were performed on gated blast cells. Both samples show strong expression of B-lineage markers (CD19 and CD22). PDX-LB12 exhibits a more immature immunophenotype, characterized by high CD34 and HLA-DR expression, with relatively low CD10 and CD20 levels. In contrast, PDX-QB4 displays a more differentiated pre-B phenotype with high CD10 and CD20 expression. Comprehensive immunophenotypic data derived from clinical diagnostic flow cytometry reports are summarized in Dataset EV3.

Appendix Table S1. Nucleotide sequences of MC2R extracellular fragments

	Sequences
MC2R NT	ATGAAGCACATTATCAACTCGTATGAAAACATCAACAA CACAGCAAGAAATAATTCCGACTGTCCTCGT
MC2R EC1	TTGAGAAACATGGGCTATCTCAAGCCACGTGGCAGTTTT GAAACCACAGCC
MC2R EC2	TCCCATCATGTGCCCACAGTGATCACCTTCACGTCG
MC2R EC3	ACATTCTGCCCAAGTAACCCCTACTGCGCCTGCTACATG TCT

Appendix Table S2. Primer sequences used for quantitative real-time PCR.

MC2R	F	GTGGTGCTTACGGTCATCTGGA
	R	AGGCACAGGATGAAGACCAGCA
GAPDH	F	GTCTCCTCTGACTTCAACAGCG
	R	ACCACCCTGTTGCTGTAGCCAA
FN1	F	ACAACACCGAGGTGACTGAGAC
	R	GGACACAACGATGCTTCCTGAG
CD44	F	CCAGAAGGAACAGTGGTTTGGC
	R	ACTGTCCTCTGGGCTTGGTGTT
MMP9	F	GCCACTACTGTGCCTTTGAGTC
	R	CCCTCAGAGAATCGCCAGTACT
BCL-xl	F	GCCACTTACCTGAATGACCACC
	R	AACCAGCGGTTGAAGCGTTCCT
MCL1	F	CCAAGAAAGCTGCATCGAACCAT
	R	CAGCACATTCCTGATGCCACCT

Appendix Table S3. All p values in this study.

Figure	Comparison	Statistical test	p value
1A	HD vs. B-ALL	One-way ANOVA	<0.0001
	HD vs. Relapsed	One-way ANOVA	<0.0001
1B	PB vs BM	Unpaired two-tailed Student's t-test	0.0053
1C	PB vs. Relapsed	One-way ANOVA	<0.0001
	BM vs. Relapsed	One-way ANOVA	<0.0001
1D	eCypA level vs WBC count	Linear correlation analysis	0.0003
	eCypA level vs lymphocyte count	Linear correlation analysis	0.0004
1E	Initial BM High vs low PPIA expression survival	Log-rank (Mantel–Cox) test	0.0163
	Initial PB High vs low PPIA expression survival	Log-rank (Mantel–Cox) test	0.0638
	Relapse BM High vs low PPIA expression survival	Log-rank (Mantel–Cox) test	0.0025
1F	NALM6 24 Hours vs 48 Hours	One-way ANOVA	<0.0001
	RS4;11 24 Hours vs 48 Hours	One-way ANOVA	<0.0001
	REH 24 Hours vs 48 Hours	One-way ANOVA	<0.0001
1G	WBCs vs. NALM6	One-way ANOVA	0.0065
	WBCs vs. RS4;11	One-way ANOVA	0.0085
	WBCs vs. N-CM+WBCs	One-way ANOVA	0.0068
	WBCs vs. R-CM+WBCs	One-way ANOVA	0.0457
1I	PB-WBCs vs. NALM6	One-way ANOVA	0.1062
	PB-WBCs vs. Indirect	One-way ANOVA	0.0174
	PB-WBCs vs. Direct	One-way ANOVA	0.0002
1J	PBMCs vs. Indirect	One-way ANOVA	0.0011
	PBMCs vs. Direct	One-way ANOVA	0.0017
1K	PB-GCs vs. Indirect	One-way ANOVA	0.015
	PB-GCs vs. Direct	One-way ANOVA	0.0003
1L	BM-WBCs vs. NALM6-CM	One-way ANOVA	0.0146
	BM-WBCs vs. Indirect	One-way ANOVA	0.0008
	BM-WBCs vs. Direct	One-way ANOVA	<0.0001
1M	BMMCs vs. NALM6-CM	One-way ANOVA	0.1212
	BMMCs vs. Indirect	One-way ANOVA	0.0006
	BMMCs vs. Direct	One-way ANOVA	0.0003
1N	BM-GCs vs. NALM6-CM	One-way ANOVA	0.0339
	BM-GCs vs. Direct	One-way ANOVA	<0.0001
	BM-GCs vs. Indirect	One-way ANOVA	0.0004
2B	IgG vs. Ctrl	One-way ANOVA	0.0185
	IgG vs. Anti-CypA	One-way ANOVA	0.0251
2C	IgG vs. Ctrl	One-way ANOVA	0.0009
	IgG vs. Anti-CypA	One-way ANOVA	0.0024
2D	CypA vs. IgG	Student's t-test	0.0453
	IgG vs. Anti-CypA	Student's t-test	0.02
2E	IgG vs. CypA	One-way ANOVA	<0.0001
	IgG vs. Anti-CypA	One-way ANOVA	0.0003
2G	IgG vs. Ctrl	One-way ANOVA	0.0006
	IgG vs. Anti-CypA	One-way ANOVA	0.0021

2H	BM- IgG vs. Anti-CypA	Two-way ANOVA	0.0328
	Spleen- IgG vs. Anti-CypA	Two-way ANOVA	0.0007
3E	0:Ctrl vs. 10:Ctrl	Two-way ANOVA	0.1482
	0:Ctrl vs. 30:Ctrl	Two-way ANOVA	<0.0001
	0:Ctrl vs. 60:Ctrl	Two-way ANOVA	<0.0001
	0:MC2R vs. 10:MC2R	Two-way ANOVA	0.0021
	0:MC2R vs. 30:MC2R	Two-way ANOVA	<0.0001
	0:MC2R vs. 60:MC2R	Two-way ANOVA	<0.0001
	0:MRAP2 vs. 10:MRAP2	Two-way ANOVA	0.9951
	0:MRAP2 vs. 30:MRAP2	Two-way ANOVA	0.2415
	0:MRAP2 vs. 60:MRAP2	Two-way ANOVA	0.0012
	0:MC2R+MRAP2 vs. 10:MC2R+MRAP2	Two-way ANOVA	0.1346
	0:MC2R+MRAP2 vs. 30:MC2R+MRAP2	Two-way ANOVA	<0.0001
	0:MC2R+MRAP2 vs. 60:MC2R+MRAP2	Two-way ANOVA	<0.0001
	60:Ctrl vs. 60:MC2R	Two-way ANOVA	<0.0001
	60:Ctrl vs. 60:MRAP2	Two-way ANOVA	0.7487
	60:Ctrl vs. 60:MC2R+MRAP2	Two-way ANOVA	<0.0001
	60:MC2R vs. 60:MRAP2	Two-way ANOVA	<0.0001
	60:MC2R vs. 60:MC2R+MRAP2	Two-way ANOVA	<0.0001
	60:MRAP2 vs. 60:MC2R+MRAP2	Two-way ANOVA	<0.0001
3F	NALM6 IgG vs. IgG+CypA	Two-way ANOVA	<0.0001
	NALM6 IgG+CypA vs. CypA+Anti-CypA	Two-way ANOVA	<0.0001
	RS4;11 IgG vs. IgG+CypA	Two-way ANOVA	<0.0001
	RS4;11 IgG+CypA vs. CypA+Anti-CypA	Two-way ANOVA	<0.0001
	REH IgG vs. IgG+CypA	Two-way ANOVA	<0.0001
	REH IgG+CypA vs. CypA+Anti-CypA	Two-way ANOVA	<0.0001
3G	Ctrl.si	Two-way ANOVA	<0.0001
	GAPDH.si	Two-way ANOVA	<0.0001
	MC2R.si1	Two-way ANOVA	0.1766
	MC2R.si2	Two-way ANOVA	0.8855
3H	WT:Ctrl vs. WT:CypA	Two-way ANOVA	<0.0001
	WT:Ctrl vs. MC2R KO:Ctrl	Two-way ANOVA	0.1207
	WT:Ctrl vs. MC2R KO:CypA	Two-way ANOVA	0.8525
	WT:CypA vs. MC2R KO:Ctrl	Two-way ANOVA	<0.0001
	WT:CypA vs. MC2R KO:CypA	Two-way ANOVA	<0.0001
	MC2R KO:Ctrl vs. MC2R KO:CypA	Two-way ANOVA	0.5699
3L	IgG+CypA vs. IgG	One-way ANOVA	0.0003
	IgG+CypA vs. CypA+Anti-CypA	One-way ANOVA	0.0009
3M	Ctrl.si	Two-way ANOVA	<0.0001
	GAPDH.si	Two-way ANOVA	<0.0001
	MC2R.si1	Two-way ANOVA	0.4795
	MC2R.si2	Two-way ANOVA	>0.9999
3N	IgG vs. CypA	One-way ANOVA	0.0028
	IgG vs. Anti-CypA	One-way ANOVA	0.0435
4C	FN1 IgG vs. CypA+IgG	Two-way ANOVA	<0.0001

	FN1 CypA+IgG vs. CypA+Anti-CypA	Two-way ANOVA	<0.0001
	CD44 IgG vs. CypA+IgG	Two-way ANOVA	<0.0001
	CD44 CypA+IgG vs. CypA+Anti-CypA	Two-way ANOVA	0.0003
	MMP9 IgG vs. CypA+IgG	Two-way ANOVA	<0.0001
	MMP9 CypA+IgG vs. CypA+Anti-CypA	Two-way ANOVA	<0.0001
4E	FN1 DMSO vs. CypA	Two-way ANOVA	<0.0001
	FN1 CREB inhibitor vs. CREB inhibitor + CypA	Two-way ANOVA	0.4587
	FN1 PKA inhibitor vs. PKA inhibitor + CypA	Two-way ANOVA	0.2146
	CD44 DMSO vs. CypA	Two-way ANOVA	<0.0001
	CD44 CREB inhibitor vs. CREB inhibitor + CypA	Two-way ANOVA	0.7531
	CD44 PKA inhibitor vs. PKA inhibitor + CypA	Two-way ANOVA	0.9635
	MMP9 DMSO vs. CypA	Two-way ANOVA	<0.0001
	MMP9 CREB inhibitor vs. CREB inhibitor + CypA	Two-way ANOVA	0.169
	MMP9 PKA inhibitor vs. PKA inhibitor + CypA	Two-way ANOVA	0.4034
4F	FN1 WT vs. WT+CypA	Two-way ANOVA	<0.0001
	FN1 MC2R KO vs. MC2R KO+CypA	Two-way ANOVA	0.0774
	CD44 WT vs. WT+CypA	Two-way ANOVA	<0.0001
	CD44 MC2R KO vs. MC2R KO+CypA	Two-way ANOVA	0.4365
	MMP9 WT vs. WT+CypA	Two-way ANOVA	<0.0001
	MMP9 MC2R KO vs. MC2R KO+CypA	Two-way ANOVA	0.0774
4H	FN1 WT vs. WT+CypA	Two-way ANOVA	<0.0001
	FN1 CypA KO vs. CypA KO+CypA	Two-way ANOVA	<0.0001
	CD44 WT vs. WT+CypA	Two-way ANOVA	<0.0001
	CD44 CypA KO vs. CypA KO+CypA	Two-way ANOVA	<0.0001
	MMP9 WT vs. WT+CypA	Two-way ANOVA	<0.0001
	MMP9 CypA KO vs. CypA KO+CypA	Two-way ANOVA	<0.0001
4J	Adhesion IgG+CypA vs. IgG	One-way ANOVA	<0.0001
	Adhesion IgG+CypA vs. CypA+Anti-CypA	One-way ANOVA	<0.0001
	Trans-endothelial IgG+CypA vs. IgG	One-way ANOVA	0.0028
	Trans-endothelial IgG+CypA vs. CypA+Anti-CypA	One-way ANOVA	0.0015
4K	Adhesion WT Ctrl vs. CypA	Two-way ANOVA	<0.0001
	Adhesion MC2R KO Ctrl vs. CypA	Two-way ANOVA	0.0007
	Adhesion WT:CypA vs. MC2R KO:CypA	Two-way ANOVA	<0.0001
	Trans-endothelial WT Ctrl vs. CypA	Two-way ANOVA	<0.0001
	Trans-endothelial MC2R KO Ctrl vs. CypA	Two-way ANOVA	0.0239
	Trans-endothelial WT:CypA vs. MC2R KO:CypA	Two-way ANOVA	0.0002
4L	Adhesion WT:Ctrl vs. WT:CypA	Two-way ANOVA	<0.0001
	Adhesion WT:CypA vs. CypA KO:CypA	Two-way ANOVA	0.0008
	Adhesion CypA KO:Ctrl vs. CypA KO:CypA	Two-way ANOVA	0.0001
	Trans-endothelial WT:Ctrl vs. WT:CypA	Two-way ANOVA	0.0005
	Trans-endothelial WT:CypA vs. CypA KO:CypA	Two-way ANOVA	0.3137
	Trans-endothelial CypA KO:Ctrl vs. CypA KO:CypA	Two-way ANOVA	0.002
5A	Bcl-xl IgG vs. CypA+IgG	Two-way ANOVA	<0.0001
	Bcl-xl IgG vs. CypA+Anti-CypA	Two-way ANOVA	0.7933
	Bcl-xl CypA+IgG vs. CypA+Anti-CypA	Two-way ANOVA	<0.0001

	MCL1 IgG vs. CypA+IgG	Two-way ANOVA	<0.0001
	MCL1 IgG vs. CypA+Anti-CypA	Two-way ANOVA	0.0699
	MCL1 CypA+IgG vs. CypA+Anti-CypA	Two-way ANOVA	<0.0001
5C	Bcl-xl DMSO vs. CypA	Two-way ANOVA	<0.0001
	Bcl-xl CREB inhibitor vs. CREB inhibitor + CypA	Two-way ANOVA	0.3468
	Bcl-xl PKA inhibitor vs. PKA inhibitor + CypA	Two-way ANOVA	0.0185
	MCL1 DMSO vs. CypA	Two-way ANOVA	<0.0001
	MCL1 CREB inhibitor vs. CREB inhibitor + CypA	Two-way ANOVA	0.7859
	MCL1 PKA inhibitor vs. PKA inhibitor + CypA	Two-way ANOVA	0.4095
5D	Bcl-xl WT vs. WT+CypA	Two-way ANOVA	<0.0001
	Bcl-xl MC2R KO vs. MC2R KO+CypA	Two-way ANOVA	0.9935
	MCL1 WT vs. WT+CypA	Two-way ANOVA	<0.0001
	MCL1 MC2R KO vs. MC2R KO+CypA	Two-way ANOVA	0.0821
5F	Bcl-xl WT vs. WT+CypA	Two-way ANOVA	<0.0001
	Bcl-xl CypA KO vs. CypA KO+CypA	Two-way ANOVA	<0.0001
	MCL1 WT vs. WT+CypA	Two-way ANOVA	<0.0001
	MCL1 CypA KO vs. CypA KO+CypA	Two-way ANOVA	0.0001
5H	0:CypA vs. 5:CypA	Two-way ANOVA	>0.9999
	0:CypA vs. 10:CypA	Two-way ANOVA	0.1055
	0:CypA vs. 20:CypA	Two-way ANOVA	0.0002
	0:CypA vs. 40:CypA	Two-way ANOVA	<0.0001
	0:CypA vs. 80:CypA	Two-way ANOVA	<0.0001
	0:Anti-CypA vs. 5:Anti-CypA	Two-way ANOVA	>0.9999
	0:Anti-CypA vs. 10:Anti-CypA	Two-way ANOVA	>0.9999
	0:Anti-CypA vs. 20:Anti-CypA	Two-way ANOVA	>0.9999
	0:Anti-CypA vs. 40:Anti-CypA	Two-way ANOVA	>0.9999
	0:Anti-CypA vs. 80:Anti-CypA	Two-way ANOVA	0.7946
	0:CypA+Anti-CypA vs. 5:CypA+Anti-CypA	Two-way ANOVA	0.9985
	0:CypA+Anti-CypA vs. 10:CypA+Anti-CypA	Two-way ANOVA	>0.9999
	0:CypA+Anti-CypA vs. 20:CypA+Anti-CypA	Two-way ANOVA	>0.9999
	0:CypA+Anti-CypA vs. 40:CypA+Anti-CypA	Two-way ANOVA	0.9996
	0:CypA+Anti-CypA vs. 80:CypA+Anti-CypA	Two-way ANOVA	0.9996
	80:CypA vs. 80:Anti-CypA	Two-way ANOVA	<0.0001
	80:CypA vs. 80:CypA+Anti-CypA	Two-way ANOVA	<0.0001
	80:Anti-CypA vs. 80:CypA+Anti-CypA	Two-way ANOVA	0.9195
5I	0:WT vs. 5:WT	Two-way ANOVA	0.8243
	0:WT vs. 10:WT	Two-way ANOVA	0.1396
	0:WT vs. 20:WT	Two-way ANOVA	<0.0001
	0:WT vs. 40:WT	Two-way ANOVA	<0.0001
	0:WT vs. 80:WT	Two-way ANOVA	<0.0001
	0:MC2R KO vs. 5:MC2R KO	Two-way ANOVA	>0.9999
	0:MC2R KO vs. 10:MC2R KO	Two-way ANOVA	>0.9999
	0:MC2R KO vs. 20:MC2R KO	Two-way ANOVA	>0.9999
	0:MC2R KO vs. 40:MC2R KO	Two-way ANOVA	>0.9999
	0:MC2R KO vs. 80:MC2R KO	Two-way ANOVA	0.3708

	80:WT vs. 80:MC2R KO	Two-way ANOVA	<0.0001
5J	0:WT vs. 5:WT	Two-way ANOVA	0.0618
	0:WT vs. 10:WT	Two-way ANOVA	0.0415
	0:WT vs. 20:WT	Two-way ANOVA	0.0015
	0:WT vs. 40:WT	Two-way ANOVA	<0.0001
	0:WT vs. 80:WT	Two-way ANOVA	<0.0001
	0:CypA KO vs. 5:CypA KO	Two-way ANOVA	0.759
	0:CypA KO vs. 10:CypA KO	Two-way ANOVA	0.139
	0:CypA KO vs. 20:CypA KO	Two-way ANOVA	0.0179
	0:CypA KO vs. 40:CypA KO	Two-way ANOVA	0.0005
	0:CypA KO vs. 80:CypA KO	Two-way ANOVA	0.0001
	80:WT vs. 80:CypA KO	Two-way ANOVA	>0.9999
5K	DMSO IgG vs. CypA+IgG	Two-way ANOVA	0.1222
	DMSO IgG vs. CypA+Anti-CypA	Two-way ANOVA	0.263
	DMSO CypA+IgG vs. CypA+Anti-CypA	Two-way ANOVA	0.8757
	Vincristine IgG vs. CypA+IgG	Two-way ANOVA	<0.0001
	Vincristine IgG vs. CypA+Anti-CypA	Two-way ANOVA	<0.0001
	Vincristine CypA+IgG vs. CypA+Anti-CypA	Two-way ANOVA	<0.0001
5L	DMSO WT-Ctrl vs. WT-CypA	Two-way ANOVA	0.9998
	DMSOWT-Ctrl vs. MC2R KO-Ctrl	Two-way ANOVA	>0.9999
	DMSO WT-Ctrl vs. MC2R KO-CypA	Two-way ANOVA	0.9995
	DMSO WT-CypA vs. MC2R KO-Ctrl	Two-way ANOVA	0.9999
	DMSO WT-CypA vs. MC2R KO-CypA	Two-way ANOVA	>0.9999
	DMSO MC2R KO-Ctrl vs. MC2R KO-CypA	Two-way ANOVA	0.9996
	Vincristine WT-Ctrl vs. WT-CypA	Two-way ANOVA	<0.0001
	Vincristine WT-Ctrl vs. MC2R KO-Ctrl	Two-way ANOVA	<0.0001
	Vincristine WT-Ctrl vs. MC2R KO-CypA	Two-way ANOVA	<0.0001
	Vincristine WT-CypA vs. MC2R KO-Ctrl	Two-way ANOVA	<0.0001
	Vincristine WT-CypA vs. MC2R KO-CypA	Two-way ANOVA	<0.0001
	Vincristine MC2R KO-Ctrl vs. MC2R KO-CypA	Two-way ANOVA	0.9829
5M	DMSO WT-Ctrl vs. WT-CypA	Two-way ANOVA	0.414
	DMSO WT-Ctrl vs. CypA KO-Ctrl	Two-way ANOVA	<0.0001
	DMSO WT-Ctrl vs. CypA KO-CypA	Two-way ANOVA	<0.0001
	DMSO WT-CypA vs. CypA KO-Ctrl	Two-way ANOVA	<0.0001
	DMSO WT-CypA vs. CypA KO-CypA	Two-way ANOVA	<0.0001
	DMSO CypA KO-Ctrl vs. CypA KO-CypA	Two-way ANOVA	0.9814
	Vincristine WT-Ctrl vs. WT-CypA	Two-way ANOVA	<0.0001
	Vincristine WT-Ctrl vs. CypA KO-Ctrl	Two-way ANOVA	<0.0001
	Vincristine WT-Ctrl vs. CypA KO-CypA	Two-way ANOVA	<0.0001
	Vincristine WT-CypA vs. CypA KO-Ctrl	Two-way ANOVA	<0.0001
	Vincristine WT-CypA vs. CypA KO-CypA	Two-way ANOVA	<0.0001
	Vincristine CypA KO-Ctrl vs. CypA KO-CypA	Two-way ANOVA	<0.0001
6A	Treated vs.Untreated	Unpaired two-tailed Student’s t-test	0.0021
6B	Treated vs.Untreated	Unpaired two-tailed Student’s t-test	0.9206
6D	IgG vs. Ctrl	One-way ANOVA	<0.0001

	IgG vs. Anti-CypA	One-way ANOVA	<0.0001
	IgG vs. VDL+IgG	One-way ANOVA	0.0001
	IgG vs. VDL+Anti-CypA	One-way ANOVA	<0.0001
	VDL+IgG vs. VDL+Anti-CypA	One-way ANOVA	0.0885
6E	IgG vs. Ctrl	One-way ANOVA	0.0216
	IgG vs. Anti-CypA	One-way ANOVA	0.002
	IgG vs. VDL+IgG	One-way ANOVA	0.1651
	IgG vs. VDL+Anti-CypA	One-way ANOVA	0.0011
	VDL+IgG vs. VDL+Anti-CypA	One-way ANOVA	0.0328
6F	Day20 IgG vs. Anti-CypA	Student's t-test	0.0383
	Day30 VDL+IgG vs. VDL+Anti-CypA	Student's t-test	0.0006
6G	IgG vs. Anti-CypA	One-way ANOVA	0.0003
	IgG vs. VDL+IgG	One-way ANOVA	<0.0001
	IgG vs. VDL+Anti-CypA	One-way ANOVA	<0.0001
	VDL+IgG vs. VDL+Anti-CypA	One-way ANOVA	0.0302
6H	IgG VS Anti-CypA	Log-rank (Mantel-Cox) test	0.0002
	IgG VS VDL	Log-rank (Mantel-Cox) test	<0.0001
	VDL VS VDL+Anti-CypA	Log-rank (Mantel-Cox) test	0.0003
	IgG VS VDL+Anti-CypA	Log-rank (Mantel-Cox) test	<0.0001
7B	2	Two-way ANOVA	>0.9999
	3	Two-way ANOVA	0.9988
	4	Two-way ANOVA	0.723
	5	Two-way ANOVA	<0.0001
7C	2	Two-way ANOVA	>0.9999
	3	Two-way ANOVA	0.9987
	4	Two-way ANOVA	0.4585
	5	Two-way ANOVA	0.0034
7D	BM	Two-way ANOVA	0.0139
	SP	Two-way ANOVA	0.0113
7E	BM	Two-way ANOVA	<0.0001
	SP	Two-way ANOVA	0.0331
7F	IgG VS Anti-CypA	Log-rank (Mantel-Cox) test	0.0048
7G	IgG VS Anti-CypA	Log-rank (Mantel-Cox) test	0.0025
EV1A	WBCs vs. REH-CM+WBCs	One-way ANOVA	0.0007
EV1B	PB-WBCs vs. Indirect	One-way ANOVA	0.0079
	PB-WBCs vs. Direct	One-way ANOVA	<0.0001
EV1C	PBMCs vs. Indirect	One-way ANOVA	0.0215
	PBMCs vs. Direct	One-way ANOVA	0.0047
EV1D	PB-GCs vs. Indirect	One-way ANOVA	<0.0001
	PB-GCs vs. Direct	One-way ANOVA	<0.0001
EV1E	PB-WBCs vs. Indirect	One-way ANOVA	0.001
	PB-WBCs vs. Direct	One-way ANOVA	<0.0001
EV1F	PBMCs vs. Indirect	One-way ANOVA	0.0275
	PBMCs vs. Direct	One-way ANOVA	0.0034
EV1G	PB-GCs vs. Indirect	One-way ANOVA	0.0001

	PB-GCs vs. Direct	One-way ANOVA	<0.0001
EV2A	Spleen (cm2) IgG vs. Ctrl	Student’s t-test	0.0002
	Spleen (cm2) IgG vs. CypA+IgG	Student’s t-test	0.0268
	Spleen (cm2) IgG vs. Anti-CypA	Student’s t-test	0.0497
	Spleen (g) IgG vs. Ctrl	Student’s t-test	0.0001
	Spleen (g) IgG vs. CypA+IgG	Student’s t-test	0.002
	Spleen (g) IgG vs. Anti-CypA	Student’s t-test	0.0063
EV2B	IgG vs. CypA	One-way ANOVA	<0.0001
	IgG vs. Anti-CypA	One-way ANOVA	0.0188
EV2D	IgG vs. Anti-CypA	Log-rank (Mantel-Cox) test	0.0089
EV3D	GFP-MC2R NT	Two-way ANOVA	<0.0001
	GFP-MC2R EC3	Two-way ANOVA	<0.0001
EV3G	HD vs. ALL	Student’s t-test	0.0426
EV3H	eCypA level vs MC2R expression evel	Linear correlation analysis	0.021
EV3I	NALM6 0 vs. 5	Two-way ANOVA	<0.0001
	NALM6 0 vs. 10	Two-way ANOVA	<0.0001
	NALM6 0 vs. 30	Two-way ANOVA	0.9833
	NALM6 0 vs. 60	Two-way ANOVA	0.1266
	RS4;11 0 vs. 5	Two-way ANOVA	0.9763
	RS4;11 0 vs. 10	Two-way ANOVA	0.0006
	RS4;11 0 vs. 30	Two-way ANOVA	<0.0001
	RS4;11 0 vs. 60	Two-way ANOVA	0.4454
	Reh 0 vs. 5	Two-way ANOVA	0.4126
	Reh 0 vs. 10	Two-way ANOVA	<0.0001
	Reh 0 vs. 30	Two-way ANOVA	<0.0001
	Reh 0 vs. 60	Two-way ANOVA	0.0002
EV3J	WT	Two-way ANOVA	<0.0001
	CypA KO	Two-way ANOVA	<0.0001
EV3M	Control 0 vs. 5	Two-way ANOVA	0.0009
	Control 0 vs. 10	Two-way ANOVA	<0.0001
	MC2R 0 vs. 5	Two-way ANOVA	<0.0001
	MC2R 0 vs. 10	Two-way ANOVA	<0.0001
	10:Control vs. 10:MC2R	Two-way ANOVA	<0.0001
EV5A	RS4;11 FN1		
	IgG vs. CypA+IgG	Two-way ANOVA	<0.0001
	IgG vs. CypA+Anti-CypA	Two-way ANOVA	0.4755
	CypA+IgG vs. CypA+Anti-CypA	Two-way ANOVA	<0.0001
	RS4;11 CD44		
	IgG vs. CypA+IgG	Two-way ANOVA	<0.0001
	IgG vs. CypA+Anti-CypA	Two-way ANOVA	0.9989
	CypA+IgG vs. CypA+Anti-CypA	Two-way ANOVA	<0.0001
	RS4;11 MMP9		
	IgG vs. CypA+IgG	Two-way ANOVA	<0.0001
	IgG vs. CypA+Anti-CypA	Two-way ANOVA	0.6112
	CypA+IgG vs. CypA+Anti-CypA	Two-way ANOVA	<0.0001

	REH FN1		
	IgG vs. CypA+IgG	Two-way ANOVA	<0.0001
	IgG vs. CypA+Anti-CypA	Two-way ANOVA	0.7433
	CypA+IgG vs. CypA+Anti-CypA	Two-way ANOVA	<0.0001
	REH CD44		
	IgG vs. CypA+IgG	Two-way ANOVA	<0.0001
	IgG vs. CypA+Anti-CypA	Two-way ANOVA	0.0049
	CypA+IgG vs. CypA+Anti-CypA	Two-way ANOVA	<0.0001
	REH MMP9		
	IgG vs. CypA+IgG	Two-way ANOVA	<0.0001
	IgG vs. CypA+Anti-CypA	Two-way ANOVA	0.535
	CypA+IgG vs. CypA+Anti-CypA	Two-way ANOVA	<0.0001
EV5B	FN1		
	DMSO vs. CypA	Two-way ANOVA	<0.0001
	CREB inhibitor vs. CREB inhibitor + CypA	Two-way ANOVA	0.9993
	PKA inhibitor vs. PKA inhibitor + CypA	Two-way ANOVA	0.9397
	CD44		
	DMSO vs. CypA	Two-way ANOVA	<0.0001
	CREB inhibitor vs. CREB inhibitor + CypA	Two-way ANOVA	0.7681
	PKA inhibitor vs. PKA inhibitor + CypA	Two-way ANOVA	0.6753
	MMP9		
	DMSO vs. CypA	Two-way ANOVA	<0.0001
	CREB inhibitor vs. CREB inhibitor + CypA	Two-way ANOVA	>0.9999
	PKA inhibitor vs. PKA inhibitor + CypA	Two-way ANOVA	0.9
EV5C	adhesion cell count		
	IgG+CypA vs. IgG	One-way ANOVA	<0.0001
	IgG+CypA vs. CypA+Anti-CypA	One-way ANOVA	<0.0001
	Trans-endothelial cell count		
	IgG+CypA vs. IgG	One-way ANOVA	0.0079
	IgG+CypA vs. CypA+Anti-CypA	One-way ANOVA	0.0151
EV5D	adhesion cell count		
	IgG+CypA vs. IgG	One-way ANOVA	0.0096
	IgG+CypA vs. CypA+Anti-CypA	One-way ANOVA	0.0038
	Trans-endothelial cell count		
	IgG+CypA vs. IgG	One-way ANOVA	0.0077
	IgG+CypA vs. CypA+Anti-CypA	One-way ANOVA	0.0065
EV5E	CD44	Student’s t-test	0.0091
	MMP9	Student’s t-test	0.0499
	FN1	Student’s t-test	0.0187
EV5F	eCypA vs. MMP9	Linear correlation analysis	0.0368
	eCypA vs. CD44	Linear correlation analysis	0.0669
	eCypA vs. FN1	Linear correlation analysis	0.0198
EV5G	MC2R vs. MMP9	Linear correlation analysis	0.0504
	MC2R vs. CD44	Linear correlation analysis	0.0438
	MC2R vs. FN1	Linear correlation analysis	0.0272

EV6A	RS4;11 Bcl-xl		
	IgG vs. CypA+IgG	Two-way ANOVA	<0.0001
	IgG vs. CypA+Anti-CypA	Two-way ANOVA	0.0061
	CypA+IgG vs. CypA+Anti-CypA	Two-way ANOVA	<0.0001
	RS4;11 MCL1		
	IgG vs. CypA+IgG	Two-way ANOVA	<0.0001
	IgG vs. CypA+Anti-CypA	Two-way ANOVA	>0.9999
	CypA+IgG vs. CypA+Anti-CypA	Two-way ANOVA	<0.0001
	REH Bcl-xl		
	IgG vs. CypA+IgG	Two-way ANOVA	<0.0001
	IgG vs. CypA+Anti-CypA	Two-way ANOVA	0.4018
	CypA+IgG vs. CypA+Anti-CypA	Two-way ANOVA	<0.0001
	REH MCL1		
	IgG vs. CypA+IgG	Two-way ANOVA	<0.0001
	IgG vs. CypA+Anti-CypA	Two-way ANOVA	0.4018
	CypA+IgG vs. CypA+Anti-CypA	Two-way ANOVA	<0.0001
EV6B	Bcl-xl		
	DMSO vs. CypA	Two-way ANOVA	<0.0001
	CREB inhibitor vs. CREB inhibitor + CypA	Two-way ANOVA	0.3863
	PKA inhibitor vs. PKA inhibitor + CypA	Two-way ANOVA	0.0003
	MCL1		
	DMSO vs. CypA	Two-way ANOVA	<0.0001
	CREB inhibitor vs. CREB inhibitor + CypA	Two-way ANOVA	0.9336
	PKA inhibitor vs. PKA inhibitor + CypA	Two-way ANOVA	0.9996
EV6C	0:CypA vs. 5:CypA	Two-way ANOVA	0.433
	0:CypA vs. 10:CypA	Two-way ANOVA	0.0003
	0:CypA vs. 20:CypA	Two-way ANOVA	<0.0001
	0:CypA vs. 40:CypA	Two-way ANOVA	<0.0001
	0:CypA vs. 80:CypA	Two-way ANOVA	<0.0001
	0:Anti-CypA vs. 5:Anti-CypA	Two-way ANOVA	>0.9999
	0:Anti-CypA vs. 10:Anti-CypA	Two-way ANOVA	>0.9999
	0:Anti-CypA vs. 20:Anti-CypA	Two-way ANOVA	0.9724
	0:Anti-CypA vs. 40:Anti-CypA	Two-way ANOVA	0.7347
	0:Anti-CypA vs. 80:Anti-CypA	Two-way ANOVA	>0.9999
	0:CypA+Anti-CypA vs. 5:CypA+Anti-CypA	Two-way ANOVA	0.9998
	0:CypA+Anti-CypA vs. 10:CypA+Anti-CypA	Two-way ANOVA	>0.9999
	0:CypA+Anti-CypA vs. 20:CypA+Anti-CypA	Two-way ANOVA	0.9751
	0:CypA+Anti-CypA vs. 40:CypA+Anti-CypA	Two-way ANOVA	0.9821
	0:CypA+Anti-CypA vs. 80:CypA+Anti-CypA	Two-way ANOVA	0.603
	80:CypA vs. 80:Anti-CypA	Two-way ANOVA	<0.0001
	80:CypA vs. 80:CypA+Anti-CypA	Two-way ANOVA	<0.0001
	80:Anti-CypA vs. 80:CypA+Anti-CypA	Two-way ANOVA	0.9889
EV6D	0:CypA vs. 5:CypA	Two-way ANOVA	0.5917
	0:CypA vs. 10:CypA	Two-way ANOVA	0.0357
	0:CypA vs. 20:CypA	Two-way ANOVA	<0.0001

	0:CypA vs. 40:CypA	Two-way ANOVA	<0.0001
	0:CypA vs. 80:CypA	Two-way ANOVA	<0.0001
	0:Anti-CypA vs. 5:Anti-CypA	Two-way ANOVA	>0.9999
	0:Anti-CypA vs. 10:Anti-CypA	Two-way ANOVA	>0.9999
	0:Anti-CypA vs. 20:Anti-CypA	Two-way ANOVA	>0.9999
	0:Anti-CypA vs. 40:Anti-CypA	Two-way ANOVA	>0.9999
	0:Anti-CypA vs. 80:Anti-CypA	Two-way ANOVA	0.9748
	0:CypA+Anti-CypA vs. 5:CypA+Anti-CypA	Two-way ANOVA	>0.9999
	0:CypA+Anti-CypA vs. 10:CypA+Anti-CypA	Two-way ANOVA	>0.9999
	0:CypA+Anti-CypA vs. 20:CypA+Anti-CypA	Two-way ANOVA	>0.9999
	0:CypA+Anti-CypA vs. 40:CypA+Anti-CypA	Two-way ANOVA	>0.9999
	0:CypA+Anti-CypA vs. 80:CypA+Anti-CypA	Two-way ANOVA	>0.9999
	80:CypA vs. 80:Anti-CypA	Two-way ANOVA	<0.0001
	80:CypA vs. 80:CypA+Anti-CypA	Two-way ANOVA	<0.0001
	80:Anti-CypA vs. 80:CypA+Anti-CypA	Two-way ANOVA	>0.9999
EV6E	DMSO		
	IgG vs. CypA+IgG	Two-way ANOVA	0.1353
	IgG vs. CypA+Anti-CypA	Two-way ANOVA	0.6098
	CypA vs. CypA+Anti-CypA	Two-way ANOVA	0.677
	Vincristine		
	IgG vs. CypA+IgG	Two-way ANOVA	<0.0001
	IgG vs. CypA+Anti-CypA	Two-way ANOVA	0.0134
	CypA vs. CypA+Anti-CypA	Two-way ANOVA	<0.0001
EV6F	DMSO		
	IgG vs. CypA+IgG	Two-way ANOVA	0.9978
	IgG vs. CypA+Anti-CypA	Two-way ANOVA	0.9479
	CypA vs. CypA+Anti-CypA	Two-way ANOVA	0.9837
	Vincristine		
	IgG vs. CypA+IgG	Two-way ANOVA	<0.0001
	IgG vs. CypA+Anti-CypA	Two-way ANOVA	<0.0001
	CypA vs. CypA+Anti-CypA	Two-way ANOVA	<0.0001
EV6G	Bcl-xl	Student's t-test	0.0194
	MCL1	Student's t-test	0.1354
EV6H	eCypA vs. Bcl-xl	Linear correlation analysis	0.0288
	eCypA vs. MCL1	Linear correlation analysis	0.7302
EV6I	MC2R vs. Bcl-xl	Linear correlation analysis	0.0039
	MC2R vs. MCL1	Linear correlation analysis	0.439
