# Supplementary material for: Extracellular cyclophilin A promotes B-cell acute lymphoblastic leukemia progression via MC2R
Source: EMBO Mol Med. 2026 Jun 10;18(8):3028–56. doi: 10.1038/s44321-026-00466-w (PMC13469882; doi:10.1038/s44321-026-00466-w)
Supplement: Supplementary file 2 [file 44321_2026_466_MOESM23_ESM.pdf]

Expanded View Figures

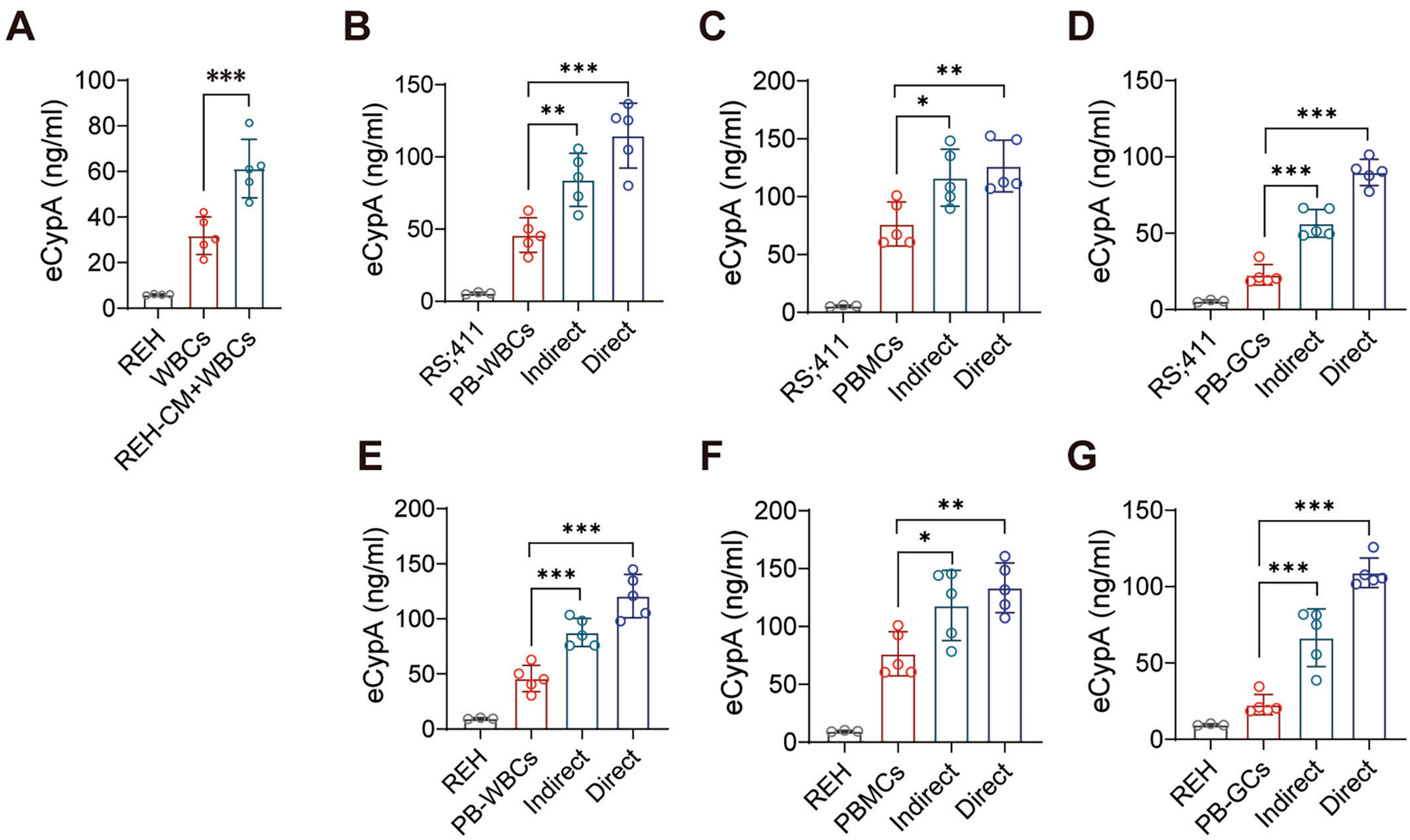

Figure EV1. ALL cells stimulate eCypA secretion from peripheral blood-derived cells.

(**A**) ELISA analysis of eCypA levels in the supernatant of WBCs cultured alone, REH cells ($n = 4$), or WBCs treated with REH-conditioned medium (REH-CM) ($n = 5$). (**B–D**) ELISA analysis of eCypA levels in the supernatant from indirect and direct co-cultures of RS4;11 cells with peripheral blood WBCs (PB-WBCs) (**B**), peripheral blood mononuclear cells (PBMCs) (**C**), and peripheral blood granulocytes (PB-GCs) (**D**) ($n = 5$). RS4;11-only control groups were analyzed using matched independent wells ($n = 3$). (**E–G**) ELISA analysis of eCypA levels in the supernatant from indirect and direct co-cultures of REH cells with PB-WBCs (**E**), PBMCs (**F**), and PB-GCs (**G**) ($n = 5$). REH-only control groups were analyzed using matched independent wells ($n = 3$). Data were presented as mean ± SD; in co-culture and conditioned medium groups, *n* represents independent donor samples. In cell line-only control groups, *n* represents independent wells. Statistical significance was determined using one-way ANOVA. $^{*}p < 0.05$, $^{**}p < 0.01$, $^{***}p < 0.001$; ns not significant. Source data are available online for this figure.

 © The Author(s)

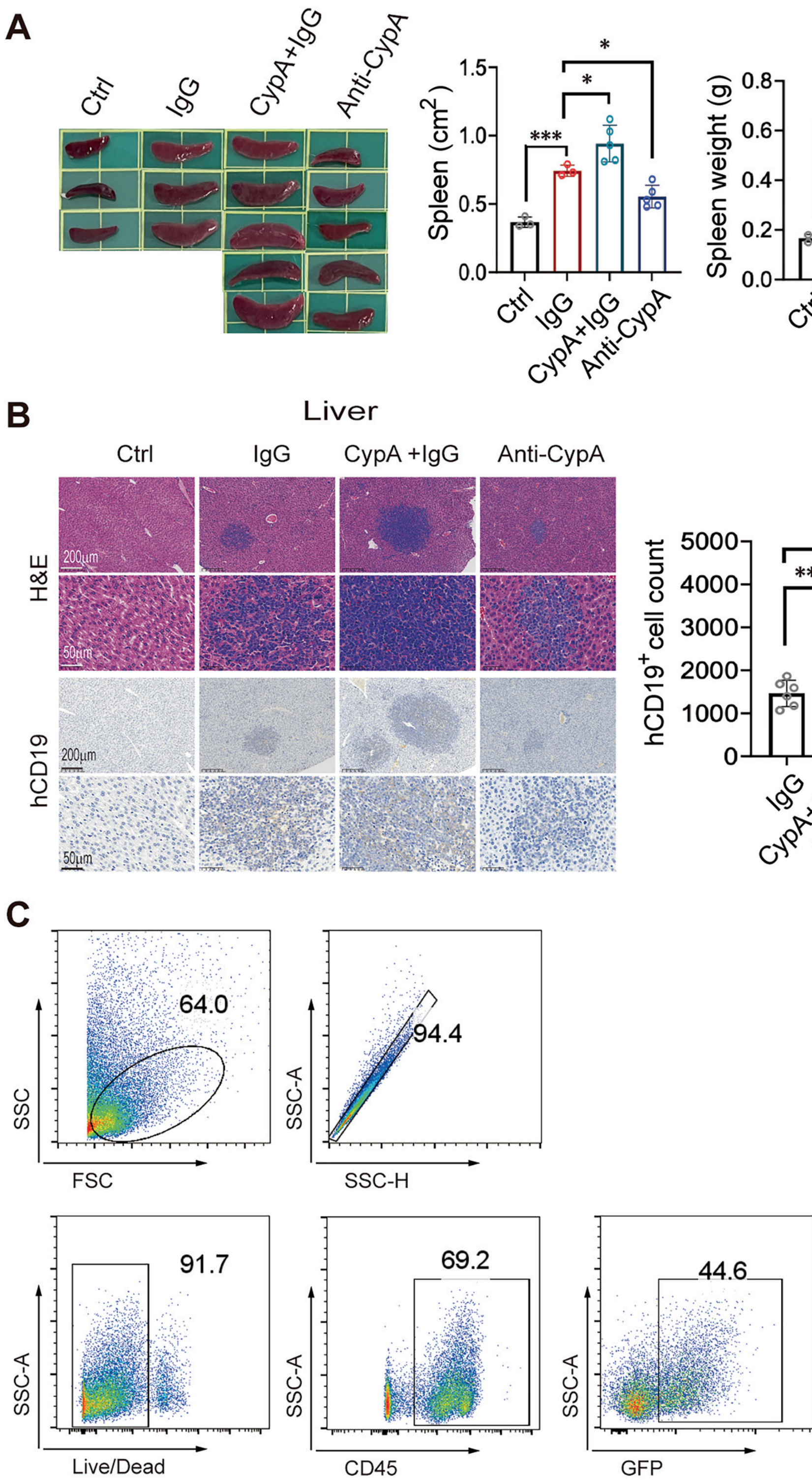
A
Ctrl
IgG
CypA+IgG
Anti-CypA
Spleen (cm^2)
Spleen weight (g)
***
*
**
B
Liver
Ctrl
IgG
CypA +IgG
Anti-CypA
H&E
hCD19
200μm
50μm
hCD19$^+$cell count
C
64.0
94.4
91.7
69.2
44.6
SSC
FSC
SSC-A
SSC-H
Live/Dead
CD45
GFP
D
Survival probability
IgG
Anti-CypA
p=0.0089
Day

Figure EV2. eCypA exacerbates splenomegaly and promotes leukemic infiltration in vivo.

(**A**) Representative images of spleens from mice on day 22 post-ALL injection (left), with quantification of spleen area using ImageJ (middle) and spleen weight (right). Groups included Ctrl ($n = 3$), IgG ($n = 3$), CypA ($n = 5$), and Anti-CypA ($n = 5$). (**B**) H&E staining and hCD19 immunohistochemistry of mouse liver sections (left), with quantitative analysis of hCD19$^+$ areas using ImageJ (right). Scale bars, 200 and 50 µm as indicated. Each data point represents one microscopic field, with two randomly selected fields analyzed per mouse (IgG, $n = 6$; CypA+IgG, $n = 10$; Anti-CypA, $n = 10$). (**C**) Flow cytometry gating strategy for the p190-BCR-ABL mouse model. Cells were sequentially gated on FSC/SSC to exclude debris, followed by singlet discrimination (SSC-H vs SSC-A), live cells, mCD45$^+$ leukocytes, and finally GFP$^+$ leukemic cells. (**D**) Kaplan–Meier survival analysis of p190-BCR-ABL mice treated with IgG ($n = 6$) or Anti-CypA mAb ($n = 6$). Data were presented as mean ± SD; in (**A**, **D**, *n* represents individual mice. In (**B**), *n* represents independently analyzed microscopic fields. Statistical significance was determined using unpaired two-tailed Student's *t*-test for (**A**), one-way ANOVA for (**B**), and log-rank (Mantel–Cox) test for (**D**). $^*p < 0.05$, $^{**}p < 0.01$, $^{***}p < 0.001$; ns not significant. Source data are available online for this figure.

 © The Author(s)

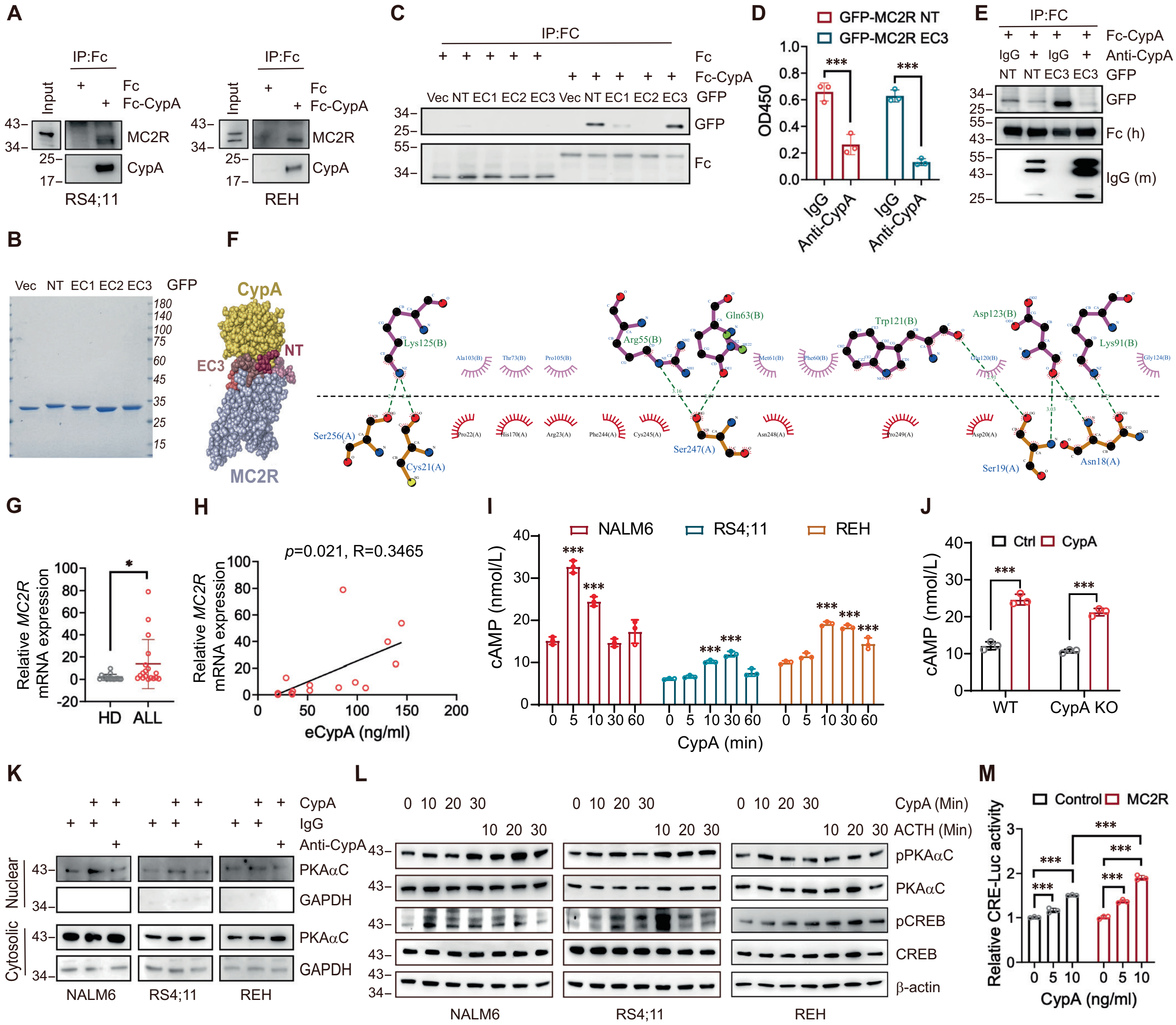

Figure EV3. eCypA activates the cAMP–PKA–CREB signaling pathway.

(**A**) RS4;11 and REH cell lysates were incubated with Fc-CypA, followed by Protein A pull-down and detection of MC2R by western blotting. (**B**) Coomassie brilliant blue staining of eukaryotically purified GFP-tagged proteins. (**C**) Fc or Fc-CypA recombinant protein was incubated with GFP-tagged fragments, followed by Protein A pull-down and western blot detection of GFP and Fc. (**D**) An ELISA-based binding assay in which recombinant CypA was coated and increasing concentrations of GFP-tagged NT and EC3 fragments, with or without Anti-CypA mAb, were added, followed by OD measurement. (**E**) Fc or Fc-CypA recombinant protein was incubated with GFP-tagged fragments in the presence of Anti-CypA mAb or IgG, followed by Protein A pull-down and western blot analysis. (**F**) Molecular docking simulation showing the predicted binding model between CypA and MC2R NT and EC3 domains. (**G**) qPCR analysis of *MC2R* mRNA levels in PBMCs from healthy donors (HD, $n = 15$) and patients with ALL ($n = 18$). (**H**) Correlation analysis between *MC2R* mRNA levels and eCypA levels in patients with ALL ($n = 15$). (**I**) cAMP levels in NALM6, RS4;11, and REH cells treated with CypA for the indicated time points. (**J**) cAMP levels in NALM6 WT and CypA KO cells treated with or without CypA for 10 min. (**K**) NALM6, RS4;11, and REH cells were treated with IgG, CypA + IgG, or CypA+Anti-CypA for 10 min. Nuclear and cytoplasmic fractions were isolated, and PKAα-C levels were detected by western blotting. (**L**) NALM6, RS4;11, and REH cells were treated with 5 ng/mL CypA or 0.8 ng/mL ACTH for the indicated time points, followed by western blotting to assess activation of the PKA–CREB pathway. (**M**) Luciferase reporter assay of CRE activity in 293 T cells transfected with Flag-Vec or Flag-MC2R and treated with increasing concentrations of CypA. Data are presented as mean ± SD; in (**D**, **I**, **J**, **M**), $n = 3$ represents independent wells. In (**G**, **H**), n represents individual donors or patient samples. Western blot and pull-down results in (**A–C**, **E**, **K**, **L**) are representative of at least three independent experiments with similar results. Statistical significance was determined using an unpaired two-tailed Student's *t*-test for (**G**), Pearson correlation analysis for (**H**), and two-way ANOVA for (**D**, **I**, **J**, **M**). $^{*}p < 0.05$, $^{**}p < 0.01$, $^{***}p < 0.001$; ns not significant. Source data are available online for this figure.

© The Author(s)

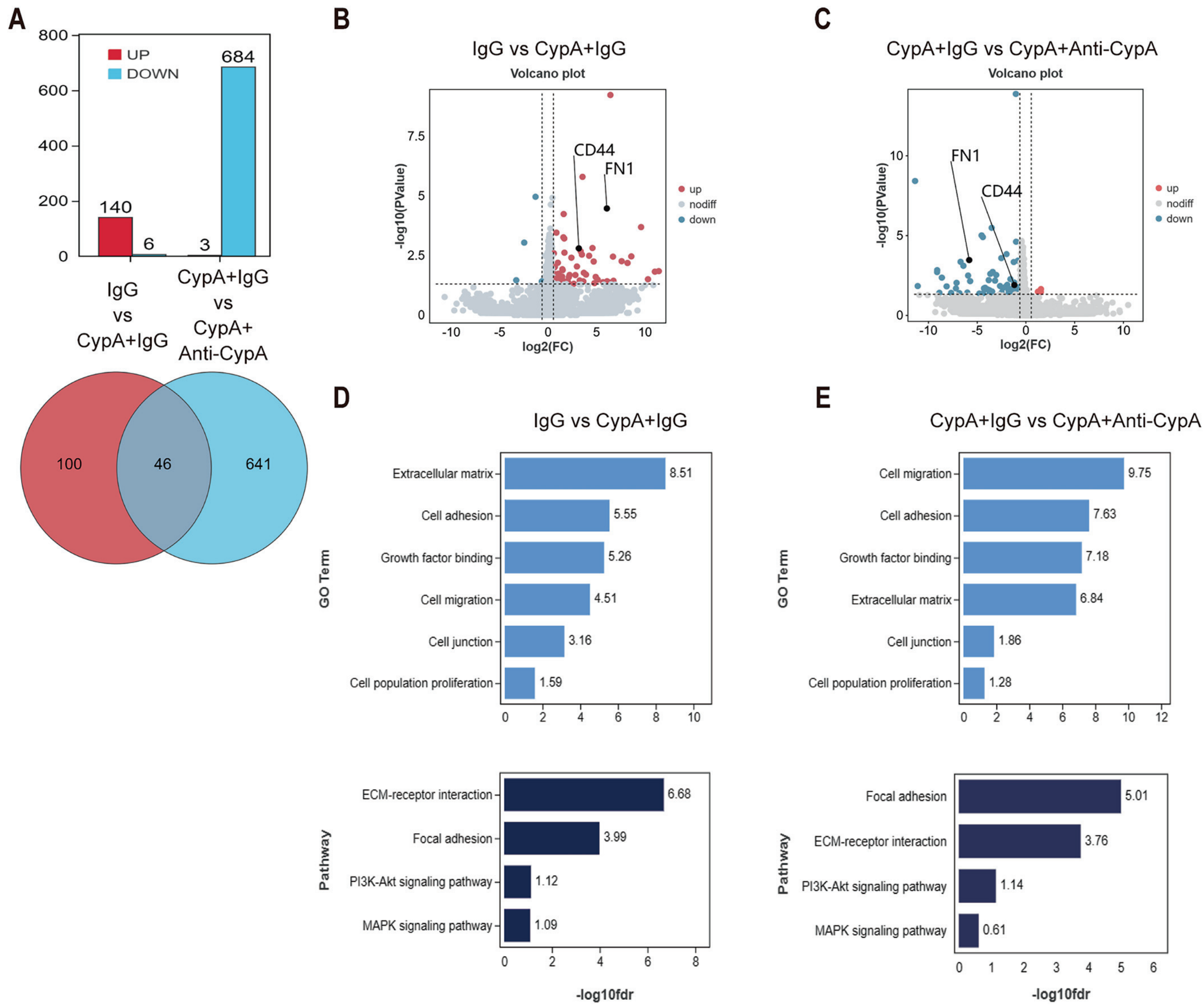

Figure EV4. Transcriptomic analysis of eCypA-regulated genes in NALM6 cells.

NALM6 cells were serum-starved for 6 h and then treated with IgG, CypA + IgG, or CypA + Anti-CypA with three biologically independent samples analyzed per group ($n = 3$ per group). Raw transcriptome reads were deposited in the China National Center for Bioinformation (BioProject accession: PRJCA035020; raw data accession: HRA010096). (**A**) Bar plot showing the numbers of upregulated and downregulated genes; 46 genes were upregulated by CypA and downregulated by Anti-CypA. (**B**, **C**) Volcano plots of differentially expressed genes generated from RNA-seq analysis. Differentially expressed genes were identified using a threshold of $P < 0.05$. (**D**) Gene Ontology (GO) enrichment analysis of differentially expressed genes between IgG and CypA + IgG-treated NALM6 cells. (**E**) KEGG pathway enrichment analysis of differentially expressed genes between CypA + IgG and CypA + anti-CypA-treated NALM6 cells. Source data are available online for this figure.

 © The Author(s)

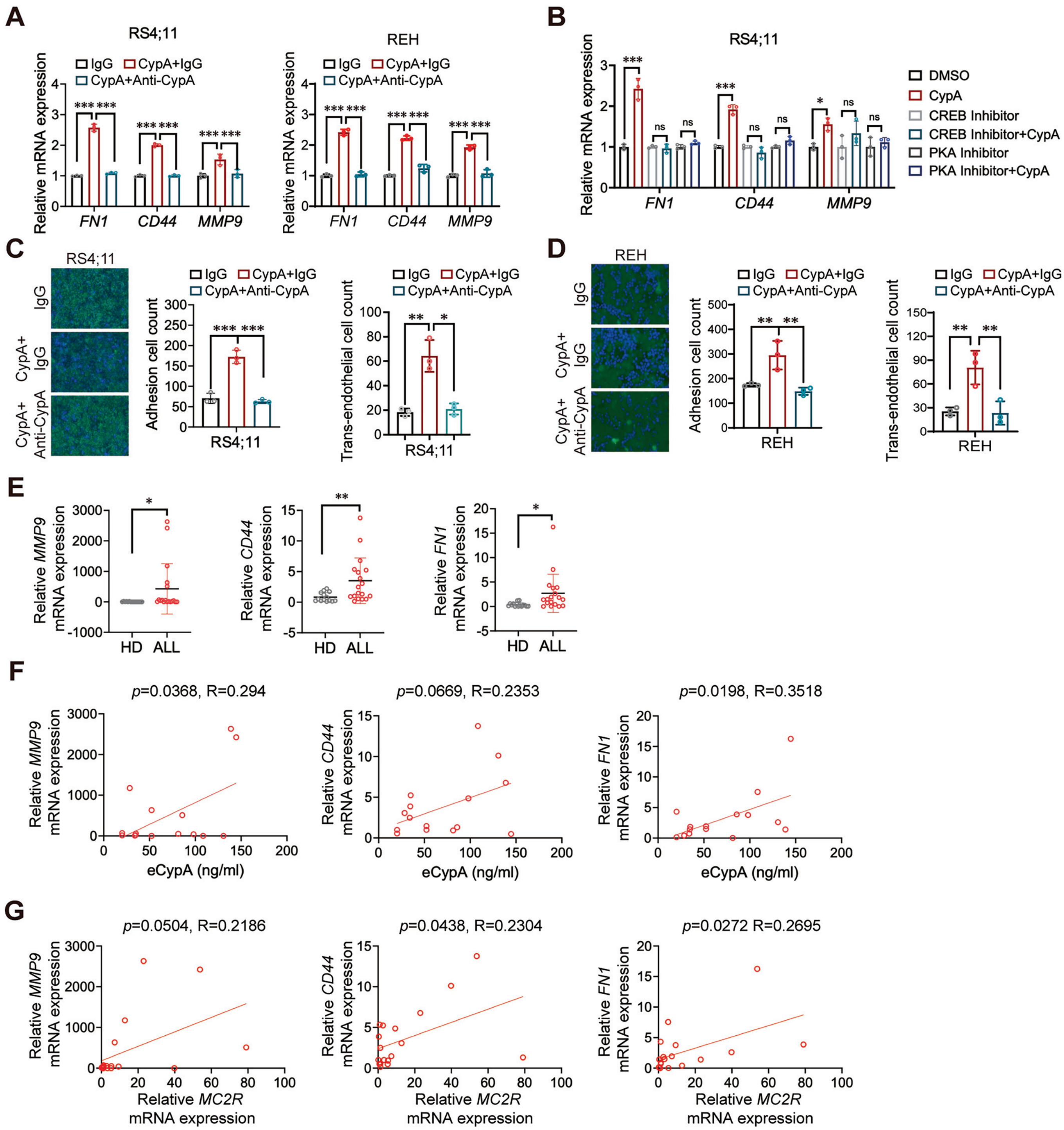

Figure EV5. Effects of eCypA on transendothelial migration of ALL cells.

(**A**) qPCR analysis of *FN1*, *CD44*, and *MMP9* mRNA levels in RS4;11 and REH cells treated with IgG, CypA + IgG, or CypA + anti-CypA for 30 min. (**B**) qPCR analysis of *FN1*, *CD44*, and *MMP9* mRNA levels in RS4;11 cells pretreated with a CREB inhibitor or a PKA inhibitor for 4 h, followed by CypA treatment for 30 min. (**C**, **D**) Transendothelial migration assay: HUVECs were seeded in the upper chamber for 48 h to form an endothelial layer. Hoechst-stained RS4;11 (**C**) and REH (**D**) cells were added and treated with IgG, CypA + IgG, or CypA + anti-CypA. The number of adherent cells was quantified after 4 h, and migrated cells were quantified after 24 h. (**E**) qPCR analysis of *FN1*, *CD44*, and *MMP9* mRNA levels in PBMCs from healthy donors (HD, $n = 15$) and patients with ALL ($n = 18$). (**F**) Pearson correlation analysis between *FN1*, *CD44*, and *MMP9* mRNA levels in PBMCs and eCypA levels in PB plasma ($n = 15$). (**G**) Pearson correlation analysis between *FN1*, *CD44*, and *MMP9* mRNA levels and *MC2R* mRNA levels in PBMCs ($n = 18$). Data were presented as mean ± SD; in (**A–D**), $n = 3$ represents independent wells. In (**E–G**), *n* represents individual donors or patient samples. Statistical significance was determined using two-way ANOVA for (**A**, **B**), one-way ANOVA for (**C**, **D**), unpaired two-tailed Student's *t*-test for (**E**), and Pearson correlation analysis for (**F**, **G**). *$p < 0.05$, **$p < 0.01$, ***$p < 0.001$; ns not significant. Source data are available online for this figure.

© The Author(s)

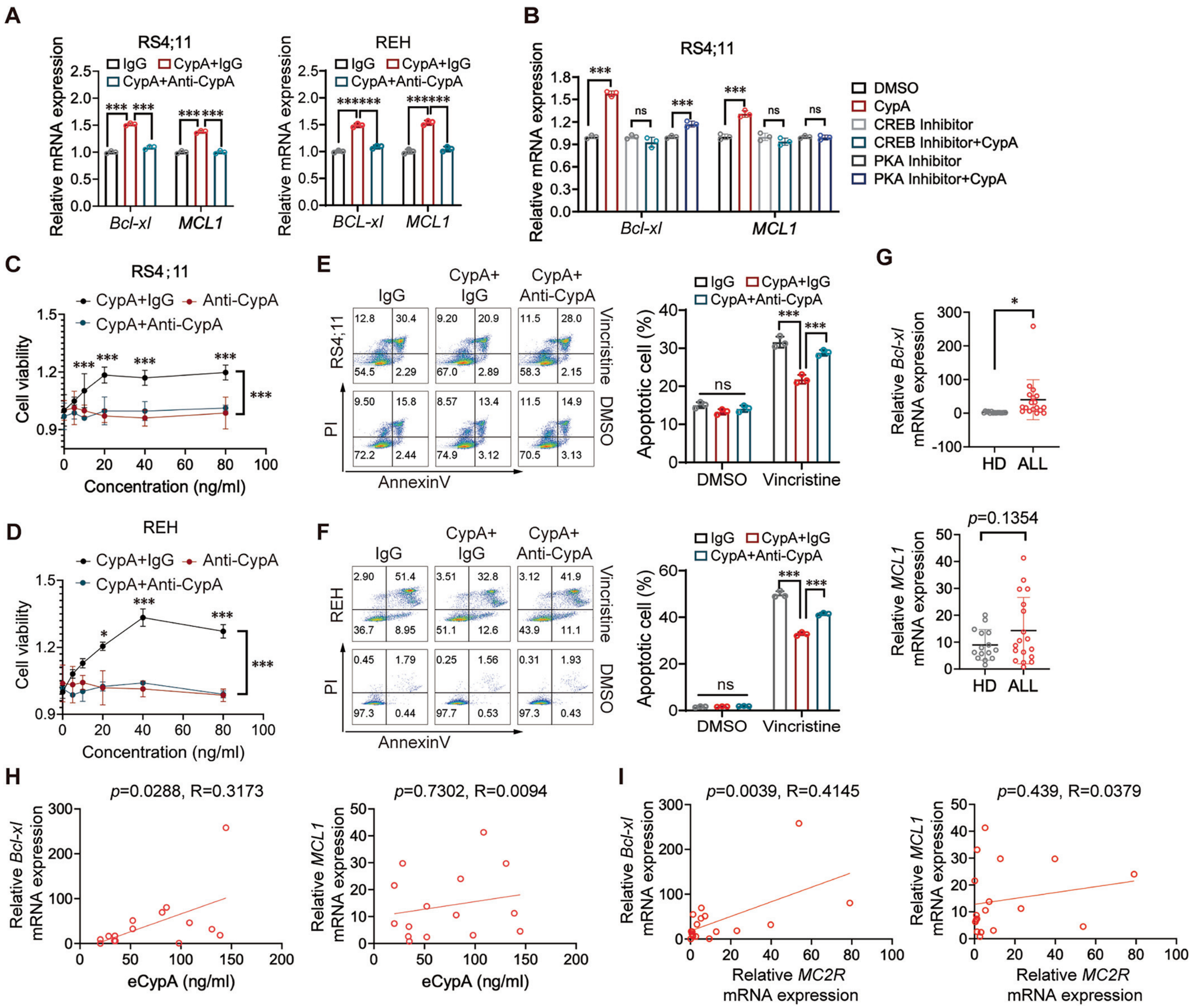

Figure EV6. Anti-apoptotic effects of CypA on ALL cells.

(**A**) qPCR analysis of Bcl-xL (BCL2L1) and MCL1 expression in RS4;11 and REH cells treated with IgG, CypA + IgG, or CypA + anti-CypA for 30 min. (**B**) qPCR analysis of Bcl-xL (BCL2L1) and MCL1 expression in RS4;11 cells pretreated with a CREB inhibitor or a PKA inhibitor for 4 h, followed by CypA treatment for 30 min. (**C**, **D**) Cell viability measured by CCK-8 assay in RS4;11 (**C**) and REH (**D**) cells treated with increasing concentrations of CypA + IgG, anti-CypA, or CypA + anti-CypA for 48 h ($n = 3$). (**E**, **F**) RS4;11 (**E**) and REH (**F**) cells were treated with IgG, CypA + IgG, or CypA + anti-CypA, with or without vincristine, for 36 h, and apoptosis was assessed by Annexin V/PI staining. (**G**) qPCR analysis of *Bcl-xL* and *MCL1* mRNA levels in PBMCs from healthy donors (HD, $n = 15$) and patients with ALL ($n = 18$). (**H**) Pearson correlation analysis between *Bcl-xL* and *MCL1* mRNA levels in PBMCs and eCypA protein levels in PB plasma ($n = 15$). (**I**) Pearson correlation analysis between *Bcl-xL* and *MCL1* mRNA levels and *MC2R* mRNA levels in PBMCs ($n = 18$). Data were presented as mean ± SD; in (**A–F**), $n = 3$ represents independent wells. In (**G–I**), n represents individual donors or patient samples. Statistical significance was determined using two-way ANOVA for (**A–F**), unpaired two-tailed Student's *t*-test for (**G**), and Pearson correlation analysis for (**H**, **I**). *$p < 0.05$, **$p < 0.01$, ***$p < 0.001$; ns not significant. Source data are available online for this figure.

© The Author(s)

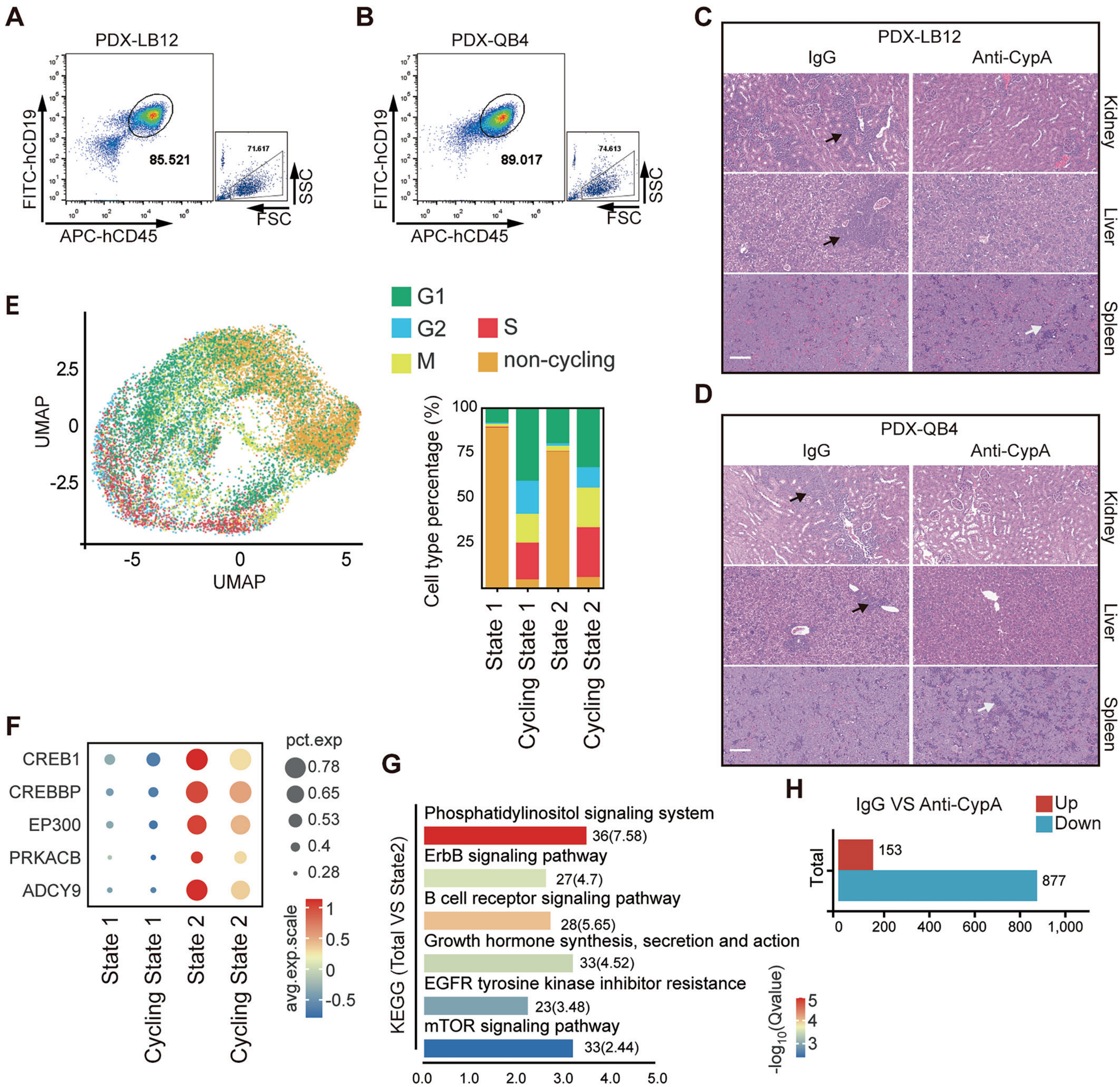

Figure EV7. scRNA-seq analysis reveals CREB pathway activation and transcriptomic changes in a PDX model.

(**A**, **B**) Representative flow cytometry plots showing the proportion of hCD19⁺hCD45⁺ leukemic cells in the spleen of first-generation PDX mice under the indicated conditions. (**C**, **D**) Representative hematoxylin and eosin (H&E) staining of kidney, liver, and spleen tissues from PDX-LB12 (**C**) and PDX-QB4 (**D**) mice on day 38. Black arrows indicate infiltrating B-ALL cells within the tissues, whereas white arrows indicate non–B-ALL cells. Scale bars, 100 µm. (**E**) UCell-based scoring of cell cycle gene signatures, classifying cells into non-cycling, G1, S, G2, and M phases. UMAP visualization of cell cycle states (right) and bar plot showing the distribution of cell cycle phases across different cell clusters (left). (**F**) Bubble plot showing elevated expression of CREB signaling-related genes in cells with multipotent progenitor features. (**G**) KEGG pathway enrichment analysis of genes upregulated in cells with multipotent progenitor features compared with total ALL cells. (**H**) Bar plot showing the number of upregulated and downregulated genes in Anti-CypA-treated versus IgG-treated ALL cells. Representative flow cytometry and histological images in (**A**–**D**) are shown from five independent mice per group with similar results. Source data are available online for this figure.
