# Supplementary material for: Extracellular cyclophilin A promotes B-cell acute lymphoblastic leukemia progression via MC2R
Source: EMBO Mol Med. 2026 Jun 10;18(8):3028–56. doi: 10.1038/s44321-026-00466-w (PMC13469882; doi:10.1038/s44321-026-00466-w)
Supplement: Supplementary file 3 [file 44321_2026_466_MOESM4_ESM.pdf]

EMBO Molecular Medicine

Extracellular cyclophilin A promotes B-Cell acute lymphoblastic leukemia progression via MC2R

Han Zhong Pei, Heqiao Li, Hongman Xue, Wenqing Wang, He Zhang, Zeqiu Fan, Xiaoyuan Bai, Fei Han, Qingmei Li, Yuna Zhao, Lu Yu, Xiaoxiao Jia, Yao Guo, Wenjun Liu, and Lei Sun

Corresponding authors: Lei Sun (sunlei362@im.ac.cn) , Yao Guo (guoy75@mail.sysu.edu.cn), Wenjun Liu (liuwenjun@smart.org.cn)

Review Timeline:

Submission Date:	23rd Oct 25
Editorial Decision:	5th Dec 25
Revision Received:	29th Apr 26
Editorial Decision:	20th May 26
Revision Received:	28th May 26
Accepted:	29th May 26

Editor: Zeljko Durdevic

Transaction Report:

(Note: With the exception of the correction of typographical or spelling errors that could be a source of ambiguity, letters and reports are not edited. Depending on transfer agreements, referee reports obtained elsewhere may or may not be included in this compilation. Referee reports are anonymous unless the Referee chooses to sign their reports.)

5th Dec 2025

Dear Dr. Sun,

Thank you for the submission of your manuscript to EMBO Molecular Medicine. We have now received feedback from the three reviewers who agreed to evaluate your manuscript. All three referees recognize interest of the study but also raise serious and partially overlapping concerns that should be addressed in a major revision. Considering that the revision will require extensive experimentation we think six months rather than three months would be more appropriate to provide the complete revision. If you would like to discuss further the points raised by the referees, I am available to do so via email or video. Let me know if you are interested in this option.

Further consideration of a revision that addresses reviewers' concerns in full will entail a second round of review. EMBO Molecular Medicine encourages a single round of revision only and therefore, acceptance or rejection of the manuscript will depend on the completeness of your responses included in the next, final version of the manuscript. For this reason, and to save you from any frustrations in the end, I would strongly advise against returning an incomplete revision.

We would welcome the submission of a revised version within six months for further consideration. Please let us know if you require longer to complete the revision.

I look forward to receiving your revised manuscript.

Yours sincerely,

Zeljko Durdevic

Zeljko Durdevic
Senior Editor
EMBO Molecular Medicine

*****
When preparing your revised manuscript, please refer to our guidelines: https://link.springer.com/journal/44321/submission-guidelines#cms-Revised-submissions. We perform an initial quality control of all revised manuscripts before re-review; failure to include requested items will delay the evaluation of your revision.

We require:

1) A .docx formatted version of the manuscript text (including legends for main figures, EV figures and tables). Please make sure that the changes are highlighted to be clearly visible.

2) Individual production quality figure files as .eps, .tif, .jpg (one file per figure). For guidance, download the 'Figure Guide PDF': https://media.springernature.com/original/springer-cms/rest/v1/content/27825798/data/v1.

3) A .docx formatted letter INCLUDING the reviewers' reports and your detailed point-by-point responses to their comments. As part of the EMBO Press transparent editorial process, the point-by-point response is part of the Review Process File (RPF), which will be published alongside your paper.

4) A complete author checklist, which you can download from our author guidelines. Please insert information in the checklist that is also reflected in the manuscript. The completed author checklist will also be part of the RPF.

5) Please note that all corresponding authors are required to supply an ORCID ID for their name upon submission of a revised manuscript.

6) It is mandatory to include a 'Data Availability' section after the Materials and Methods. Before submitting your revision, primary

datasets produced in this study need to be deposited in an appropriate public database, and the accession numbers and database listed under 'Data Availability'. Please remember to provide a reviewer password if the datasets are not yet public.

In case you have no data that requires deposition in a public database, please state so in this section. Note that the Data Availability Section is restricted to new primary data that are part of this study.

7) For data quantification: please specify the name of the statistical test used to generate error bars and P values, the number (n) of independent experiments (specify technical or biological replicates) underlying each data point and the test used to calculate p-values in each figure legend. The figure legends should contain a basic description of n, P and the test applied. Graphs must include a description of the bars and the error bars (s.d., s.e.m.).

8) At EMBO Press we ask authors to provide source data for the main manuscript figures. You will receive a separate email with instructions for providing source data with your revised manuscript, including how to upload and organize the files.

9) Our journal encourages inclusion of *data citations in the reference list* to directly cite datasets that were re-used and obtained from public databases. Data citations in the article text are distinct from normal bibliographical citations and should directly link to the database records from which the data can be accessed. In the main text, data citations are formatted as follows: "Data ref: Smith et al, 2001" or "Data ref: NCBI Sequence Read Archive PRJNA342805, 2017". In the Reference list, data citations must be labeled with "[DATASET]". A data reference must provide the database name, accession number/identifiers and a resolvable link to the landing page from which the data can be accessed at the end of the reference.

10) We replaced Supplementary Information with Expanded View (EV) Figures and Tables that are collapsible/expandable online. A maximum of 5 EV Figures can be typeset. EV Figures should be cited as 'Figure EV1, Figure EV2" etc... in the text and their respective legends should be included in the main text after the legends of regular figures.

- For the figures that you do NOT wish to display as Expanded View figures, they should be bundled together with their legends in a single PDF file called *Appendix*, which should start with a short Table of Content. Appendix figures should be referred to in the main text as: "Appendix Figure S1, Appendix Figure S2" etc.

- Additional Tables/Datasets should be labeled and referred to as Table EV1, Dataset EV1, etc. Legends have to be provided in a separate tab in case of .xls files. Alternatively, the legend can be supplied as a separate text file (README) and zipped together with the Table/Dataset file.

11) The paper explained: EMBO Molecular Medicine articles are accompanied by a summary of the articles to emphasize the major findings in the paper and their medical implications for the non-specialist reader. Please provide a draft summary of your article highlighting

- the medical issue you are addressing,

- the results obtained and

- their clinical impact.

This may be edited to ensure that readers understand the significance and context of the research. Please refer to any of our published articles for an example.

12) Author contributions: You will be asked to provide CRediT (Contributor Role Taxonomy) terms in the submission system. These replace a narrative author contribution section in the manuscript.

13) A Conflict of Interest statement should be provided in the main text.

14) Every published paper includes a 'Synopsis' to further enhance discoverability. Synopses are displayed on the journal webpage and are freely accessible to all readers. They include a short stand first (maximum of 300 characters, including space) as well as 2-5 one-sentences bullet points that summarizes the paper. Please write the bullet points to summarize the key NEW findings. They should be designed to be complementary to the abstract - i.e. not repeat the same text. We encourage inclusion of key acronyms and quantitative information (maximum of 30 words / bullet point). Please use the passive voice. Please attach these in a separate file or send them by email, we will incorporate them accordingly.

Please also suggest a striking image or visual abstract to illustrate your article as a PNG file 550 px wide x 300-600 px high.

15) Include a Reagents and Tools Table as part of the Methods section, which can be downloaded from our author guidelines.

EMBO Molecular Medicine has a "scooping protection" policy, whereby similar findings that are published by others during

review or revision are not a criterion for rejection. Should you decide to submit a revised version, I do ask that you get in touch after three months if you have not completed it, to update us on the status.

***** Reviewer's comments *****

Referee #1 (Comments on Novelty/Model System for Author):

Overall the manuscript and experimental systems used are good.
I would suggest if possible using also a primary model of ALL, so that recipient mice are immunocompetent, to see if the therapeutic effect holds (or is increased/decreased) in the presence of the host's immune system

Referee #1 (Remarks for Author):

In this article, Pei et al show that extracellular cyclophylin A (eCypA) is upregulated in ALL in autocrine and paracrine ways, and eCypA accelerates ALL progression in vivo. This is due to eCypA binding melanocortin 2 receptor and its consequent activation of AMOK-CREB, which up regulates CD44/FN1/MMP9 to promote migration and prevent apoptosis. In line with this, in vivo treatment with neutralizing antibodies against CypA slowed disease progression in PDXs. Overall, this is an interesting manuscript. I have some minor comments for authors to address:
1. Authors make reference to ALL throughout the manuscript, but all the models used are B-ALL, so I would ask them to clarify this. Is eCypA only relevant in B-ALL? Or also T-ALL? If only B-ALL, they should specifically mention B-ALL in the abstract and elsewhere.
2. In Fig 2b and 2c authors measure mouse and human CypA. Are authors completely sure that these assays aren't cross reactive? This should be mentioned and/or discussed.
3. The binding showed in Fig3 uses 293T cells. Authors should show this in ALL cells (ideally endogenously or, if antibodies aren't good for IP, upon expressing of tagged-versions).
4. Authors use a PDX in Fig 6, but don't mention anything regarding its mutations or clinical subgroup (although the reader can infer it is MLL-rearranged from the scRNAseq data). This information should be added.
5. All the in vivo assays are done in immunodeficient mice, as they involve either human cell lines or PDXs. It would be interesting to see the effects in the context of mouse primary ALL, where hosts do have their competent immune system. Would the effects of anti-eCypA be the same, increased, decreased?

Referee #2 (Comments on Novelty/Model System for Author):

1. Technical quality (including statistical analysis). Materials and methods are inadequately explained.
Strong evidence for the conclusions that are drawn. The evidence for some of the conclusions is relatively strong. Other conclusions are less well-supported, among others because only one cell line (NALM6) or in some cases two cell lines (including RS4;11) are used. In the in vivo treatment with antibodies against eCypA, differences in survival with controls are modest in all three experiments presented [one with a cell line, and two with patient samples]. Moreover, although the investigators do use antibodies against eCypA and knock out MC2R in the ALL cells, they do not include experiments in which knock out of the prolyl isomerase A gene [PPIA] encoding CypA in the ALL cells was done. Mice deficient for Ppia are viable [for example PMID: 26932182]. Seeing that they indicate that CypA is produced endogenously in the ALL cells it is difficult to understand why no knockout or knockdown experiments were done to support the importance of CypA to ALL survival.
2. Novelty. The finding of a signaling pathway from extracellular CypA via the MC2R in ALL has not been reported.
3. Medical impact. Results could be important and point to a possible new treatment target. The most important finding, which will have to be better supported, is that the levels of eCypA in PB could be used to assess whether a patient is minimal residual disease-positive after 14 days of chemotherapy.
4. Adequacy of model system. The investigators use standard techniques, which are adequate to investigate the importance of eCypA in ALL.

Referee #2 (Remarks for Author):

In this study the authors identify a new potential target pathway for treatment of acute lymphoblastic leukemia [ALL]. The authors performed an extensive number of experiments, quite broadly investigating different aspects of the interactions of secreted CypA [eCypA] with MC2R as the novel key receptor on ALL cells. For some points they used complementary methods including

an antibody presumed to block the interaction between eCypA and MC2R, and MC2R knockdown or KO Nalm6 cells. Among others, they link the interaction of this ligand/receptor to downstream activation of cAMP production and phosphorylation of the transcription factor CREB. They also show that eCypA stimulation of cells is antiapoptotic and stimulates endothelial transmigration. In vivo, the anti- eCypA has an anti-leukemia effect in two PDX models.
Overall this is an interesting study that reports on a novel interaction between extracellular cyclophilinA and MC2R, the melanocortin 2 receptor (MC2R). No involvement of MC2R in leukemia has been reported.

Specific numbered comments
Major:
1. The initial specific rationale for focusing on CypA and MC2R is not clear. The Introduction only states: CypA is associated with various inflammatory conditions... "However the involvement of eCypA in hematological malignancies such as ALL remains unclear". The rationale for investigating MC2R in the Introduction cites a review outlining the importance of the TF CREB in ALL. However many other factors can stimulate CREB activity other than MC2R signaling- why focus on MC2R?
2. In the in vivo treatment with antibodies against eCypA, differences in survival with controls are modest in all three experiments presented [one with a cell line, and two with patient samples]. Moreover, although the investigators do use antibodies against eCypA and knock out MC2R in the ALL cells, they do not include experiments in which knock out of the prolyl isomerase A gene [PPIA] encoding CypA in the ALL cells was done. Mice deficient for Ppia are viable [for example PMID: 26932182]. Seeing that they indicate that CypA is produced endogenously in the ALL cells it is difficult to understand why no knockout or knockdown experiments were done to support the importance of CypA to ALL survival.

3. Patient samples are inadequately described. The manuscript does not even discriminate if samples are exclusively B-ALL or also T-ALL. Line 97. We measured expression of eCypA in PB of patients with ALL. Are these untreated pts? Diagnosis? Relapsed? Did these pts have ALL cells in PB? Adult? Pediatric? Line 102. The authors cite Brady et al 2022 as the source of data on which they based Fig 1c. That paper focused on "2754 children, adolescents and young adults (AYA) with newly diagnosed B-ALL (n=2288) or T-ALL (n=466) enrolled on St. Jude Children's Research Hospital (St. Jude, n=899) and Children's Oncology Group (COG, n=1855) protocols". Which samples were included in the analysis presented here? The graph shown in Fig 1E is presumed to be adult B-ALL based on the poor OS? Line 106. What are four external patient cohorts? Figure 7: What subtype of B-ALL are PDX-LB12 and QB4? The methods only states that they are pediatric samples. Then on line 299 the authors remark 'MLL-rearranged'. Which sample? Both? How is it possible that after a second passage in mice the samples contain both normal [pre-B, cycling pre-B] and leukemic ["notable MLL-r'] B cells? Then on line 300 they talk about drug-resistant MLL-R cells. Was this sample from a previously treated patient? Line 348/351: ..'small but distinct MLL-R-ALL cells' Small? 'This sub clone is clinically significant because MLL R ALL is strongly associated with chemoresistance and early relapse'. What subclone?

4. What is the significance of the secretion of eCypA by normal PBMC exposed to conditioned medium from Nalm6? Do the authors suggest that the eCypA secreted by the ALL cells induces eCypA secretion by normal cells? No evidence is presented for this. Would that take place through the MC2R receptor on these diverse immune cells?

5. Based on publicly available RNA expression data, MC2R expression in different B-ALLs appears to be quite low. On the other hand, based on the studies presented here, expression of eCypA is high. Thus it is difficult to reconcile the proposed role of MC2R as the major receptor for eCypA in B-ALL.

6. Methods and reagents are inadequately documented and described. What is the source of the eCypA antibody used for treatment of the mice? The antibody used for Western blots? This antibody is used in Fig. 3J where it seems to detect at least 3 bands. No molecular weight markers are included and the blots are all cropped. Full Western blots need to be included as supplementary materials. The ab is also used in Fig 4G and Fig 5e. Where are data showing quality controls / specificity of the ab? Where are data showing these abs bind to both mouse and human eCypA? What is the source of the eCypA used to treat mice [line 135]?. The authors state that MC2R was identified as a potential binding partner of Fc CypA in two ALL cell lines without providing the full data on what other proteins were identified and what their scores were. Corroboration of the interaction between eCypA and MC2R [Fig. 3a-f] was done by overexpression in 293T cells, not by showing that the endogenous proteins in B-ALL cells interact.

7. A single cell line, Nalm6 is used for many of the experiments.

8. There is no mention in the figure legends how many times experiments were repeated independently.

9. Line 183. NALM6 cells were treated with recombinant CypA alone or with CypA mAb. No control IgG? No control recombinant protein?

10. The study presents a large number of interesting experimental results related to the main topic but these are not discussed. For example, the identification of where eCypA binds to MC2R is interesting but is not further discussed "Molecular docking simulation to visualize the binding model between CypA and MC2R NT and EC3".

11. The finding of increased expression of FN1, CD44 and MMP9 after specific stimulation of cells with eCypA is interesting but

the authors did not show that the increased expression of these genes is responsible for changes in migration. Expression was also not connected to apoptosis.

12. Lines 247-251. One of the potentially most interesting results is that eCypA levels, as measured by ELISA in PB, decreased in patients who turned out to become MRD-negative after 14 days of chemotherapy but remained unchanged in patients who were MRD-positive. If this finding was robustly supported by data, an assay for eCypA in PB could be a valuable non-invasive tool to assess the success of first-line treatment of patients. However, again, patient samples are not documented at all [high/low risk? B/T? adult/pediatric?], and only 13 samples were assayed. This should be expanded to include larger numbers of well-documented samples.

13. Fig. 6. The extension of survival of mice treated transplanted with Nalm6 with the anti-CypA antibody compared to control IgG was 4 days, and that of mice treated with the anti-CypA antibody + VDL compared to VDL was 5 days. These differences are very small and do not support the author's suggestion that 'targeting eCypA during ALL chemotherapy may enhance treatment efficacy and improve patient outcome'

Minor
Line 102. 'Consistently' as used here does not make sense in this context.
Methods: The authors describe the co-culture system in which they measure eCypA as "Target and effector cells were mixed in a specified ratio and co-seeded into the same well." This is terminology usually used for cytotoxicity assays. Are the 'effector' cells the ALL cells?
Line 212. The involvement of CD44 or MMP9 in ALL is not new. The authors do not discuss their results in the context of published literature.

Referee #3 (Comments on Novelty/Model System for Author):

Experimentation looks sound. Some stats require review. Role of CypA in ALL has not been investigated before, but a role of CypA in other cancer entities has been reported before. The strength and novelty of the paper are the molecular characterization of the CypA-MC2R-CREB signalling pathway in ALL linked it to migration and survival pathways.
Medical impact has to be awaited. Model systems used do reflect ALL, but do not necessarily represent the large heterogeneous spectrum of human ALL.

Referee #3 (Remarks for Author):

The authors' work demonstrates that extracellular cyclophilin A (eCypA) promotes the progression of B-cell precursor ALL and desensitizes leukemic cells toward chemotherapy. The observed effects can be at least partially reversed by the application of an eCypA antibody in selected in vitro and in vivo ALL models. The authors provide experimental evidence that eCypA binds MC2R, also known as ACTH binding receptor, which signals downstream along the cAMP-PKA-CREB pathway. Phosphorylated CREB elicits pleiotropic effects including trans-endothelial migration and upregulation of anti-apoptotic effectors such as BCL-xl and MCL1. The authors propose that eCypA constitutes a potential target for the treatment of ALL, since blocking of CypA by anti-CypA delayed disease progression and prolonged survival in ALL disease models including PDX-ALL.

Upregulation of CypA in various cancer entities has been previously described linked to increased proliferation, inhibition of apoptosis, and promotion of cell migration and invasion. Hence, the observed phenotype of CypA modulation in ALL is somewhat expected, albeit the ALL context had not been explored yet. The merit of the paper lies in the detailed investigation of the mechanistic underpinnings of the CypA-mediated effects on selected ALL models by deciphering the direct binding of CypA to MC2R, recapitulating downstream signaling effects through cAMP-PKA-CREB, and defining the CypA/MC2R-dependent transcriptional and translational output. The concept of therapeutical intervention by use of a monoclonal antibody directed against CypA that was widely applied throughout this study has been recently published by the same group in the context of viral pneumonia (Bai et al. 2024).

Despite the authors' commendable work on CypA-MC2R interaction and its impact on growth and survival of several B-cell precursor ALL models, this referee raises concerns regarding the relevance of the reported findings to essential features of ALL biology. Overall, experimental data provided in the paper fall short to convincingly demonstrate that ALL cells critically depend on CypA-MC2R signaling activities. Arguably, CypA might promote growth, migration, and survival of ALL models to a certain extent, but the described effects are rather moderate even though statistically significant as presented. Similarly, blocking CypA by anti-CypA mAb mostly reverses CypA in vitro effects but does not efficiently kill ALL cells, clearly discernible under in vivo

conditions. Beside a small effect size, inhibition of CypA likely causes collateral (off-target) leukemia-extrinsic effects, since CypA is involved in many biological processes often mediated through CD147 or integrin ß2 as protein folding, trafficking, T cell activation (Th1 activation/Th2 inhibition), response to inflammatory stimuli such as hypoxia, infection, and oxidative stress among others. To bring an anti-CypA treatment approach forward, the authors should look at off-target effects in suitable in vitro and immunocompetent in vivo models. Likewise, the authors did not address leukemia-intrinsic effects that are relayed through CD147 and integrin ß2, the latter of which has been identified as CypA binding receptor in Jurkat T-ALL cells by the same authors. Another caveat is the uncertain applicability of a CypA-directed treatment approach toward a broader spectrum of ALL, since the authors mainly demonstrate experimental data on gene-modified, t(5;12) near-diploid NALM6(-Luc) ALL cells complemented by some data from MLL::AF4 (KMT2A::AFF1) RS4;11 pro-B ALL and ETV6::RUNX1 t(12;21) pre-B cell lines. Commendably, the authors finally included two PDX-ALL models to prove their point in vivo. However, those PDX-ALL models lack proper subtyping (immunological and genetic features) and the characterization of their molecular features by scRNA analysis lacks rigor and clarity.
Hence, it is uncertain if the work presented herein will ultimately advance our approach toward ALL. There are several points outlined in more detail below that might be addressed by the authors to improve the quality of the manuscript.

Major points:
1. Fig.1C and E. It is unclear which NCI data sets precisely had been used for the PPIA expression and the survival analysis. Apparently, the authors refer to the TARGET data base. The authors just briefly mention 4 external cohorts of deceased ALL patients without further details. The reference given (Brady et al. 2022) is very irritating, since it is a report on genomics of pediatric ALL that is associated with an excellent overall survival (>90%). Please clarify and describe which patients (including subtypes and age groups - presumably adult ALL patients) had been included and how the survival analysis had been performed. Did you compare upper (PPIA-high) vs lower (PPIA-low) quartiles of pooled cohorts (treated to a uniform protocol?). Has PPIA expression been tested for independence in a multivariate analysis or do the authors present a univariate analysis? A multivariate analysis is crucial to draw meaningful conclusions with regard to the relevance of a potential biomarker to outcome.
2. Fig.3I: phospho-IB PKA/CREB. In RS4;11 cell lysates (right-handed panel) anti-CypA does not prevent an increase in phosphorylation of CREB at 10' and 20' time points contradicting the proposed inhibitory effects of CypA blocking on CREB-activation. Varying GAPDH levels do not correspond to evenly loaded CREB levels. It is recommended to present the whole blot including molecular size markers as supplemental material to the referees.
3. Fig.5 reveals some inconsistencies that require explanation or re-experimentation. In subfigures F and G cell viability is measured by substrate metabolism (CCK-8 assay) which is clearly increased upon CypA administration but remains rather constant over time (>0.9=90% viability) under conditions of anti-CypA +/- CypA or MC2R knockout. This finding indicates inhibitory effects on growth/proliferation rather than a significant induction of apoptotic death, which are hard to reconcile with the reduction in BCL-XL and MCL1 in subfigure E. Under VCR treatment, a partial rescue from apoptotic death is noted upon CypA administration that is compensated by anti-CypA (could you provide additional data on anti-CypA alone?). By contrast, in the absence of cytotoxic stress (DMSO control) CypA/anti-CypA did not measurably increase the apoptotic rate of NALM6 cells suggesting that anti-CypA dependent cytotoxic efficiency is generally limited by the anti-apoptotic effect size of CypA induction. In other words, the success of a CypA-directed treatment might depend on a prior challenge of ALL cells with exogenous (therapeutically applied) CypA?
4. Fig.6 F-H. It is hard to reconcile the presented bioluminescence data (VDL+IgG vs VDL+anti-CypA) in F with the life tables in H. Tumor burden does not significantly differ on d24 between the two treatment arms, but graphs diverge by d30 when about half of the VDL-CHX cohort and the first two animals of the VDL-CHX + anti-CypA cohort had to be sacrificed indicative of treatment cohort-overlapping events. By d38 all animals in the experiment had died questioning the rigor and robustness of the survival analyses. Did the authors perform a log rank test to analyze survival in H? Could the authors provide the test statistic and exact p-values?
5. Fig.7 H,I: Marginal survival differences between PDX-ALL treatment, but highly significant p-values are given. Log rank test? Test statistic?
J: Annotation of ALL subclusters in scRNA-seq UMAP is unusual. Authors differentiate between Pre-B, cycling pre-B, and MLL-R cells. ScRNA seq does allow for cell cycle phase specific annotation (early G1/GO-S-G2/M) which should be performed thoroughly for all clusters. It is unclear how the MLL-R cluster was determined. The heatmap in K suggests the annotation is simply based on KMT2A, AFF1, MEF2C etc. single cell mRNA reads. To authenticate their claim of an MLL-R subclone, the authors should provide genomic/transcriptomic/RT-PCR data on a KMT2A::AFF1 fusion that would be associated with a transactivated HOX7-10 (in particular HOX9), MEIS1 and others which are not presented. KMT2A and AFF1 mRNA are not necessarily upregulated in MLL-r. Maybe the authors rather identified a quiescent subclone with stem cell like features that resembles MLL-r ALL?

Minor points:

6. While the title of the manuscript is suitable, the 'in brief' synopsis contains claims that are not sufficiently backed by experimental data such that blocking CypA 'suppresses' ALL progression. Clearly, it does delay progression (by several days) rather than suppress ALL progression (Fig.6F,H and Fig.7H,I).
7. Physical properties, source and binding specificity (mouse-human cross-reactivity?) should be briefly described. Did the authors look at MRAP as accessory heterodimeric binding partner that modulates ACTH-MC2R downstream signaling? Authors only refer to a previous publication from the same group, in which anti-CypA was applied in the context of viral pneumonia (Bai

et al. 2024).
8. All figure legends lack sufficient information on the number of independent experiments and biological vs technical replicates.
9. Comprehensive mass spec data on CypA binding in ALL lines should be made accessible to assess MC2R binding specificity and to comply with data availability policies. CypA has been shown to bind other receptors such as CD147 and integrin beta 2, the latter of which is expressed Jurkat T-ALL cells according to the authors' own data (Bai et al. 2024).
10. p12 line 199: Specify PKA and CREB inhibitors in Mat & Meth.
11. p14: line 238 (refers to Fig. S5F). PCR-based expression analysis of BCL-XL (species=human) is likely skewed, because authors used non-defined PBMCs (leukemic blasts? Normal lymphocytes?) from ALL vs healthy donors (mononuclear cells= heterogeneous population).
12. p14 line 247-248: MRD has not been described in the paper: TCR/IGH PCR MRD or FACS MRD? Define MRD time points (postinduction or postconsolidation?) and levels for MRD negativity vs positivity.
13. p16 lines 287-290 and p18 lines 330-331: It is recommended to use more cautious wording when describing and interpreting anti-CypA-mAb treatment effects. Life tables in Fig. 7 speak their own language with minimal delay in disease progression and survival. Findings herein suggest that eCypA contributes to ALL progression, but this referee doubts that it acts as a critical driver of ALL pathogenesis questioning its potential as a therapeutic target.
14. p16 line 299 and p19 lines 347-349: The authors describe an MLL-rearranged (MLL-R) subset based on sc-RNA-seq data from PDX-ALL models. Did the authors detect a genomic MLL rearrangement by FISH or breakpoint specific (RT-)PCR or do they infer the MLL-R status from the expression of sc mRNA reads (KMTA2 and AFF1 expression)? Please clarify if you refer to an authentic MLL-rearrangement or an MLL-R like transcriptional phenotype.
The cited papers (Krivtsov et al. 2019; Lopez-Millan et al. 2024) refer to new drug developments for MLL-R rather than original reports on CHX-resistance or relapse (menin inhibitor and NG2/FLT3 PPI inhibitor, respectively). The authors should cite the original work on MLL-R.
15. P22 line 405: Information on CREB and PKA inhibitors is not sufficient.
16. P22 line 418: Describe physico-chemical details of CypA-mAb (cross-reactivity/ species/ mouse humanized?)
17. P23 lines 436-438: Immunological and genetic characterization of PDX-ALL models is missing.
18. P24: Basic mass spec experimentation details should be given. Pre-analytics, instrument etc.
19. Fig.1E: Citation of GDC target-ALL-P2 is confusing, because the authors refer to an ALL genomics paper by the US-American SJCRH and COG groups that deposited data in the TARGET data base GDC target-ALL-P2 (Brady et al. 2022 Nat Genet). A more appropriate reference would be the TARGET data base. See also above point 1.
20. Fig.4D: IB is of poor quality. Blots are overexposed and FN1 as well as MMP9 signals are hard to decipher.
21. Fig. 6A: Provide information on MRD analyses in the figure legend: PCR/flow (?) postinduction time point etc.? Legend Fig.6 (C): Typo NSG instead of NCG?
22. D,E: Please describe properties of anti-CypA in more detail in the main text or supplement. Binding affinity and species cross-reactivity etc.
23. Fig.7 N: Heatmap of TF activities could be improved by use of complementary colors (+ - color bar).

Referee #1 (Comments on Novelty/Model System for Author):

Overall the manuscript and experimental systems used are good.
I would suggest if possible using also a primary model of ALL, so that recipient mice are immunocompetent, to see if the therapeutic effect holds (or is increased/decreased) in the presence of the host's immune system

We thank the reviewer for the constructive comments. We have revised the manuscript to improve clarity and strengthen support for our conclusions, including consistent B-ALL–specific terminology, ELISA species-specificity validation, expanded evidence for eCypA–MC2R interaction across B-ALL models, improved PDX molecular annotation, and addition of a p190-BCR-ABL immunocompetent B-ALL model confirming the anti-CypA effect in a physiological context. We believe these changes address the reviewer's concerns and enhance the rigor and generalizability of the study.

Referee #1 (Remarks for Author):

In this article, Pei et al show that extracellular cyclophylin A (eCypA) is upregulated in ALL in autocrine and paracrine ways, and eCypA accelerates ALL progression in vivo. This is due to eCypA binding melanocortin 2 receptor and its consequent activation of AMOK-CREB, which up regulates CD44/FN1/MMP9 to promote migration and prevent apoptosis. In line with this, in vivo treatment with neutralizing antibodies against CypA slowed disease progression in PDXs. Overall, this is an interesting manuscript. I have some minor comments for authors to address:

1. Authors make reference to ALL throughout the manuscript, but all the models used are B-ALL, so I would ask them to clarify this. Is eCypA only relevant in B-ALL? Or also T-ALL? If only B-ALL, they should specifically mention B-ALL in the abstract and elsewhere.

We thank the reviewer for raising this important point. We agree that, although we initially used the term "ALL" for general disease context, all experimental models employed in this study, including cell lines, xenograft models, and in vivo functional assays, are derived from B-cell acute lymphoblastic leukemia (B-ALL). To improve precision, we have revised the manuscript throughout to use "B-ALL" where appropriate and to clearly define the scope of our conclusions.

Regarding the relevance of eCypA in T-ALL, our current study does not include functional validation in T-ALL models. Therefore, we cannot formally conclude that the eCypA–MC2R axis is operative in T-ALL. This point has now been explicitly acknowledged as a limitation in the Discussion, and the potential role of eCypA in T-ALL is proposed as an important direction for future investigation.

2. In Fig 2b and 2c authors measure mouse and human CypA. Are authors completely sure that these assays aren't cross reactive? This should be mentioned and/or discussed.

Prior to performing these experiments, we confirmed with the manufacturers that both

ELISA kits are species-specific and do not exhibit cross-reactivity. In addition, we further examined this issue experimentally using the same set of peripheral blood plasma samples from mice and humans. As shown in newly added **Appendix Figure S1**, when mouse and human plasma samples were measured using the mouse eCypA ELISA kit and the human eCypA ELISA kit, respectively, no cross-reactivity was detected between the two assays. These results support the specificity of the measurements shown in **Figure 2B and 2C**. We have included this validation result as Appendix Figure S1 and added the corresponding statement in the Results section.

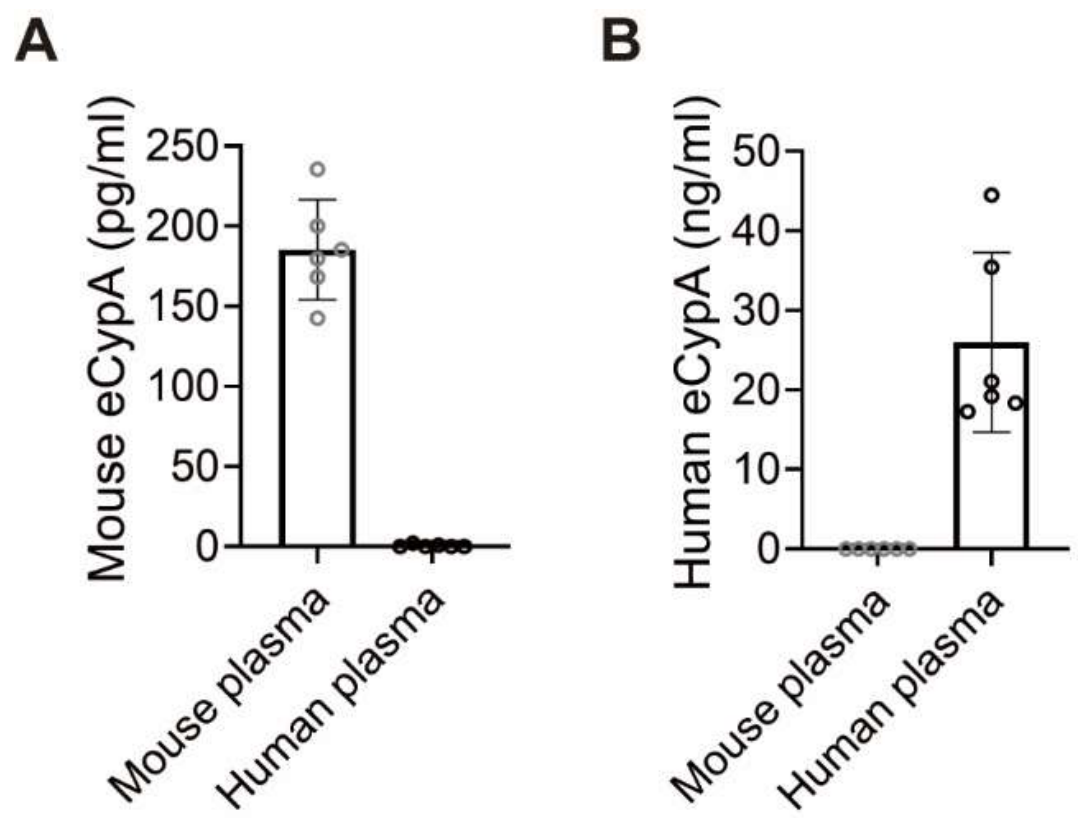

Appendix Figure S1. Cross-reactivity validation of the eCypA ELISA kits between human and mouse. (A–B) The same set of peripheral blood plasma samples from mice and humans were measured using the mouse eCypA ELISA kit (A) and the human eCypA ELISA kit (B), respectively (n = 6).

3. The binding showed in Fig3 uses 293T cells. Authors should show this in ALL cells (ideally endogenously or, if antibodies aren't good for IP, upon expressing of tagged-versions).

Fc-tagged CypA was incubated with cell lysates to pull down endogenous MC2R, followed by immunoblotting with anti-MC2R antibodies. Using this strategy, we confirmed that Fc-CypA efficiently pulled down endogenous MC2R in ALL cells. Representative results from NALM-6 cells are shown in **Figure 3B**, and additional validation in REH and RS4;11 cells is provided in **Figure EV3A**. We have added these data and revised the manuscript accordingly.

Figure 3B

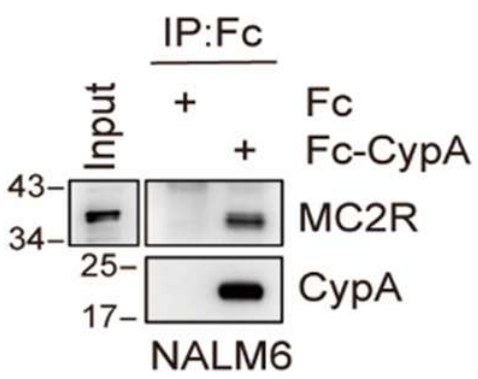

Figure EV3A

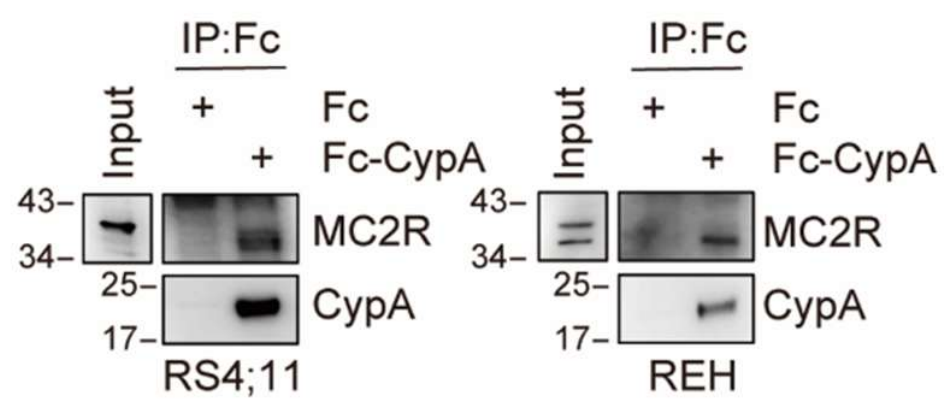

Figure 3B. NALM6 cell lysates were incubated with Fc-CypA, followed by Protein A pull-down and detection of MC2R by western blotting.

Figure EV3A. RS4;11 and REH cell lysates were incubated with Fc-CypA, followed by Protein A pull-down and detection of MC2R by western blotting.

4. Authors use a PDX in Fig 6, but don't mention anything regarding its mutations or clinical subgroup (although the reader can infer it is MLL-rearranged from the scRNAseq data). This information should be added.

We have added the molecular characteristics of the PDX models used in this study to the Methods section. PDX-LB12 represents a KMT2A::AFF1 (MLL-rearranged) B-ALL carrying an FLT3-ITD mutation, whereas PDX-QB4 harbors a FLT3 Y572C missense mutation without detectable fusion events, as determined by RNA sequencing. The detailed genetic and clinical characteristics of these PDX models have been included in Table EV5 in the revised manuscript.

5. All the in vivo assays are done in immunodeficient mice, as they involve either human cell lines or PDXs. It would be interesting to see the effects in the context of mouse primary ALL, where hosts do have their competent immune system. Would the effects of anti-eCypA be the same, increased, decreased?

We thank the reviewer for this valuable suggestion. To evaluate the effect of eCypA blockade in an immunocompetent setting, we established a p190-BCR-ABL–driven murine B-ALL model in C57BL/6 mice. Leukemia cells were subsequently transplanted into secondary recipient mice for therapeutic experiments. Leukemia burden was assessed in the bone marrow and spleen, and treatment with the anti-eCypA monoclonal antibody significantly reduced leukemia burden and prolonged survival compared with the control group. These findings indicate that the anti-leukemia effect of eCypA blockade is also preserved in an immunocompetent setting, suggesting that its therapeutic efficacy is not dependent on an immunodeficient background. The results have been added to the revised manuscript (**Figure 2F–H and Figure EV2C–D**).

Figure 2

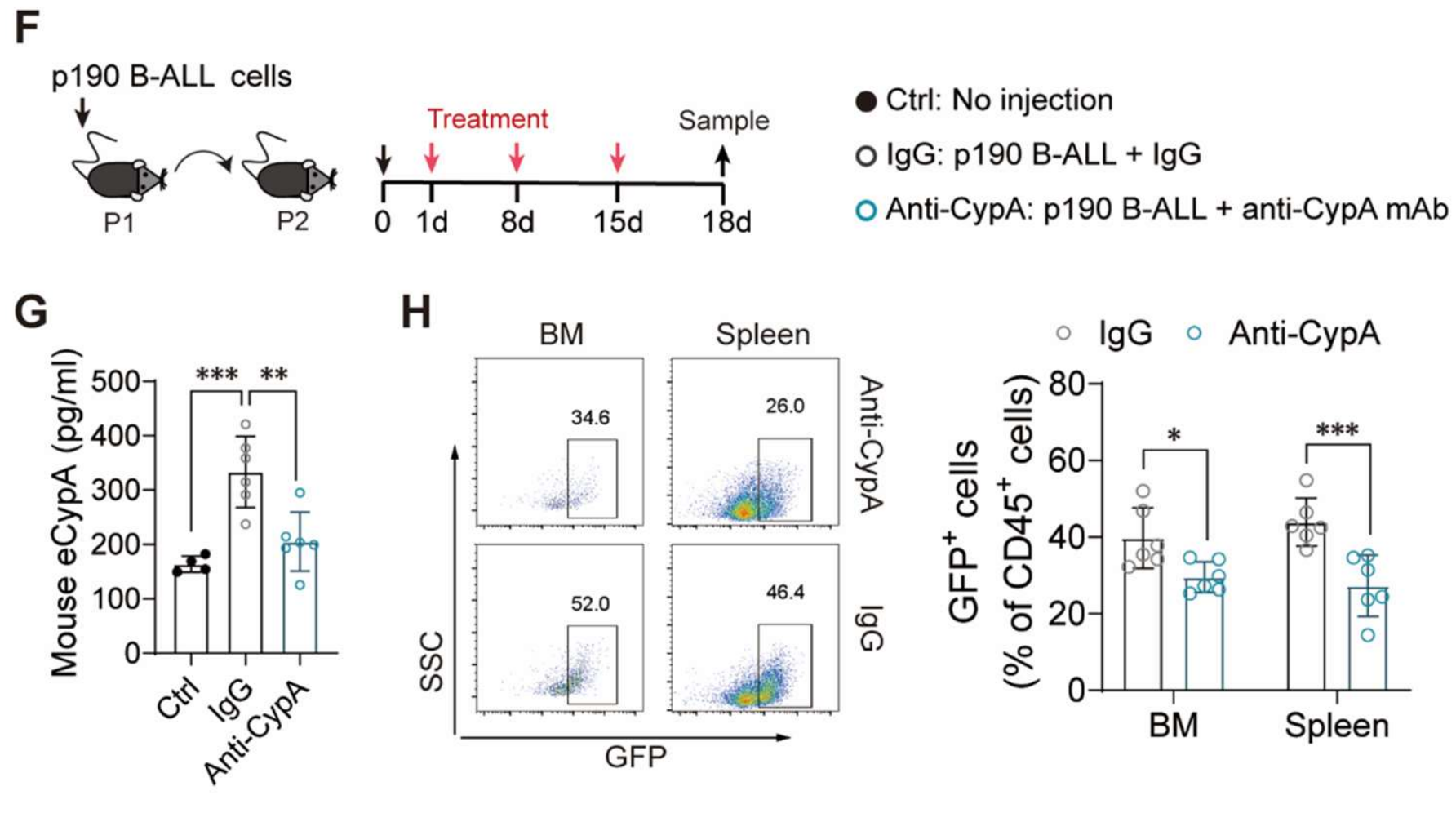

Figure EV2

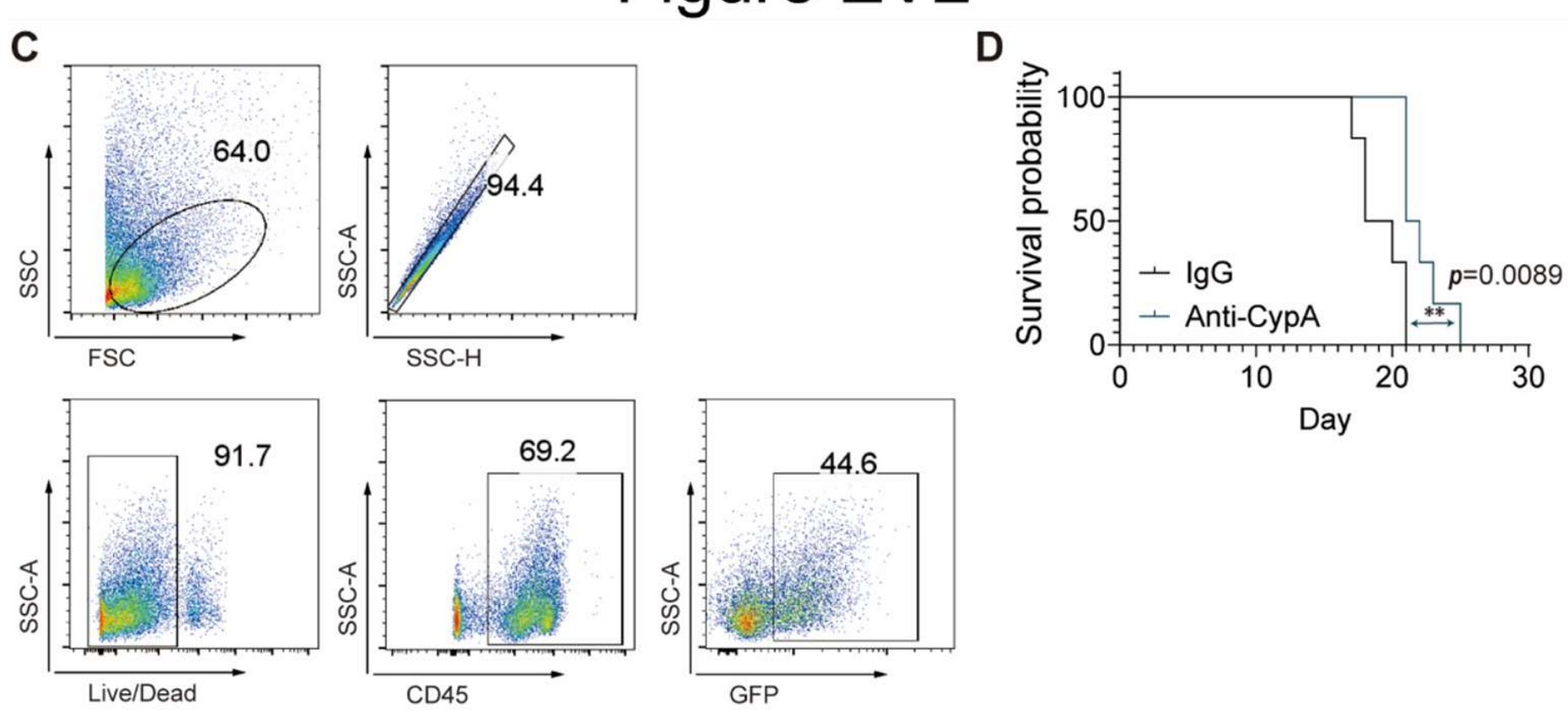

Figure 2. (F) Schematic of the p190-BCR-ABL-driven B-ALL model in C57BL/6 mice. Primary leukemic spleen cells were harvested after 2 weeks of disease development and transplanted into secondary recipient mice via tail vein injection. Anti-CypA mAb treatment was initiated the next day and administered weekly for 3 weeks; mice were sacrificed on day 18 post-transplantation. (G) ELISA analysis of mouse eCypA levels in PB plasma from Ctrl (n=4), IgG (n=6), and Anti-CypA (n=6) groups. (H) Flow cytometric analysis of GFP^+ leukemic cell infiltration in bone marrow (BM) and spleen from IgG and Anti-CypA groups, including representative flow cytometry plots and quantification (n = 6).

Figure EV2. (C) Flow cytometry gating strategy for the p190 BCR-ABL mouse model. Cells were sequentially gated on FSC/SSC to exclude debris, followed by singlet discrimination (SSC-H vs SSC-A), live cells, $mCD45^+$ leukocytes, and finally GFP^+ leukemic cells. (D) Kaplan–Meier survival analysis of p190 BCR-ABL mice treated with IgG or Anti-CypA mAb.

Referee #2 (Comments on Novelty/Model System for Author):

1. Technical quality (including statistical analysis). Materials and methods are inadequately explained.
Strong evidence for the conclusions that are drawn. The evidence for some of the conclusions is relatively strong. Other conclusions are less well-supported, among others because only one cell line (NALM6) or in some cases two cell lines (including RS4;11) are used. In the in vivo treatment with antibodies against eCypA, differences in survival with controls are modest in all three experiments presented [one with a cell line, and two with patient samples]. Moreover, although the investigators do use antibodies against eCypA and knock out MC2R in the ALL cells, they do not include experiments in which knock out of the prolyl isomerase A gene [PPIA] encoding CypA in the ALL cells was done. Mice deficient for Ppia are viable [for example PMID: 26932182]. Seeing that they indicate that CypA is produced endogenously in the ALL cells it is difficult to understand why no knockout or knockdown experiments were done to support the importance of CypA to ALL survival.
2. Novelty. The finding of a signaling pathway from extracellular CypA via the MC2R in ALL has not been reported.
3. Medical impact. Results could be important and point to a possible new treatment target. The most important finding, which will have to be better supported, is that the levels of eCypA in PB could be used to assess whether a patient is minimal residual disease-positive after 14 days of chemotherapy.
4. Adequacy of model system. The investigators use standard techniques, which are adequate to investigate the importance of eCypA in ALL.

We sincerely thank the reviewer for the thorough and constructive evaluation. In response, we substantially strengthened rigor and interpretation by expanding methodological transparency, adding CypA (*PPIA*) knockout evidence showing eCypA–MC2R signaling is independent of intracellular CypA, validating key findings across multiple B-ALL lines, improving and extending patient-cohort/MRD analyses with Cox modeling, and revising the narrative to avoid overstatement, and the state-based scRNA-seq framework.

Referee #2 (Remarks for Author):

In this study the authors identify a new potential target pathway for treatment of acute lymphoblastic leukemia [ALL]. The authors performed an extensive number of experiments, quite broadly investigating different aspects of the interactions of secreted CypA [eCypA] with MC2R as the novel key receptor on ALL cells. For some points they used complementary methods including an antibody presumed to block the interaction between eCypA and MC2R, and MC2R knockdown or KO Nalm6 cells. Among others, they link the interaction of this ligand/receptor to downstream activation of cAMP production and phosphorylation of the transcription factor CREB. They also show that eCypA stimulation of cells is antiapoptotic and stimulates endothelial

transmigration. In vivo, the anti- eCypA has an anti-leukemia effect in two PDX models. Overall this is an interesting study that reports on a novel interaction between extracellular cyclophilinA and MC2R, the melanocortin 2 receptor (MC2R). No involvement of MC2R in leukemia has been reported.

Specific numbered comments

Major:

1. The initial specific rationale for focusing on CypA and MC2R is not clear. The Introduction only states: CypA is associated with various inflammatory conditions... "However the involvement of eCypA in hematological malignancies such as ALL remains unclear". The rationale for investigating MC2R in the Introduction cites a review outlining the importance of the TF CREB in ALL. However many other factors can stimulate CREB activity other than MC2R signaling- why focus on MC2R?

In the revised manuscript, we have clarified the rationale for investigating CypA and MC2R. We expanded the Introduction to emphasize that eCypA functions as a signaling molecule that can interact with multiple cell-surface receptors, and that receptor-mediated eCypA signaling may vary across different cellular contexts and disease conditions. Importantly, MC2R was not selected a priori based on CREB signaling. Instead, it was identified as a candidate binding partner of CypA through IP–MS analysis aimed at uncovering receptors mediating eCypA signaling in ALL cells. We have clarified this point in the revised Introduction.

2. In the in vivo treatment with antibodies against eCypA, differences in survival with controls are modest in all three experiments presented [one with a cell line, and two with patient samples]. Moreover, although the investigators do use antibodies against eCypA and knock out MC2R in the ALL cells, they do not include experiments in which knock out of the prolyl isomerase A gene [PPIA] encoding CypA in the ALL cells was done. Mice deficient for Ppia are viable [for example PMID: 26932182]. Seeing that they indicate that CypA is produced endogenously in the ALL cells it is difficult to understand why no knockout or knockdown experiments were done to support the importance of CypA to ALL survival.

We thank the reviewer for this important suggestion. Regarding the modest survival differences observed in the *in vivo* experiments, we note that although the extension of survival is limited, the effects are statistically significant across three independent models, including one cell-line–derived model and two patient-derived xenograft models. Given the aggressive nature of these leukemia models and the fact that anti-eCypA antibody was evaluated as a single-agent treatment, we interpret these results as proof-of-concept evidence that targeting extracellular CypA can impair leukemia progression.

In response to the reviewer's suggestion, we generated CypA (*PPIA*) knockout ALL cell lines and examined their functional responses. CypA knockout cells remained responsive to eCypA stimulation, showing activation of the cAMP–PKA–CREB

pathway, as well as induction of downstream gene expression, transendothelial migration, and resistance to apoptosis comparable to wild-type cells. These findings indicate that eCypA can activate MC2R signaling independently of intracellular CypA. This supports the concept that eCypA primarily functions as an extracellular signaling molecule in this context. The corresponding data have been included in the revised manuscript (**Figure 3K, EV3J, 4H–L, and 5F–M**).

Figure 3K

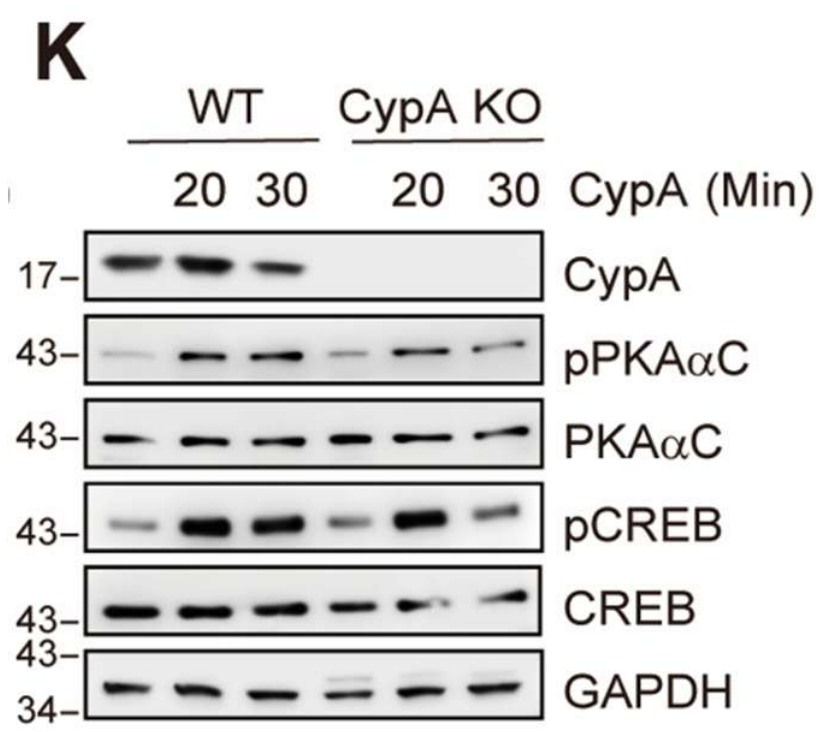

Figure EV3J

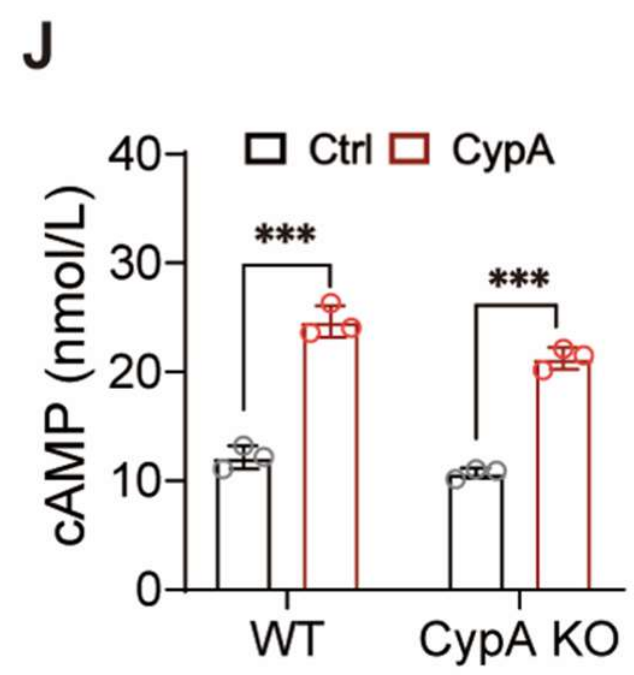

Figure 3. (K) Western blot analysis of WT and CypA KO cells.

Figure EV3. (J) cAMP levels in NALM6 WT and CypA KO cells treated with or without eCypA for 10 min (n = 3 independent biological replicates).

Figure 4

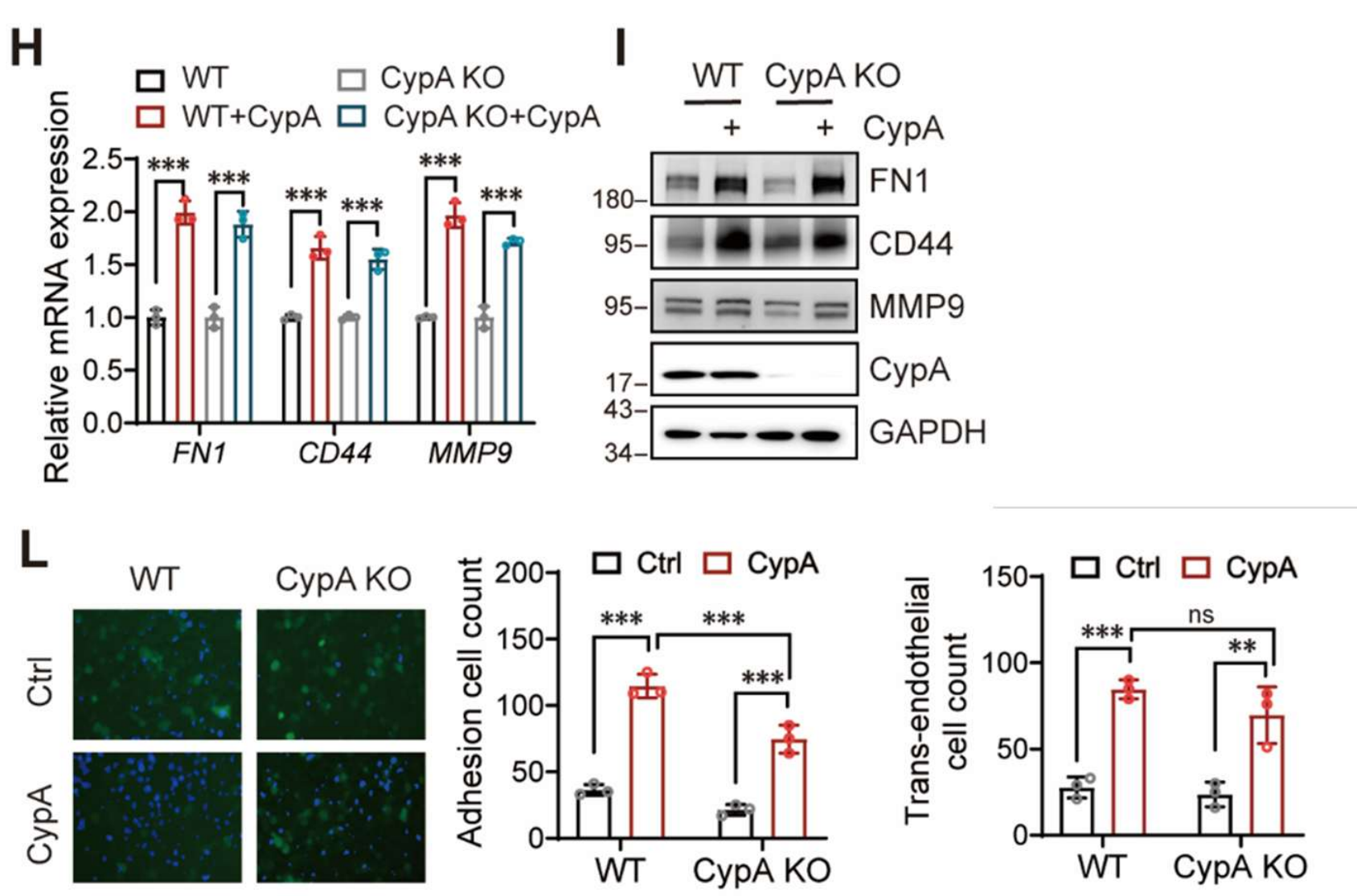

Figure 4. (H) qPCR analysis of *FN1*, *CD44*, and *MMP9* expression in NALM6 WT and CypA KO cells treated with eCypA for 30 min (n = 3 independent biological replicates). (I) Western blot analysis of FN1, CD44, and MMP9 expression in NALM6 WT and CypA KO cells treated with eCypA for 24 h. (L) Trans-endothelial migration assay showing the number of adherent and migrated NALM6 WT and CypA KO cells after eCypA treatment (4 h for adhesion and 24 h for migration) (n = 3 independent biological replicates).

Figure 5

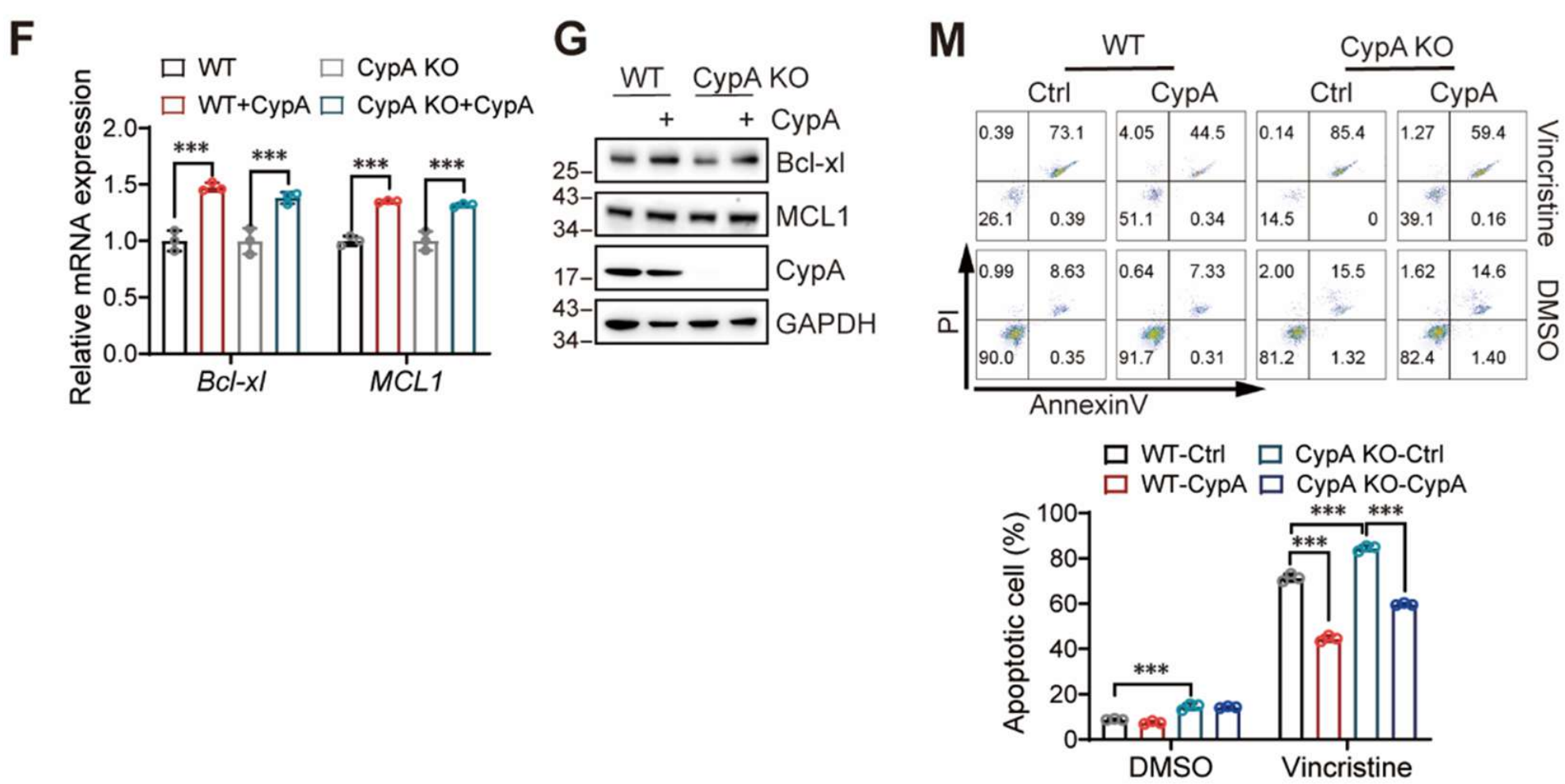

Figure 5. (F) qPCR analysis of Bcl-xL and MCL1 expression in NALM6 WT and CypA KO cells treated with or without eCypA for 30 min (n = 3 independent biological replicates). (G) Western blot analysis of Bcl-xL and MCL1 expression in NALM6 WT and CypA KO cells treated with eCypA for 24 h. (M) NALM6 WT and CypA KO cells were treated with vincristine, with or without eCypA, for 36 h, and apoptosis was assessed by Annexin V/PI staining (n = 3 independent biological replicates).

3. Patient samples are inadequately described. The manuscript does not even discriminate if samples are exclusively B-ALL or also T-ALL. Line 97. We measured expression of eCypA in PB of patients with ALL. Are these untreated pts? Diagnosis? Relapsed? Did these pts have ALL cells in PB? Adult? Pediatric?Line 102. The authors cite Brady et al 2022 as the source of data on which they based Fig 1c. That paper focused on "2754 children, adolescents and young adults (AYA) with newly diagnosed B-ALL (n=2288) or T-ALL (n=466) enrolled on St. Jude Children's Research Hospital (St. Jude, n=899) and Children's Oncology Group (COG, n=1855) protocols". Which samples were included in the analysis presented here? The graph shown in Fig 1E is presumed to be adult B-ALL based on the poor OS? Line 106. What are four external patient cohorts? Figure 7: What subtype of B-ALL are PDX-LB12 and QB4? The methods only states that they are pediatric samples. Then on line 299 the authors remark 'MLL-rearranged'. Which sample? Both? How is it possible that after a second passage

in mice the samples contain both normal [pre-B, cycling pre-B] and leukemic ["notable MLL-r'] B cells? Then on line 300 they talk about drug-resistant MLL-R cells. Was this sample from a previously treated patient? Line 348/351: ..'small but distinct MLL-R-ALL cells' Small? 'This sub clone is clinically significant because MLL R ALL is strongly associated with chemoresistance and early relapse'. What subclone?

We have revised the manuscript to clarify the patient cohort description, data sources, and single-cell analysis as follows.

First, we have substantially improved the description of the patient cohort used for serum eCypA analysis. A new supplementary table (Table EV1) has been added, summarizing the clinical characteristics of all samples, including sex, age, disease status (newly diagnosed or relapsed), leukemia type, cytogenetic/molecular features, sample source, and available hematological parameters. Samples used for eCypA measurement were derived from patient peripheral blood or bone marrow specimens, as summarized in Table EV1**.** All cases included in this analysis are B-ALL. In addition, patients were stratified into newly diagnosed and relapsed groups, and increased eCypA levels were observed in both groups (**Figure 1A**).

Figure 1

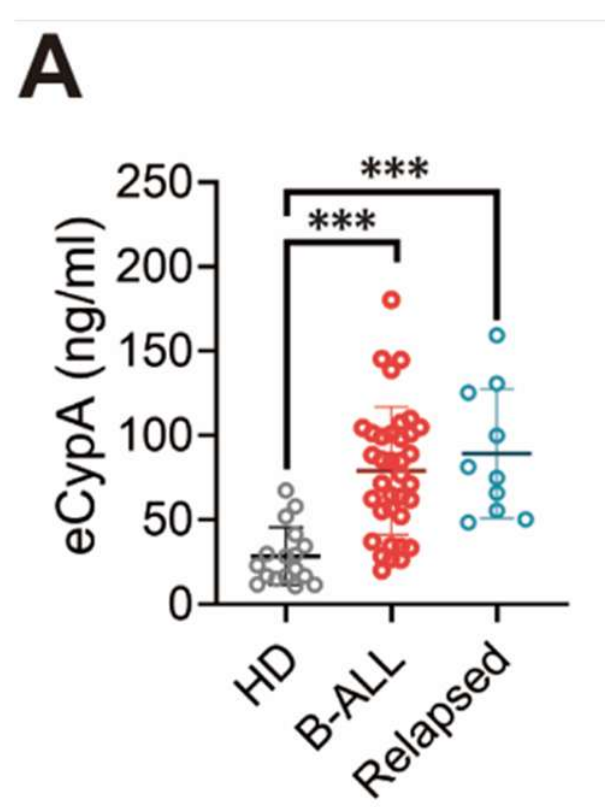

Second, we have clarified the source and analysis of the public datasets used in **Figure 1C**. In the revised manuscript, **Figure 1E** has been updated, and the survival analysis has been re-performed using the TARGET-ALL-P2 dataset (NCI Genomic Data Commons) to ensure data consistency and reliability. The TARGET-ALL-P2 dataset comprises predominantly pediatric and adolescent/young adult (AYA) patients. For the present study, only patients diagnosed with B-ALL were included in the analysis. The corresponding clinical information of the included patients is provided in Table EV3. Samples were included in the analysis based on the availability of *PPIA* expression data. According to sample source and disease stage, samples were stratified into three groups: initial peripheral blood (PB), initial bone marrow (BM), and relapse bone marrow (BM). Multivariate Cox regression analyses for each subgroup are summarized in Table EV2. In addition, Kaplan–Meier survival analyses were performed for the three groups based

on *PPIA* expression levels, and the updated survival curves are presented in **Figure 1E**. In **Figure 1C**, *PPIA* expression was compared across PB, initial BM, and relapse BM samples.

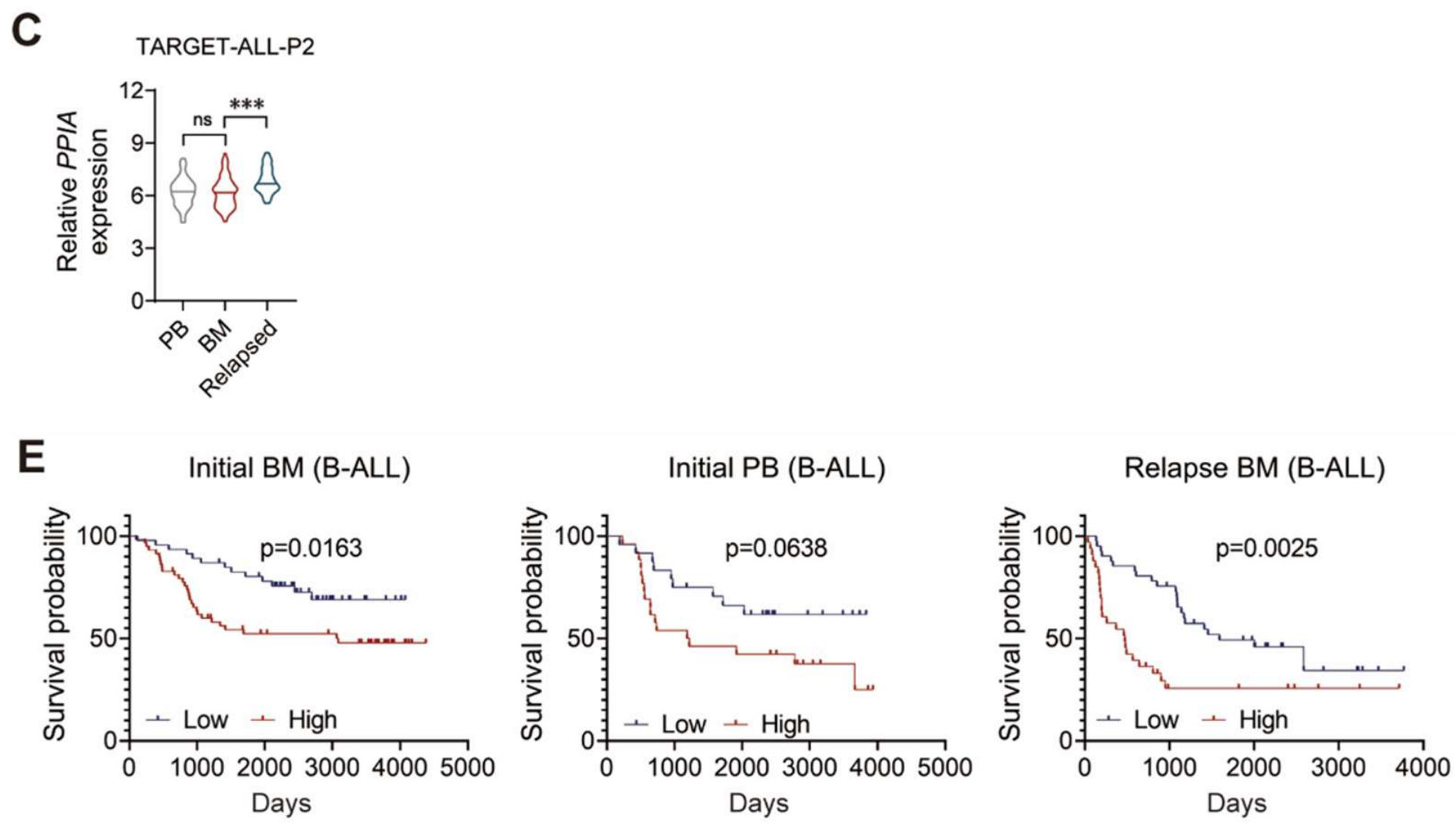

Figure 1. (C) PPIA mRNA expression levels in bone marrow (BM) and peripheral blood (PB) samples from the TARGET-ALL-P2 cohort obtained from the UCSC Xena platform. Only B-ALL samples were included. Samples were stratified into initial PB (n = 52), initial BM (n = 105), and relapse BM (n = 74). (E) Kaplan–Meier survival curves of B-ALL patients stratified by PPIA expression in initial BM (n = 104), relapse BM (n = 74), and initial PB (n = 50). Patients were divided into high and low expression groups using the median expression level as the cutoff. Statistical significance was assessed using the log-rank test. For the relapse BM cohort, survival time was calculated from relapse to death or last follow-up.

Table EV2. Multivariate Cox regression analysis of overall survival in B-ALL patients based on initial and relapse bone marrow samples

Variable	Initial BM HR (95% CI)	P value	Relapse BM HR (95% CI)	P value	Initial PB HR (95% CI)	P value
PPIA expression	2.05 (1.38–3.03)	<0.001	1.80 (1.14–2.82)	0.011	1.92 (1.08–3.42)	0.026
Age	1.07 (1.02–1.13)	0.009	1.04 (0.98–1.11)	0.184	1.07 (0.99–1.16)	0.079
Gender (Male vs Female)	1.50 (0.80–2.82)	0.207	2.23 (1.20–4.17)	0.012	0.88 (0.38–2.03)	0.762
Fusion status	1.45 (0.65–3.24)	0.367	1.25 (0.59–2.69)	0.561	1.29 (0.53–3.15)	0.570

Initial BM: n = 105, events = 42; Relapse BM: n = 74, events = 47; Initial PB: n = 50, events = 26

Third, we have added detailed information on the PDX models used in this study (**Table EV5**). PDX-LB12 is a pediatric B-ALL sample harboring KMT2A::AFF1 and FLT3-ITD, whereas PDX-QB4 carries an FLT3 Y572C mutation without detectable fusion events. Both models were established from primary bone marrow mononuclear cells. In this study, both models were used for in *vivo* validation, and PDX-LB12 was used for single-cell RNA sequencing.

Fourth, we thank the reviewer for this important comment. We agree that our initial annotation of the leukemic clusters as “pre-B” and “MLL-like” was not sufficiently rigorous and may be misleading. In particular, the term “MLL-like” implies a specific genetic subtype that cannot be reliably inferred from transcriptomic data alone, and the designation “pre-B” suggests a strict correspondence to normal B-cell developmental stages, which is not supported by our data. Therefore, to avoid overinterpretation, we have revised our annotation strategy and now refer to these populations using a neutral, state-based nomenclature (“State 1” and “State 2”). This change reflects our intention to describe transcriptional heterogeneity without imposing predefined developmental identities.

Fifth, consistent with the reviewer’s concern, we found that canonical B-cell developmental markers (e.g., DNTT, VPREB1, RAG1/2) were broadly expressed across clusters and did not define discrete stages corresponding to pre-B or pro-B cells. As shown in **Appendix Figure S5a**, these populations do not exhibit marker patterns that can be specifically or exclusively assigned to a canonical pre-B state. Accordingly, we have avoided using potentially misleading terms such as “pre-B-like.” Instead, differential expression analysis revealed that genes previously reported to be enriched in immature or progenitor-like hematopoietic and leukemic states, including FLT3, MSI2, MEF2C, and RUNX1, were significantly upregulated in State 2. In addition, cell cycle–associated genes such as MKI67 and TOP2A were enriched in the cycling subsets, allowing us to define “Cycling State 1” and “Cycling State 2” populations (**Appendix Figure S5**). The revised UMAP visualization reflecting this updated annotation is presented in **Figure 7H**. Furthermore, the heatmap (**Figure 7I**) displays representative marker genes associated with B-lineage programs, cell cycle activity, and progenitor-related transcriptional features, providing a comprehensive overview of the transcriptional differences between the identified states.

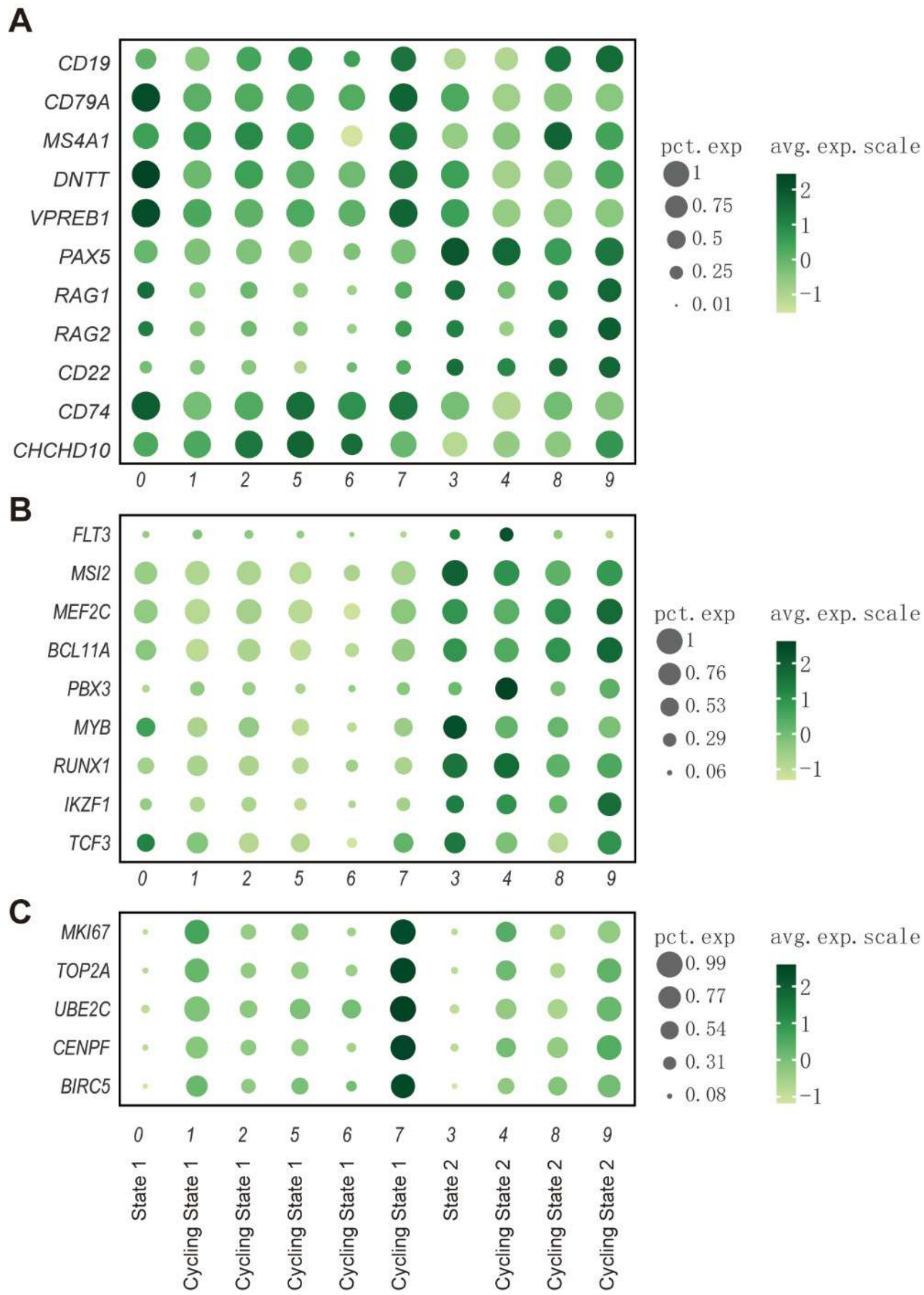

Appendix Figure S5. Transcriptional features underlying cell state annotation in scRNA-seq analysis. (A) Dot plot showing the expression of B-lineage–associated genes across the identified cell states. Canonical B-cell markers were broadly and consistently expressed across all states, supporting a homogeneous B-lineage identity. (B) Dot plot showing the expression of genes associated with progenitor-related transcriptional programs (FLT3, MYB, MSI2, RUNX1, PBX3, BCL11A, and MEF2C) across the defined states, highlighting their enrichment in State 2. (C) Dot plot showing the expression of cell cycle–related genes (MKI67, TOP2A, UBE2C, CENPF, and BIRC5), indicating proliferative activity and supporting the identification of Cycling State 1 and Cycling State 2 subpopulations.

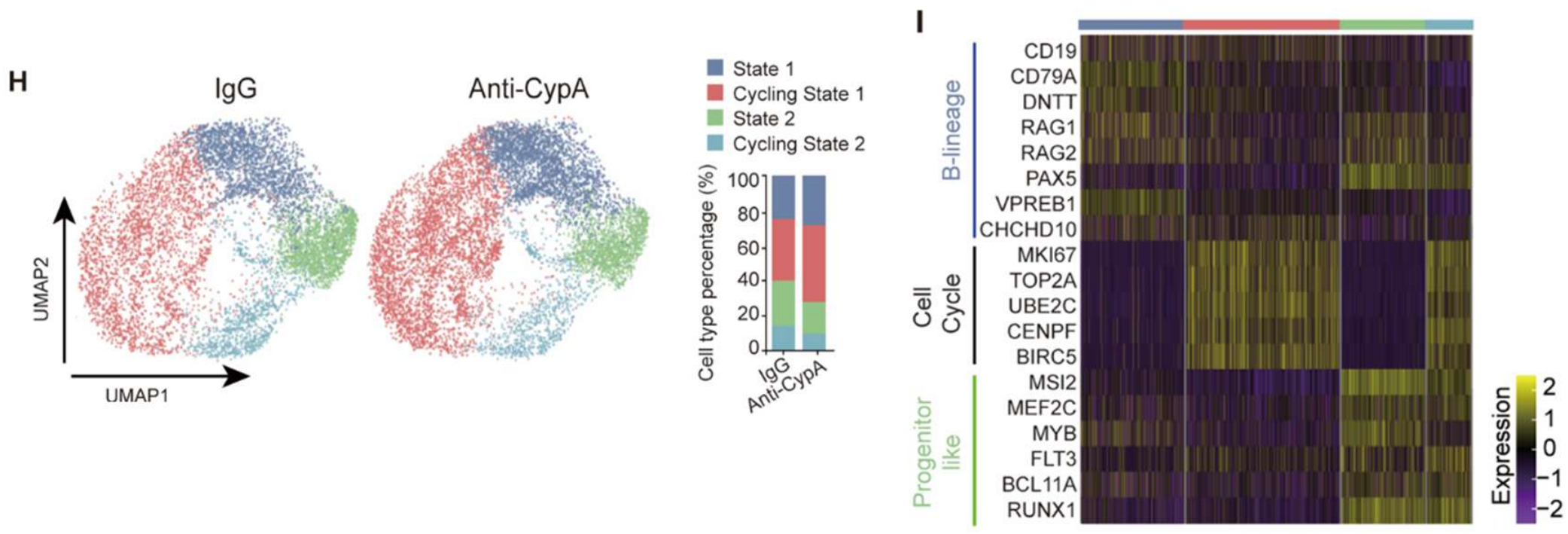

Figure 7. (H) UMAP plots (left) and corresponding bar plot (right) showing the distribution of cell states in IgG- and Anti-CypA–treated PDX-ALL samples. (I) Heatmap showing the expression of representative marker genes used for state annotation, including genes associated with B-lineage programs, cell cycle activity, and progenitor-like transcriptional programs.

Sixth, we apologize for the unclear description regarding drug resistance. The sample used in this study was derived from a treatment-naïve patient, and thus the observed features do not reflect prior therapy exposure. We have revised the manuscript to remove statements implying treatment-induced resistance.

Finally, we have revised the manuscript throughout to clarify that the identified clusters represent transcriptionally defined cell states rather than genetically distinct subclones, and to ensure consistent and accurate terminology.

4. What is the significance of the secretion of eCypA by normal PBMC exposed to conditioned medium from Nalm6? Do the authors suggest that the eCypA secreted by the ALL cells induces eCypA secretion by normal cells? No evidence is presented for this. Would that take place through the MC2R receptor on these diverse immune cells?

In our study, we observed that conditioned medium from ALL cells increased eCypA levels in normal peripheral blood white blood cells (PB-WBCs), peripheral blood mononuclear cells (PBMCs), and granulocytes. This result was intended to demonstrate that leukemic cells can influence the surrounding hematopoietic environment and contribute to elevated eCypA levels.

We agree that the underlying mechanism of this phenomenon remains unclear. Previous studies have shown that eCypA can be released from various cell types under conditions such as oxidative stress, inflammatory stimulation, and cellular activation through non-classical secretion pathways(Jin, Lungu et al., 2004, Satoh, Matoba et al., 2008, Seko, Fujimura et al., 2004, Suzuki, Jin et al., 2006). In this context, it is possible that soluble factors present in the conditioned medium from leukemic cells may induce stress or activation responses in normal hematopoietic cells, thereby contributing to increased eCypA release. However, in the current study, we did not investigate whether this effect is directly mediated by eCypA itself or by other factors present in the conditioned

medium, nor did we assess the existence of a feedback mechanism. Importantly, our study focused on the functional role of extracellular CypA in leukemic cells and its interaction with MC2R. We did not examine MC2R expression or signaling in normal immune cells, and therefore we do not draw conclusions regarding MC2R-dependent mechanisms in PBMCs or other hematopoietic cell populations. We have revised the manuscript to avoid overinterpretation and to clarify that this observation reflects a potential contribution of multiple cellular sources to extracellular CypA levels, rather than a defined mechanistic pathway.

5. Based on publicly available RNA expression data, MC2R expression in different B-ALLs appears to be quite low. On the other hand, based on the studies presented here, expression of eCypA is high. Thus it is difficult to reconcile the proposed role of MC2R as the major receptor for eCypA in B-ALL.

We appreciate the reviewer's important comment regarding the apparently low MC2R expression in publicly available transcriptomic datasets of B-ALL. Indeed, analysis of multiple public RNA-seq databases indicates that MC2R mRNA levels are generally low or close to the detection limit in most B-ALL samples. Consistently, in our own transcriptomic data from NALM6 cells, MC2R expression was also not readily detectable. Our selection of MC2R was primarily based on unbiased IP–MS analysis, in which MC2R was identified as a high-confidence interacting partner of eCypA. In addition, although MC2R transcripts are barely detectable in bulk RNA-seq datasets, our qPCR analysis using validated plasmid templates as controls consistently detected specific MC2R amplification with identical melting curves in B-ALL cells (**Appendix Figure S2**), supporting the presence of bona fide MC2R transcripts.

It is well established that GPCR signaling is highly efficient and involves substantial signal amplification cascades and high ligand sensitivity, enabling robust downstream responses even with limited receptor engagement (Rosenbaum, Rasmussen et al., 2009). Therefore, low transcript abundance does not necessarily exclude functional relevance. Importantly, our functional data further support a critical role of MC2R in eCypA signaling. Genetic deletion of MC2R markedly impaired eCypA-induced activation of the cAMP–CREB pathway and significantly suppressed leukemia cell migration and resistance to apoptosis. These results indicate that MC2R is required for efficient eCypA-mediated signaling in B-ALL cells.

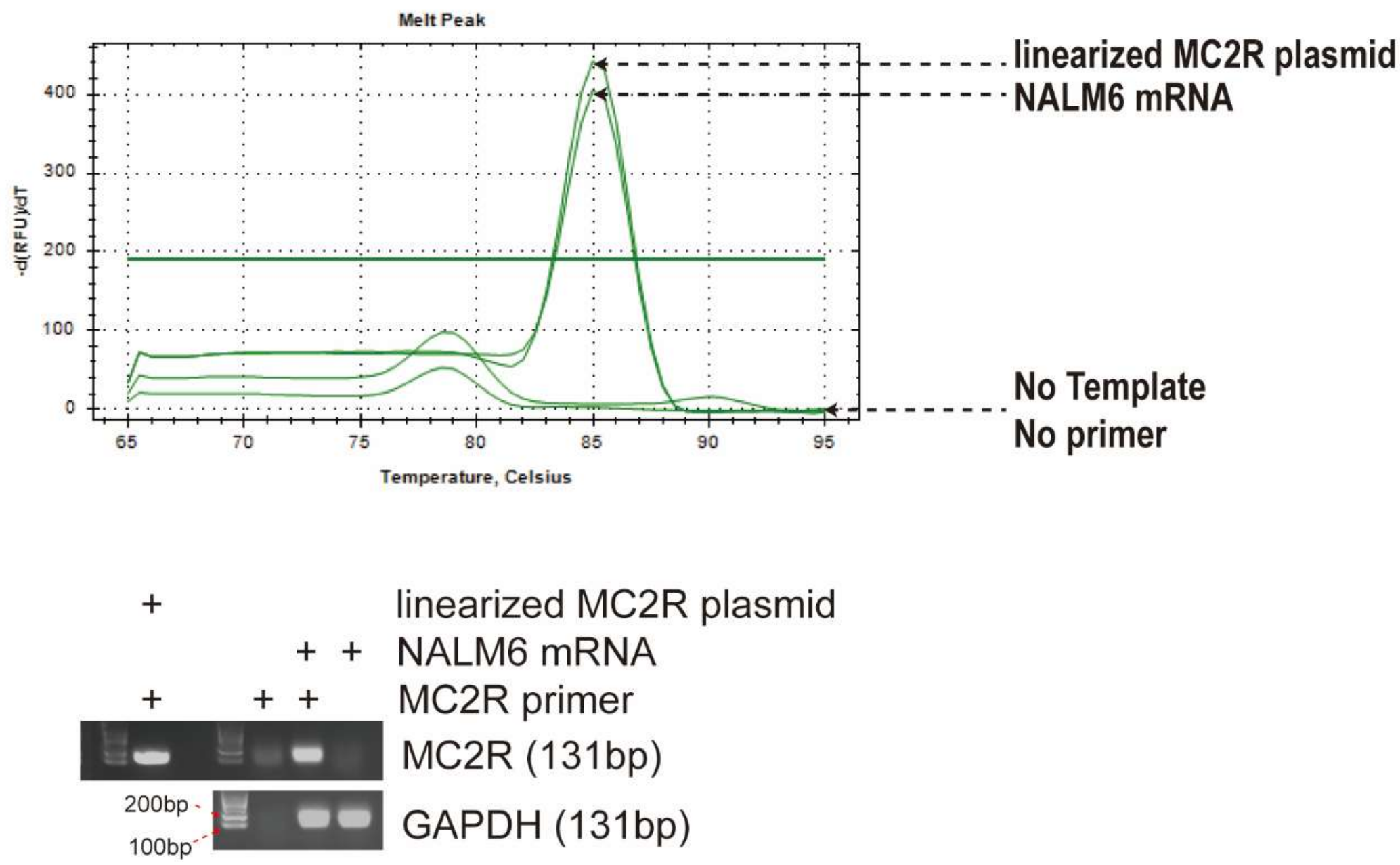

Appendix Figure S2. Validation of MC2R expression in NALM6 cells by qPCR melting curve analysis. Melting curve analysis was performed following qPCR amplification using MC2R-specific primers. A linearized MC2R plasmid was used as a positive control. NALM6-derived mRNA was used as the experimental sample with MC2R primers. Negative controls included reactions containing mRNA only or primers only. GAPDH was used as an internal control for all samples except the linearized plasmid.

6. Methods and reagents are inadequately documented and described. What is the source of the eCypA antibody used for treatment of the mice? The antibody used for Western blots? This antibody is used in Fig. 3J where it seems to detect at least 3 bands. No molecular weight markers are included and the blots are all cropped. Full Western blots need to be included as supplementary materials. The ab is also used in Fig 4G and Fig 5e. Where are data showing quality controls / specificity of the ab? Where are data showing these abs bind to both mouse and human eCypA? What is the source of the eCypA used to treat mice [line 135]?. The authors state that MC2R was identified as a potential binding partner of Fc CypA in two ALL cell lines without providing the full data on what other proteins were identified and what their scores were. Corroboration of the interaction between eCypA and MC2R [Fig. 3a-f] was done by overexpression in 293T cells, not by showing that the endogenous proteins in B-ALL cells interact.

We thank the reviewer for these important comments and have revised the manuscript to improve the documentation of methods, reagents, and supporting data.

The anti-CypA monoclonal antibody used for both *in vivo* treatment and western blotting was developed by our group and has been previously described (doi: 10.1016/j.ymthe.2024.03.008. Epub 2024 Mar 7). We have clearly stated the source and

relevant reference in the Methods section.

We have included full-length, uncropped western blot images of **Figure 3J, 4G, and 5E** with molecular weight markers in the supplementary raw data files.

Figure 3J

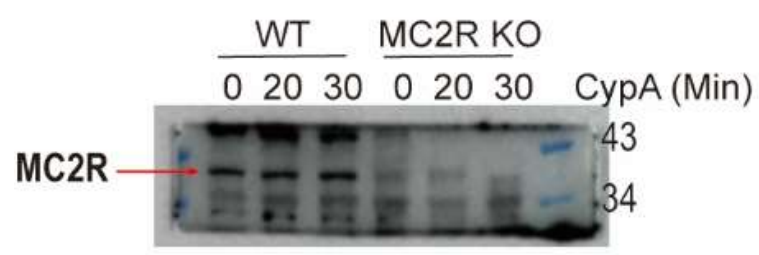

Figure 4G

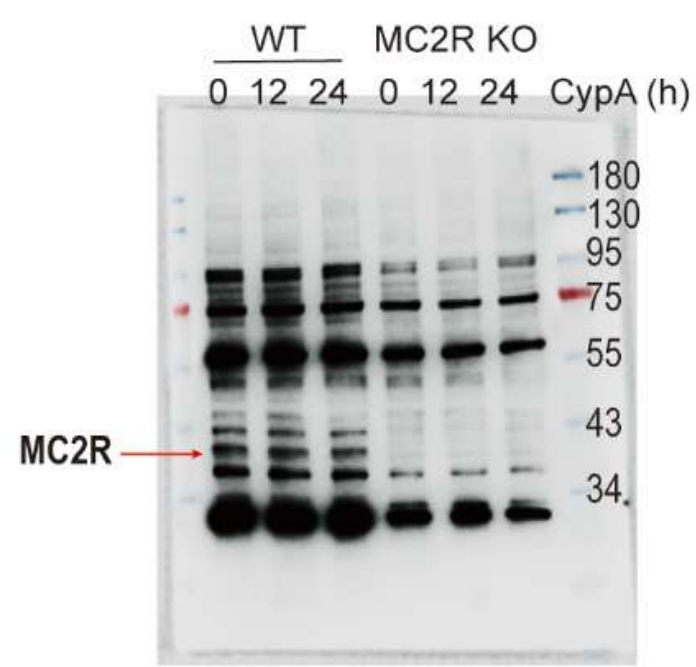

Figure 5E

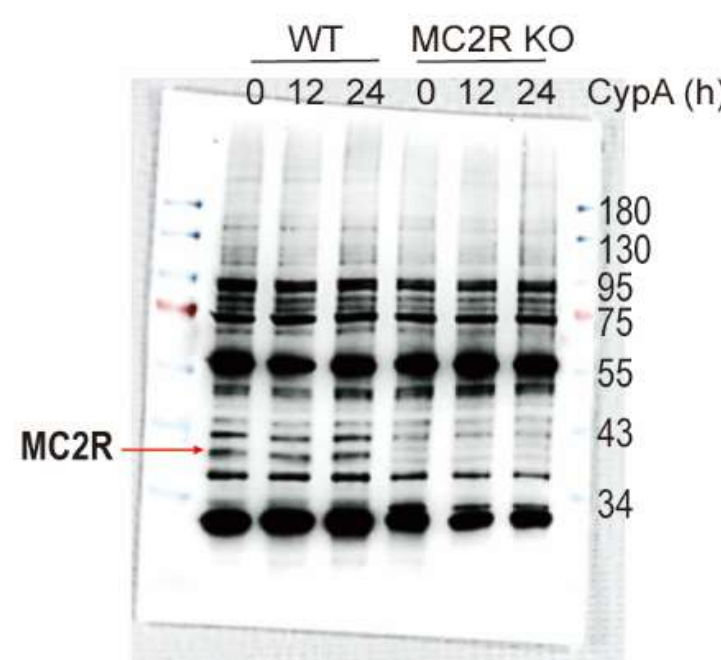

To address the cross-species validation (human and mouse CypA), we have included additional data in the **Appendix Figure S4** materials demonstrating that the anti-CypA monoclonal antibody recognizes both human and mouse CypA by western blot.

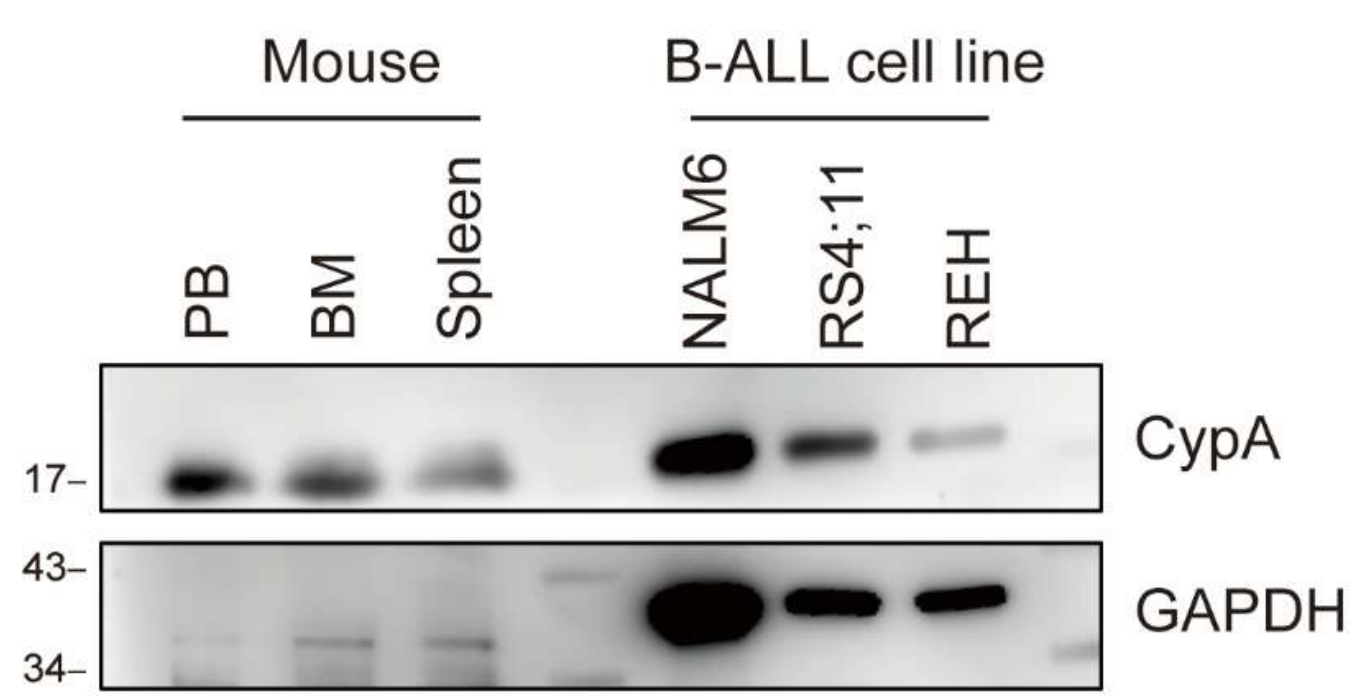

Appendix Figure S4. Validation of CypA interaction in human leukemic cells and murine hematopoietic tissues. Red blood cells were lysed from mouse peripheral blood (PB), bone marrow (BM), and spleen prior to protein extraction. Whole-cell lysates from these murine tissues, together with human B-ALL cell lines (NALM6, RS4;11, and REH), were subjected to Western blot analysis to detect CypA. GAPDH was used as a loading control.

The complete list of proteins identified by mass spectrometry, including their corresponding scores, has now been provided in **Table EV4**.

In addition to the previously shown overexpression-based assays in 293T cells, we have included pull-down experiments using Fc-CypA in three B-ALL cell lines,

demonstrating that endogenous MC2R can be captured by Fc-CypA (**Figure 3B and Figure EV3A**). These results support the interaction between eCypA and MC2R in a more physiologically relevant context.

Figure 3B

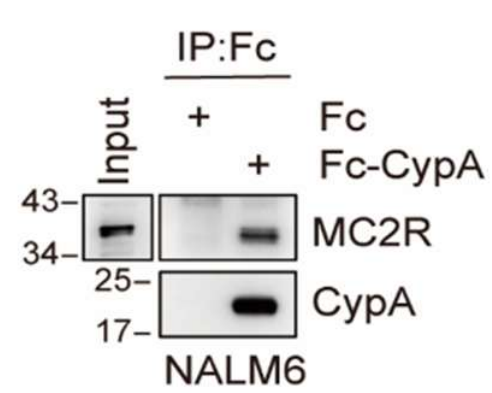

Figure EV3A

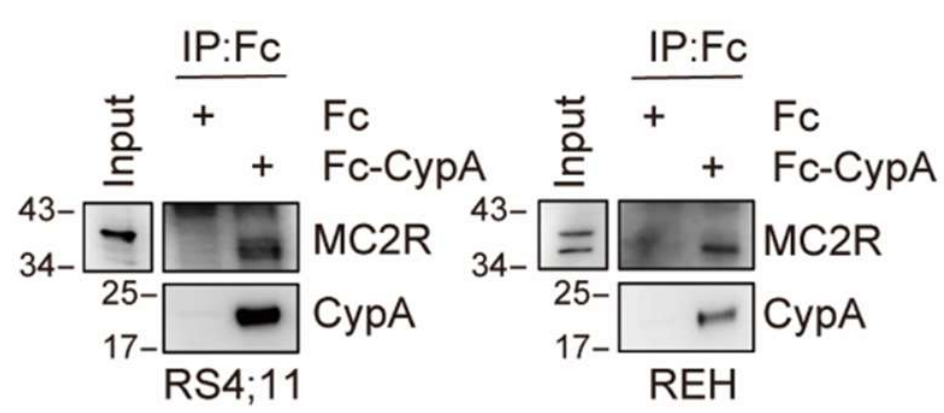

Figure 3B. NALM6 cell lysates were incubated with Fc-CypA, followed by Protein A pull-down and detection of MC2R by western blotting.

Figure EV3A. RS4;11 and REH cell lysates were incubated with Fc-CypA, followed by Protein A pull-down and detection of MC2R by western blotting.

7. A single cell line, Nalm6 is used for many of the experiments.

To address this concern, we have now extended our analyses to additional B-ALL cell lines, including RS4;11 and REH.

1). Performed co-culture experiments with these cell lines and assessed eCypA secretion from PBMCs, PB-GCs, and PB-WBCs by ELISA (**Figure EV1A–G**);

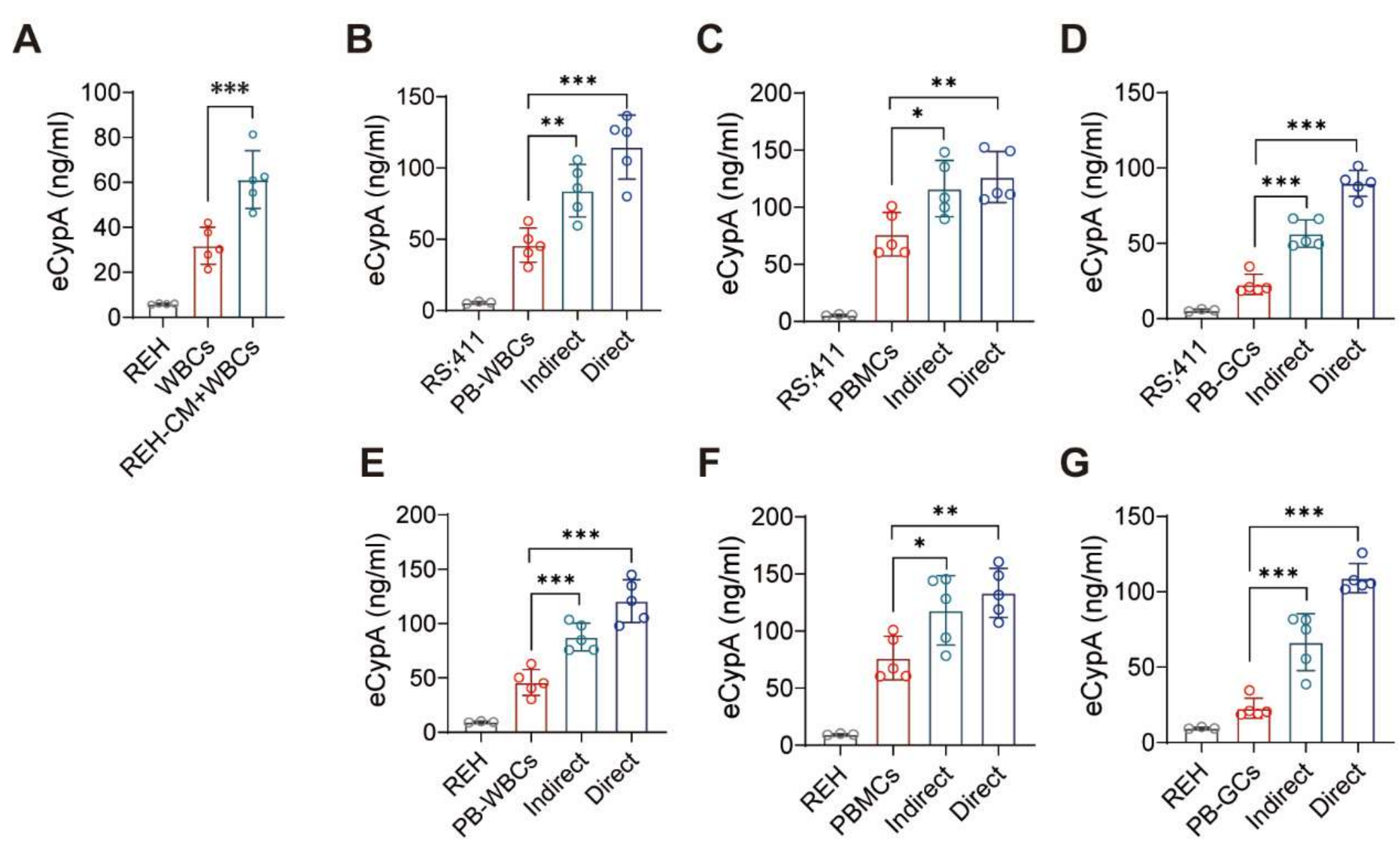

Figure EV1. ALL cells stimulate eCypA secretion from peripheral blood-derived cells. (A) ELISA analysis of eCypA levels in the supernatant of WBCs cultured alone, REH cells, or WBCs treated with REH-conditioned medium (REH-CM) (n = 5 independent donors). (B–D) ELISA analysis of eCypA levels in the supernatant from indirect and direct co-cultures of RS4;11 cells with peripheral blood WBCs (PB-WBCs) (B), peripheral blood mononuclear cells (PBMCs) (C), and peripheral blood granulocytes (PB-GCs) (D) (n = 5 independent donors). (E–G) ELISA analysis of eCypA levels in the supernatant from indirect and direct co-cultures of REH cells with PB-WBCs (E), PBMCs (F), and PB-GCs (G) (n = 5 independent donors). Data are presented as mean ± SD; each data point represents an independent donor sample measured in technical triplicates. Statistical significance was determined using one-way ANOVA. $^*p < 0.05$, $^{**}p < 0.01$, $^{***}p < 0.001$; ns, not significant.

2). Included REH (and/or RS4;11) cells in key mechanistic and functional assays, as shown in **Figure 3F, 3I, 4D, 5B, EV3I/L, EV5A/D, and EV6A/D/F**.

Figure 3F and I

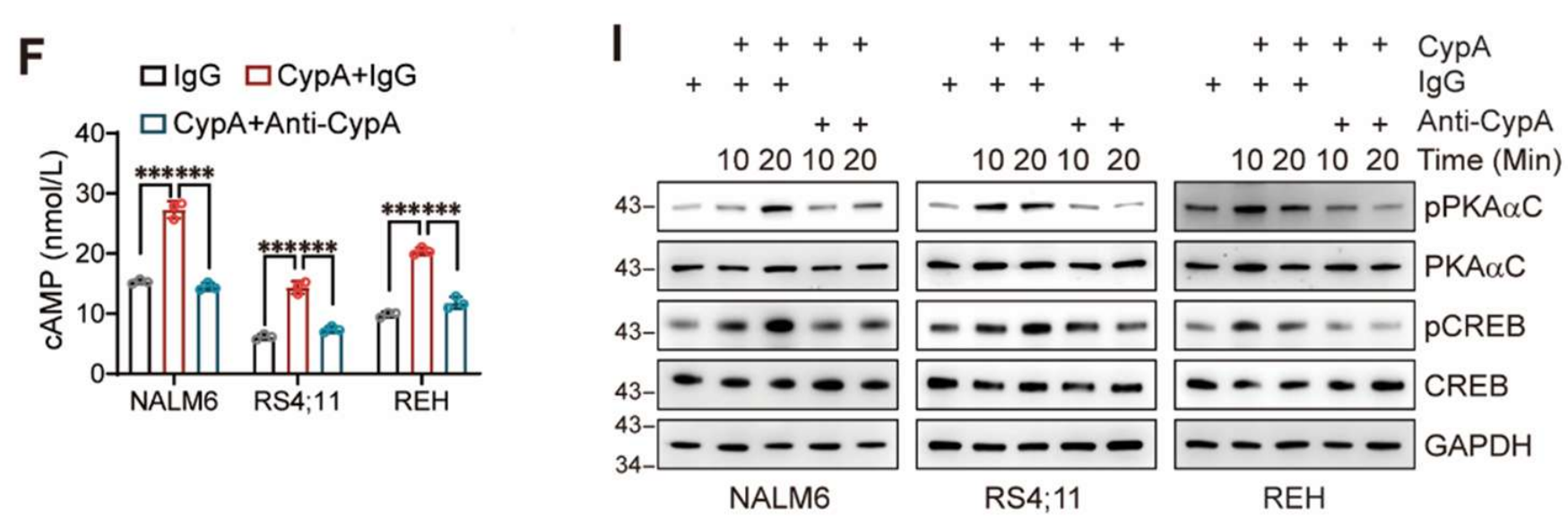

Figure EV3I and L

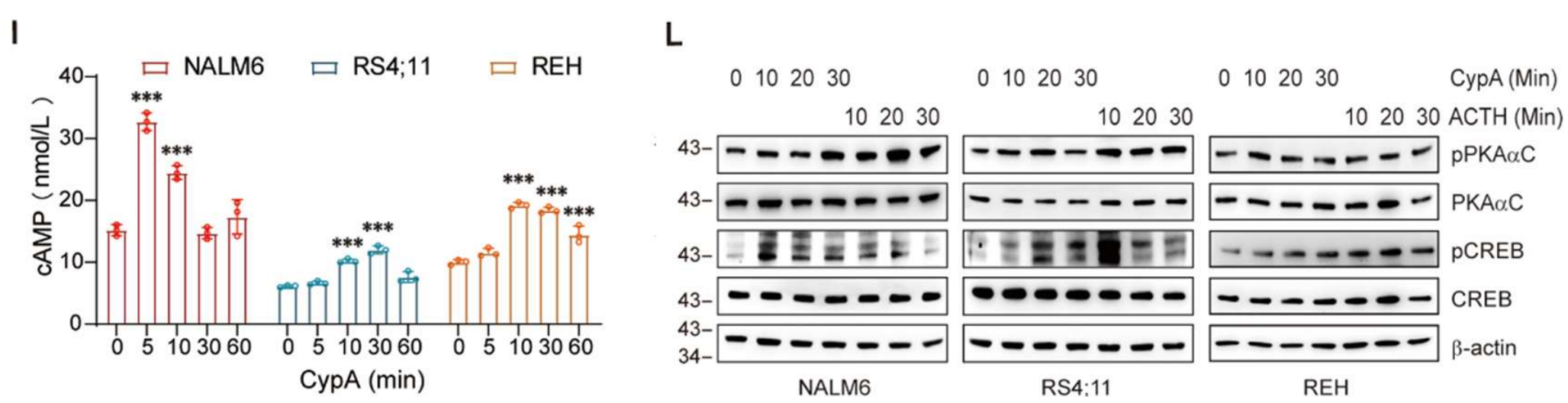

Figure 3. (F) NALM6, RS4;11, and REH cells were treated with IgG, IgG+eCypA, or Anti-CypA+eCypA for 10 min, followed by cAMP measurement (n = 3). (I) NALM6, RS4;11, and REH cells were treated as indicated, and phosphorylation of PKAαC and CREB was analyzed by western blotting.

Figure EV3. (I) cAMP levels in NALM6, RS4;11, and REH cells treated with recombinant CypA for the indicated time points (n = 3 independent biological

replicates). (L) NALM6, RS4;11, and REH cells were treated with 5 ng/mL CypA or 0.8 ng/mL ACTH for the indicated time points, followed by western blotting to assess activation of the PKA–CREB pathway.

Figure 4D

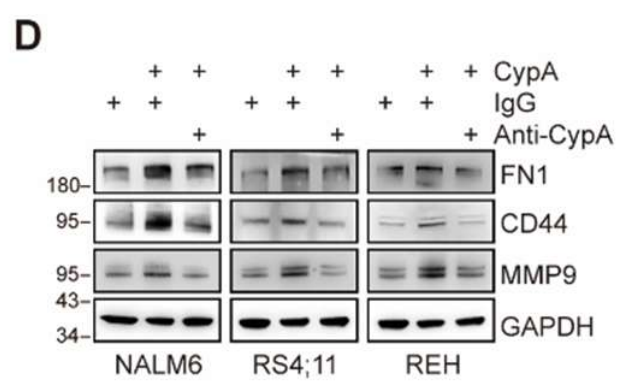

Figure 5B

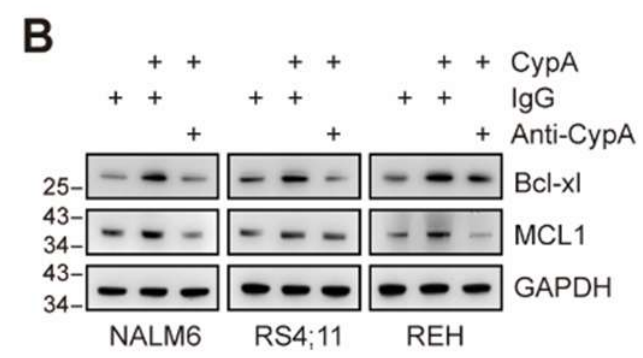

Figure EV5A and D

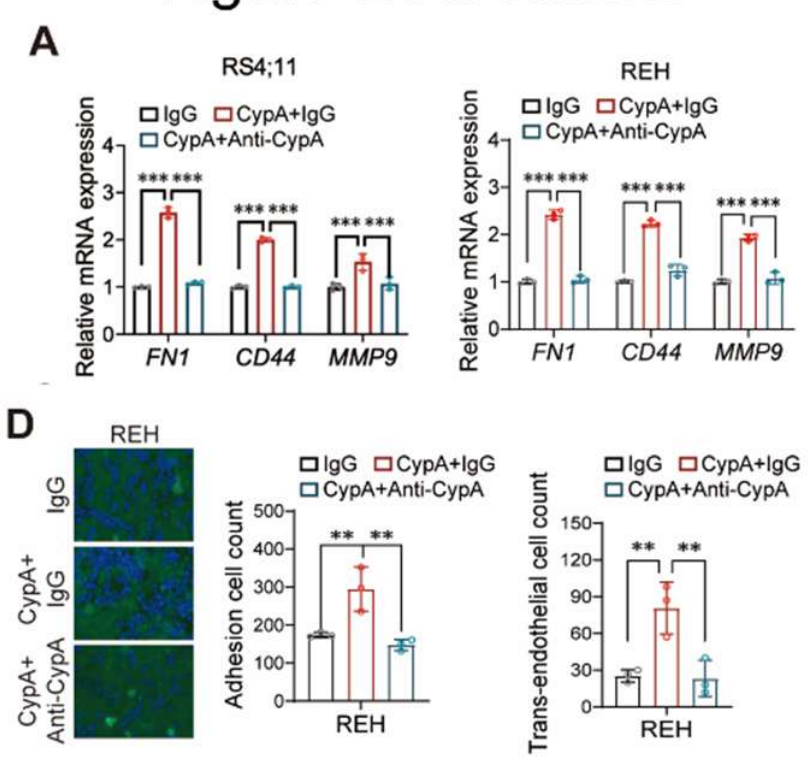

Figure EV6A,D and F

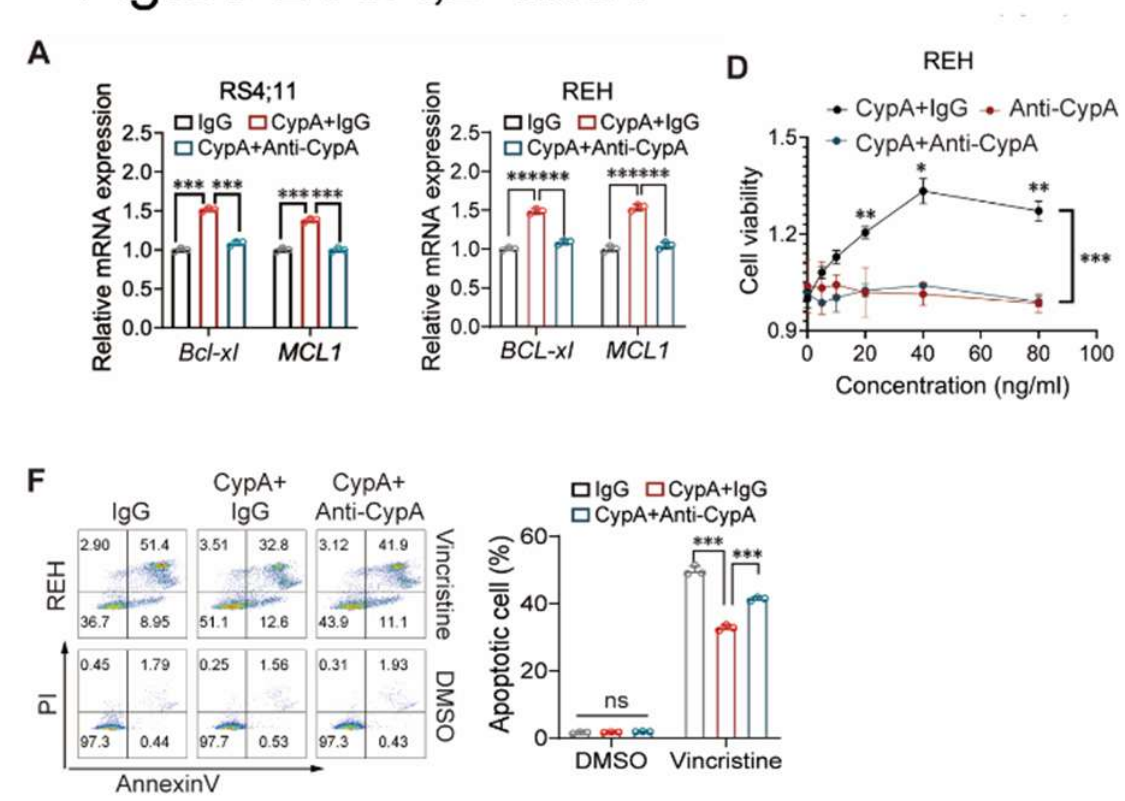

Figure 4. (D) Western blot analysis of FN1, CD44, and MMP9 expression in NALM6, RS4;11, and REH cells treated with IgG, IgG+eCypA, or Anti-CypA+eCypA for 24 h.

Figure 5. (B) Western blot analysis of Bcl-xL and MCL1 expression in NALM6, RS4;11, and REH cells treated with IgG, IgG+eCypA, or Anti-CypA+eCypA for 24 h.

Figure EV5. (A) qPCR analysis of FN1, CD44, and MMP9 expression in RS4;11 and REH cells treated with IgG, IgG+eCypA, or Anti-CypA+eCypA for 30 min (n = 3 independent biological replicates). Trans-endothelial migration assay: HUVECs were seeded in the upper chamber for 48 h to form an endothelial layer. Hoechst-stained RS4;11 (C) and REH (D) cells were added and treated with IgG, IgG+eCypA, or Anti-CypA+eCypA. The number of adherent cells was quantified after 4 h, and migrated cells were quantified after 24 h (n = 3 independent biological replicates).

Figure EV6. (A) qPCR analysis of Bcl-xL (BCL2L1) and MCL1 expression in RS4;11 and REH cells treated with IgG, IgG+eCypA, or Anti-CypA+eCypA for 30 min (n = 3 independent biological replicates). (C–D) Cell viability measured by CCK-8 assay in RS4;11 (C) and REH (D) cells treated with increasing concentrations of eCypA, Anti-CypA, or Anti-CypA+eCypA for 48 h (n = 3 independent biological replicates). (E–F) RS4;11 (E) and REH (F) cells were treated with eCypA or eCypA+Anti-CypA, with or without vincristine, for 36 h, and apoptosis was assessed by Annexin V/PI staining (n

= 3 independent biological replicates).

8. There is no mention in the figure legends how many times experiments were repeated independently.

We have carefully revised all figure legends throughout the manuscript to explicitly indicate the number of independent biological replicates for each experiment. These revisions have been applied consistently across all main figures and supplementary figures to ensure clarity, transparency, and reproducibility.

For all quantitative assays (including qPCR, ELISA, CCK-8, apoptosis assays, flow cytometry, and reporter assays), the number of independent biological replicates is now clearly stated (typically *n = 3 independent biological replicates*, unless otherwise specified). Each biological replicate was measured in technical triplicates, which is now also indicated in the figure legends.

For Western blot analyses, which are presented as representative images, we have clarified that all experiments were independently repeated at least three times with consistent results.

For experiments involving patient samples or primary cells (e.g., ELISA measurements and co-culture assays), the number of independent donors or samples is now explicitly indicated.

9. Line 183. NALM6 cells were treated with recombinant CypA alone or with CypA mAb. No control IgG? No control recombinant protein?

We apologize for the use of the IgG control group was not clearly indicated in the previous manuscript. Our experimental design was intended to focus specifically on the biological effects triggered by recombinant CypA and on whether these effects could be neutralized by the anti-CypA monoclonal antibody. The actual treatment groups were IgG-treated control cells, recombinant CypA plus IgG-treated cells, and recombinant CypA plus anti-CypA monoclonal antibody-treated cells. Therefore, the principal comparisons were IgG-treated control cells versus recombinant CypA plus IgG-treated cells, and recombinant CypA plus IgG-treated cells versus recombinant CypA plus anti-CypA monoclonal antibody-treated cells. We have revised the manuscript to clearly define the treatment groups (**Page 12**) and to clarify the labels in **Figures 4A, 4B, and EV4**.

10. The study presents a large number of interesting experimental results related to the main topic but these are not discussed. For example, the identification of where eCypA binds to MC2R is interesting but is not further discussed "Molecular docking simulation to visualize the binding model between CypA and MC2R NT and EC3".

We thank the reviewer for this insightful comment. We agree that the interaction between eCypA and MC2R, particularly the identification of the binding regions, merits further discussion. Importantly, the binding interface was first experimentally defined through domain-mapping approaches, which demonstrated that eCypA interacts with

the N-terminal (NT) region and the third extracellular loop (EC3) of MC2R. Based on these experimentally identified regions, molecular docking simulations were performed to visualize the binding model, rather than to predict the interaction de novo. In the revised manuscript, we have expanded the Discussion to clarify the biological implications of this finding. Specifically, we now highlight that the localization of eCypA binding to the extracellular domains of MC2R is consistent with the established mechanism of GPCR ligand engagement and supports a model in which eCypA interacts with MC2R to contribute to downstream signaling (**Page 21**).

11. The finding of increased expression of FN1, CD44 and MMP9 after specific stimulation of cells with eCypA is interesting but the authors did not show that the increased expression of these genes is responsible for changes in migration. Expression was also not connected to apoptosis.

Although we did not directly perform loss-of-function experiments for FN1, CD44, and MMP9 in this study, previous studies have reported that these molecules are involved in processes related to cell adhesion and migration. CD44 is a leukocyte adhesion molecule that mediates cell–cell interaction, adhesion, and migration, and has been shown to regulate the interaction between leukemic cells and the extracellular matrix (PMID: 38736891). In addition, CD44 engagement can enhance leukemic cell adhesion through cooperation with integrins such as VLA-4, thereby promoting interactions with stromal cells in the bone marrow microenvironment (PMID: 32616529). Fibronectin (FN1), as a major extracellular matrix protein, is involved in cell migration-related processes and contributes to cell motility in various physiological and pathological contexts (PMID: 38372136). Furthermore, MMP-9 has been shown to facilitate extracellular matrix remodeling in the bone marrow microenvironment and is associated with leukemic cell invasion and disease progression in B-ALL (PMID: 31919471). Therefore, the upregulation of FN1, CD44, and MMP9 observed upon eCypA stimulation is consistent with the enhanced adhesion and migration phenotypes in B-ALL cells. Similarly, we have not further demonstrated that the effect of eCypA on apoptosis is attributed to the increased expression of BCL-XL and MCL1. We have clarified these points in the Discussion section (Page 20).

12. Lines 247-251. One of the potentially most interesting results is that eCypA levels, as measured by ELISA in PB, decreased in patients who turned out to become MRD-negative after 14 days of chemotherapy but remained unchanged in patients who were MRD-positive. If this finding was robustly supported by data, an assay for eCypA in PB could be a valuable non-invasive tool to assess the success of first-line treatment of patients. However, again, patient samples are not documented at all [high/low risk? B/T? adult/pediatric?], and only 13 samples were assayed. This should be expanded to include larger numbers of well-documented samples.

We thank the reviewer for this important comment. To address this concern, we have expanded the MRD-associated cohort by including additional patient samples obtained from our biobank. In the revised **Figure 6A and 6B**, the number of MRD-negative cases

has been increased from 8 to 18, and MRD-positive cases from 7 to 12, thereby improving the robustness of the analysis. These additional samples were incorporated to provide a more representative evaluation of circulating eCypA levels in relation to treatment response.

All available patient samples (n = 46) with corresponding clinical annotations have now been summarized in **Table EV1**. The MRD subset represents patients with available post-treatment MRD assessment, while the remaining samples were included to provide broader clinical context. Due to the retrospective nature of sample collection, some clinical variables are not available for all patients and are indicated as "NA". We have clarified this point in the revised manuscript.

13. Fig. 6. The extension of survival of mice treated transplanted with Nalm6 with the anti-CypA antibody compared to control IgG was 4 days, and that of mice treated with the anti-CypA antibody + VDL compared to VDL was 5 days. These differences are very small and do not support the author's suggestion that 'targeting eCypA during ALL chemotherapy may enhance treatment efficacy and improve patient outcome'

We agree with the reviewer that the observed survival benefit is modest in this model. Accordingly, we have revised the text to avoid overinterpretation and now describe the effect as a limited but detectable survival advantage. We have also removed statements suggesting direct clinical benefit (**Page 17**).

Minor

Line 102. 'Consistently' as used here does not make sense in this context.
Methods: The authors describe the co-culture system in which they measure eCypA as "Target and effector cells were mixed in a specified ratio and co-seeded into the same well." This is terminology usually used for cytotoxicity assays. Are the 'effector' cells the ALL cells?

We agree that the term "effector cells" is not appropriate in this context, as it is typically used in cytotoxicity assays and may cause confusion. In the revised manuscript, we have replaced this terminology with more precise descriptions. Specifically, we now refer to the two cell populations as "ALL cells" and "peripheral blood-derived cells" throughout the Methods section. The description of the co-culture system has been revised accordingly to clearly reflect the experimental design (**Page 26**). In addition, we have removed the term "consistently" from Line 102, as it was not appropriate in this context.

Line 212. The involvement of CD44 or MMP9 in ALL is not new. The authors do not discuss their results in the context of published literature.

We have revised the Discussion section to better contextualize our findings within the existing literature. We have incorporated relevant references and clarified that CD44 and MMP9 are established regulators of leukemic cell adhesion, microenvironment interaction, and invasion (**Page 20**).

Referee #3 (Comments on Novelty/Model System for Author):
Experimentation looks sound. Some stats require review. Role of CypA in ALL has not been investigated before, but a role of CypA in other cancer entities has been reported before. The strength and novelty of the paper are the molecular characterization of the CypA-MC2R-CREB signalling pathway in ALL linked it to migration and survival pathways.
Medical impact has to be awaited. Model systems used do reflect ALL, but do not necessarily represent the large heterogeneous spectrum of human ALL.

We thank the reviewer for the thoughtful evaluation. In response to concerns about interpretation, statistical rigor, and generalizability, we clarified data sources and reanalyzed TARGET-ALL-P2 with transparent selection criteria and univariate/multivariate Cox models, tempered conclusions to match the moderate in vitro/in vivo effect sizes, and revised the scRNA-seq analysis using a state-based annotation framework while removing unsupported subtype assignments.

Referee #3 (Remarks for Author):
The authors' work demonstrates that extracellular cyclophilin A (eCypA) promotes the progression of B-cell precursor ALL and desensitizes leukemic cells toward chemotherapy. The observed effects can be at least partially reversed by the application of an eCypA antibody in selected in vitro and in vivo ALL models. The authors provide experimental evidence that eCypA binds MC2R, also known as ACTH binding receptor, which signals downstream along the cAMP-PKA-CREB pathway. Phosphorylated CREB elicits pleiotropic effects including trans-endothelial migration and upregulation of anti-apoptotic effectors such as BCL-xl and MCL1. The authors propose that eCypA constitutes a potential target for the treatment of ALL, since blocking of CypA by anti-CypA delayed disease progression and prolonged survival in ALL disease models including PDX-ALL.

Upregulation of CypA in various cancer entities has been previously described linked to increased proliferation, inhibition of apoptosis, and promotion of cell migration and invasion. Hence, the observed phenotype of CypA modulation in ALL is somewhat expected, albeit the ALL context had not been explored yet. The merit of the paper lies in the detailed investigation of the mechanistic underpinnings of the CypA-mediated effects on selected ALL models by deciphering the direct binding of CypA to MC2R, recapitulating downstream signaling effects through cAMP-PKA-CREB, and defining the CypA/MC2R-dependent transcriptional and translational output. The concept of therapeutical intervention by use of a monoclonal antibody directed against CypA that was widely applied throughout this study has been recently published by the same group in the context of viral pneumonia (Bai et al. 2024).

Despite the authors' commendable work on CypA-MC2R interaction and its impact on growth and survival of several B-cell precursor ALL models, this referee raises concerns regarding the relevance of the reported findings to essential features of ALL

biology. Overall, experimental data provided in the paper fall short to convincingly demonstrate that ALL cells critically depend on CypA-MC2R signaling activities. Arguably, CypA might promote growth, migration, and survival of ALL models to a certain extent, but the described effects are rather moderate even though statistically significant as presented. Similarly, blocking CypA by anti-CypA mAb mostly reverses CypA in vitro effects but does not efficiently kill ALL cells, clearly discernible under in vivo conditions. Beside a small effect size, inhibition of CypA likely causes collateral (off-target) leukemia-extrinsic effects, since CypA is involved in many biological processes often mediated through CD147 or integrin ß2 as protein folding, trafficking, T cell activation (Th1 activation/Th2 inhibition), response to inflammatory stimuli such as hypoxia, infection, and oxidative stress among others. To bring an anti-CypA treatment approach forward, the authors should look at off-target effects in suitable in vitro and immunocompetent in vivo models. Likewise, the authors did not address leukemia-intrinsic effects that are relayed through CD147 and integrin ß2, the latter of which has been identified as CypA binding receptor in Jurkat T-ALL cells by the same authors. Another caveat is the uncertain applicability of a CypA-directed treatment approach toward a broader spectrum of ALL, since the authors mainly demonstrate experimental data on gene-modified, t(5;12) near-diploid NALM6(-Luc) ALL cells complemented by some data from MLL::AF4 (KMT2A::AFF1) RS4;11 pro-B ALL and ETV6::RUNX1 t(12;21) pre-B cell lines. Commendably, the authors finally included two PDX-ALL models to prove their point in vivo. However, those PDX-ALL models lack proper subtyping (immunological and genetic features) and the characterization of their molecular features by scRNA analysis lacks rigor and clarity.
Hence, it is uncertain if the work presented herein will ultimately advance our approach toward ALL. There are several points outlined in more detail below that might be addressed by the authors to improve the quality of the manuscript.

Major points:
1. Fig.1C and E. It is unclear which NCI data sets precisely had been used for the PPIA expression and the survival analysis. Apparently, the authors refer to the TARGET data base. The authors just briefly mention 4 external cohorts of deceased ALL patients without further details. The reference given (Brady et al. 2022) is very irritating, since it is a report on genomics of pediatric ALL that is associated with an excellent overall survival (>90%). Please clarify and describe which patients (including subtypes and age groups - presumably adult ALL patients) had been included and how the survival analysis had been performed. Did you compare upper (PPIA-high) vs lower (PPIA-low) quartiles of pooled cohorts (treated to a uniform protocol?). Has PPIA expression been tested for independence in a multivariate analysis or do the authors present a univariate analysis? A multivariate analysis is crucial to draw meaningful conclusions with regard to the relevance of a potential biomarker to outcome.

We thank the reviewer for this important and insightful comment. We agree that the original description of the dataset and survival analysis was insufficiently precise and may have led to confusion. To address this, we have revised the manuscript throughout

to clearly describe the data source, patient selection criteria, and statistical workflow in the Results and Appendix sections. In addition, we have removed the reference to Brady et al. (2022).

First, we clarify that all expression and survival analyses in **Figure 1C and 1E** were based exclusively on the TARGET-ALL-P2 cohort obtained from the NCI Genomic Data Commons (GDC). Processed gene expression data were accessed through the UCSC Xena platform, while the corresponding clinical information was retrieved from the original GDC portal. These datasets were matched at the individual patient level, and all downstream analyses were performed independently by us.

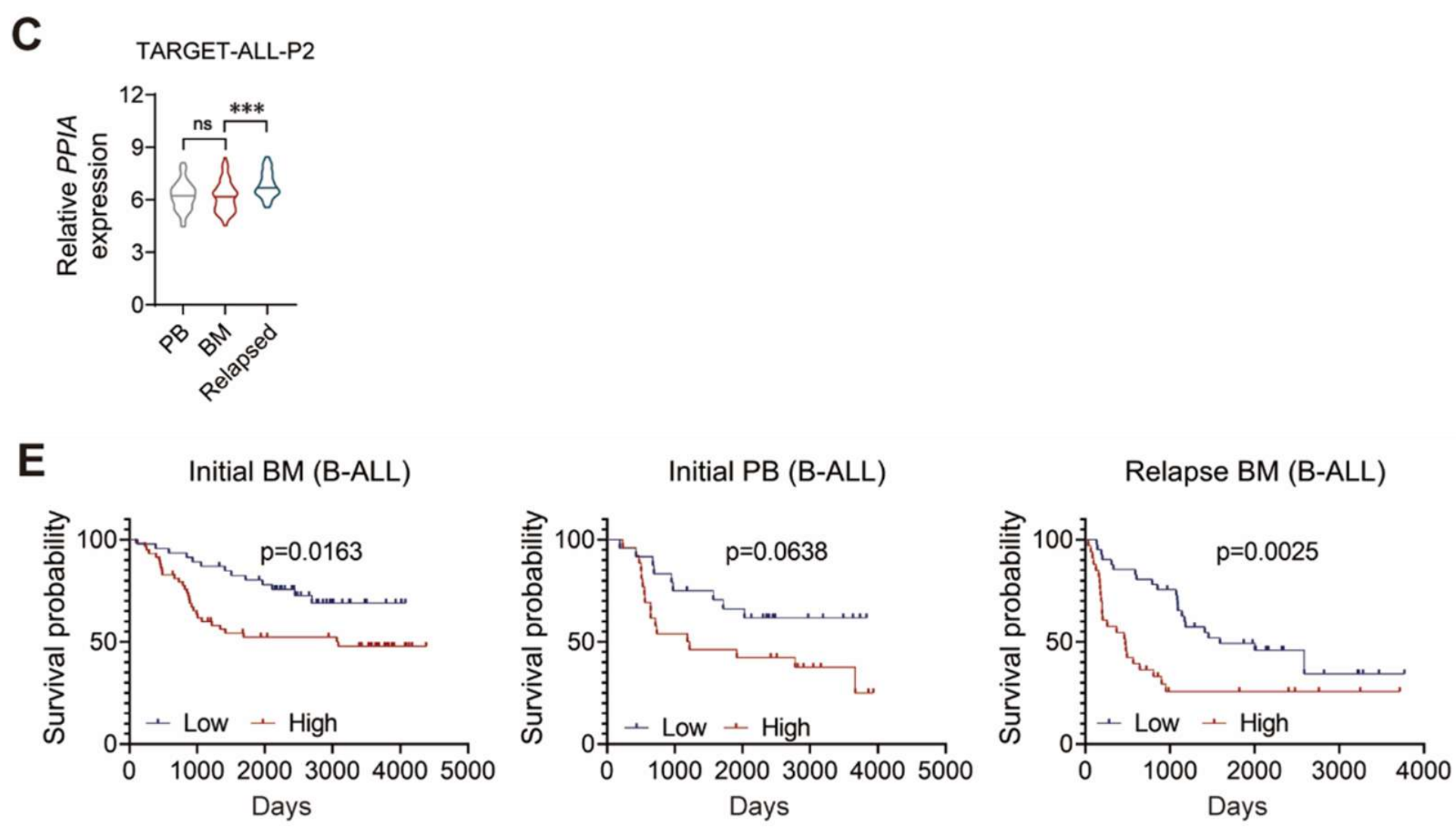

Figure 1. (C) *PPIA* mRNA expression levels in bone marrow (BM) and peripheral blood (PB) samples from the TARGET-ALL-P2 cohort obtained from the UCSC Xena platform. Only B-ALL samples were included. Samples were stratified into initial PB (n = 52), initial BM (n = 105), and relapse BM (n = 74). (E) Kaplan–Meier survival curves of B-ALL patients stratified by PPIA expression in initial BM (n = 104), relapse BM (n = 74), and initial PB (n = 50). Patients were divided into high and low expression groups using the median expression level as the cutoff. Statistical significance was assessed using the log-rank test. For the relapse BM cohort, survival time was calculated from relapse to death or last follow-up.

Second, regarding patient composition, the TARGET-ALL-P2 cohort consists predominantly of pediatric and adolescent/young adult (AYA) patients and includes both B-ALL and T-ALL cases. In the present study, we restricted our analysis to B-ALL samples only. Samples were included based on the availability of both *PPIA* expression and clinical survival data. Detailed clinical characteristics of the analyzed cohort are provided in **Table EV3**.

Third, regarding the survival analysis methodology, patients were stratified into high and low *PPIA* expression groups using the median expression value as the cutoff, rather than upper/lower quartiles. Survival analyses were conducted within the TARGET

cohort and not across pooled heterogeneous cohorts with assumed uniform treatment. To account for disease stage and sample source, patients were analyzed separately in three groups: initial peripheral blood (PB), initial bone marrow (BM), and relapse bone marrow (BM). Kaplan–Meier survival curves were generated for each subgroup, and statistical significance was assessed using the log-rank test, as shown in **Figure 1E**.

Finally, we performed both univariate and multivariate Cox proportional hazards analyses. In the multivariate models, *PPIA* expression (as a continuous variable), age, sex, and fusion status (where available) were included as covariates. These analyses show that *PPIA* expression is not an independent prognostic factor in primary PB or BM samples, whereas it is an independent risk factor in relapse BM samples. The full results are now provided in **Table EV2**.

Table EV2. Multivariate Cox regression analysis of overall survival in B-ALL patients based on initial and relapse bone marrow samples

Variable	Initial BM HR (95% CI)	P value	Relapse BM HR (95% CI)	P value	Initial PB HR (95% CI)	P value
PPIA expression	2.05 (1.38–3.03)	<0.001	1.80 (1.14–2.82)	0.011	1.92 (1.08–3.42)	0.026
Age	1.07 (1.02–1.13)	0.009	1.04 (0.98–1.11)	0.184	1.07 (0.99–1.16)	0.079
Gender (Male vs Female)	1.50 (0.80–2.82)	0.207	2.23 (1.20–4.17)	0.012	0.88 (0.38–2.03)	0.762
Fusion status	1.45 (0.65–3.24)	0.367	1.25 (0.59–2.69)	0.561	1.29 (0.53–3.15)	0.570

Initial BM: n = 105, events = 42; Relapse BM: n = 74, events = 47; Initial PB: n = 50, events = 26

2. Fig.3I: phospho-IB PKA/CREB. In RS4;11 cell lysates (right-handed panel) anti-CypA does not prevent an increase in phosphorylation of CREB at 10' and 20' time points contradicting the proposed inhibitory effects of CypA blocking on CREB-activation. Varying GAPDH levels do not correspond to evenly loaded CREB levels. It is recommended to present the whole blot including molecular size markers as supplemental material to the referees.

We agree that the original blot in **Figure 3I** did not clearly demonstrate the inhibitory effect of anti-CypA on CREB phosphorylation in RS4;11 cells, and that the loading consistency was suboptimal. To address this concern, we have repeated the experiment with improved experimental conditions and optimized antibody performance. The updated data show that anti-CypA treatment effectively reduces CREB phosphorylation. In addition, we have included results from an independent ALL cell line (REH), which consistently support the inhibitory effect of CypA blockade on CREB activation.

Furthermore, we have provided the full-length, uncropped western blots with molecular weight markers as supplementary material, along with the corresponding raw data, to ensure transparency and allow proper evaluation of protein loading and specificity.

Figure 3I

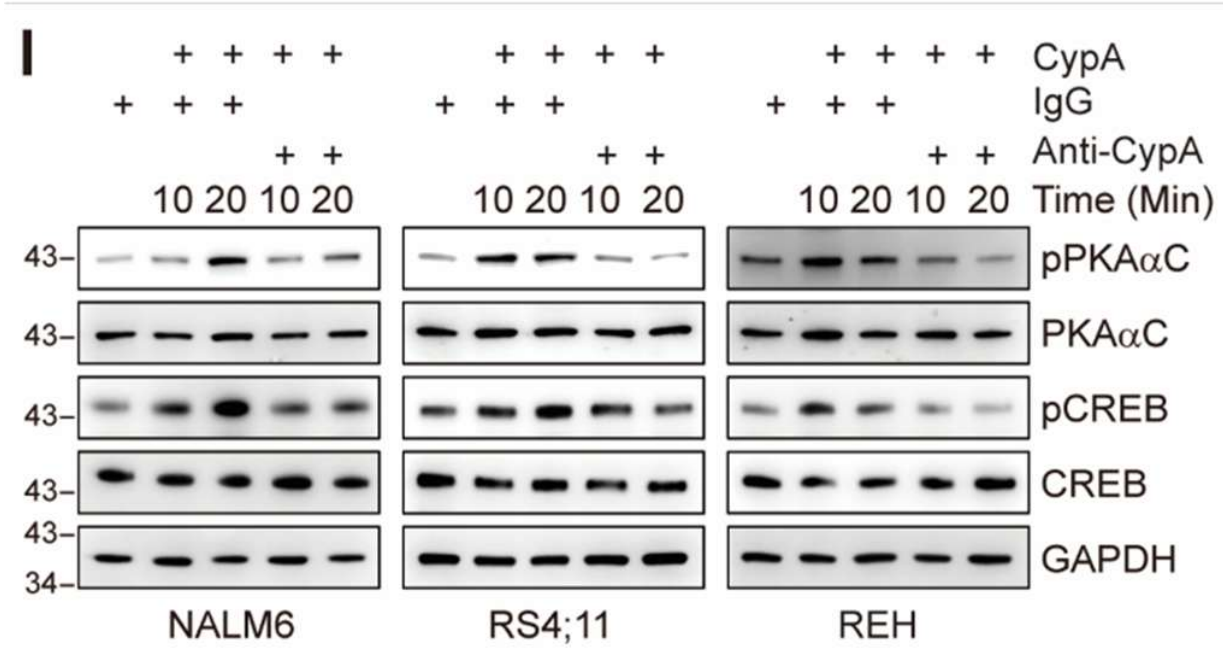

3. Fig.5 reveals some inconsistencies that require explanation or re-experimentation. In subfigures F and G cell viability is measured by substrate metabolism (CCK-8 assay) which is clearly increased upon CypA administration but remains rather constant over time (>0.9=90% viability) under conditions of anti-CypA +/- CypA or MC2R knockout. This finding indicates inhibitory effects on growth/proliferation rather than a significant induction of apoptotic death, which are hard to reconcile with the reduction in BCL-XL and MCL1 in subfigure E. Under VCR treatment, a partial rescue from apoptotic death is noted upon CypA administration that is compensated by anti-CypA (could you provide additional data on anti-CypA alone?). By contrast, in the absence of cytotoxic stress (DMSO control) CypA/anti-CypA did not measurably increase the apoptotic rate of NALM6 cells suggesting that anti-CypA dependent cytotoxic efficiency is generally limited by the anti-apoptotic effect size of CypA induction. In other words, the success of a CypA-directed treatment might depend on a prior challenge of ALL cells with exogenous (therapeutically applied) CypA?

We agree that the CCK-8 assay primarily reflects cell viability/metabolic activity rather than apoptosis. Regarding the apparent discrepancy between changes in BCL-XL/MCL1 expression and the lack of apoptosis under basal conditions, we further confirmed that neither eCypA (**Figure 5K, EV6E, F**) nor anti-CypA mAb alone (**Appendix Figure S3**) significantly altered apoptosis in the absence of cytotoxic stress, suggesting that these molecular changes do not translate into detectable apoptosis under basal conditions. In contrast, under vincristine treatment, eCypA clearly attenuated apoptosis, whereas anti-CypA mAb reversed this protective effect (**Figure 5K and Figure EV6E, F**). These findings suggest that the functional impact of eCypA–MC2R signaling becomes more evident under cytotoxic stress, where regulation of BCL-XL and MCL1 is associated with altered cellular response to apoptotic stimuli. We have revised the manuscript to describe the distinct effects of eCypA under basal conditions and following vincristine treatment (**Page 15**).

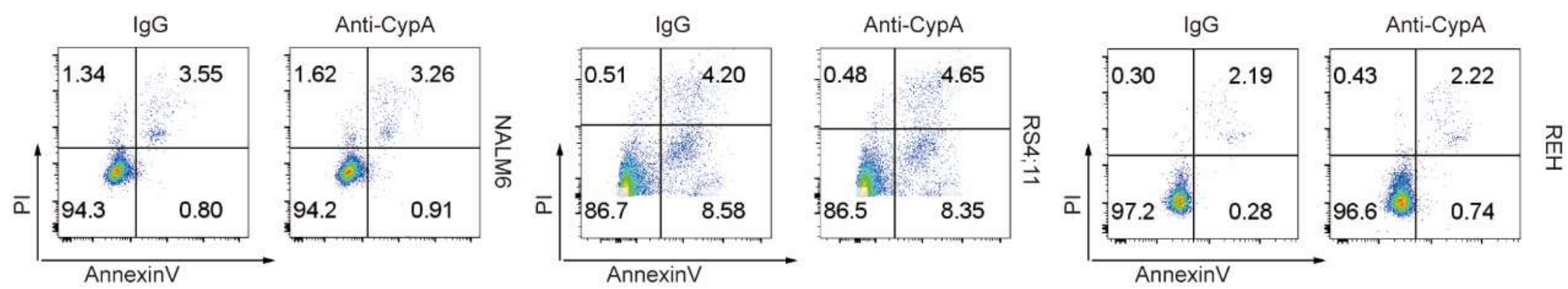

Appendix Figure S3. Effects of anti-CypA monoclonal antibody alone on apoptosis in B-ALL cells. NALM6, RS4;11, and REH cells were treated with IgG control or anti-CypA monoclonal antibody at 10 ng/mL for 36 h, followed by Annexin V/PI staining to assess apoptosis.

4. Fig.6 F-H. It is hard to reconcile the presented bioluminescence data (VDL+IgG vs VDL+anti-CypA) in F with the life tables in H. Tumor burden does not significantly differ on d24 between the two treatment arms, but graphs diverge by d30 when about half of the VDL-CHX cohort and the first two animals of the VDL-CHX + anti-CypA cohort had to be sacrificed indicative of treatment cohort-overlapping events. By d38 all animals in the experiment had died questioning the rigor and robustness of the survival analyses. Did the authors perform a log rank test to analyze survival in H? Could the authors provide the test statistic and exact p-values?

First, we agree that differences in tumor burden between treatment groups are not prominent at earlier time points (e.g., day 24), and that survival events occur within a relatively narrow time window in this aggressive leukemia model. BLI provides a snapshot of tumor burden at discrete time points, whereas survival reflects the cumulative progression of disease. Therefore, modest differences in tumor burden at earlier stages may translate into differences in time-to-endpoint at later stages.

Consistent with this, although total flux did not significantly differ at day 24, divergence between groups became more apparent by day 30, followed by the onset of survival events. The partial temporal overlap of events reflects the aggressive nature of the model. Importantly, all animals were monitored using predefined and consistent humane endpoint criteria, which were applied uniformly across all groups.

Survival curves in **Figure 6H** were analyzed using the log-rank (Mantel–Cox) test, and the corresponding statistical parameters have now been added to the figure legend. A significant difference between groups was observed ($\chi^2 = 12.9$, df = 1, $p = 0.0003$). Consistent results were obtained using the Gehan–Breslow–Wilcoxon test ($\chi^2 = 10.6$, $p = 0.0011$), supporting a difference in survival distributions. For completeness and transparency, pairwise survival comparisons among all four groups are provided in the source data.

We note that, although the difference in median survival is modest (29.5 vs 34.5 days), the statistical significance reflects a shift in the overall survival distribution rather than a single time point. Given the aggressive disease course and limited survival window, we have revised the manuscript to more cautiously describe the treatment effect as a delay in disease progression, rather than a large survival benefit (**Page 17**).

5. Fig.7 H,I: Marginal survival differences between PDX-ALL treatment, but highly significant p-values are given. Log rank test? Test statistic?
J: Annotation of ALL subclusters in scRNA-seq UMAP is unusual. Authors differentiate between Pre-B, cycling pre-B, and MLL-R cells. ScRNA seq does allow for cell cycle phase specific annotation (early G1/GO-S-G2/M) which should be performed thoroughly for all clusters. It is unclear how the MLL-R cluster was determined. The heatmap in K suggests the annotation is simply based on KMT2A, AFF1, MEF2C etc. single cell mRNA reads. To authenticate their claim of an MLL-R subclone, the authors should provide genomic/transcriptomic/RT-PCR data on a KMT2A::AFF1 fusion that would be associated with a transactivated HOX7-10 (in particular HOX9), MEIS1 and others which are not presented. KMT2A and AFF1 mRNA are not necessarily upregulated in MLL-r. Maybe the authors rather identified a quiescent subclone with stem cell like features that resembles MLL-r ALL?

The survival analyses in **Figure 7** were analyzed using the log-rank (Mantel–Cox) test, and the corresponding test statistics and exact p values have been added to the figure legend and provided in the source data. Although the difference in median survival appears modest, the statistical significance reflects the overall separation of survival curves across the entire follow-up period, rather than median values alone. To avoid overinterpretation, we have revised the text to more cautiously describe the effect of anti-CypA mAb.

We agree that our initial annotation using terms such as “pre-B” and “MLL-like” was not sufficiently rigorous. In particular, these leukemic populations could not be reliably assigned to canonical B-cell developmental stages based on transcriptional profiles alone, as shown in **Appendix Figure S8a**, where classical B-lineage markers (e.g., DNTT, VPREB1, RAG1/2) are broadly expressed across clusters without defining discrete developmental stages. Therefore, we have removed the use of “pre-B” or related terminology to avoid overinterpretation.

Regarding the “MLL-like” annotation, we would like to clarify that the corresponding patient sample used to generate the PDX model harbors a KMT2A::AFF1 fusion, as identified by clinical bulk transcriptomic (RNA-seq) analysis at diagnosis. However, we acknowledge that standard droplet-based scRNA-seq data do not allow reliable detection of gene fusions, such as KMT2A rearrangements, due to limited transcript coverage and lack of breakpoint-spanning reads. Therefore, fusion status cannot be inferred from our single-cell dataset. Based on this limitation, and to avoid conflating genomic alterations with transcriptional states, we have removed the “MLL-like” designation in the revised manuscript. Instead, guided by the reviewer’s suggestion, we adopted a more conservative, state-based annotation strategy. Differential expression analysis revealed that a subset of cells (State 2) exhibited increased expression of genes previously reported to be enriched in immature or progenitor-like hematopoietic and leukemic states, including FLT3, MSI2, MEF2C, and RUNX1 (**Appendix Figure S5B**). We emphasize that these genes are used to describe relative transcriptional features rather than to define a specific progenitor cell identity. Accordingly, we refer to these

populations as "State 1" and "State 2" without assigning predefined developmental or genetic labels (**Figure 7H and I**).

In addition, in response to the reviewer's comment regarding cell cycle annotation, we performed systematic cell cycle analysis across all clusters. Based on the enrichment of cell cycle–associated gene signatures, we classified cells into cycling and non-cycling populations, resulting in four groups (State 1, State 2, Cycling State 1, and Cycling State 2). The results are presented in **Figure EV7E** and are also visualized in the updated UMAP representation.

Finally, we have revised the manuscript throughout to clarify that the identified clusters represent transcriptionally defined cell states rather than genetically defined subclones, and to ensure consistent and accurate terminology.

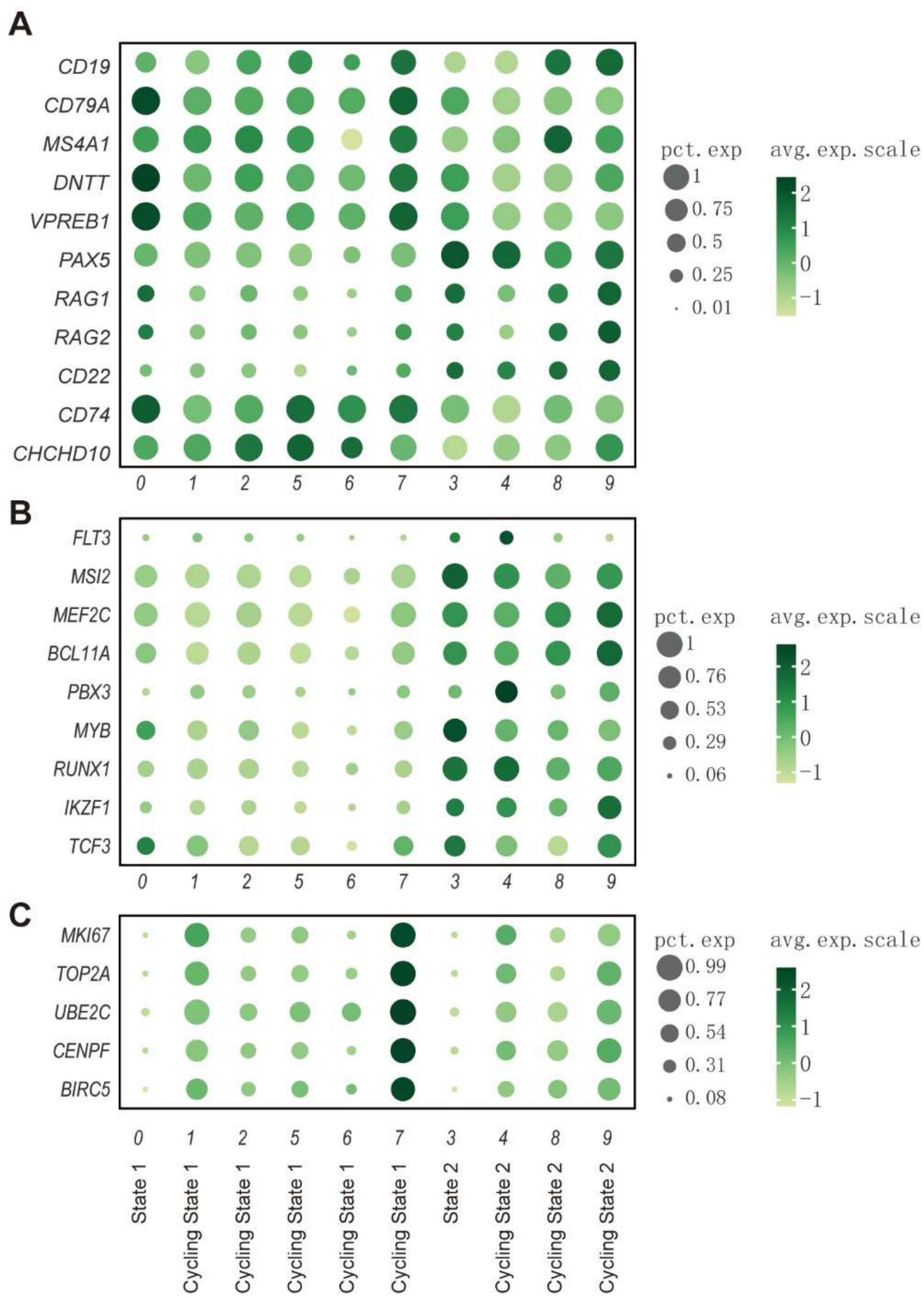

Appendix Figure S5. Transcriptional features underlying cell state annotation in scRNA-seq analysis. (A) Dot plot showing the expression of B-lineage–associated genes across the identified cell states. Canonical B-cell markers were broadly and consistently expressed across all states, supporting a homogeneous B-lineage identity. (B) Dot plot showing the expression of genes associated with progenitor-related transcriptional programs (FLT3, MYB, MSI2, RUNX1, PBX3, BCL11A, and MEF2C) across the defined states, highlighting their enrichment in State 2. (C) Dot plot showing the expression of cell cycle–related genes (MKI67, TOP2A, UBE2C, CENPF, and BIRC5), indicating proliferative activity and supporting the identification of Cycling State 1 and Cycling State 2 subpopulations.

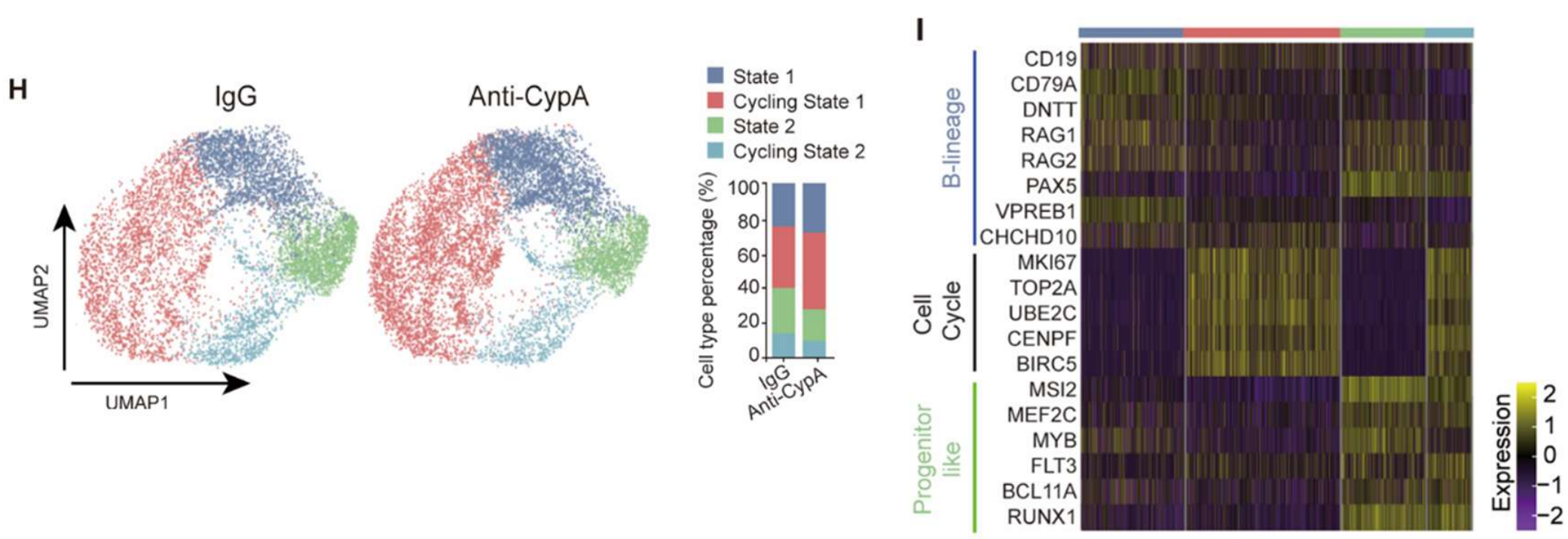

Figure7. (H) UMAP plots (left) and corresponding bar plot (right) showing the distribution of cell states in IgG- and Anti-CypA–treated PDX-ALL samples. (I) Heatmap showing the expression of representative marker genes used for state annotation, including genes associated with B-lineage programs, cell cycle activity, and progenitor-like transcriptional programs.

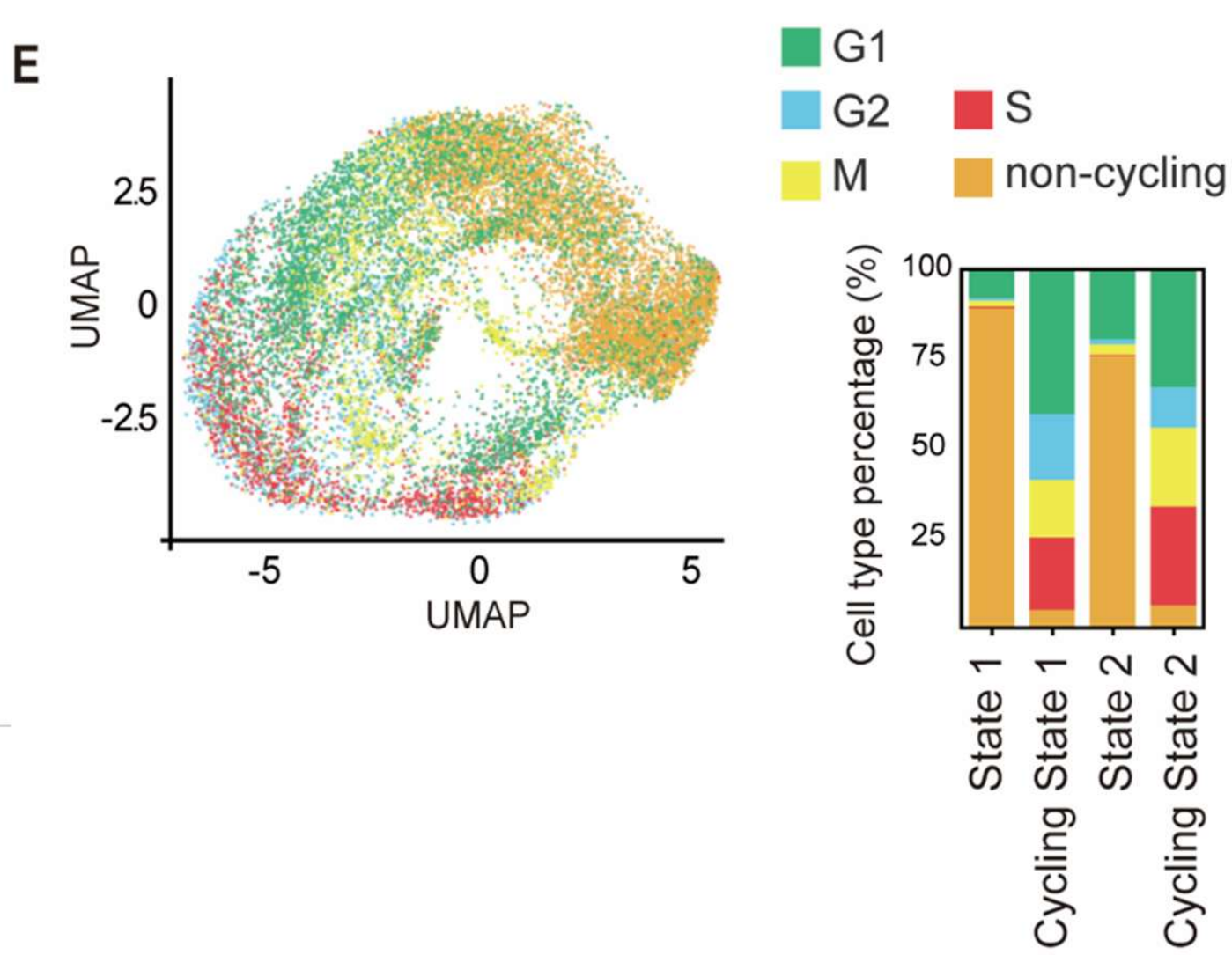

Figure EV7. (E) UCell-based scoring of cell cycle gene signatures, classifying cells into non-cycling, G1, S, G2, and M phases. UMAP visualization of cell cycle states (right) and bar plot showing the distribution of cell cycle phases across different cell clusters (left).

Minor points:

6. While the title of the manuscript is suitable, the 'in brief' synopsis contains claims that are not sufficiently backed by experimental data such that blocking CypA 'suppresses' ALL progression. Clearly, it does delay progression (by several days) rather than suppress ALL progression (Fig.6F,H and Fig.7H,I).

As suggested, we have replaced "suppresses" with "delays" in the revised manuscript.

7. Physical properties, source and binding specificity (mouse-human cross-reactivity?) should be briefly described. Did the authors look at MRAP as accessory heterodimeric binding partner that modulates ACTH-MC2R downstream signaling? Authors only refer to a previous publication from the same group, in which anti-CypA was applied in the context of viral pneumonia (Bai et al. 2024).

To distinguish human-derived and mouse-derived CypA, species-specific ELISA kits were used. Human CypA levels were quantified using a human-specific ELISA kit (CSB-E09920h, Cusabio), while mouse CypA levels were measured using a mouse-specific ELISA kit (CSB-E09282m, Cusabio). According to the manufacturer's specifications, these kits are designed to selectively detect their respective species and do not exhibit cross-reactivity.

We further examined this issue experimentally using the same set of peripheral blood plasma samples from mice and humans. As shown in newly added Appendix **Figure S1**, when mouse and human plasma samples were measured using the mouse eCypA ELISA kit and the human eCypA ELISA kit, respectively, no cross-reactivity was detected between the two assays.

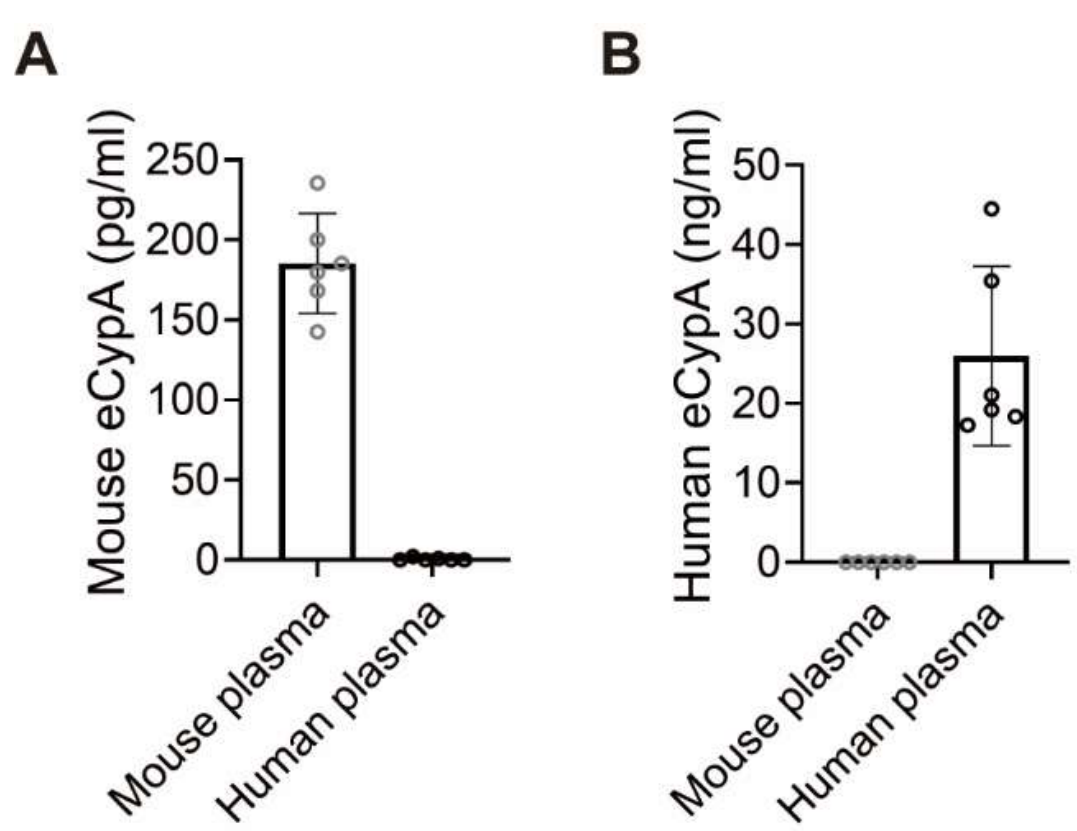

Appendix Figure S1. Cross-reactivity validation of the eCypA ELISA kits between human and mouse. (A–B) The same set of peripheral blood plasma samples from mice

and humans were measured using the mouse eCypA ELISA kit (A) and the human eCypA ELISA kit (B), respectively (n = 6).

In addition, the antibody exhibits cross-reactivity with both human and mouse CypA, as demonstrated by detection in human leukemia cells and mouse-derived samples (**Appendix Figure S6**). In addition, previous surface plasmon resonance analysis showed high-affinity binding to recombinant human CypA (KD ≈ 0.295 nM) (Bai et al., 2024) .

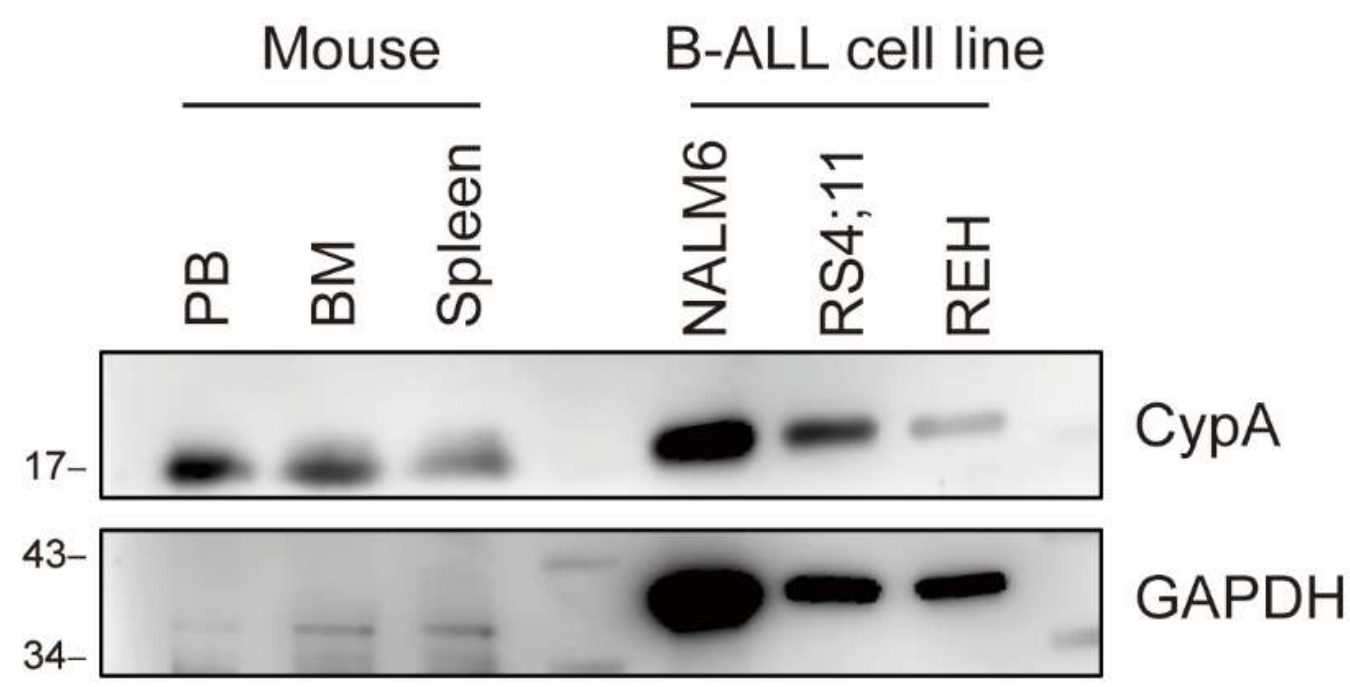

Appendix Figure S6. Validation of CypA interaction in human leukemic cells and murine hematopoietic tissues. Red blood cells were lysed from mouse peripheral blood (PB), bone marrow (BM), and spleen prior to protein extraction. Whole-cell lysates from these murine tissues, together with human B-ALL cell lines (NALM6, RS4;11, and REH), were subjected to Western blot analysis to detect CypA. GAPDH was used as a loading control.

In our proteomic analysis, both MC2R and MRAP2 were identified among the CypA-associated proteins, particularly in RS4;11 cells (**Table EV4**), which further supports the biological relevance of MC2R in this system. In preliminary experiments using 293T cells, we co-expressed MRAP2 together with MC2R and observed that MRAP2 enhanced eCypA-induced cAMP production. This is consistent with previous reports that MRAP proteins facilitate MC2R trafficking and signaling. Importantly, we further confirmed that MC2R expression alone was sufficient to mediate cAMP production upon eCypA stimulation (**Figure 3E**), indicating that MRAP2 is not strictly required for receptor activation in this context but rather functions as a positive modulator that enhances signaling efficiency. Given that the primary aim of this study was to establish MC2R as a functional receptor mediating eCypA-induced signaling and downstream effects in ALL cells, we focused our mechanistic investigation on the MC2R-dependent axis. While MRAP2 likely contributes to optimizing MC2R membrane localization and signaling output, its specific role was not further dissected in this study and warrants future investigation.

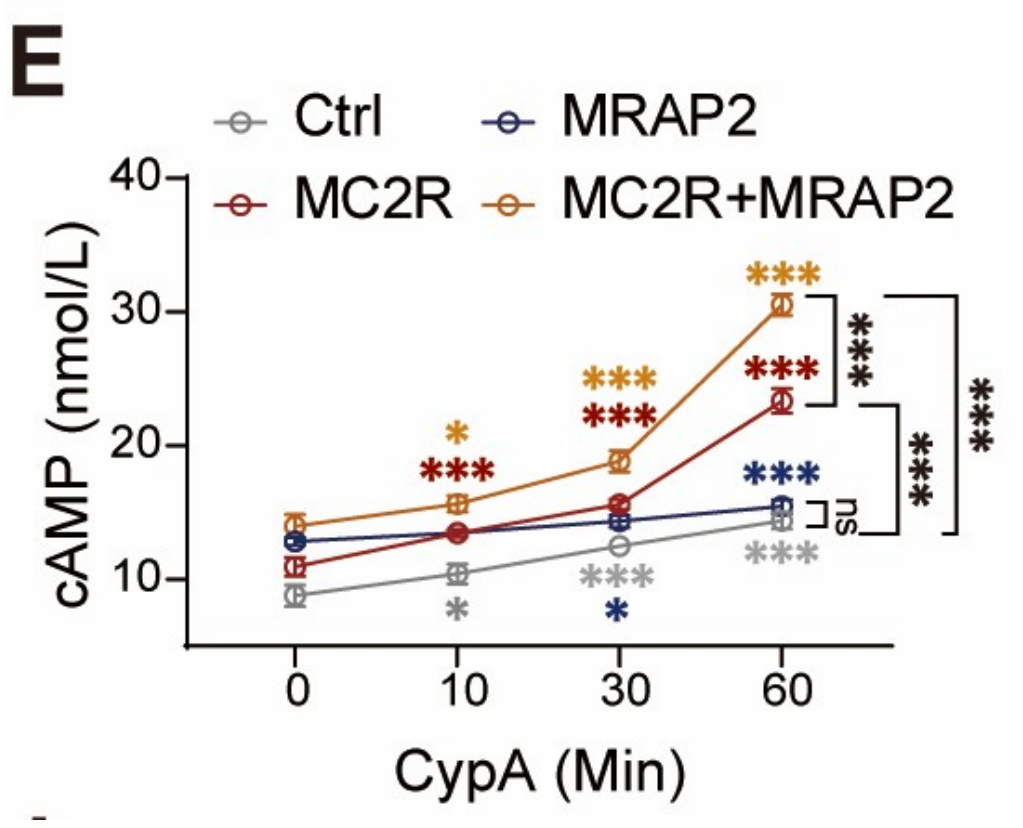

Figure 3. (E) 293T cells were transfected with Flag-Vec, Flag-MC2R, Flag-MRAP2, or co-transfected with Flag-MC2R and Flag-MRAP2 for 36 h, followed by treatment with eCypA for the indicated time points. Intracellular cAMP levels were then measured (n = 3 independent biological replicates).

8. All figure legends lack sufficient information on the number of independent experiments and biological vs technical replicates.

We have revised all figure legends to explicitly include the number of independent experiments and to clearly distinguish biological and technical replicates.

9. Comprehensive mass spec data on CypA binding in ALL lines should be made accessible to assess MC2R binding specificity and to comply with data availability policies. CypA has been shown to bind other receptors such as CD147 and integrin beta 2, the latter of which is expressed Jurkat T-ALL cells according to the authors' own data (Bai et al. 2024).

The mass spectrometry proteomics data have been deposited to the ProteomeXchange Consortium via the iProX partner repository with the dataset identifier PXD076421. The dataset can be accessed for peer review using the following URL: https://www.iprox.cn/page/PSV023.html;?url=1775632113131QGpU (password: fWjP).

We agree that CypA has been reported to interact with multiple receptors, including CD147 and integrin β2. Our data support MC2R as a novel functional receptor in B-ALL cells.

10. p12 line 199: Specify PKA and CREB inhibitors in Mat & Meth.

We have specified the PKA and CREB inhibitors in the Methods section. All reagents, antibodies, and experimental materials used in this study, have also been comprehensively provided in the submitted **Reagents and Tools Table**.

11. p14: line 238 (refers to Fig. S5F). PCR-based expression analysis of BCL-XL (species=human) is likely skewed, because authors used non-defined PBMCs (leukemic blasts? Normal lymphocytes?) from ALL vs healthy donors (mononuclear cells= heterogeneous population).

We agree that PBMCs represent a heterogeneous population, which may include both leukemic blasts and normal immune cells in patients with ALL, as well as diverse immune cell subsets in healthy donors. Therefore, differences in cellular composition could potentially influence the measured gene expression levels. In this study, PBMC-based qPCR analysis was included as a descriptive observation of circulating samples rather than as definitive evidence of cell-intrinsic regulation. We have therefore revised the text to clarify this point and have toned down the interpretation accordingly. Specifically, these data are now described as being directionally consistent with the *in vitro* findings in NALM6 and RS4;11 cells, rather than serving as direct mechanistic evidence (**Page 15**).

12. p14 line 247-248: MRD has not been described in the paper: TCR/IGH PCR MRD or FACS MRD? Define MRD time points (postinduction or postconsolidation?) and levels for MRD negativity vs positivity.

We have added a clear definition of MRD in the revised manuscript. MRD status was determined by flow cytometry analysis of bone marrow samples at the end of induction therapy (day 28–30), and patients were classified as MRD-positive or MRD-negative based on routine clinical assessment of residual leukemic cells. In addition, we have included a supplementary table summarizing the bone marrow blast percentages at diagnosis, day 14, and day 28–30 for each patient (**Table EV1**). The MRD classification used in this study is based on the day 28–30 assessment, while the day 14 time point was used to evaluate early treatment response.

Serum eCypA levels were measured in peripheral blood plasma samples collected at day 14 during induction therapy, and their association with the final MRD status was analyzed. The manuscript has been revised accordingly to clearly define MRD and describe the timing of sample collection (**Page 29**).

13. p16 lines 287-290 and p18 lines 330-331: It is recommended to use more cautious wording when describing and interpreting anti-CypA-mAb treatment effects. Life tables in Fig. 7 speak their own language with minimal delay in disease progression and survival. Findings herein suggest that eCypA contributes to ALL progression, but this referee doubts that it acts as a critical driver of ALL pathogenesis questioning its potential as a therapeutic target.

We thank the reviewer for this thoughtful and important comment. We agree that the survival benefit observed in our models is modest. We have revised the manuscript to adopt more cautious wording when describing both the biological role of eCypA and the effects of anti-CypA mAb treatment. (Line 352) (**Page 18 and 20**)

14. p16 line 299 and p19 lines 347-349: The authors describe an MLL-rearranged (MLL-R) subset based on sc-RNA-seq data from PDX-ALL models. Did the authors detect a genomic MLL rearrangement by FISH or breakpoint specific (RT-)PCR or do they infer the MLL-R status from the expression of sc mRNA reads (KMTA2 and AFF1 expression)? Please clarify if you refer to an authentic MLL-rearrangement or an MLL-

R like transcriptional phenotype.
The cited papers (Krivtsov et al. 2019; Lopez-Millan et al. 2024) refer to new drug developments for MLL-R rather than original reports on CHX-resistance or relapse (menin inhibitor and NG2/FLT3 PPI inhibitor, respectively). The authors should cite the original work on MLL-R.

Regarding the annotation of an "MLL-rearranged (MLL-R)" subset, we acknowledge that our original description was not sufficiently precise and may have been misleading. The PDX-LB12 model used in this study was derived from a patient sample harboring a KMT2A::AFF1 fusion, as identified by clinical bulk transcriptomic analysis at diagnosis. Detailed molecular information for both PDX models is now provided in **Table EV5**, and only PDX-LB12 was subjected to single-cell RNA sequencing in this study. However, we did not perform FISH or breakpoint-specific RT-PCR validation within the scope of this work. Importantly, we recognize that standard droplet-based scRNA-seq data do not allow reliable detection of gene fusions, such as KMT2A rearrangements, due to limited transcript coverage and the lack of breakpoint-spanning reads. Therefore, the MLL-R status cannot be inferred from our single-cell dataset, nor can it be concluded from the expression levels of KMT2A, AFF1, or related genes. Based on this limitation, and to avoid conflating genomic alterations with transcriptional features, we have removed the "MLL-R" or "MLL-like" terminology throughout the revised manuscript. Accordingly, no claim of an authentic MLL-rearranged subclone is made in the revised version. Instead, we now describe these populations using a neutral, state-based annotation (State 1 and State 2), reflecting transcriptional heterogeneity rather than genetically defined subtypes.

We agree with the reviewer that the cited references (Krivtsov et al. 2019; Lopez-Millan et al. 2024) were not appropriate in this context. As we have removed the MLL-R-related interpretation, these references have been deleted from the revised manuscript, and the text has been revised accordingly to avoid unsupported associations.

15. P22 line 405: Information on CREB and PKA inhibitors is not sufficient.

We have specified the PKA and CREB inhibitors in the Methods section (**Reagents and Tools Table**).

16. P22 line 418: Describe physico-chemical details of CypA-mAb (cross-reactivity/ species/ mouse humanized?)

The anti-CypA antibody is a mouse monoclonal antibody generated against recombinant human CypA and purified using Protein A affinity chromatography. This antibody exhibits cross-reactivity with both human and mouse CypA, as demonstrated by consistent detection in human leukemia cells and mouse-derived tissues, including peripheral blood, bone marrow, and spleen (**Appendix Figure S6**). In addition, previous surface plasmon resonance analysis demonstrated high-affinity binding of this antibody to recombinant human CypA (KD ≈ 0.295 nM) (Bai et al., 2024). Detailed physicochemical and validation information for the anti-CypA monoclonal antibody

has been added to the revised manuscript (**Page 25**).

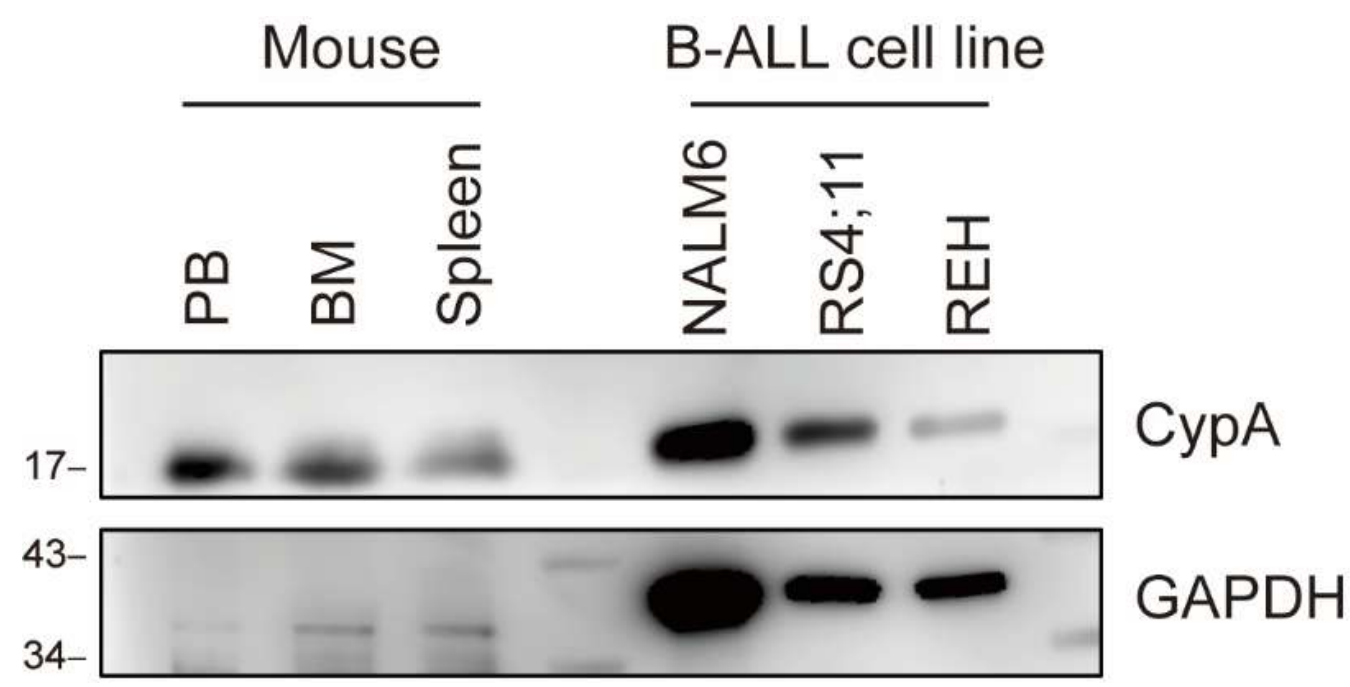

Appendix Figure S6. Validation of CypA interaction in human leukemic cells and murine hematopoietic tissues. Red blood cells were lysed from mouse peripheral blood (PB), bone marrow (BM), and spleen prior to protein extraction. Whole-cell lysates from these murine tissues, together with human B-ALL cell lines (NALM6, RS4;11, and REH), were subjected to Western blot analysis to detect CypA. GAPDH was used as a loading control.

17. P23 lines 436-438: Immunological and genetic characterization of PDX-ALL models is missing.

We have added a comprehensive description of the immunological and genetic characteristics of the PDX-ALL models in the revised manuscript.

First, the genetic features of both PDX models have been clarified. PDX-LB12 harbors a *KMT2A::AFF1* (MLL-rearranged) fusion together with an *FLT3-ITD* mutation, whereas PDX-QB4 is a fusion-negative case carrying an *FLT3 Y572C* mutation. These data were derived from RNA-seq–based transcriptomic analysis and are summarized in **Table EV5**.

Second, the immunophenotypic characterization has been added based on clinical diagnostic flow cytometry reports of the original patient bone marrow samples used for PDX establishment. Both samples exhibit a B-lineage phenotype with high expression of CD19 and CD22, together with precursor-associated markers including CD34 and HLA-DR. Additional markers (e.g., CD10, CD20, CD38, CD79a, CD13, and CD33) are summarized in **Table EV5**.

To further support these data, we have included representative flow cytometry plots in the revised manuscript (**Appendix Figure EV7**), showing key immunophenotypic features. Due to the retrospective nature of sample acquisition, raw FCS files were not available; therefore, representative clinical report images are provided.

Finally, we have clarified in the Methods section that both PDX models were generated from diagnostic bone marrow samples collected at initial presentation, ensuring consistency between the clinical immunophenotype and the xenograft models.

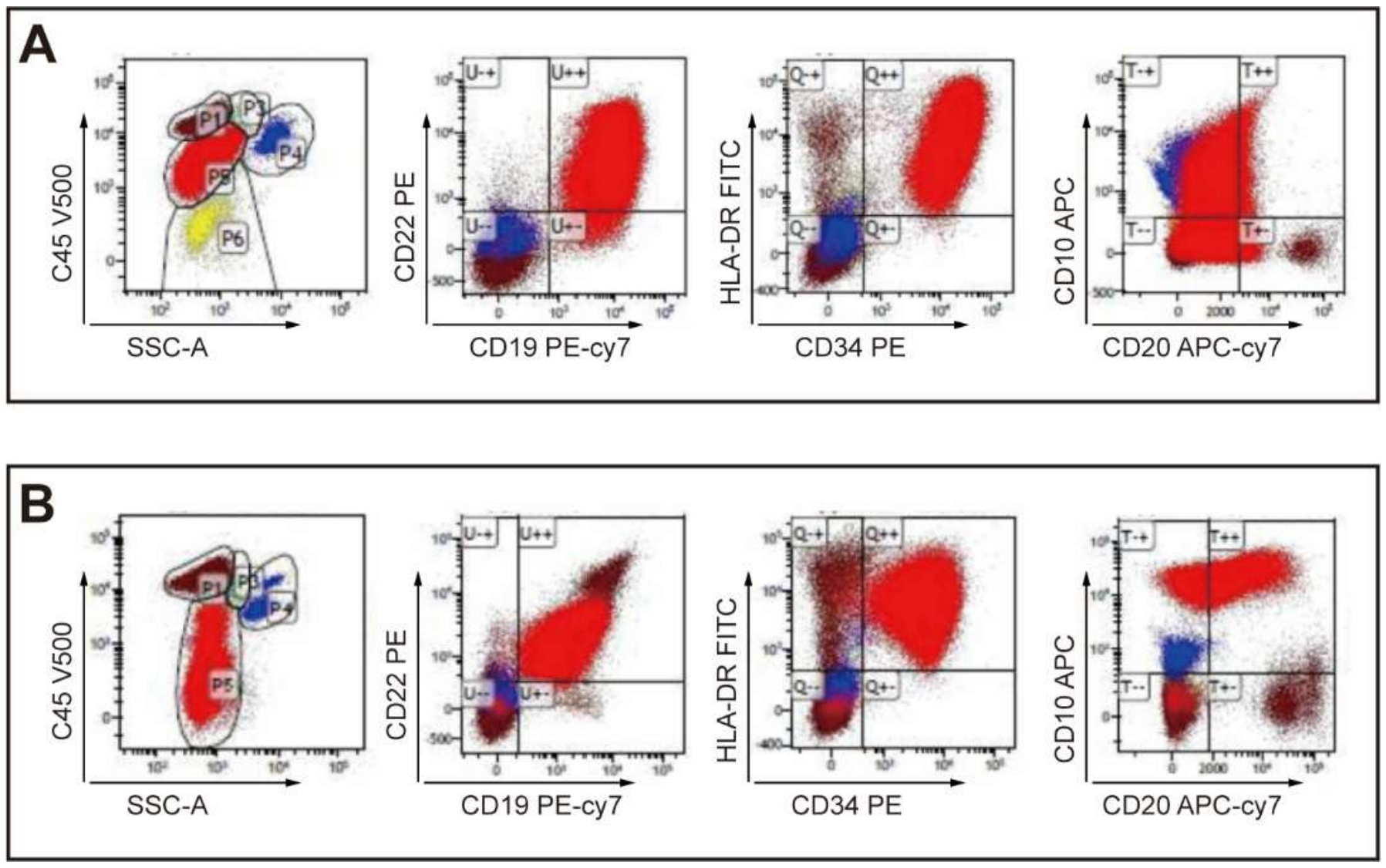

Appendix Figure S7. Representative clinical flow cytometry analysis of donor samples used to establish PDX-ALL models. (A) PDX-LB12 and (B) PDX-QB4 donor samples. Flow cytometry data were obtained from diagnostic bone marrow samples at initial presentation, which were subsequently cryopreserved and used to establish the corresponding PDX models. Leukemic blast populations were identified in the CD45dim/SSC low region (P5 gate), and subsequent analyses were performed on gated blast cells. Both samples show strong expression of B-lineage markers (CD19 and CD22). PDX-LB12 exhibits a more immature immunophenotype, characterized by high CD34 and HLA-DR expression, with relatively low CD10 and CD20 levels. In contrast, PDX-QB4 displays a more differentiated pre-B phenotype with high CD10 and CD20 expression. Comprehensive immunophenotypic data derived from clinical diagnostic flow cytometry reports are summarized in **Table EV5**.

18. P24: Basic mass spec experimentation details should be given. Pre-analytics, instrument etc.

We have added detailed mass spectrometry experimental procedures, including sample preparation, instrument platform, and data processing parameters, in the Methods section (**Page 33**).

19. Fig.1E: Citation of GDC target-ALL-P2 is confusing, because the authors refer to an ALL genomics paper by the US-American SJCRH and COG groups that deposited data in the TARGET data base GDC target-ALL-P2 (Brady et al. 2022 Nat Genet). A more appropriate reference would be the TARGET data base. See also above point 1.

We agree that the previous citation of the TARGET ALL dataset was not appropriate in the context of our survival analysis. The Kaplan–Meier survival analysis shown in **Figure 1E** has therefore been removed due to limitations associated with the use and interpretation of the visualization platform.

Following the reviewer's suggestion in point 1, we have re-analyzed the data using a Cox proportional hazards model based on the TARGET ALL cohort obtained from the GDC data portal. The revised analysis and corresponding results have been updated accordingly in the manuscript.

20. Fig.4D: IB is of poor quality. Blots are overexposed and FN1 as well as MMP9 signals are hard to decipher.

We have repeated this experiment and replaced the original images with newly generated data of improved quality. The MMP9 antibody (abcam, ab283575) has been replaced to enhance signal specificity and clarity.

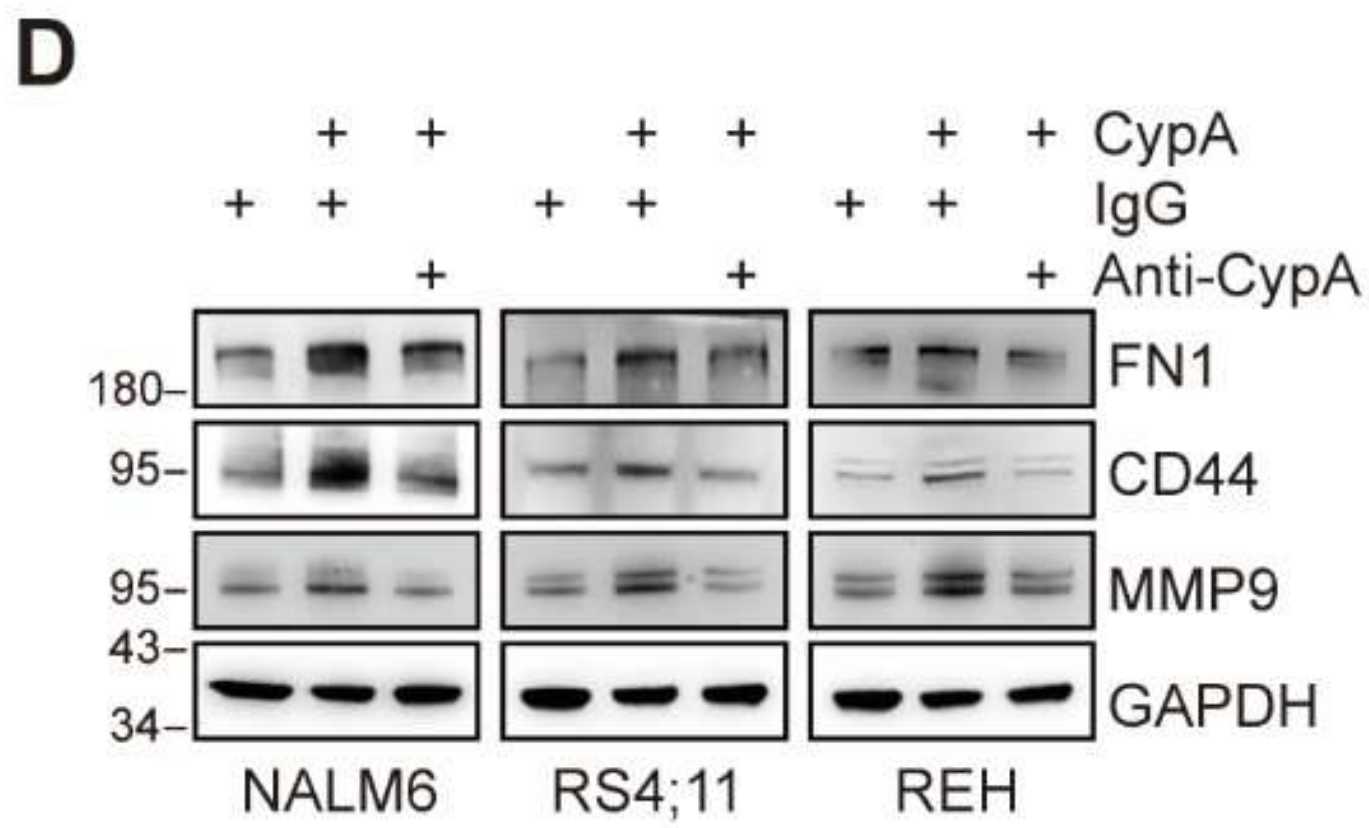

Figure 4. (D) Western blot analysis of FN1, CD44, and MMP9 expression in NALM6, RS4;11, and REH cells treated with IgG, IgG+eCypA, or Anti-CypA+eCypA for 24 h. Representative images from at least three independent biological experiments are shown.

21. Fig. 6A: Provide information on MRD analyses in the figure legend: PCR/flow (?) postinduction time point etc.? Legend Fig.6 (C): Typo NSG instead of NCG?

We have revised the figure legend of **Figure 6A–B** to clearly specify that MRD status was determined by flow cytometry analysis of bone marrow samples at the end of induction therapy (day 28–30), based on standard clinical criteria. We have also clarified that peripheral blood plasma samples used for eCypA measurement were collected at day 14 during induction therapy.

In addition, we have provided a detailed description of MRD assessment criteria in the Methods section (**Page 29**) and included a supplementary table summarizing MRD-related clinical data (**Table EV1**).

Furthermore, the description of the in vivo experiment in **Figure 6C** has been refined for clarity.

22. D,E: Please describe properties of anti-CypA in more detail in the main text or supplement. Binding affinity and species cross-reactivity etc.

We have described properties of anti-CypA in more detail in the Methods section (**Page 25**).

23. Fig.7 N: Heatmap of TF activities could be improved by use of complementary colors (+ - color bar).

We thank the reviewer for this helpful suggestion. The heatmap has been revised using a complementary color scale to improve contrast and visualization of positive and negative transcription factor activity. The original **Figure 7N** is now presented as **Figure 7L** in the revised manuscript.

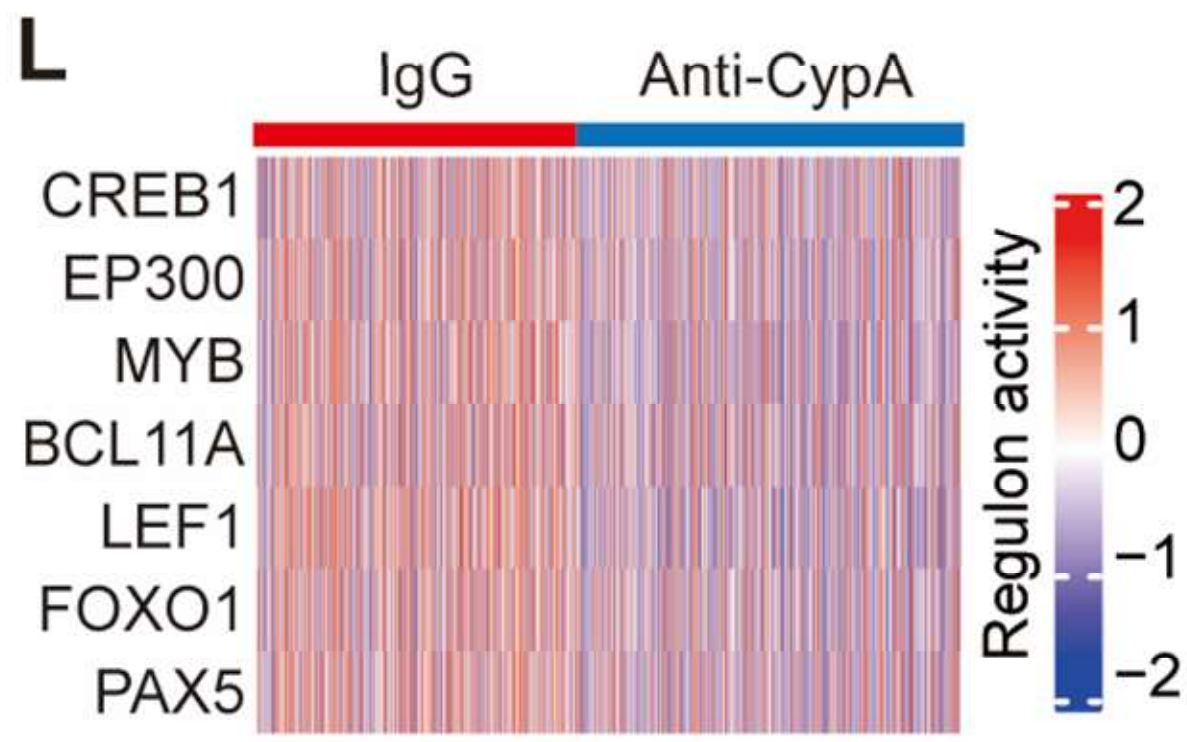

References:

Bai X, Yang W, Zhao Y, Cao T, Lin R, Jiao P, Li H, Li H, Min J, Jia X, Zhang H, Fan W, Jia X, Bi Y, Liu W, Sun L (2024) The extracellular cyclophilin A-integrin beta2 complex as a therapeutic target of viral pneumonia. *Mol Ther* 32: 1510-1525

Bendall LJ, Nilsson SK, Khan NI, James A, Bonnet C, Lock RB, Papa R, Bradstock KF, Gottlieb DJ (2004) Role of CD44 variant exon 6 in acute lymphoblastic leukaemia: association with altered bone marrow localisation and increased tumour burden. *Leukemia* 18: 1308-11

Chan LF, Webb TR, Chung TT, Meimaridou E, Cooray SN, Guasti L, Chapple JP, Egertova M, Elphick MR, Cheetham ME, Metherell LA, Clark AJ (2009) MRAP and MRAP2 are bidirectional regulators of the melanocortin receptor family. *Proc Natl Acad Sci U S A* 106: 6146-51

Gutjahr JC, Bayer E, Yu X, Laufer JM, Hopner JP, Tesanovic S, Harzschel A, Auer G, Riess T, Salmhofer A, Szenes E, Haslauer T, Durand-Onayli V, Ramspacher A, Pennisi SP, Artinger M, Zaborsky N, Chigaev A, Aberger F, Neureiter D et al. (2021) CD44 engagement enhances acute myeloid leukemia cell adhesion to the bone marrow microenvironment by increasing VLA-4 avidity. *Haematologica* 106: 2102-2113

Ji KY, Kim SM, Yee SM, Kim MJ, Ban YJ, Kim EM, Lee EH, Choi HR, Yun H, Lee CW, Yun CH, Lee CJ, Lee HB, Kang HS (2021) Cyclophilin A is an endogenous ligand for the triggering receptor expressed on myeloid cells-2 (TREM2). *FASEB J* 35: e21479

Jin ZG, Lungu AO, Xie L, Wang M, Wong C, Berk BC (2004) Cyclophilin A is a proinflammatory cytokine that activates endothelial cells. *Arterioscler Thromb Vasc Biol* 24: 1186-91

Kim H, Kim WJ, Jeon ST, Koh EM, Cha HS, Ahn KS, Lee WH (2005) Cyclophilin A may contribute to the inflammatory processes in rheumatoid arthritis through induction of matrix degrading enzymes and inflammatory cytokines from macrophages. *Clin Immunol* 116: 217-24

Kim J, Kim J, Choi C, Bae J, Choi HJ (2025) Structural insights into GPCR signaling activated by peptide ligands: from molecular mechanism to therapeutic application. *Exp Mol Med* 57: 1467-1481

Longstreth JH, Wang K (2024) The role of fibronectin in mediating cell migration. *Am J Physiol Cell Physiol* 326: C1212-C1225

Peeters MC, van Westen GJ, Li Q, AP IJ (2011) Importance of the extracellular loops in G protein-coupled receptors for ligand recognition and receptor activation. *Trends Pharmacol Sci* 32: 35-42

Rosenbaum DM, Rasmussen SG, Kobilka BK (2009) The structure and function of G-protein-coupled receptors. *Nature* 459: 356-63

Satoh K, Matoba T, Suzuki J, O'Dell MR, Nigro P, Cui Z, Mohan A, Pan S, Li L, Jin ZG, Yan C, Abe J, Berk BC (2008) Cyclophilin A mediates vascular remodeling by promoting inflammation and vascular smooth muscle cell proliferation. *Circulation* 117: 3088-98

Seizer P, von Ungern-Sternberg SNI, Haug V, Dicenta V, Rosa A, Butt E, Nothel M, Rohlfing AK, Sigle M, Nawroth PP, Nussbaum C, Sperandio M, Kusch C, Meub M, Sauer M, Munzer P, Bieber K, Stanger A, Mack AF, Huber R et al. (2024) Cyclophilin A is a ligand for RAGE in thrombo-inflammation. *Cardiovasc Res* 120: 385-402

Seko Y, Fujimura T, Taka H, Mineki R, Murayama K, Nagai R (2004) Hypoxia followed by reoxygenation induces secretion of cyclophilin A from cultured rat cardiac myocytes. *Biochem Biophys Res Commun* 317: 162-8

Sigle M, Rohlfing AK, Cruz Santos M, Kopp T, Krutzke K, Gidlund V, Kollotzek F, Marzi J, von Ungern-Sternberg S, Poso A, Heikenwalder M, Schenke-Layland K, Seizer P, Mollmann J, Marx N, Feil R, Feil S, Lukowski R, Borst O, Schaffer TE et al. (2024) Targeting Cyclophilin A in the Cardiac Microenvironment Preserves Heart Function and Structure in Failing Hearts. *Circ Res* 135: 758-773

Suzuki J, Jin ZG, Meoli DF, Matoba T, Berk BC (2006) Cyclophilin A is secreted by a vesicular pathway in vascular smooth muscle cells. *Circ Res* 98: 811-7

Verma D, Zanetti C, Godavarthy PS, Kumar R, Minciacchi VR, Pfeiffer J, Metzler M, Lefort S, Maguer-Satta V, Nicolini FE, Burroni B, Fontenay M, Krause DS (2020) Bone marrow niche-derived extracellular matrix-degrading enzymes influence the progression of B-cell acute lymphoblastic leukemia. *Leukemia* 34: 1540-1552

Wang L, Wang CH, Jia JF, Ma XK, Li Y, Zhu HB, Tang H, Chen ZN, Zhu P (2010) Contribution of cyclophilin A to the regulation of inflammatory processes in rheumatoid arthritis. *J Clin Immunol* 30: 24-33

Wu S, Tan Y, Li F, Han Y, Zhang S, Lin X (2024) CD44: a cancer stem cell marker and therapeutic target in leukemia treatment. *Front Immunol* 15: 1354992

Yang W, Bai X, Jia X, Li H, Min J, Li H, Zhang H, Zhou J, Zhao Y, Liu W, Xin H, Sun L (2024) The binding of extracellular cyclophilin A to ACE2 and CD147 triggers psoriasis-like inflammation. *J Autoimmun* 148: 103293

Zhao Y, Jiao P, Bai X, Li H, Pei HZ, Lin R, Zhang H, Fan Z, Jia X, Luo T, Bi Y, Liu Y, Yan X, Duan H, Liu W, Sun L (2026) Extracellular cyclophilin a binding to CD146 is critical for Th17 cell differentiation in rheumatoid arthritis. *Mol Ther* 34: 1258-1276

Zheng T, Huang J, Xiang X, Li S, Yu J, Qu K, Xu Z, Han P, Dong Z, Liu Y, Xu F, Yang H, Jaattela M, Luo Y, Liu B (2021) Systematical analysis reveals a strong cancer relevance of CREB1-regulated genes. *Cancer Cell Int* 21: 530

20th May 2026

Dear Dr. Sun,

Thank you for submitting your revised manuscript to EMBO Molecular Medicine. Your manuscript will be ready for acceptance pending the following final amendments:

1) Please implement referees' suggestions.
2) In the main manuscript file, please do the following:
- Please address all comments suggested by our data editors listed below:
o Data availability statement:
1. Please note that the exact p values are not provided in the legends of figures 1A, B, C, F, G, I, J, K, L, M, N; 2B, C, D, E, G, H; 3E, F, G, H, L, M, N; 4C, E, F, H, J, K, L; 5A, C, D, F, H, I, J, K, 6D, E, G, H; 7B,C, D, E; EV1 A-E; EV2 A, B; EV3 D, G, I, J, M; EV5 A-E; EV6 A-G.
2. Please indicate the statistical test used for data analysis in the legends of figures EV4 B, C.
3. Please note that information related to n is missing in the legends of figures 2D, E; EV4 B, C.
4. Please note that the black and white arrows are not defined in the legend of figure EV7 C, D. This needs to be rectified.
- Please update the order and the headings of the sections in the manuscript text to: Abstract / The Paper Explained / Introduction / Results / Discussion / Methods / Data Availability / Acknowledgements / Disclosure and Competing Interests Statement / References / Figure Legends Expanded View Figure Legends
- In Methods, statistical paragraph should reflect all information that you have filled in the Authors Checklist, especially regarding randomization, blinding, replication.
- Indicate in legends number and nature of replicates and exact p= values, not a range, along with the statistical test used. To keep the figures "clear" some authors found providing an Appendix table Sx with all exact p-values preferable. You are welcome to do this if you want to.
- Rename "Ethics declaration" to "Disclosure and Competing Interests Statement".
- Author contributions: Please remove it from the manuscript and specify author contributions in our submission system. CRediT has replaced the traditional author contributions section because it offers a systematic machine-readable author contributions format that allows for more effective research assessment. You are encouraged to use the free text boxes beneath each contributing author's name to add specific details on the author's contribution. More information is available in our guide to authors:
https://www.embopress.org/page/journal/17574684/authorguide#authorshipguidelines
- Data availability statement should contain information only about raw data from datasets produced in this study. Datasets obtained from public resources should be cited at the appropriate place in the text (e.g. Methods section) together with the original article and included in the References in form of data citations. Please use the Use the following format to report the accession number of your data:

The datasets produced in this study are available in the following databases:
[data type]: [full name of the resource] [accession number/identifier] ([doi or URL or identifiers.org/DATABASE:ACCESSION])

Please check our Author Guidelines for more information:
https://link.springer.com/journal/44321/submission-guidelines#cms-Reference-guidelines
- Correct the reference citation in the text and reference list. In the text, a reference should be cited by first author and year of publication. In the reference list, where there are more than 10 authors on a paper, 10 will be listed, followed by "et al.". Please check "Author Guidelines" for more information.
https://www.embopress.org/page/journal/17574684/authorguide#referencesformat
3) Appendix: Please upload it as a PDF file.
4) Tables: Please rename Table EV3, EV4, EV5, EV6 to Dataset EV1-EV4 and update their callouts in the main text. Table EV1 and EV2 should remain EV tables, but Table EV1 should be upldd as Expanded View content and the legend should be in the same sheet.
5) The Paper Explained: Please provide "The Paper Explained" and add it to the main manuscript text. Please check "Author Guidelines" for more information. https://www.embopress.org/page/journal/17574684/authorguide#researcharticleguide
6) Synopsis:
- Synopsis image: Please upload the image as a high-resolution jpeg file, exactly 550 px-wide x 300 pixels high.
- Please check your synopsis text and image before submission with your revised manuscript. Please be aware that in the proof stage minor corrections only are allowed (e.g., typos).
7) As part of the EMBO Publications transparent editorial process (see our Editorial at http://embomolmed.embopress.org/content/2/9/329), EMBO Molecular Medicine will publish online a Review Process File (RPF) to accompany accepted manuscripts. This file will be published in conjunction with your paper and will include the anonymous referee reports, your point-by-point response and all pertinent correspondence relating to the manuscript. Let us know if you want to remove or not any figures from it prior to publication. Please note that the Authors checklist will be published at the end of the RPF.

8) Please provide a point-by-point letter INCLUDING my comments as well as the reviewer's reports and your detailed responses (as Word file).

I look forward to reading a new revised version of your manuscript as soon as possible.

Yours sincerely,

Zeljko Durdevic

Zeljko Durdevic
Senior Editor
EMBO Molecular Medicine

*** Instructions to submit your revised manuscript ***

*** PLEASE NOTE *** As part of the EMBO Publications transparent editorial process initiative (see our Editorial at https://www.embopress.org/doi/pdf/10.1002/emmm.201000094), EMBO Molecular Medicine will publish online a Review Process File to accompany accepted manuscripts.

In the event of acceptance, this file will be published in conjunction with your paper and will include the anonymous referee reports, your point-by-point response and all pertinent correspondence relating to the manuscript. If you do NOT want this file to be published, please inform the editorial office at contact@embomolmed.org.

*****

When preparing your revised manuscript, please refer to our guidelines: https://link.springer.com/journal/44321/submission-guidelines#cms-Revised-submissions. We perform an initial quality control of all revised manuscripts before re-review; failure to include requested items will delay the evaluation of your revision.

We require:

1) A .docx formatted version of the manuscript text (including legends for main figures, EV figures and tables). Please make sure that the changes are highlighted to be clearly visible.

2) Individual production quality figure files as .eps, .tif, .jpg (one file per figure). For guidance, download the 'Figure Guide PDF': https://media.springernature.com/original/springer-cms/rest/v1/content/27825798/data/v1.

3) A .docx formatted letter INCLUDING the reviewers' reports and your detailed point-by-point responses to their comments. As part of the EMBO Press transparent editorial process, the point-by-point response is part of the Review Process File (RPF), which will be published alongside your paper.

4) A complete author checklist, which you can download from our author guidelines. Please insert information in the checklist that is also reflected in the manuscript. The completed author checklist will also be part of the RPF.

5) Please note that all corresponding authors are required to supply an ORCID ID for their name upon submission of a revised manuscript.

6) It is mandatory to include a 'Data Availability' section after the Materials and Methods. Before submitting your revision, primary datasets produced in this study need to be deposited in an appropriate public database, and the accession numbers and database listed under 'Data Availability'. Please remember to provide a reviewer password if the datasets are not yet public.

In case you have no data that requires deposition in a public database, please state so in this section. Note that the Data Availability Section is restricted to new primary data that are part of this study.

7) For data quantification: please specify the name of the statistical test used to generate error bars and P values, the number (n) of independent experiments (specify technical or biological replicates) underlying each data point and the test used to calculate p-values in each figure legend. The figure legends should contain a basic description of n, P and the test applied. Graphs must include a description of the bars and the error bars (s.d., s.e.m.).

8) At EMBO Press we ask authors to provide source data for the main manuscript figures. You will receive a separate email with instructions for providing source data with your revised manuscript, including how to upload and organize the files.

9) Our journal encourages inclusion of *data citations in the reference list* to directly cite datasets that were re-used and obtained from public databases. Data citations in the article text are distinct from normal bibliographical citations and should directly link to the database records from which the data can be accessed. In the main text, data citations are formatted as follows: "Data ref: Smith et al, 2001" or "Data ref: NCBI Sequence Read Archive PRJNA342805, 2017". In the Reference list, data citations must be labeled with "[DATASET]". A data reference must provide the database name, accession number/identifiers and a resolvable link to the landing page from which the data can be accessed at the end of the reference.

10) We replaced Supplementary Information with Expanded View (EV) Figures and Tables that are collapsible/expandable online. A maximum of 5 EV Figures can be typeset. EV Figures should be cited as 'Figure EV1, Figure EV2" etc... in the text and their respective legends should be included in the main text after the legends of regular figures.

- For the figures that you do NOT wish to display as Expanded View figures, they should be bundled together with their legends in a single PDF file called *Appendix*, which should start with a short Table of Content. Appendix figures should be referred to in the main text as: "Appendix Figure S1, Appendix Figure S2" etc.

- Additional Tables/Datasets should be labeled and referred to as Table EV1, Dataset EV1, etc. Legends have to be provided in a separate tab in case of .xls files. Alternatively, the legend can be supplied as a separate text file (README) and zipped together with the Table/Dataset file.

11) The paper explained: EMBO Molecular Medicine articles are accompanied by a summary of the articles to emphasize the major findings in the paper and their medical implications for the non-specialist reader. Please provide a draft summary of your article highlighting

- the medical issue you are addressing,

- the results obtained and

- their clinical impact.

This may be edited to ensure that readers understand the significance and context of the research. Please refer to any of our published articles for an example.

12) Author contributions: You will be asked to provide CRediT (Contributor Role Taxonomy) terms in the submission system. These replace a narrative author contribution section in the manuscript.

13) A Conflict of Interest statement should be provided in the main text.

14) Every published paper includes a 'Synopsis' to further enhance discoverability. Synopses are displayed on the journal webpage and are freely accessible to all readers. They include a short stand first (maximum of 300 characters, including space) as well as 2-5 one-sentences bullet points that summarizes the paper. Please write the bullet points to summarize the key NEW findings. They should be designed to be complementary to the abstract - i.e. not repeat the same text. We encourage inclusion of key acronyms and quantitative information (maximum of 30 words / bullet point). Please use the passive voice. Please attach these in a separate file or send them by email, we will incorporate them accordingly.

Please also suggest a striking image or visual abstract to illustrate your article as a PNG file 550 px wide x 300 px high.

15) Include a Reagents and Tools Table as part of the Methods section, which can be downloaded from our author guidelines.

EMBO Molecular Medicine has a "scooping protection" policy, whereby similar findings that are published by others during review or revision are not a criterion for rejection. Should you decide to submit a revised version, I do ask that you get in touch after three months if you have not completed it, to update us on the status.

*Additional important information regarding Figures

Each figure should be given in a separate file and should have the following resolution:

Graphs 800-1,200 DPI
Photos 400-800 DPI
Colour (only CMYK) 300-400 DPI"

Figures are not edited by the production team. All lettering should be the same size and style; figure panels should be indicated by capital letters (A, B, C etc). Gridlines are not allowed except for log plots. Figures should be numbered in the order of their appearance in the text with Arabic numerals. Each Figure must have a separate legend and a caption is needed for each panel.

*Additional important information regarding figures and illustrations can be found at
https://media.springernature.com/original/springer-cms/rest/v1/content/27825798/data/v1

***** Reviewer's comments *****

Referee #2 (Comments on Novelty/Model System for Author):

The authors have addressed many of my concerns by expanding the Methods section, providing uncropped western blots, including basic information on patient samples, expanding the number of different cell lines used as well as the number of patient serum samples examined for eCypA expression.

Referee #2 (Remarks for Author):

1. The authors indicate that three proteins produced by B-ALL cells and regulated by eCypA include CD44, MMP9 and FN2. The gene that produces fibronectin is officially designated FN1. The authors note and cite relevant literature for CD44 and MMP9 in B-ALL, as these are known endogenously produced proteins. For FN they cite a general review. This ECM protein is a known abundant product made by and secreted by bone marrow stromal cells, and is abundantly present in serum (reported in PMID: 7251892). The experiments reported by the authors was presumably done on B-ALL cell lines without the presence of stroma but in the presence of normal amounts of bovine serum.
B-ALL cells are not known producers of FN1 and RNA expression in B-ALL cells is very low. However surprisingly the authors measure robust expression detectable by WB. In the results of qPCR they only report relative values making it impossible to evaluate how much FN mRNA is expressed in the cells. The authors should include the primary data on which the relative expression data are based in the supplemental information.

2. In this revision the authors have tandem sentences with the same meaning:
93 assessed the potential therapeutic relevance of a monoclonal antibody (mAb) targeting
94 CypA. By blocking eCypA, we assessed the potential therapeutic relevance of a
95 monoclonal antibody (mAb) targeting CypA.

368 cells within each state were further stratified based on
369 cell cycle activity into cycling and non-cycling fractions (Appendix Figure S5). Within
370 each state, cells were further stratified based on cell cycle activity into cycling and non
371 cycling fractions,

Referee #3 (Remarks for Author):

This reviewer's main concerns have been sufficiently addressed in the revised manuscript.
Minor: I would advise the authors to clarify a few terms more specifically:
p3 abstract lines 39-40: Be more specific about 'the proportion of specific leukemic cell populations following anti-CypA treatment.' It is unclear what is meant here.
p6 intro lines 92-94: redundant
p6 line 109: The authors should avoid the interchangeable usage of PPIA and CypA. You are referring to the mRNA expression of PPIA and not the gene itself, hence the italicised gene name is not suitable in this context.
p7 lines 122-125: In the results section you state that multivariate analysis identified PPIA as an independent risk factor in all three groups (BM, PB, BM relapse), whereas in your point-by-point reply to the reviewers you wrote independence of PPIA was shown only for BM relapse samples. Please correct accordingly.
p18 lines 364-369 (and p22 lines 449-452): 'up regulation of progenitor-associated genes' is confusing, since you are dealing with B-cell precursor/progenitor (BCP) ALL. You might come up with a more suitable and precise description of the identified

cellular state such as 'multipotent progenitor features'. Strictly speaking, the term 'stemness' (as used in the discussion) also cannot be rigorously applied in this context.
p22 lines 446-448: Fix citations in the main text (first author et al.).

Referee #2 (Comments on Novelty/Model System for Author):

The authors have addressed many of my concerns by expanding the Methods section, providing uncropped western blots, including basic information on patient samples, expanding the number of different cell lines used as well as the number of patient serum samples examined for eCypA expression.

Referee #2 (Remarks for Author):

1. The authors indicate that three proteins produced by B-ALL cells and regulated by eCypA include CD44, MMP9 and FN2. The gene that produces fibronectin is officially designated FN1. The authors note and cite relevant literature for CD44 and MMP9 in B-ALL, as these are known endogenously produced proteins. For FN they cite a general review. This ECM protein is a known abundant product made by and secreted by bone marrow stromal cells, and is abundantly present in serum (reported in PMID: 7251892). The experiments reported by the authors was presumably done on B-ALL cell lines without the presence of stroma but in the presence of normal amounts of bovine serum.

B-ALL cells are not known producers of FN1 and RNA expression in B-ALL cells is very low. However surprisingly the authors measure robust expression detectable by WB. In the results of qPCR they only report relative values making it impossible to evaluate how much FN mRNA is expressed in the cells. The authors should include the primary data on which the relative expression data are based in the supplemental information.

We thank the reviewer for this important comment regarding FN1 expression in B-ALL cells. As the reviewer presumed, our experiments were performed using B-ALL cell lines cultured without stromal cells but in the presence of standard fetal bovine serum. We agree that FN1 is an extracellular matrix protein predominantly produced by bone marrow stromal cells and is abundant in serum. In our qPCR analyses, *FN1* mRNA levels were detectable but low in B-ALL cell lines (Ct values ~29-32), substantially lower than *CD44*. Importantly, the qPCR assay measures cellular mRNA and is not affected by exogenous FN1 present in bovine serum. By contrast, we acknowledge that FN1 protein detection by WB could potentially be influenced by serum-derived FN1, and thus we cannot fully exclude this possibility. Nevertheless, under our experimental conditions, eCypA stimulation consistently increased FN1 levels at both the mRNA and protein levels, supporting an eCypA-associated upregulation of FN1, which was the primary objective of these experiments. In response to the reviewer's suggestion, we have provided the primary qPCR Ct values in the Supplementary Information.

2. In this revision the authors have tandem sentences with the same meaning:
93 assessed the potential therapeutic relevance of a monoclonal antibody (mAb) targeting

94 CypA. By blocking eCypA, we assessed the potential therapeutic relevance of a

95 monoclonal antibody (mAb) targeting CypA.

We have revised the duplicated sentences to improve clarity and readability in the revised manuscript (**Page 7**).

368 cells within each state were further stratified based on
369 cell cycle activity into cycling and non-cycling fractions (Appendix Figure S5). Within

370 each state, cells were further stratified based on cell cycle activity into cycling and non

371 cycling fractions,

We have revised this section to remove the redundant sentence and improve clarity in the revised manuscript (**Page 19**).

Referee #3 (Remarks for Author):

This reviewer's main concerns have been sufficiently addressed in the revised manuscript.

Minor: I would advise the authors to clarify a few terms more specifically:

p3 abstract lines 39-40: Be more specific about 'the proportion of specific leukemic cell populations following anti-CypA treatment.' It is unclear what is meant here.

We thank the reviewer for this helpful suggestion. We have revised the abstract as follows: "Furthermore, single-cell transcriptomics suggests that anti-CypA treatment reduces the proportion of cells with multipotent progenitor features and is accompanied by decreased CREB pathway activation." (**Page 2**)

p6 intro lines 92-94: redundant

We have revised the corresponding sentences to improve conciseness and readability in the revised manuscript (**Page 7**).

p6 line 109: The authors should avoid the interchangeable usage of PPIA and CypA. You are referring to the mRNA expression of PPIA and not the gene itself, hence the italicised gene name is not suitable in this context.

To improve clarity and avoid using PPIA and CypA interchangeably, we revised the relevant text to refer specifically to the "*PPIA* mRNA level" when describing PPIA at the transcriptomic level, thereby clearly distinguishing it from the CypA protein in the revised manuscript.

p7 lines 122-125: In the results section you state that multivariate analysis identified PPIA as an independent risk factor in all three groups (BM, PB, BM relapse), whereas in your point-by-point reply to the reviewers you wrote independence of PPIA was shown only for BM relapse samples. Please correct accordingly.

We apologize for the inaccurate description in our first response letter, and we have revised it as follows:

…… As shown in **Figure 1E**, Kaplan-Meier analysis showed that higher *PPIA* mRNA level was associated with poorer overall survival in the initial BM (p = 0.0163) and relapse BM (p = 0.0025) groups, whereas the association in the initial PB group did not reach statistical significance (p = 0.0638).

Finally, we performed both univariate and multivariate Cox proportional hazards analyses. In the multivariate models, *PPIA* expression (as a continuous variable), age, sex, and fusion status (where available) were included as covariates. These analyses showed that *PPIA* expression was an independent prognostic risk factor in all three groups. The full results are now provided in **Table EV2**.

p18 lines 364-369 (and p22 lines 449-452): 'up regulation of progenitor-associated genes' is confusing, since you are dealing with B-cell precursor/progenitor (BCP) ALL. You might come up with a more suitable and precise description of the identified cellular state such as 'multipotent progenitor features'. Strictly speaking, the term 'stemness' (as used in the discussion) also cannot be rigorously applied in this context.

We agree that the terms “progenitor-associated genes” and “stemness” do not precisely reflect the biological context of BCP-ALL. Accordingly, we have revised the relevant text throughout the manuscript, including the sections highlighted by the reviewer, and have consistently replaced these terms with “multipotent progenitor features” as suggested (**Page 2, 19, 20, 23, and 32**).

p22 lines 446-448: Fix citations in the main text (first author et al.).

The references in the corresponding section have been revised and updated to conform to the reference style of EMBO Molecular Medicine (**Page 23**).

29th May 2026

Dear Dr. Sun,

We are pleased to inform you that your manuscript is accepted for publication and is now being sent to our publisher to be included in the next available issue of EMBO Molecular Medicine.

Your manuscript will be processed for publication by EMBO Press. It will be copy edited and you will receive page proofs prior to publication. Please note that you will be contacted by Springer Nature Author Services to complete licensing and payment information.

You may qualify for financial assistance for your publication charges - either via a Springer Nature fully open access agreement or an EMBO initiative. Check your eligibility: https://link.springer.com/journal/44321/how-to-publish-with-us

Should you be planning a Press Release on your article, please notify the production team via this form in order to coordinate publication and release dates: https://forms.cloud.microsoft/e/eH4A8JVP6g

If you have any questions, please do not hesitate to contact the Editorial Office. Thank you for your contribution to EMBO Molecular Medicine.

Yours sincerely,
Zeljko Durdevic

Zeljko Durdevic
Senior Editor
EMBO Molecular Medicine

>>> Please note that it is EMBO Molecular Medicine policy for the transcript of the editorial process (containing referee reports and your response letter) to be published as an online supplement to each paper. If you do NOT want this, you will need to inform the Editorial Office via email immediately. More information is available here: https://link.springer.com/partners/embo-press/editorial-policies#Peer%20review

EMBO Press Author Checklist

Corresponding Author Name: Sun Lei	
Journal Submitted to: EMBO Molecular Medicine	
Manuscript Number: EMM-2025-22848	

USEFUL LINKS FOR COMPLETING THIS FORM
The EMBO Journal - Author Guidelines
EMBO Reports - Author Guidelines
Molecular Systems Biology - Author Guidelines
EMBO Molecular Medicine - Author Guidelines

Reporting Checklist for Life Science Articles (updated January
This checklist is adapted from Materials Design Analysis Reporting (MDAR) Checklist for Authors. MDAR establishes a minimum set of requirements in transparent reporting in the life sciences (see Statement of Task: 10.31222/osf.io/9sm4x). Please follow the journal's guidelines in preparing your
Please note that a copy of this checklist will be published alongside your article.

Abridged guidelines for figures
1. Data
The data shown in figures should satisfy the following conditions:
- the data were obtained and processed according to the field's best practice and are presented to reflect the results of the experiments in an accurate and unbiased manner.
- ideally, figure panels should include only measurements that are directly comparable to each other and obtained with the same assay.
- plots include clearly labeled error bars for independent experiments and sample sizes. Unless justified, error bars should not be shown for technical
- if n<5, the individual data points from each experiment should be plotted. Any statistical test employed should be justified.
- Source Data should be included to report the data underlying figures according to the guidelines set out in the authorship guidelines on Data

2. Captions
Each figure caption should contain the following information, for each panel where they are relevant:
- a specification of the experimental system investigated (eg cell line, species name).
- the assay(s) and method(s) used to carry out the reported observations and measurements.
- an explicit mention of the biological and chemical entity(ies) that are being measured.
- an explicit mention of the biological and chemical entity(ies) that are altered/varied/perturbed in a controlled manner.
- the exact sample size (n) for each experimental group/condition, given as a number, not a range;
- a description of the sample collection allowing the reader to understand whether the samples represent technical or biological replicates (including how many animals, litters, cultures, etc.).
- a statement of how many times the experiment shown was independently replicated in the laboratory.
- definitions of statistical methods and measures:
  - common tests, such as t-test (please specify whether paired vs. unpaired), simple χ2 tests, Wilcoxon and Mann-Whitney tests, can be unambiguously identified by name only, but more complex techniques should be described in the methods section;
  - are tests one-sided or two-sided?
  - are there adjustments for multiple comparisons?
  - exact statistical test results, e.g., P values = x but not P values < x;
  - definition of 'center values' as median or average;
  - definition of error bars as s.d. or s.e.m.

Please complete ALL of the questions below.
Select "Not Applicable" only when the requested information is not relevant for your study.

Materials

Newly Created Materials	Information included in the manuscript?	In which section is the information available? (Reagents and Tools Table, Materials and Methods, Figures, Data Availability Section)
New materials and reagents need to be available; do any restrictions apply?	Yes	Materials are available from the corresponding author upon reasonable request.

Antibodies	Information included in the manuscript?	In which section is the information available? (Reagents and Tools Table, Materials and Methods, Figures, Data Availability Section)
For **antibodies** provide the following information:<br>- Commercial antibodies: RRID (if possible) or supplier name, catalogue number and or/clone number<br>- Non-commercial: RRID or citation	Yes	Reagents and Tools Table; Materials and Methods (including in-house generated anti-CypA monoclonal antibody)

DNA and RNA sequences	Information included in the manuscript?	In which section is the information available? (Reagents and Tools Table, Materials and Methods, Figures, Data Availability Section)
**Short novel DNA or RNA including primers, probes**: provide the sequences.	Yes	Reagents and Tools Table

Cell materials	Information included in the manuscript?	In which section is the information available? (Reagents and Tools Table, Materials and Methods, Figures, Data Availability Section)
**Cell lines:** Provide species information, strain. Provide accession number in repository **OR** supplier name, catalog number, clone number, and/**OR** RRID.	Yes	Reagents and Tools Table
**Primary cultures:** Provide species, strain, sex of origin, genetic modification status.	Yes	Methods
Report if the cell lines were recently **authenticated** (e.g., by STR profiling) and tested for mycoplasma contamination.	Yes	Methods

Experimental animals	Information included in the manuscript?	In which section is the information available? (Reagents and Tools Table, Materials and Methods, Figures, Data Availability Section)
**Laboratory animals or Model organisms:** Provide species, strain, sex, age, genetic modification status. Provide accession number in repository **OR** supplier name, catalog number, clone number, **OR** RRID.	Yes	Reagents and Tools Table
**Animal observed in or captured from the field:** Provide species, sex, and age where possible.	Not Applicable	
Please detail **housing and husbandry conditions**.	Yes	Methods

Plants and microbes	Information included in the manuscript?	In which section is the information available? (Reagents and Tools Table, Materials and Methods, Figures, Data Availability Section)
**Plants:** provide species and strain, ecotype and cultivar where relevant, unique accession number if available, and source (including location for collected wild specimens).	Not Applicable	
**Microbes:** provide species and strain, unique accession number if available, and source.	Not Applicable	

Human research participants	Information included in the manuscript?	In which section is the information available? (Reagents and Tools Table, Materials and Methods, Figures, Data Availability Section)
If collected and within the bounds of privacy constraints report on age, sex and gender or ethnicity for all study participants.	Yes	Methods

Core facilities	Information included in the manuscript?	In which section is the information available? (Reagents and Tools Table, Materials and Methods, Figures, Data Availability Section)
If your work benefited from core facilities, was their service mentioned in the acknowledgments section?	Yes	Acknowledgements

Design

Study protocol	Information included in the manuscript?	In which section is the information available? (Reagents and Tools Table, Materials and Methods, Figures, Data Availability Section)
If study protocol has been **pre-registered, provide DOI in the manuscript**. For clinical trials, provide the trial registration number **OR** cite DOI.	Not Applicable	
Report the **clinical trial registration number** (at ClinicalTrials.gov or equivalent), where applicable.	Not Applicable	

Laboratory protocol	Information included in the manuscript?	In which section is the information available? (Reagents and Tools Table, Materials and Methods, Figures, Data Availability Section)
Provide DOI OR other citation details if **external detailed step-by-step protocols** are available.	Not Applicable	

Experimental study design and statistics	Information included in the manuscript?	In which section is the information available? (Reagents and Tools Table, Materials and Methods, Figures, Data Availability Section)
Include a statement about **sample size** estimate even if no statistical methods were used.	Yes	Methods; Figure Legends
Were any steps taken to minimize the effects of subjective bias when allocating animals/samples to treatment (e.g. **randomization procedure**)? If yes, have they been described?	Yes	Methods
Include a statement about **blinding** even if no blinding was done.	Yes	Methods
Describe **inclusion/exclusion criteria** if samples or animals were excluded from the analysis. Were the criteria pre-established?<br><br>If sample or data points were omitted from analysis, report if this was due to attrition or intentional exclusion and provide justification.	Yes	Methods
For every figure, are **statistical tests** justified as appropriate? Do the data meet the assumptions of the tests (e.g., normal distribution)? Describe any methods used to assess it. Is there an estimate of variation within each group of data? Is the variance similar between the groups that are being statistically compared?	Yes	Figure Legends; Methods

Sample definition and in-laboratory replication	Information included in the manuscript?	In which section is the information available? (Reagents and Tools Table, Materials and Methods, Figures, Data Availability Section)
In the figure legends: state number of times the experiment was **replicated** in laboratory.	Yes	Figure Legends
In the figure legends: define whether data describe **technical or biological replicates**.	Yes	Figure Legends

Ethics

Ethics	Information included in the manuscript?	In which section is the information available? (Reagents and Tools Table, Materials and Methods, Figures, Data Availability Section)
Studies involving **human participants**: State details of **authority granting ethics approval** (IRB or equivalent committee(s), provide reference number for approval.	Yes	Methods
Studies involving **human participants**: Include a statement confirming that **informed consent** was obtained from all subjects and that the experiments conformed to the principles set out in the WMA Declaration of Helsinki and the Department of Health and Human Services Belmont Report.	Yes	Methods
Studies involving **human participants:** For publication of **patient photos**, include a statement confirming that consent to publish was obtained.	Not Applicable	
Studies involving experimental **animals**: State details of **authority granting ethics approval** (IRB or equivalent committee(s), provide reference number for approval. Include a statement of compliance with ethical regulations.	Yes	Methods
Studies involving **specimen and field samples:** State if relevant **permits** obtained, provide details of authority approving study; if none were required, explain why.	Not Applicable	

Dual Use Research of Concern (DURC)	Information included in the manuscript?	In which section is the information available? (Reagents and Tools Table, Materials and Methods, Figures, Data Availability Section)
Could your study fall under dual use research restrictions? Please check biosecurity documents and list of **select agents and toxins** (CDC): https://www.selectagents.gov/sat/list.htm	Not Applicable	
If you used a select agent, is the security level of the lab appropriate and reported in the manuscript?	Not Applicable	
If a study is subject to dual use research of concern regulations, is the name of the **authority granting approval and reference number** for the regulatory approval provided in the manuscript?	Not Applicable	

Reporting

The MDAR framework recommends adoption of discipline-specific guidelines, established and endorsed through community initiatives. Journals have their own policy about requiring specific guidelines and recommendations to complement MDAR.

Adherence to community standards	Information included in the manuscript?	In which section is the information available? (Reagents and Tools Table, Materials and Methods, Figures, Data Availability Section)
State if relevant guidelines or checklists (e.g., **ICMJE, MIBBI, ARRIVE, PRISMA**) have been followed or provided.	Yes	Methods
For **tumor marker prognostic studies,** we recommend that you follow the **REMARK** reporting guidelines (see link list at top right). See author guidelines, under 'Reporting Guidelines'. Please confirm you have followed these guidelines.	Not Applicable	
For **phase II and III randomized controlled trials**, please refer to the **CONSORT** flow diagram (see link list at top right) and submit the CONSORT checklist (see link list at top right) with your submission. See author guidelines, under 'Reporting Guidelines'. Please confirm you have submitted this list.	Not Applicable	

Data Availability

Data availability	Information included in the manuscript?	In which section is the information available? (Reagents and Tools Table, Materials and Methods, Figures, Data Availability Section)
Have **primary datasets** been deposited according to the journal's guidelines (see 'Data Deposition' section) and the respective accession numbers provided in the Data Availability Section?	Yes	Data Availability Section
Were **human clinical and genomic datasets** deposited in a public access-controlled repository in accordance to ethical obligations to the patients and to the applicable consent agreement?	Yes	Data Availability Section
Are **computational models** that are central and integral to a study available without restrictions in a machine-readable form? Were the relevant accession numbers or links provided?	Not Applicable	
If publicly available data were reused, provide the respective **data citations in the reference list**.	Yes	Materials and Methods; References
