# Supplementary material for: Extracellular cyclophilin A promotes B-cell acute lymphoblastic leukemia progression via MC2R
Source: EMBO Mol Med. 2026 Jun 10;18(8):3028–56. doi: 10.1038/s44321-026-00466-w (PMC13469882; doi:10.1038/s44321-026-00466-w)
Supplement: Supplementary file 4 [file 44321_2026_466_MOESM2_ESM.docx]

Table EV2. Multivariate Cox regression analysis of overall survival in B-ALL patients based on initial and relapse bone marrow samples

| **Variable** | **Initial BM HR (95% CI)** | **P value** | **Relapse BM HR (95% CI)** | **P value** | **Initial PB HR (95% CI)** | **P value** |
| --- | --- | --- | --- | --- | --- | --- |
| *PPIA* mRNA expression | 2.05 (1.38–3.03) | <0.001 | 1.80 (1.14–2.82) | 0.011 | 1.92 (1.08–3.42) | 0.026 |
| Age | 1.07 (1.02–1.13) | 0.009 | 1.04 (0.98–1.11) | 0.184 | 1.07 (0.99–1.16) | 0.079 |
| Gender (Male vs Female) | 1.50 (0.80–2.82) | 0.207 | 2.23 (1.20–4.17) | 0.012 | 0.88 (0.38–2.03) | 0.762 |
| Fusion status | 1.45 (0.65–3.24) | 0.367 | 1.25 (0.59–2.69) | 0.561 | 1.29 (0.53–3.15) | 0.570 |

Initial BM: n = 105, events = 42; Relapse BM: n = 74, events = 47; Initial PB: n = 50, events = 26

Table EV2. Multivariate Cox proportional hazards regression analysis of overall survival in B-ALL patients from the TARGET-ALL-P2 cohort stratified by sample source and disease stage. *PPIA* mRNA expression was analyzed as a continuous variable. Age, gender, and fusion status were included as covariates where available.

Abbreviations: BM, bone marrow; PB, peripheral blood; HR, hazard ratio; CI, confidence interval.
